# Breaking the activity-selectivity trade-off in Fenton-like catalysis by d-orbital modulation of single-atom sites within a nano-island-like structure

**DOI:** 10.1038/s41467-026-74072-2

**Published:** 2026-06-08

**Authors:** Yuxin Chen, Xing Xu, Jianrong Zeng, Yang Yu, Yingshuai Ma, Peilin Zhang, Tao Zeng, Haiguang Zhang, Liang Tang, Runzeng Liu, Youcai Zhu

**Affiliations:** 1 Shandong Key Laboratory of Environmental Processes and Health, School of Environmental Science and Engineering, Shandong University, Qingdao, P. R. China; 2 Shanghai Synchrotron Radiation Facility, Shanghai Advanced Research Institute, Chinese Academy of Sciences, Shanghai, P. R. China; 3 Shanghai Institute of Applied Physics, Chinese Academy of Sciences, Shanghai, P. R. China; 4 Zhejiang Key Laboratory of Environment and Health of New Pollutants, School of Environment, Hangzhou Institute for Advanced Study, University of Chinese Academy of Sciences, Hangzhou, P. R. China; 5 Key Laboratory of Organic Compound Pollution Control Engineering (MOE), School of Environmental and Chemical Engineering, Shanghai University, Shanghai, China

**Keywords:** Pollution remediation, Materials for energy and catalysis

## Abstract

Direct electron transfer (ETP) during peroxymonosulfate (PMS) activation enables selective, matrix-resistant organic contaminants oxidation, yet its precise control over competing radical pathways remains elusive. Here we report a nano-island-like single-atom catalyst- carbon nitride islands immobilize cobalt single atoms on reduced graphene oxide (CoN_3_C/rGO)- that leverages an island-sea architecture to direct PMS activation toward ETP. Experimental and density functional theory (DFT) analyses show an rGO induced elevation of the Co *d*-band center and a sharpened *d*_*z2*_ orbital near the Fermi level, promoting directional hybridization with PMS *p* orbitals and suppressing antibonding occupation. Consequently, CoN_3_C/rGO/PMS degrade bisphenol A (BPA) completely within 5 min, with ~94% contribution from ETP. Furthermore, catalytic membrane coatings enable stable 100 h continuous operation in diverse real water matrices with minimal Co leaching. Our results demonstrate a design principle-orbital-level modulation via island-sea architectures to reconcile activity and selectivity in Fenton-like systems and advance translating practical water treatment technologies based on single-atom electronic control.

## Abstract (web summary)

A nano-island-like catalyst (CoN₃C/rGO) modulates cobalt d-orbitals to break the activity-selectivity trade-off in Fenton-like reactions, enabling direct electron transfer for efficient pollutant degradation and stable water treatment.

## Introduction

Heterogeneous peroxymonosulfate (PMS) activation has evolved from traditional radical-based processes to more selective non-radical pathways^1^. Among these, the direct electron transfer pathway (ETP) has emerged as a pivotal non-radical mechanism in PMS based advanced oxidation processes (PMS-AOPs), distinguished by its triple merits: (1) Compared with radical pathways: Superior resistance to matrix interference (e.g., anions, humic acid matter), higher oxidant utilization efficiency, and low damage to catalyst; (2) Compared with other non-radical pathways (e.g., singlet oxygen, high valent metal-oxo): Stronger selectivity, and reaction kinetics independent of diffusion distances or short-lived species^2–5^. Moreover, this mechanism makes it possible to avoid directly introducing PMS into the wastewater, as the purpose of electron transfer can be achieved through the salt bridge device, avoiding the environmental problem resulting from SO_4_^2-^ ^6,7^. The current main activation process of PMS depends on multivalent transition metals (TMs) catalysts^8^. However, although many research has achieved the regulation of the free radical or non-free radical pathways during the PMS activation process through TMs catalysts, research is scarce on the targeted regulation of the ETP mechanism. Thus, the targeted design with ETP as the main mechanism in AOPs is extremely necessary for addressing the increasingly serious water resource issues.

The main obstacle in obtaining ETP with high selectivity is the low dissociation energy of the O–O bond in PMS, which means when only one electron is obtained, the O–O bond can be broken, and a free radical would be generated^9^. In the catalytic process, the compromised selectivity despite high activity is partially attributed to the broad energy distribution of elevated *d*-orbital density near the Fermi level, which facilitates indiscriminate hybridization with multiple molecular orbitals of substrates^10^. Among them, the *d*_*z2*_ orbital, which is perpendicular to the basal plane, can maximize the orbital overlap during the bonding process, thereby facilitating the electron transfer at the interface^11^. Therefore, precisely modulating the occupancy range and intensity of *d*_*z2*_ orbital electrons at metal sites could resolve the issue of stochastic single-electron injection into O–O antibonding orbitals, thereby enabling highly selective ETP.

Single-atom catalysts (SACs) provide a possibility for this precise regulation at orbital level. Research has shown the potential to alter the center and the width of the *d* band by regulating the interaction between metal atoms and supports of SACs, thereby achieving the modulation of electrons on *d* orbitals^12^. However, previous studies mainly focused on modulating metal-support interactions through coordination number and ligand type adjustments at the atomic scale^13–16^. Such precise control over the coordination environment remains technically demanding and often lacks accuracy. The emergence of nano-island catalysts has made it possible to achieve regulation from another perspective. Nano-island catalysts, where single atom sites on small functional supports (“islands”) are dispersed across a high-surface-area support (“sea”), have emerged as a promising platform for balancing catalytic activity and stability^17,18^. However, beyond this structural role, the “sea” support can profoundly influence the electronic and orbital structure of the atoms loaded at “islands” through the metal-support interaction^19–21^. Hence, we propose that utilizing the island-sea synergistic effect offers a promising strategy for precisely modulating the *d* orbital electrons of metal catalytic centres, providing creative insights into selectively steering the ETP path in Fenton-like systems. The realization of this concept relies on the design of island-sea catalysts. Due to the well-defined nitrogen-rich motifs that can firmly anchor transition metal atoms via coordination, creating stable and atomically dispersed active sites, carbon nitride has potential to be the “island”^22^. Meanwhile, the advantages that superior electrical conductivity, large specific surface area, and a delocalized π-electron system that can potentially modulate the electronic structure of the metal centers over long range makes reduced graphene oxide (rGO) would be a candidate of “sea”^23,24^.

Here, we utilize a cobalt single atom catalyst anchored on carbon nitride supported by reduced graphene oxide (CoN_3_C/rGO) to activate PMS, achieving a synergistic enhancement of activity and selectivity through an island-sea effect in this nano-island-like structure. The carbon nitride as the “islands” and rGO as the “sea”. Utilizing the asymmetric coordination configuration to promote charge distribution and construct a highly active site. The abundant delocalized π electrons in rGO are utilized to regulate the electronic and energy band structures of the Co atom. The synthesized CoN_3_C/rGO can achieve robust PMS activation performance compared to CoN_3_C and most of the previously reported catalysts. In the CoN_3_C/rGO/PMS system, the contribution of ETP reached about 94%. Density functional theory (DFT) calculations revealed that compared with CoN_3_C, asymmetric electronic distribution, higher *d*-band center, and sharper *d*_*z2*_ orbital electron occupation states enhance the activity and selectivity of CoN_3_C/rGO/PMS system. These advantages provide a remarkable degradation efficiency for electron-donating contaminants in CoN_3_C/rGO/PMS system. This work achieved the high selectivity of ETP path generation from the perspective of electron and orbit control, providing new insights for the reactive oxygen species (ROS) directed regulation of the AOPs.

## Results

### Synthesis and characterization

**Fig. 1:**
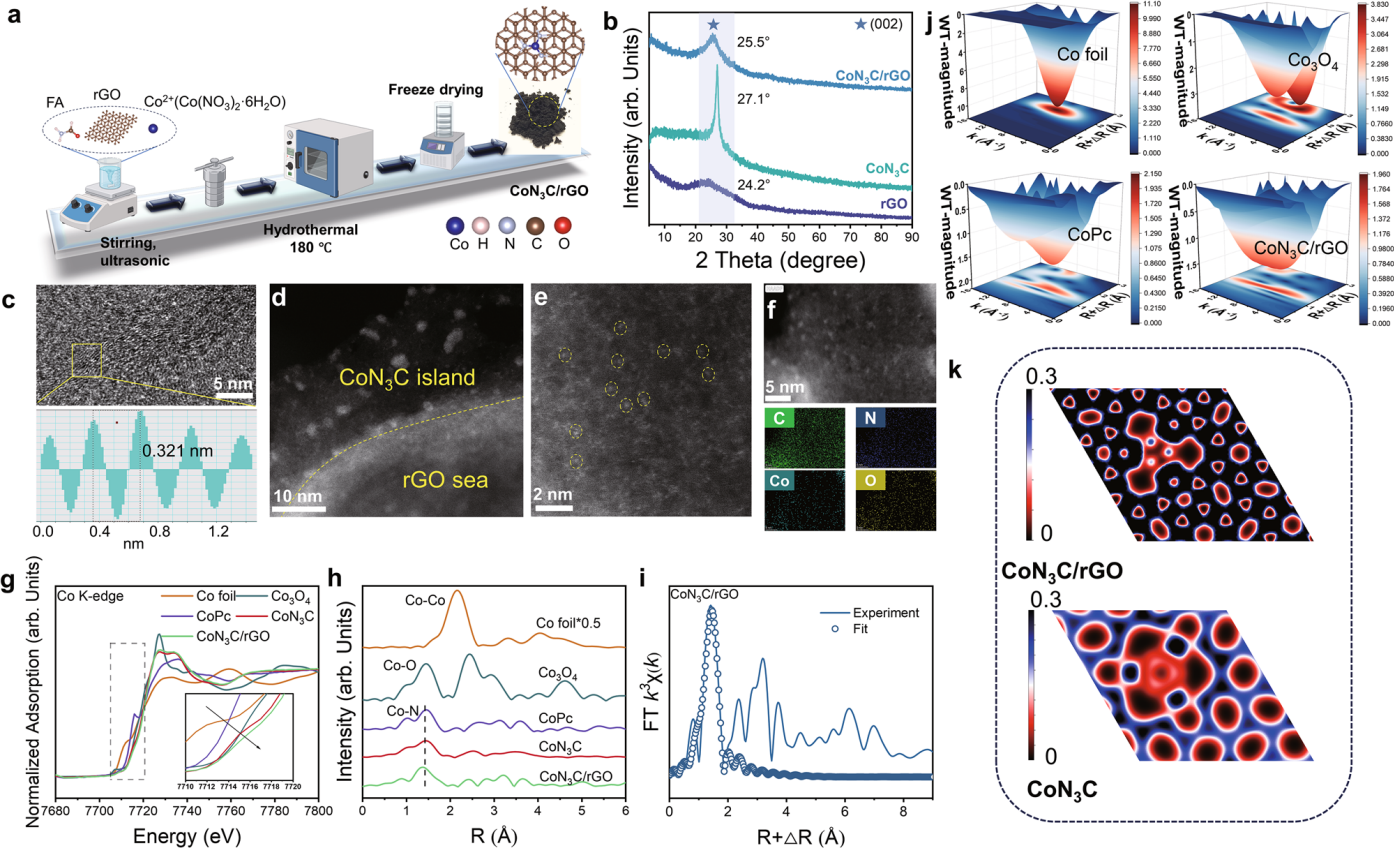
Synthesis and microscopic structures of catalysts. **a** Synthesis procedure of CoN_3_C/rGO. “*Stirrer, hydrothermal reactor, oven, and cold dryer*” adapted from BioRender. Zhu, Y. (2026). **b** XRD pattern of different catalysts. **c** Interlayer spacing of CoN_3_C island (The area within the yellow box is where the inverse Fourier transform is performed to calculate the interlayer spacing). **d**,** e** AC-HAADF-STEM images of CoN_3_C/rGO (Co single atoms are marked by yellow circles). **f** EDS-mapping of CoN_3_C/rGO. **g** Normalized Co k-edge XANES of CoN_3_C/rGO, CoN_3_C and reference samples. **h** Fourier transform EXAFS of CoN_3_C/rGO, CoN_3_C and reference samples. **i** Co k-edge EXAFS fitting analysis of CoN_3_C/rGO in R space. **j** Wavelet transforms of Co foil, Co_3_O_4_, CoPc, and CoN_3_C/rGO. **k** The two-dimensional electron localization functions of CoN_3_C/rGO and CoN_3_C. Source data are provided as a Source data file.

The formamide hydrothermal method was employed to synthesize CoN_3_C and CoN_3_C/rGO (Fig. 1a). The X-ray diffraction (XRD) patterns reveal a critical structural evolution upon compositing (Fig. 1b). Unlike the physical mixture, the CoN_3_C/rGO composite exhibits a distinct diffraction peak at 2θ = 25.5°, which sits between the characteristic peaks of rGO (24.2°) and CoN_3_C (27.1°), suggesting the original highly crystalline structure of CoN_3_C was disrupted and interfaced with rGO in a small-sized and low-stacked manner^25^. The results of Raman spectra and Fourier-transform infrared (FTIR) further demonstrate the formation of nano-island interface and the resulting local structural changes from the perspectives of chemical bond and defect level (Supplementary Figs. 1 and 2). The electronic states and surface elements content of the prepared catalysts were analyzed by X-ray photoelectron spectra (XPS) (Supplementary Figs. 3–6 and Supplementary Table 3). More pronounced N-C=N peak appears at CoN_3_C/rGO than CoN_3_C, indicating the existence of rGO anchors and disperses the CoN_3_C “islands”, which maximizes the exposure of the N-C=N coordination network to the surface. Compared with CoN_3_C, the negative shift in the N-C=N skeleton and positive shifts in the Co 2*p* and pyridinic N of CoN_3_C/rGO suggests an electron injection from the conductive rGO “sea” to the electron-deficient carbon nitride “islands”, confirming a significant interfacial electronic coupling.

In the scanning electron microscopic (SEM) and transmission electron microscopy (TEM) images, the pristine rGO presents a smooth and sheet-like structure, limiting mass transfer (Supplementary Figs. 7 and 8). Conversely, the pure CoN_3_C exhibits an irregular, and aggregated morphology, typical of bulk carbon nitride, which severely buries the active Co sites. Upon hydrothermal process, the bulky CoN_3_C aggregates completely disappear, replaced by ultra-thin nano-island uniformly dispersed on the rGO surface. This further indicates that the rGO substrate effectively exfoliates and anchors the CoN_3_C precursors, preventing their self-aggregation. And these rigid islands induce significant wrinkling of the rGO sheets. SEM–energy dispersive spectroscopy (EDS) mapping verifies the homogeneous distribution of C, N, O, Co and low content of Co in catalysts (Supplementary Figs. 9–14 and Supplementary Tables 4–6). In the high-resolution TEM (HRTEM) results, the formation of carbon nitride islands is confirmed by layer spacing of 0.321 nm (Fig. 1c)^26^. The CoN_3_C/rGO exhibits a highly disordered and distorted lattice structure, indicating the growth of CoN_3_C islands induces significant lattice strain and defects on the rGO surface, which might facilitate the electron transfer capacity. A clear boundary line was obtained between the CoN_3_C island and the rGO sea in high-angle annular dark-field scanning transmission electronmicroscopy (HAADF-STEM) (Fig. 1d). As shown in Fig. 1e, the isolated bright spots provides the evidence of atomically dispersed Co sites in CoN_3_C/rGO. In addition to the uniform distribution of elements on smaller scales, STEM-EDS mapping also reflects the low content of Co (Fig. 1f). The exact Co loading content of catalysts was further detected by atomic adsorption spectroscopy (AAS), which showed CoN_3_C and CoN_3_C/rGO contain 1.97% and 1.25% Co, respectively (Supplementary Table 7).

The valence state and chemical coordination environment of Co were further studied by X-ray absorption fine structure (XAFS). In the X-ray absorption near-edge structure (XANES) spectroscopy, the adsorption of CoN_3_C/rGO and CoN_3_C is very near to Co_3_O_4_, indicating the valence of Co in two catalysts is between +2 and +3 (Fig. 1g). The specific valence state is shown in linear combination fitting (LCF) results, which the proportion of Co^2+^/Co^3+^ of CoN_3_C/rGO (0.55/0.45) and CoN_3_C (0.56/0.44) is similar, indicating that the introduction of the rGO sea did not drastically alter the average oxidation state of Co (Supplementary Fig. 15). The extended X-ray absorption fine structure (EXAFS) spectra reveals that no Co–Co interaction exists in CoN_3_/rGO and CoN_3_C, while obvious Co–N can be observed at 1.4–1.6 Å, further demonstrating the single atom form of Co (Fig. 1h). The wavelet transform (WT) EXAFS shows a peak at about 5 Å in k space, which is also ascribed to Co–N (Fig. 1j and Supplementary Fig. 16)^27^. EXAFS fitting results indicate that Co atoms were coordinated with three N atoms in the first shell of CoN_3_C/rGO and CoN_3_C (Fig. 1i and Supplementary Figs. 17 and 18). Meanwhile, the bond length of Co–N is 1.98 Å in two catalysts, which means Co and N are more likely to exist in planar triangular configurations (Supplementary Table 8). The electronic structure of CoN_3_C/rGO and CoN_3_C was gained by DFT calculations. The Electron Localization Function (ELF) indicates the existence of the π-electron structure of rGO, causing the electrons around Co atom in CoN_3_C/rGO to exhibit an asymmetric distribution (Fig. 1k and Supplementary Fig. 19). This asymmetry will further facilitate the electron transfer at the interface and enhance the catalytic performance^28^. Furthermore, the electrostatic potential (ESP) results show that rGO shifts the ESP positively of the Co center, which would enhance the interaction between Co site in CoN_3_C/rGO with HSO_4_^−^ in PMS (Supplementary Fig. 20).

### Performance assessment of nano-island-like catalyst

**Fig. 2:**
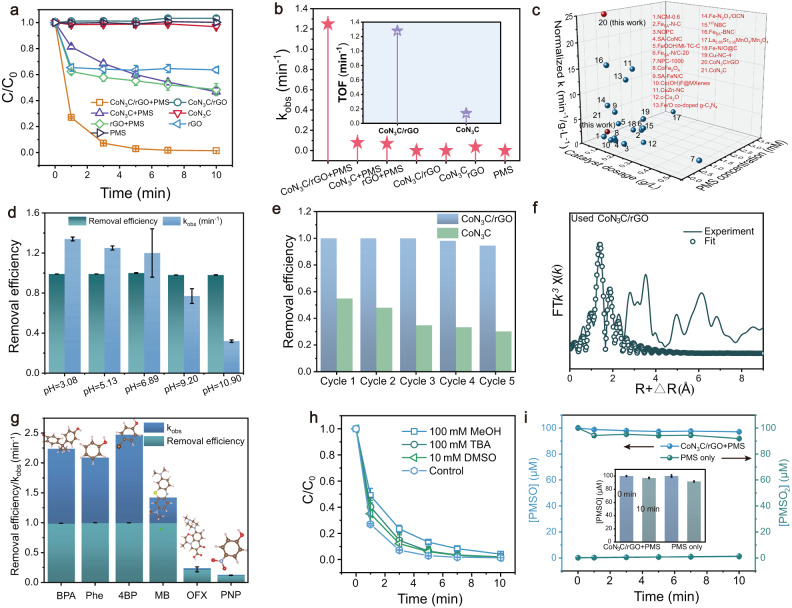
Catalytic performance and ROS generation of different systems. **a** Degradation curves of BPA in different systems. **b** *k*_*obs*_ and TOF of different catalyst systems. **c** The normalized *k*_*obs*_ of BPA in different reported catalysts mediated PMS activation systems (The relevent references are listed in Supplementary Table 9). **d** The effect of initial pH for CoN_3_C/rGO/PMS system (The removal efficiency and *k*_*obs*_ share the same scale). **e** Cyclic experiment of CoN_3_C/rGO/PMS and CoN_3_C/PMS system. **f** Co k-edge EXAFS fitting analysis of used CoN_3_C/rGO in R space. **g** Removal effect of CoN_3_C/rGO/PMS for different contaminants. **h** Quenching experiment of CoN_3_C/rGO/PMS. **i** PMSO conversion results of CoN_3_C/rGO/PMS system. Experiment conditions: [catalyst]= 0.05 g/L, [PMS] = 0.5 mM, [BPA] = 10 mg/L, pH = 6.0, room temperature if not otherwise specified. The error bars are standard deviation of three replicate tests (*n* = 3). Source data are provided as a Source data file.

Systematic optimization of the hydrothermal conditions and rGO loading revealed that the CoN_3_C/rGO catalyst prepared at 180 °C with 0.1 g rGO exhibits optimal PMS activation performance (Supplementary Figs. 21–27). This optimal synthesis window maximizes the formation of electron-rich pyrrolic N defects and creates the most electron-donating Co centers, as evidenced by XPS, which underpins its superior activity and stability. The oxidizing capability of CoN_3_C/rGO/PMS system was evaluated by degradation performance for bisphenol A (BPA). The catalysts before and after compositing exhibited significant differences in the activation of PMS. As shown in Fig. 2a, almost no BPA was removed within 10 min when only PMS, CoN_3_C/rGO, or CoN_3_C was added. In contrast, about 35% BPA could be adsorbed by rGO, while this effect disappeared after forming composite, which might be due to the rGO layer spacing being reduced and the surface being covered by islands. After adding PMS, the removal efficiency of BPA rose to about 50%, indicating the weak activation effect of rGO to PMS. Similar data was obtained in CoN_3_C/PMS system. However, in the CoN_3_C/rGO/PMS system, the BPA was degraded completely only within 5 min. The degradation rate of BPA in different systems was evaluated by calculating reaction rate constant (*k*_*obs*_) (Fig. 2b). The CoN_3_C/rGO/PMS shows the highest *k*_*obs*_ for BPA degradation, which is 15-fold, 18-fold, and 34-fold enhancement over CoN_3_C/PMS, rGO/PMS, and rGO only systems, respectively. Additionally, CoN_3_C/rGO exhibits 1.28 min^−1^ of turnover frequency (TOF), which is about 9 times higher than CoN_3_C, demonstrating the island-sea effect greatly improved the activity of per Co site. The PMS utilization efficiency is also an important index to reflect the performance of catalysts (Supplementary Fig. 28). In the CoN_3_C/rGO/PMS system, about 74% of PMS would be decomposed within 10 min, while only 37% and 13% of PMS were utilized effectively for CoN_3_C/PMS and rGO/PMS. Hence, sole CoN_3_C or rGO would cause higher oxidant waste. In conclusion, CoN_3_C/rGO exhibits the highest reactivity in the Fenton-like reaction, and the degradation effect of BPA didn’t include adsorption or PMS self-decomposition. As evidenced by normalized *k*_*obs*_, CoN_3_C/rGO exhibits superior activity for BPA degradation compared to most of the previously reported single atom catalysts and other catalysts (Fig. 2c and Supplementary Table 9). Additionally, the effect of PMS concentration and catalyst dosage is also studied systematically (detailed discussion is in Supplementary Figs. 29–30). In the range of 3.0–11.0, almost 100% removal efficiency could be achieved within 10 min through CoN_3_C/rGO activating PMS, even though the *k*_*obs*_ decreased in the strong base condition, exhibiting a broad pH adaptability (Fig. 2d and Supplementary Fig. 31).

The Co leaching was analyzed through the whole degradation process, which was far lower than Chinese Environmental Quality standards for surface water of 1 mg/L, showing the environmental safety of CoN_3_C/rGO (Supplementary Fig. 32). Additionally, five cyclic performance tests were conducted under the same conditions (Fig. 2e). During all cycles, the BPA removal efficiency remained above 90%, indicating the carbon nitride island provide the robust cyclic stability of the central Co atom. The AAS results show that the Co loading decreased from 1.25 wt% to 0.8 wt% after the stability test, which is likely attributed to the leaching of some loosely bound or unstable Co species during the initial stage of the reaction (Supplementary Table 7). However, the subsequent stable catalytic performance indicates that the core Co-N_x_ active sites remain robust. The EXAFS and Raman spectra of the used CoN_3_C/rGO reveal that no significant changes in the coordination environment, graphitization degree and defect density of the catalyst (Fig. 2f and Supplementary Figs. 33–34). The high-resolution XPS spectra reveal negligible shifts in the binding energies of C 1 s and N 1 s after the reaction, confirming the stability of catalyst structure (Supplementary Fig. 35). In comparison, only 30% of BPA could be degraded within the same time in CoN_3_C/PMS system. Analysis of Co leaching concentrations after each cycle revealed no detectable cobalt release beyond the second cycle, also demonstrating the stability of CoN_3_C/rGO (Supplementary Fig. 36). Moreover, 6 kinds of pollutants, including BPA, ofloxacin (OFX), 4-Bromophenol (4BP), methylene blue (MB), phenol (Phe), p-Nitrophenol (PNP) as target to be degraded to explore the general applicability of CoN_3_C/rGO/PMS (Fig. 2g and Supplementary Fig. 37), while result showing the system has selectivity for different contaminants.

### Selectivity of ETP in CoN_3_C/rGO/PMS system

To verify the contribution of various ROS paths, the quenching experiment and EPR analysis were conducted. Methanol (MeOH), tert-butanol (TBA), p-benzoquinone (p-BQ), L-histidine (L-his), and dimethyl sulfoxide (DMSO) were used as quenching agents for ·OH, SO_4_^·−^, O_2_^·−^, ^1^O_2_, and Co(IV) = O (Fig. 2h and Supplementary Fig. 38). In CoN_3_C/rGO/PMS system, a negligible inhibition effect occurred after adding MeOH, TBA, p-BQ, or DMSO. Combined with EPR results, in the 5,5-dimethyl-1-pyrroline-N-oxide (DMPO)/water system, a typical seven-line peak represents DMPOX, which was a result of oxidation of strong oxidized species generated (Supplementary Fig. 39a)^29^. After filtering, no signals of ·OH and SO_4_^·−^ were recorded (Supplementary Fig. 39b). In addition, no O_2_^·−^ signal was generated in DMPO/MeOH system, further excluding the role of ·OH, SO_4_^·−^, O_2_^·−^ (Supplementary Fig. 39c). The specific contribution of Co(IV) = O was verified by PMSO oxidation experiment (Fig. 2i). Nearly no PMSO was oxidized to PMSO_2_ in sole PMS and CoN_3_C/rGO/PMS system, demonstrating that no Co(IV) = O was generated in CoN_3_C/rGO/PMS system. The quenching results show that L-his hardly inhibited the BPA degradation process, and EPR spectra only exhibit a very weak 1:1:1 signal of ^1^O_2_, indicating nearly no contribution of ^1^O_2_ (Supplementary Fig. 39d)^30^. In contrast, the addition of L-his and p-BQ significantly inhibited BPA degradation in the CoN_3_C/PMS system. Furthermore, EPR spectroscopy detected signals corresponding to O_2_^·−^ and ^1^O_2_, verifying their pivotal roles in the process. The presence of KSCN, a representative cobalt complexing agent, inhibit the BPA degradation completely, indicating Co atom is the active site in the activation of PMS (Supplementary Fig. 40).

**Fig. 3:**
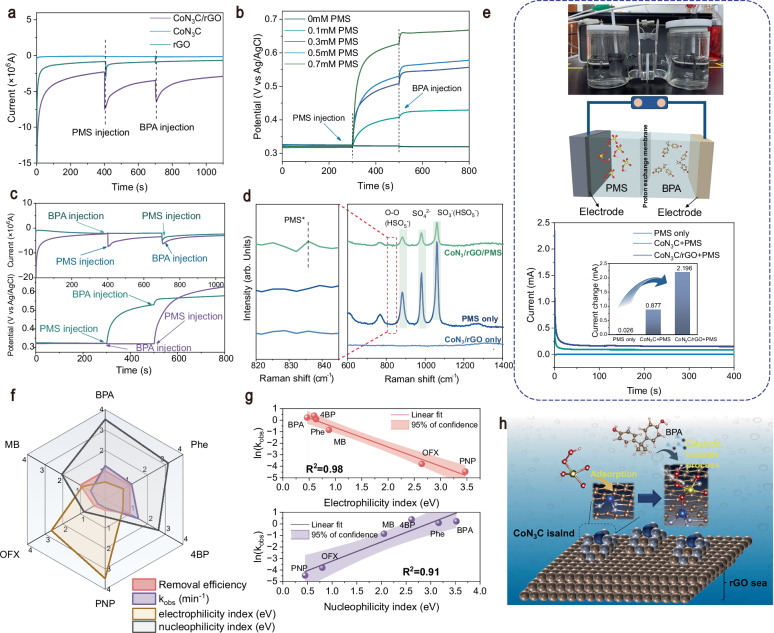
The direct electron transfer phenomenon of CoN_3_C/PMS system. **a** i-t curves upon the addition of PMS and BPA. **b** OCP changes under different PMS concentrations. **c** i-t and OCP upon changing the addition sequences of PMS and BPA for CoN_3_C/rGO/PMS system (Green and purple curves represent the results that different addition sequences). **d** In situ Raman spectra of CoN_3_C/rGO/PMS system (The area within the red box represents the region where the PMS peak appears). **e** Schematic diagram and current changes of two-cell experiment. **f** The relationship between degradation effect and electronic structure of various pollutants. **g** Quantitative structure-activity relationship (QSAR) between electrophilicity/nucleophilicity index of different pollutants and their ln(*k*_*obs*_) (The removal efficiency, *k*_*obs*_, and electrophilicity/nucleophilicity index share the same scale). **h** The ETP dominant path in CoN_3_C/rGO/PMS system, which is achieved through island-sea effect. The error bars are standard deviation of three replicate tests (*n* = 3). Source data are provided as a Source data file.

The ETP mechanism was demonstrated by an electrochemical test. As shown in Supplementary Fig. 41, the PMS addition has an obvious influence on OCP, and a significant increase in potential occurs after the addition of BPA. A similar phenomenon is shown in i-t curve and LSV results, the injection of PMS and BPA caused the rise of current, indicating ETP occurred in CoN_3_C/rGO/PMS system (Fig. 3a and Supplementary Fig. 42). Additionally, the increased dosage of PMS correlates with amplified potential shifts during the reaction (Fig. 3b). To clarify the reaction sequence, the addition order of PMS and BPA was changed. If PMS was added first, the above results could be observed, while when BPA was introduced initially, no significant current variation was detected until PMS was subsequently added, after which distinct potential and current changes emerged (Fig. 3c). The conclusion was also demonstrated by in situ Raman spectra, the peak of PMS became weaker after mixing with CoN_3_C/rGO, while a distinct peak at 836 cm^−1^ was shown, corresponding to CoN_3_C/rGO-PMS* (Fig. 3d)^31^. The ETP mechanism was further elucidated by two-cell experiment. The BPA and PMS were isolated in two separated cells with a proton exchange membrane to maintain the balance of charge and pH. When no catalyst was coated on the electrode, no current change occurred, while an obvious change could be observed after using CoN_3_C/rGO or CoN_3_C coated electrode (Fig. 3e). Hence, CoN_3_C/rGO on the electrode would react with PMS to form a complex (CoN_3_C/rGO-PMS*) at first, and then ETP occurred between CoN_3_C/rGO-PMS* and BPA, achieving the oxidation of BPA (Fig. 3h). Moreover, the potential and current variations in the CoN_3_C/rGO/PMS system were larger than those observed in CoN_3_C/PMS, and negligible changes were detected in the rGO/PMS system, indicating enhanced electron transfer interaction existed due to the nano-island-like structure.

To quantify the steady concentration of ROS, nitrobenzene (NB), benzoic acid (BA), furfuryl alcohol (FFA), 1,4-benzoquinone (p-CBA), and methyl phenyl sulfoxide (PMSO) were used as chemical probes (Supplementary Figs. 43–45). According to the result, the contribution rate of ETP achieved around 94%, demonstrating its dominant pathway for BPA degradation in CoN_3_C/rGO/PMS. While for the CoN_3_C/PMS system, this contribution decreases to only about 11%, further confirming the significant regulation role of rGO sea.

On this basis, the relationship between different electronic parameters (electrophilic index, nucleophilicity index, softness, and hardness) of various pollutants and ln(*k*_*obs*_) reflects the correlation. For example, electrophilic index (R^2^ = 0.98) and nucleophilicity index (R^2^ = 0.91) of all pollutants show the obvious linear relationships with their ln(*k*_*obs*_) (Fig. 3f and Supplementary Table 10). While the hardness (R^2^ = 0.15) and softness (R^2^ = 0.19) reflect no correlations with ln(*k*_*obs*_) (Supplementary Fig. 46). These findings suggest that electron-donating organic pollutants exhibit a tendency to be degraded more easily, which is consistent with characteristics of ETP mechanism (Fig. 3g).

### Mechanism of island-sea effect in CoN_3_C/rGO/PMS

**Fig. 4:**
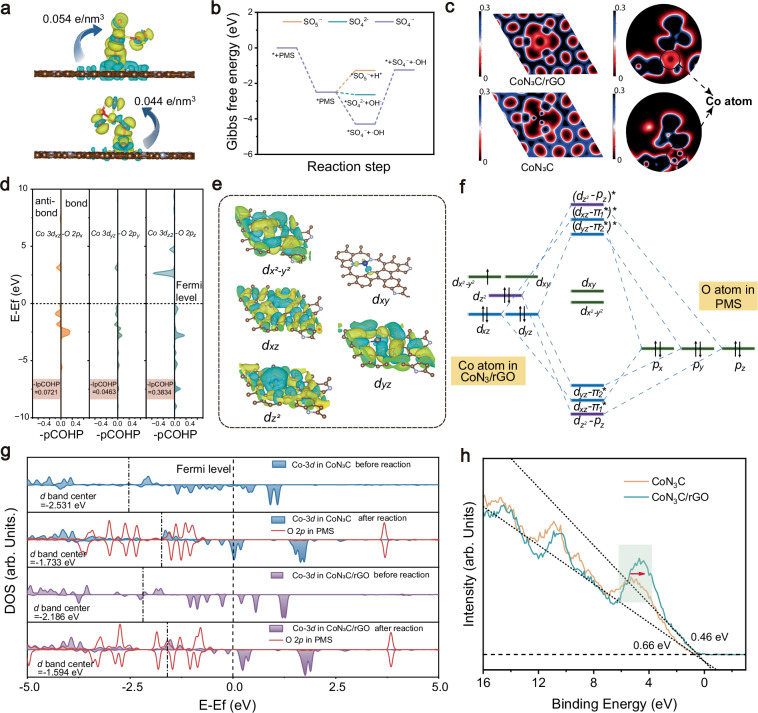
The enhanced mechanism of island-sea effect. **a** Differential charge density of PMS adsorbed on CoN_3_C/rGO and CoN_3_C. **b** Gibbs free energy of CoN_3_C/rGO/PMS reaction. **c** Two-dimensional electron localization functions of CoN_3_C/rGO/PMS and CoN_3_C/PMS. **d** Projected crystal orbital Hamilton populations (pCOHP) of Co *d*_*xz*_-O *p*_*x*_, Co *d*_*yz*_-O *p*_*y*_, and Co *d*_*z2*_-O *p*_*z*_. **e** Wave functions of *d* orbitals in CoN_3_C/rGO. **f** Schematic illustration of bonding and anti-bonding orbitals between Co atom in CoN_3_C/rGO and O atom in PMS. **g** Co 3*d* projected density of states (PDOS) of CoN_3_C/rGO and CoN_3_C, O 2*p* PDOS of adsorbed PMS. **h** Ultraviolet Photoelectron Spectroscopy results of CoN_3_C/rGO and CoN_3_C (The dot-dashed lines represent linear fits to the leading edges of the spectra used to determine the valence band maximum (VBM) by extrapolation to the baseline (horizontal dashed line). The details of dot-dashed lines are shown in Methods—“Determination of valence band maximum (VBM) from UPS spectra)”. Source data are provided as a Source data file.

DFT calculation was employed to unravel the electronic-level mechanism behind the island-sea effect. Although CoN_3_C exhibits a stronger adsorption energy toward PMS than CoN_3_C/rGO (−2.659 eV vs. −1.863 eV), the latter facilitates more efficient electron transfers (Fig. 4a and Supplementary Figs. 47–48). Combined with thermodynamic analysis provided by Gibbs free energy calculations that while radical generation is thermodynamically feasible for both systems, the dominant reaction pathway is different. For CoN_3_C, the diffuse orbital results in an overly strong adsorption, inducing excessive O–O bond stretching and breaking (radical pathway) (Fig. 4b). Conversely, CoN_3_C/rGO features an optimized, moderate adsorption energy, which preserves the PMS molecular integrity (Supplementary Fig. 49)^32,33^. The differential charge density further illustrates the charge redistribution upon PMS adsorption, with electron-donating regions localized around the Co atom and electron-accepting regions around the O atom of PMS (Supplementary Fig. 50). Additionally, for CoN_3_C-PMS*, only the first-shell N atoms participate in electron replenishment, whereas for CoN_3_C/rGO-PMS*, second-shell C atoms in island also contribute electrons, demonstrating rGO’s role as an electron sea that modifies the electron transport properties of CoN_3_C island.

The electronic modulation within the island-sea architecture is illustrated in ELF results (Fig. 4c and Supplementary Fig. 51). Upon the formation of catalyst-PMS* complex, the Co center in CoN_3_C/rGO exhibits a significantly increased degree of electron delocalization. While for CoN_3_C, electrons form highly localized regions, which lead to enhanced electron mobility for CoN_3_C/rGO. This transition from localization to delocalization suggests that the rGO “sea” does not merely act as a passive support, its extended π-conjugated network actively participates in charge redistribution, thereby lowering the energy barrier for electron transport from the Co “island” to the adsorbed PMS. The enhanced electron mobility is further demonstrated by the work function (Supplementary Fig. 52). The ESP of Co atom in CoN_3_C/rGO increases from −20.34 eV to −19.30 eV, whereas the values for CoN_3_C remain significantly more negative (−23.31 eV to −22.74 eV). That indicates sole CoN_3_C has a stronger binding force on electrons, hindering interface electron transfer.

To gain deeper insights into the orbital level interactions, crystal orbital Hamilton populations (COHP) and density of states (DOS) analyses were conducted. Results of integrals of projected COHP show that Co 3*d*- O 2*p* bonding in CoN_3_C/rGO-PMS* is significantly stronger than CoN_3_C (Supplementary Fig. 53). Specifically, the -IpCOHP of Co *d*_*z2*_- O *p*_*z*_ is higher than other orbitals, verifying its dominant channel for interfacial electron transfer (Fig. 4d and Supplementary Figs. 54–56). In contrast, CoN_3_C shows a more pronounced occupancy of antibonding states below the Fermi level (E_F_), which inherently reduces its bond order and stability. To visualize this difference, wave function plots reveals that the Co *d*_*z2*_ orbital in CoN_3_C/rGO exhibits a radially contracted and axially elongated shape, which maximizes the directional orbital overlap with the axial PMS ligand, suppressing random hybridization (Fig. 4e, f and Supplementary Figs. 57–59). While the CoN_3_C displays a diffuse and radially expanded electron cloud, limiting the effective interfacial coupling. This suggests that the island-sea architecture facilitates the formation of a more robust catalyst-PMS* complex favoring the ETP. From the DOS of Co 3*d*, the introduction of rGO leads to change of spin polarization, and spin-down electrons contribute more to reaction (Fig. 4g). Meanwhile, the rGO support induces a significant upshift of Co *d*-band center from −2.531 eV to −2.186 eV, optimizing the antibonding orbital filling states, thereby strengthening the adsorption energy of the key intermediate (PMS*). After adsorbing the PMS, the *d*-band center further up, which of CoN_3_C/rGO-PMS* still higher than CoN_3_C-PMS*, indicating the higher reactivity of CoN_3_C/rGO-PMS*. Furthermore, in CoN_3_C/rGO, the *d*_*z2*_ orbital of Co exhibits more concentrated and intense electronic states near the Fermi level, corresponding to wave function results (Supplementary Figs. 60–63). In comparison, the dispersed and weak electronic states were observed in the *d*_*z2*_ orbital of CoN_3_C, which can also explain its low activity and is prone to generating ROS. The above results were also confirmed by Ultraviolet Photoelectron Spectroscopy (UPS) (Fig. 4h). Compared with CoN_3_C, the occupied states near the E_F_ become narrower and more significant for CoN_3_C/rGO. And the upshift of the valence band maximum (VBM) (0.46 eV for CoN_3_C/rGO vs. 0.66 eV for CoN_3_C) towards the E_F_ suggests an elevation of the *d*-band center. In summary, the island-sea effect optimizes the orbital coupling strength and directionality, effectively lowering the activation energy barrier for the ETP pathway, which concurrently boosts the catalytic activity and reaction selectivity of CoN_3_C/rGO/PMS system through the regulation of *d*_*z2*_ orbital electrons.

Notably, the ETP mechanism demonstrates pollutant-dependent selectivity that scales with the electronic structure and energy gap between the HOMO of typical electron-donating (BPA, 4BP, Phe) or electron-withdrawing pollutants (PNP) and the LUMO of CoN_3_C/rGO/PMS, revealing an electronic structure-activity relationship for both electron-donating and electron-withdrawing compounds (detailed discussion is in Supplementary Figs. 64–69, Supplementary Tables 11–14 and Supplementary Data 1 and 2).

### Assessment of application potential

The nonradical dominant pathway of ETP in CoN_3_C/rGO/PMS system shows highly promising application potential. Common cations, anions, and natural humic acid matter did not affect the degradation efficiency of BPA in CoN_3_C/rGO/PMS obviously except H_2_PO_4_^−^ reduced the *k*_*obs*_, while Fe^3+^ increased it, which resulted from H_2_PO_4_^−^ complexing with Co^2+^ and catalytic role of Fe^3+^ (Supplementary Fig. 70)^34^. Tap water, river water, spring water, and groundwater were selected as background to explore the influence of practical water matrix, and over 95% of BPA could be removed under every kind of water (Supplementary Fig. 71). Therefore, CoN_3_C/rGO maintains high resistance against common aqueous interfering substances. The detoxification ability for BPA of CoN_3_C/rGO system was evaluated by the detection of degradation intermediates. During the UPLC-QTOF-MS and Fukui index of BPA, the possible degradation pathways could be obtained (Supplementary Figs. 72 and 73). The results from the toxicity estimation software tool (T.E.S.T.) based QSAR show that the acute toxicity, developmental toxicity, and bioaccumulation factor of almost all final products are significantly lower than BPA (Supplementary Fig. 74).

**Fig. 5:**
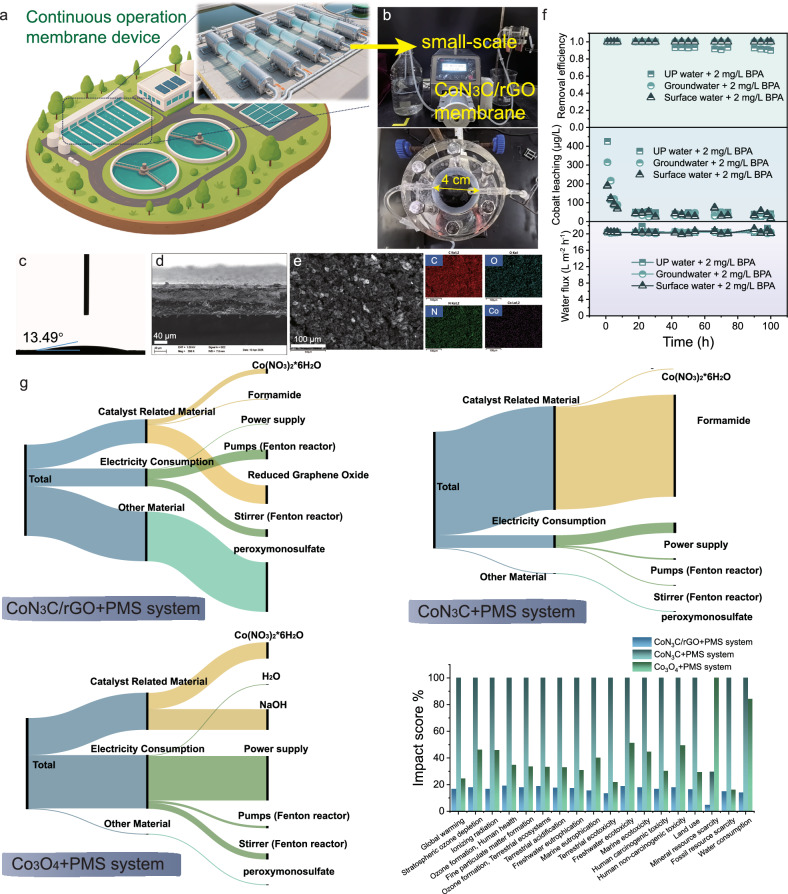
The application potential of CoN_3_C/rGO. **a** Concepts of CoN_3_C/rGO membrane continuous flow system. **b** Small scale continuous flow unit of CoN_3_C/rGO/PVDF membrane. **c** Contact angle of CoN_3_C/rGO/PVDF membrane. **d** SEM cross-section and **e** EDS mappings of CoN_3_C/rGO/PVDF membrane (The micrograph is representative of observations made from at least five different locations across prepared samples). **f** BPA removal performance in CoN_3_C/rGO/PVDF system under different water matrices. **g** Impact scores of various descriptors in LCA for different Co-based catalytic systems. Source data are provided as a Source data file.

Considering the problem that powder catalysts are difficult to recycle and reuse, CoN_3_C/rGO was coated on PVDF membrane to construct a continuous flow reactor (Fig. 5a, b). The water contact angle test shows a 13.49° at 30 ms, the characteristic of typical hydrophilic, demonstrating that CoN_3_C/rGO/PVDF membrane would be beneficial for treating water-soluble contaminants (Fig. 5c). The SEM images of cross-section and surface suggest that CoN_3_C/rGO is tightly attached to the PVDF membrane and has a pore structure, which is conducive to the sufficient contact between PMS and pollutants (Fig. 5d and Supplementary Fig. 75). EDS mapping exhibits the uniform distribution of Co, N, C, and O elements (Fig. 5e and Supplementary Fig. 76). Ultrapure water, groundwater, and surface water were used as typical water matrices to be treated by a continuous flow reactor at a flow rate of 1.0 mL/min (Fig. 5f). The results show that BPA in three water matrices could be removed > 90% during the entire 100-hour operation period with stable water flux. Moreover, Co leaching concentration remained at an eco-friendly level (<1 mg/L) in the whole process. A proof-of-concept dual-chamber device successfully achieved ~40% BPA removal about 36 h via long-distance electron transfer, fundamentally eliminating the risk of SO_4_^2−^ secondary pollution in the treated water (Supplementary Fig. 77). Although the macroscopic degradation kinetics are inherently limited by the mass transfer resistance and ohmic drop of the decoupled setup, this result robustly validates the practical environmental feasibility of the ETP mechanism. These findings indicate the application potential of CoN_3_C/rGO/PMS system in water treatment.

A comprehensive assessment of the energy and environmental impacts of the CoN_3_C/rGO/PMS system was conducted using the life cycle assessment (LCA) methodology, with comparative analyses performed against both the CoN_3_C/PMS system and the conventional Co_3_O_4_/PMS system (Fig. 5g, Supplementary Tables 14 and 15 and Supplementary Data 3–5). Based on the data obtained from the input materials and energy required for each system to treat one ton of BPA wastewater, it is evident that the CoN_3_C/rGO/PMS system demonstrates superior performance across all assessed indicators of environmental impact and resource utilization when compared to both the CoN_3_C/PMS system and the Co_3_O_4_/PMS system. That is due to when synthesizing the same amount of catalyst, CoN_3_C/rGO uses less raw materials, and the amount of catalyst and oxidant required for treating BPA wastewater is also lower. Furthermore, it is noteworthy that the use of PMS contributes the most to the environmental impact in the CoN_3_C/rGO/PMS system, which means future efforts should prioritize the reduction of oxidant usage.

## Discussion

In this work, we designed and demonstrated an nano-island-like catalyst CoN_3_C/rGO can achieve the high selectivity of the ETP path in Fenton-like reaction through island-sea synergistic effect. The CoN_3_C/rGO/PMS system can remove the target contaminant BPA completely within 5 min, and the normalized *k*_*obs*_ can reach 25 min^−1^·g^−1^·L, which is superior to CoN_3_C and most of the reported catalysts. Additionally, this catalyst exhibits 94% selectivity to generate the ETP path, which can obtain robust degradation efficiency for various electron-rich contaminants. Mechanistic analysis revealed that in the structure of CoN_3_C/rGO, as the “island”, carbon nitride provides the unsaturated coordination environment, asymmetric electron distribution, and the stabilizing effect of Co atom. As the “sea”, rGO utilized its delocalized π electron network to regulate the electronic structure and orbital of active sites. Specifically, this nano-island-like structure elevates the d-band center while sharpening the electron occupation states of the *d*_*z2*_ orbital—the primary reactive channel for PMS activation, thereby suppressing antibonding generation and stochastic hybridization processes. Hence, this island-sea effects synergistically enhance both activity and selectivity. Moreover, CoN_3_C/rGO shows the stability in a wide pH range and actual water matrix. The continuous flow test exhibited CoN_3_C/rGO/PVDF membrane system has potential for application in treating pollutants under various water matrices. This study highlights the directional regulation of electron and orbital through nano-island-like structure, achieving the high selectivity generation of the ETP path in AOPs.

## Methods

### Chemicals

Bisphenol A (BPA, ≥99%), benzoic acid (BA, ≥99.5%), furfuryl alcohol (FFA, ≥98%), norfloxacin (NOR, ≥98%), ofloxacin (OFX, ≥98%), 4-Bromophenol (4-BP) were purchased from Aladdin (Shanghai, China). Sodium hydrogen carbonate (NaHCO_3_, ≥99.5%), potassium iodide (KI, ≥99.5%), sodium thiosulfate (Na_2_S_2_O_3_, ≥99%), sodium chloride (NaCl, ≥99.5%), sodium nitrate (NaNO_3_, ≥99%), sodium fluoride (NaF, ≥98%), sodium dihydrogen phosphate (NaH_2_PO_4_, ≥99%), sodium sulfate (Na_2_SO_4_, ≥99%), anhydrous ethanol (EtOH, ≥99.7%), methanol (MeOH, ≥99.5%), potassium thiocyanate (KSCN, ≥99%), cobaltous nitrate hexahydrate (Co(NO_3_)_2_•6H_2_O, ≥98.5%), phenol (Phe, ≥99%), calcium sulfate (CaSO_4_, ≥99%), magnesium sulfate (MgSO_4_, ≥98%), sulfuric acid (H_2_SO_4_, 95–98%), ferric chloride (FeCl_3_, ≥99%), p-Nitrophenol (PNP, 99%) and sodium hydroxide (NaOH, ≥96%) were provided by Sinopharm Chemical Reagent Co., Ltd (Shanghai, China). Peroxymonosulfate (PMS, 42–46%), methylene blue (MB, ≥98%), methyl phenyl sulfone (PMSO_2_, 98%), tert-butyl alcohol (TBA, 97%), dimethyl sulfoxide (DMSO, ≥99%), 1,4-benzoquinone (p-BQ, 97%), L-Histidine (L-his, 99.5%), and nitrobenzene (NB, 99%) were obtained from Macklin (Shanghai, China). Methyl phenyl sulfoxide (PMSO, 98.68%) was provided by Bide Pharmatech Co., Ltd (Shanghai, China). Acetonitrile (MeCN, ≥99.9%) was obtained by Tedia (USA). 4-Chlorobenzoicacid (p-CBA, 99%) was obtained by Meryer (Shanghai, China). All chemicals were analytical grade or higher and were used without further purification. The whole experiment used ionized water as solvent.

### Synthesis of CoN_3_C/rGO and CoN_3_C

CoN_3_C/rGO was synthesized by one-step formamide hydrothermal method. Specifically, 0.1 g of rGO powder was ultrasonically dispersed in 30 mL of formamide firstly. Then 0.04 g Co(NO_3_)_2_•6H_2_O was added to the mixture and dispersed by ultrasound for 30 min. The resulting mixture was moved into a Teflon autoclave and heated for 12 h in 180 °C. Finally, the products were cleaned with ultra-pure water, collected, and freeze-dried. The synthesis of CoN_3_C is similar to CoN_3_C/rGO, except that rGO was not added.

### Characterization methods

The morphologies of catalysts were examined by SEM (ZEISS Sigma 360) and TEM (JEOL JEM-2100F). The HAADF-STEM images were recorded on a Thermofisher Spectra 300 aberration-corrected transmission electron microscope. The XRD (Rigaku SmartLab SE) equipped with a Cu Kα was used to analyze the phase structure. The XPS (Thermo Scientific K-Alpha) was used to investigate the chemical state information of surface elements. UPS results were obtained by XPS (AXIS Supra, Shimadzu). Raman spectra were recorded in a Horiba LabRAM HR Evolution Raman Spectrometer at 532 nm, together with FT-IR acquired on a Thermo Fisher Scientific Nicolet iS20 Fourier Transform Infrared Spectrometer to explore chemical bonding information. The Co load on the catalyst and the leaching concentration in the degradation process was measured by AAS (GGX-920, Haiguang). The signals of ROS were trapped by electron paramagnetic resonance EPR (ESR5000, Bruker), using DMPO and 2, 2, 6, 6-tetramethyl-4-piperidinol (TEMP) as trapping agents. The Co K-edge XAFS spectra were collected at beamline BL13SSW of the Shanghai Synchrotron Radiation Laboratory, and the data were analyzed by Athena and Artemis software.

### Measurement of catalytic performance

The batch degradation experiments were conducted in a 200 mL beaker at room temperature and stirred at 500 rpm. Firstly, 2.5 mg catalyst was added to 50 mL BPA solution (10 mg·L^−1^, pH = 6.2 without adjusting and buffer). Then 0.5 mM PMS was added to the above solution, which marked the start of degradation reaction. The reaction solution was sampled at regular intervals. Then the samples were filtered through membrane filters of 0.22 µm and mixed with 50 μL Na_2_S_2_O_3_ (150 mM) solution. The concentration of BPA was measured by high-performance liquid chromatography (HPLC, LC-20, Shimadzu) equipped with a C18 column (4.6 mm × 150 mm, 5 μm) and UV-vis detector. To explore the pH adaptability of CoN_3_C/rGO, the pH of the solutions was adjusted by using H_2_SO_4_ (0.01 M) and NaOH (0.01 M). In the cycling experiment, the catalyst after each reaction was filtered and extracted for the next experiment. In the degradation experiments for various contaminants, the concentration of PNP, OFX, Phe, 4-BP was measured by HPLC, while the concentration of MB was recorded in UV-vis spectrophotometer (UV-2600i, Shimadzu). The operator conditions were followed by Supplementary Table 1. All the above experiments were performed three times to ensure reproducibility.

The removal efficiency was calculated by Eq. 1. The degradation kinetics were fitted by a pseudo-first-order model, which was followed by Eq. 2. The turnover frequency (TOF) of catalysts was calculated by Eqs. 3–4:

1 $${\rm{Removal}} {\rm{efficiency}}=\frac{{C}_{0}-{C}_{t}}{{C}_{0}}$$

2 $$-{\mathrm{ln}}\left(\frac{{C}_{t}}{{C}_{0}}\right)={{\rm{k}}}_{\mathrm{obs}}\mathrm{t}$$

3 $${\mathrm{TOF}}=\frac{{\rm{C}}_{0}\times ({\rm{BPA}}\, {\rm{removal}}\, {\rm{efficiency}}\%)}{{C}_{{Co}}\times {\rm{t}}}$$

4 $${{C}}_{{Co}}=\frac{{{C}}_{\mathrm{catalyst}} \times ({\rm{Co}}\,{\rm{content}}\%)}{59}$$

Where the *C*_*t*_ (mg·L^−1^) is the contaminant’s concentration at the sampled time, the *C*_0_ (mg·L^−1^) is the initial concentration of the contaminant. The *t* is the time to reach degradation equilibrium, and *k*_*obs*_ (min^−1^) is the reaction rate constant. The *C*_*catalyst*_ is the catalyst dosage in the experiment. The Co content is the Co loading content of catalyst analyzed by AAS.

The contribution of ·OH and the direct electron transfer pathway were obtained by the following equations:

5 $${R}_{{\cdot }\mathrm{OH}}=\frac{{\rm{k}}_{\cdot \mathrm{OH},\mathrm{BPA}}{[\cdot \mathrm{OH}]}_{\mathrm{ss}}}{{k}_{\mathrm{obs},BPA}}$$

6 $${R}_{{ETP}}=1-{R}_{\cdot OH}$$

The ${{\rm{k}}}_{\cdot \mathrm{OH},\mathrm{BPA}}$ is the second-order reaction rate constants between ·OH and BPA (6.9 × 10^9^ M^−1^ s^−1^). The [·OH]_ss_ is the steady state concentration of ·OH.

The intermediates in the BPA degradation process were analyzed by UPLC equipped with quadrupole-time of flight mass spectrometry (UPLC-QTOF-MS, impactHD, Bruker). Additional analysis methods are provided in Supplementary Method.

### Analytical methods for PMS concentration

The concentration of PMS residual in solution was measured by the following method: 0.1 mL filtered sample and 2.9 mL chromogenic agent (10 mM KI and 5 mM NaHCO_3_) were mixed for 5 min. Then the mixture was analyzed by UV-vis spectrophotometer at 352 nm.

### Electrochemical measurements

Open circuit potential (OCP), current time curve (i-t), and linear sweep voltammetry (LSV) were conducted in Na_2_SO_4_ solution (50 mM) and tested on the electrochemical workstation (660D, Chenhua). The Ag/AgCl and Pt wire electrode were used as the reference electrode and counter electrode, while the glassy carbon electrode was the working electrode. The preparation of the working electrode follows the following steps: 20 mg of catalysts, 40 µL Nafion solution (5%) and 60 µL of EtOH were mixed with ultrasonic dispersion for 30 min. 10 µL mixed solution was placed on a glassy carbon electrode and dried at room temperature. The two cell electrochemical experiment was carried out in a dual-chamber electrolytic cell (50 mL for each chamber). Two carbon paper electrode coated with 2 mg catalysts are, respectively, used as the cathode and the anode.

### Quantitative method for ROS

Competitive dynamics experiments were used to quantify ROS in CoN_3_/rGO/PMS system. The steady-state concentrations of ·OH, SO_4_^·−^, ^1^O_2_, O_2_^·−^, and Co(IV) = O ([·OH]_ss_, [SO_4_^·−^]_ss_, [O_2_^·−^]_ss_, [^1^O_2_]_ss_, [Co(IV) = O]_ss_) were calculated by using probe compound NB, BA, FFA, p-CBA, and PMSO. The initial concentration of the probe compound was set to 10 mg/L, except the PMSO was 100 µM. Finally, the [·OH]_ss_, [SO_4_^·−^]_ss_, [O_2_^·−^]_ss_, [O_2_^·−^]_ss_, and [Co(IV) = O]_ss_ can be obtained by the following equations:

7 $$\mathrm{ln}\frac{[\mathrm{NB}]}{{[\mathrm{NB}]}_{0}}=-{k}_{NB,\cdot \mathrm{OH}}{[\cdot \mathrm{OH}]}_{\mathrm{ss}}t=-{{k}_{obs,NB}}^{t}$$

8 $${\mathrm{ln}}\frac{[\mathrm{BA}]}{{[\mathrm{BA}]}_{0}}=-\left({\mathrm{k}}_{\mathrm{BA},\cdot {\mathrm{OH}}}{[\cdot {\mathrm{OH}}]}_{\mathrm{ss}}+{{\rm{k}}}_{{\mathrm{BA}},{\mathrm{SO}}_{4}^{\cdot -}}{[{\mathrm{SO}}_{4}^{\cdot -}]}_{\mathrm{ss}}\right)t=-{k}_{{obs},{BA}}t$$

9 $$\mathrm{ln}\frac{[\mathrm{FFA}]}{{[\mathrm{FFA}]}_{0}} 	=-\left({k}_{\mathrm{FFA},\mathrm{\cdot OH}}{\left[\mathrm{\cdot OH}\right]}_{\mathrm{ss}}+{{\rm{k}}}_{\mathrm{FFA},{\mathrm{SO}}_{4}^{\cdot -}}{[{\mathrm{SO}}_{4}^{\cdot -}]}_{\mathrm{ss}}+{k}_{\mathrm{FFA},{}^{1}O_{2}}{[{}^{1}O_{2}]}_{\mathrm{ss}}\right)t \\ 	=-{k}_{{obs},{FFA}}t$$

10 $$\mathrm{ln}\frac{[p-\mathrm{CBA}]}{{[p-\mathrm{CBA}]}_{0}}=	-\left({k}_{p-\mathrm{CBA},\cdot \mathrm{OH}}\left[ \cdot \mathrm{OH}\right]+{\rm{k}}_{p-{\mathrm{CBA}},{\mathrm{SO}}_{4}^{\cdot -}}[{\mathrm{SO}}_{4}^{\cdot -}]\right. \\ 	\left.+{k}_{p-{\mathrm{CBA}}{,}^{1}O_{2}}{[{\scriptstyle{1}\atop}O_{2}]}_{\mathrm{ss}}+{\rm{k}}_{p-\mathrm{CBA},{\rm{O}}_{2}^{\cdot -}}[{\rm{O}}_{2}^{\cdot -}]\right)t =-{k}_{{obs},p-{CBA}}t$$

11 $$\eta \mathrm{ln}\frac{{[\mathrm{PMSO}]}_{0}}{{[\mathrm{PMSO}]}_{0}-\frac{1}{\eta }[{\mathrm{PMSO}}_{2}]}={k}_{\mathrm{PMSO},\mathrm{Co}(\mathrm{IV})=O}[{\mathrm{PMSO}}_{\mathrm{ss}}]t={k}_{{obs},{{PMSO}}_{2}}t$$

The pseudo first-order rate constants of NB (*k*_*obs, NB*_), BA (*k*_*obs, BA*_), FFA (*k*_*obs, FFA*_), and p-CBA (*k*_*obs, p-CBA*_) could be obtained from the plots of −ln([NB]_t_/[NB]_0_), −ln([BA]_t_/[BA]_0_), −ln([FFA]_t_/[FFA]_0_), and −ln([p-CBA]_t_/[p-CBA]_0_) versus time *t*, respectively. The pseudo-first-order reaction rate constants (*k*_*obs, PMSO2*_) can be obtained from plots of $\eta {\mathrm{ln}}\frac{[{\mathrm{PMSO}}]_{0}}{[{\mathrm{PMSO}}]_{0}-\frac{1}{\eta }[{\mathrm{PMSO}}_{2}]}$ versus time *t*. The second-order reaction rate constants between the probe compounds and reactive species are shown in Supplementary Table 3. η is the conversion rate of PMSO, which means moles of PMSO_2_ generated per mole of PMSO oxidized.

### Determination of valence band maximum (VBM) from UPS spectra

The valence band maximum (VBM) of the samples was determined from the UPS valence band spectra using the standard linear extrapolation method. For each sample, a linear function was fitted to the intensity rise region using the least-squares method in the binding energy range where the signal increases linearly from the background. The functional form used for the fitting is shown as follows:

12 $${\rm{y}}={\rm{kE}}+{\rm{b}}$$

where y is the photoemission intensity, E is the binding energy, k is the slope, and b is the intercept.

### Fabrications of CoN_3_C/rGO/PVDF membrane

Add an appropriate amount of CoN_3_C/rGO powder to ultrapure water, sonicate for 1 h to prepare a dispersion solution with a concentration of 0.2 mg/mL. Then, filter the dispersion solution uniformly through a vacuum onto a PVDF membrane. After natural drying, the membrane is obtained.

### LCA analysis details

Here, we used Simapro 9.5 and data Ecoinvtent 3.9.1 for modeling, and quantified the model through ReCiPe 2016 Midpoint (H) V1.10/World (2010) H. The life cycle environmental effects of treating 1 ton of wastewater by three systems (CoN_3_C/rGO/PMS system, CoN_3_C/PMS system, and Co_3_O_4_/PMS system) were obtained. Through Simapro Monte Carlo simulation (1000 times), the uncertainty analysis results were obtained, including the mean value, median value, confidence interval, and other statistical parameters.

### DFT calculation methods

Here, we employed first-principles calculations based on density functional theory (DFT) using the Vienna Ab initio Simulation Package (VASP) to investigate the charge distribution and Gibbs energy of the samples^35^. Atomic coordinates of the optimized configurations of all samples are shown in Supplementary Data 6–9. The exchange and correlation interactions between electrons were described using the Perdew-Burke-Ernzerhof (PBE) functional within the Generalized Gradient Approximation (GGA)^36^. A plane-wave cutoff energy of 500 eV was set to ensure accurate and convergent results. The convergence criteria for total energy and force components were set to 1 × 10⁻⁵ eV and 0.05 eV/Å, respectively. A Monkhorst-Pack k-point grid with a density of 1 × 1 × 1 was used to sample the reciprocal space for geometry optimization. Considering the magnetic atoms, the spin polarization approach was employed in the present work, and the initial moment was randomly set. Due to the strong on-site Coulomb repulsion between the d electrons of Co atoms, the DFT + U method was used. COHP of Co–O bonds was calculated by lobster-5.1.0^37^. The Fukui functions calculations were performed using the Gaussian 16 package. The geometry of the investigated structures was optimized and frequency analyses using the B3LYP level in conjunction with the 6–31 G(d) basis set.

## Supplementary information

Supplementary information

Description of Additional Supplementary Files

Supplementary Data 1

Supplementary Data 2

Supplementary Data 3

Supplementary Data 4

Supplementary Data 5

Supplementary Data 6

Supplementary Data 7

Supplementary Data 8

Supplementary Data 9

Transparent Peer Review file

## Source data

Source data

## Data Availability

The data that support the findings of this study are openly available in Figshare at 10.6084/m9.figshare.32011896^38^. Source data are provided with this paper.
