# Supplementary material for: Breaking the activity-selectivity trade-off in Fenton-like catalysis by d-orbital modulation of single-atom sites within a nano-island-like structure
Source: Nat Commun. 2026 Jun 8;17:7293. doi: 10.1038/s41467-026-74072-2 (PMC13402592; doi:10.1038/s41467-026-74072-2)
Supplement: Supplementary file 1 — Supplementary information [file 41467_2026_74072_MOESM1_ESM.pdf]

Supplementary Information

Breaking the activity-selectivity trade-off in Fenton-like catalysis by d-orbital modulation of single-atom sites within a nano-island-like structure

Yuxin Chen [1], Xing Xu [1], Jianrong Zeng [2, 3], Yang Yu [1], Yingshuai Ma [1], Peilin Zhang [1], Tao Zeng [4], Haiguang Zhang [1], Liang Tang [5] *, Runzeng Liu [1] *, Youcai Zhu [1] *

[1] Shandong Key Laboratory of Environmental Processes and Health, School of Environmental Science and Engineering, Shandong University, Qingdao 266237, PR China.

[2] Shanghai Synchrotron Radiation Facility, Shanghai Advanced Research Institute, Chinese Academy of Sciences, Shanghai 201204, PR China.

[3] Shanghai Institute of Applied Physics, Chinese Academy of Sciences, Shanghai 201800, PR China.

[4] Zhejiang Key Laboratory of Environment and Health of New Pollutants, School of Environment, Hangzhou Institute for Advanced Study, University of Chinese Academy of Sciences, Hangzhou, 310024, P.R. China.

[5] Key Laboratory of Organic Compound Pollution Control Engineering (MOE), School of Environmental and Chemical Engineering, Shanghai University, Shanghai 200444, China

*Corresponding authors, E-mail: tang1liang@shu.edu.cn (Liang Tang); rz.liu@sdu.edu.cn (Runzeng Liu); zhuucai@sdu.edu.cn (Youcai Zhu)

Supplementary Figures

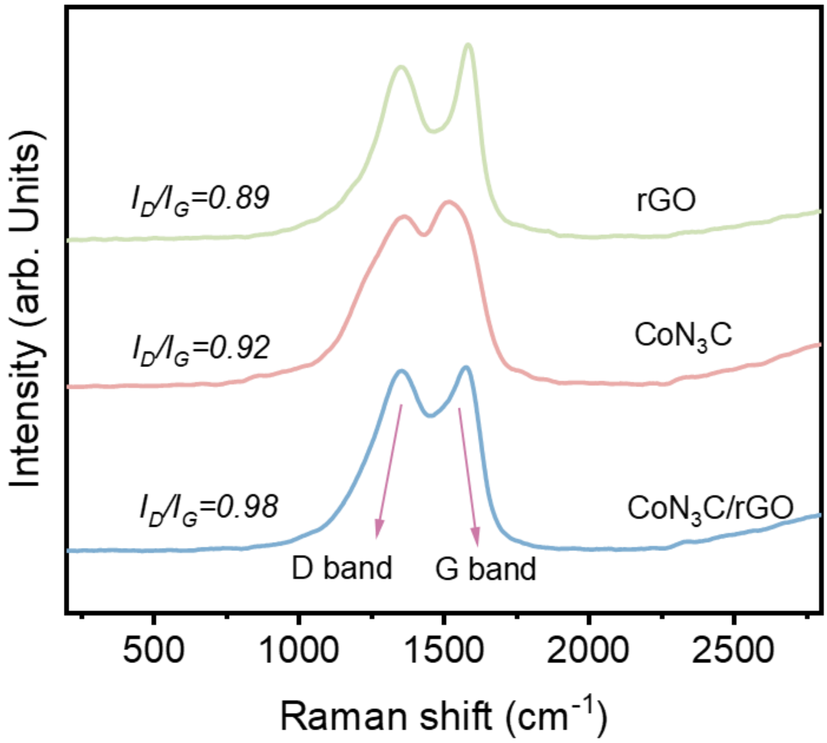

Supplementary Fig. 1. Raman characterization of catalysts. Raman spectra of CoN_3C/rGO, CoN_3C, rGO.

According to Raman spectra, the charateristic D band (disorder induced phonon mode) and G band (graphite band) of CoN_3C/rGO, CoN_3C, and rGO are obtained at 1352 cm^{-1} and 1580 cm^{-1}. The defect level of materials represented by the peak intensity ratio (I_D/I_G) indicate that more defects were generated in CoN_3C/rGO (0.98 vs 0.89 and 0.92), which can support more anchoring sites, and regulate the electronic structure of active site, enhancing the catalytic activity[1-3].

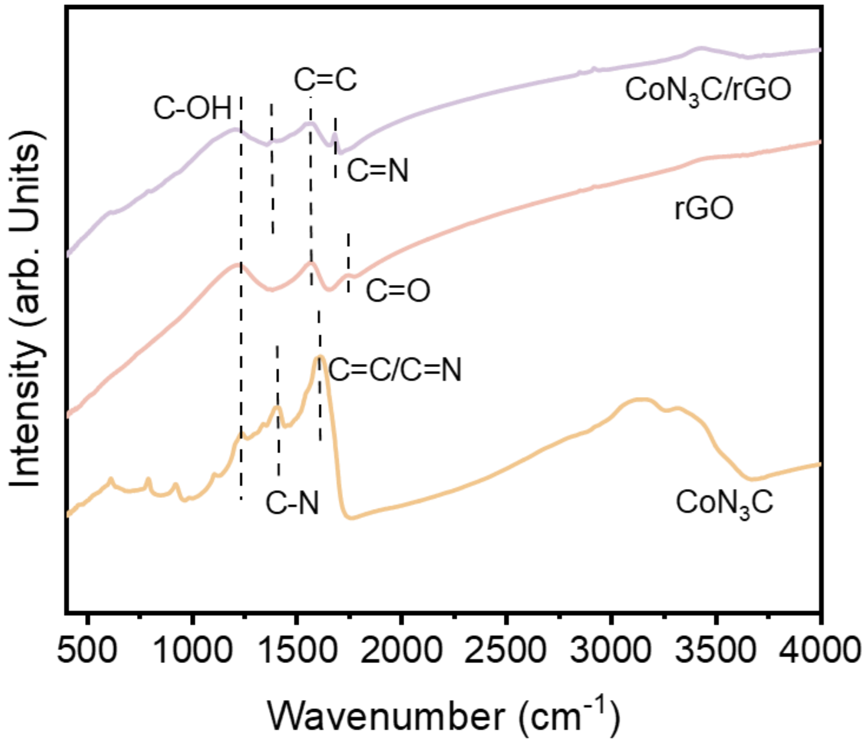

Supplementary Fig. 2. FT-IR characterization of catalysts. FT-IR of CoN_3C/rGO, CoN_3C, rGO.

For the FTIR spectra, the peak of C-OH stretching vibrations (1220 cm^{-1}) is present in three materials[4]. The characteristics of C-N (1370-1420 cm^{-1}) and C=N (1610-1680cm^{-1}) are observed in CoN_3C/rGO and CoN_3, confirming the CoN_3C island maintain their frameworks[5-9]. While a peak at 1750 cm^{-1} corresponding to C=O of rGO disappeared after compositing with CoN_3C to form CoN_3C/rGO, indicating that rGO was further reduced under hydrothermal conditions, which restore its graphitic conjugated π-electron network[10].

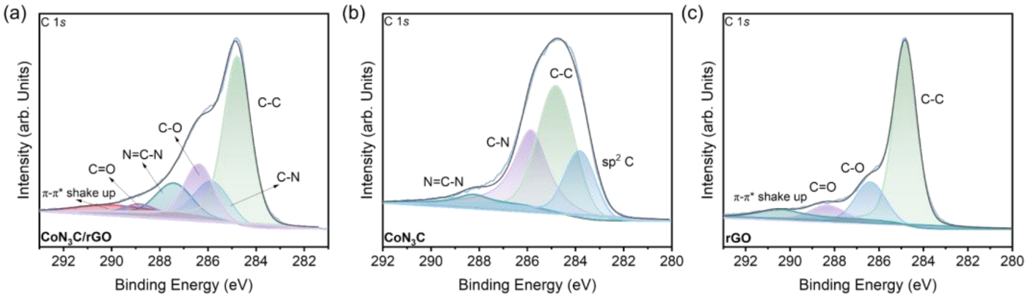

Supplementary Fig. 3. XPS characterization of catalysts. High-resolution XPS spectra of C 1*s* for (a) CoN_3C/rGO, (b) CoN_3C, (c) rGO.

The high resolution C 1*s* spectra provide detailed insights into the structural evolution of the carbon matrix. For the CoN_3C, two peaks at 283.8 eV and 284.8 eV are assigned to the graphitized sp^2 C=C framework and sp^3 C-C species, respectively[11]. The N-C=N peak, representing the triazine/heptazine heterocyclic skeleton, is identified at 288.3 eV[12]. In contrast, the N-C=N peak in CoN_3C/rGO appears at 287.4 eV[13]. This significant negative shift indicates an increased electron density on the carbon atoms in the vicinity of the Co-N_3 centers.

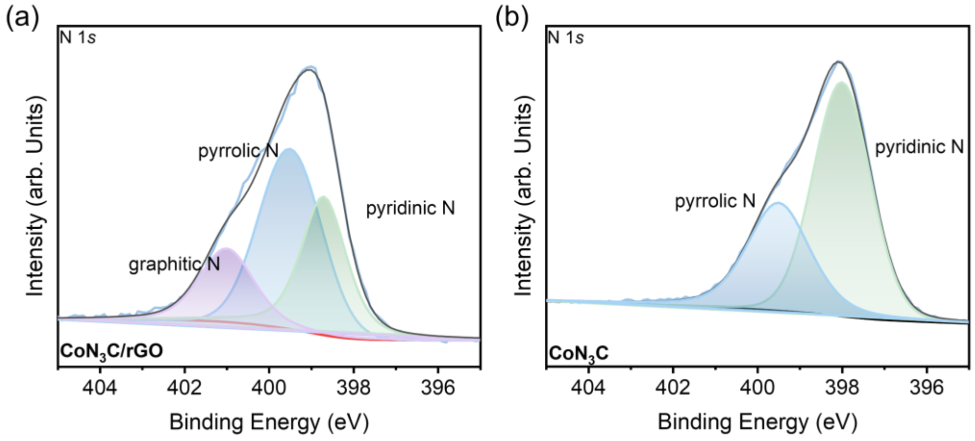

Supplementary Fig. 4. XPS characterization of catalysts. High-resolution XPS spectra of N 1*s* for (a) CoN_3C/rGO, (b) CoN_3C.

The N 1*s* spectra further corroborate the specific coordination environment. The spectrum of CoN_3C/rGO can be deconvoluted into three distinct peaks at 398.7 eV, 399.5 eV, and 401.0 eV, corresponding to pyridinic N, Co-N/pyrrolic N, and Graphitic N, respectively[14-17]. More importantly, higer ratio of pyrrolic N than pyridinic N exists in CoN_3C/rGO,while the opposite data is exhibited for CoN_3C. This suggests that the strong interfacial interaction with rGO induces the transformation of some rigid six-membered pyridinic rings into more flexible, electron-rich five-membered pyrrolic defects.

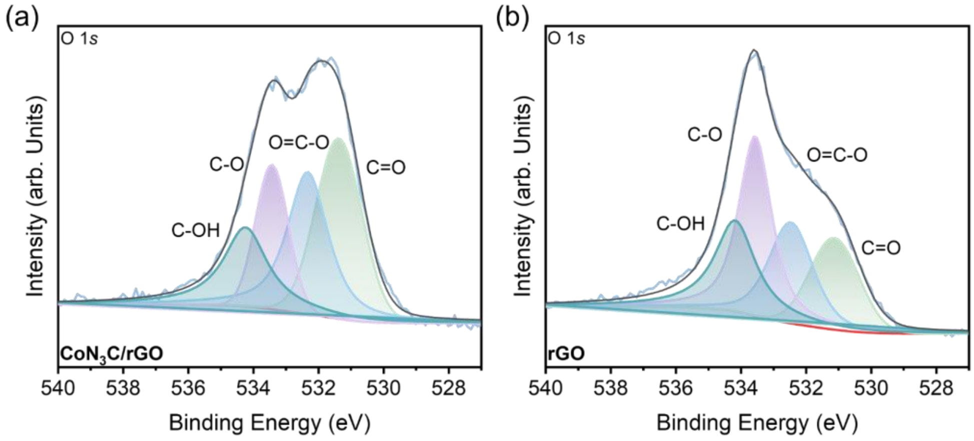

Supplementary Fig. 5. XPS characterization of catalysts. High-resolution XPS spectra of O 1*s* for (a) CoN_3C/rGO, (b) rGO.

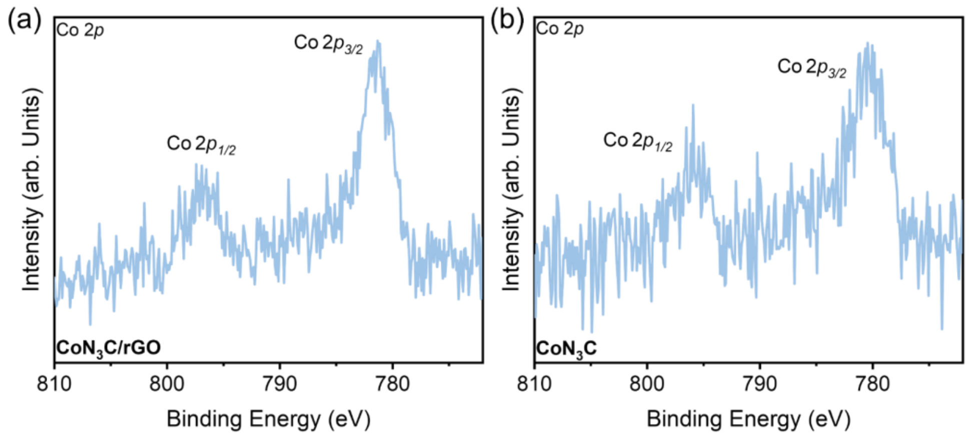

Supplementary Fig. 6. XPS characterization of catalysts. High-resolution XPS spectra of Co 2*p* for (a) CoN_3C/rGO, (b) CoN_3C.

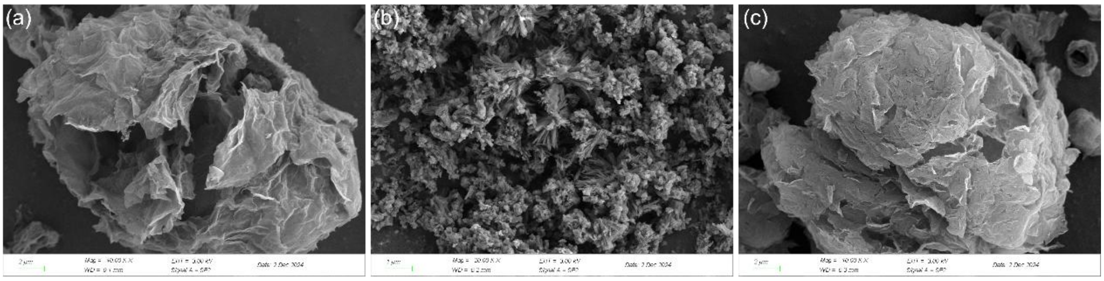

Supplementary Fig. 7. SEM characterization of catalysts. (a) SEM images of CoN_3C/rGO. (b) SEM images of CoN_3C. (c) SEM images of rGO.

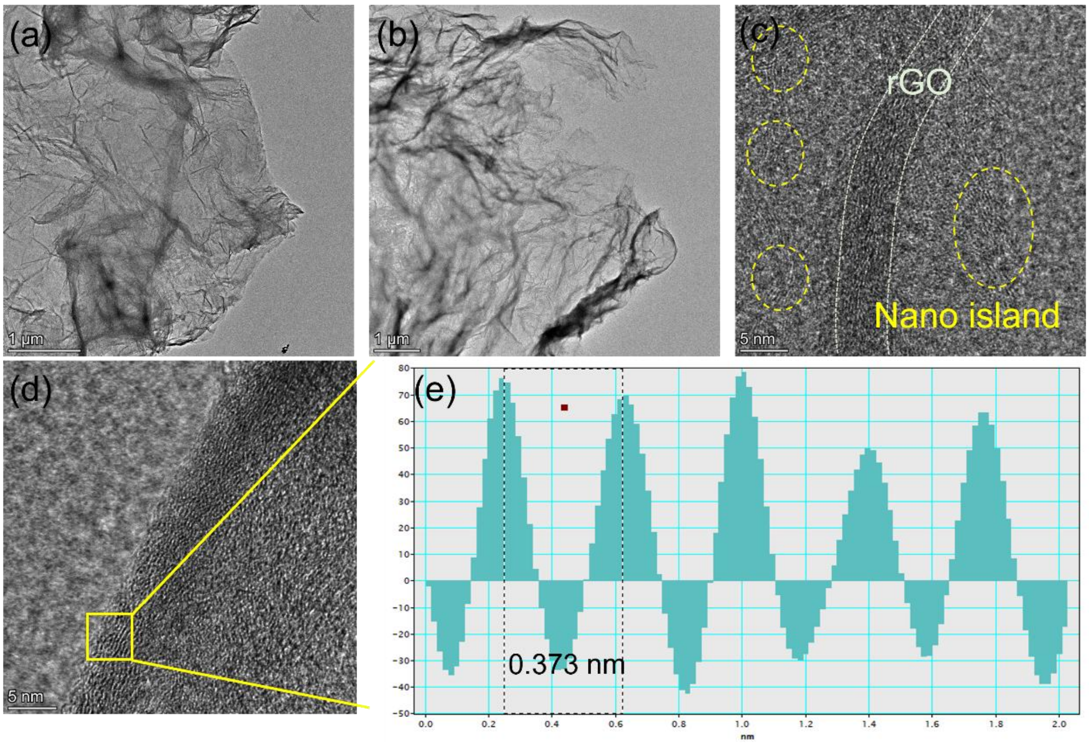

Supplementary Fig. 8. TEM characterization of CoN_3C/rGO and rGO. (a) TEM images of CoN_3C/rGO. (b) TEM images of rGO. (c) HRTEM images of CoN_3C/rGO. (d) HRTEM images of rGO (The area within the yellow box is where the inverse Fourier transform is performed to calculate the interlayer spacing). (e) Interlayer spacing of rGO.

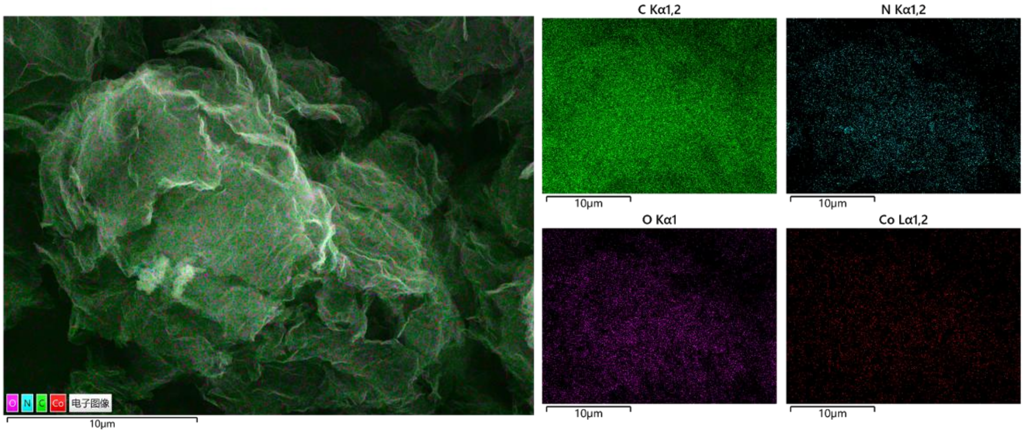

Supplementary Fig. 9. EDS mapping characterization of CoN_3C/rGO. SEM-EDS mapping of CoN_3C/rGO.

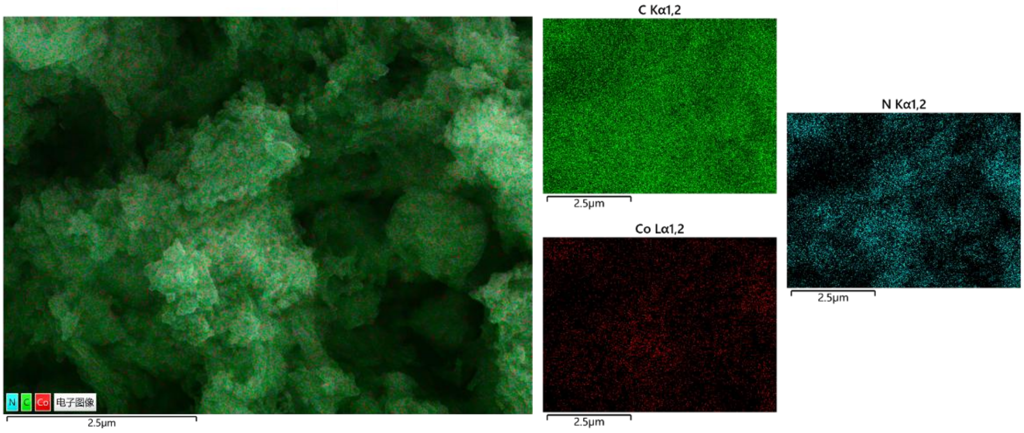

Supplementary Fig. 10. EDS mapping characterization of CoN_3C. SEM-EDS mapping of CoN_3C.

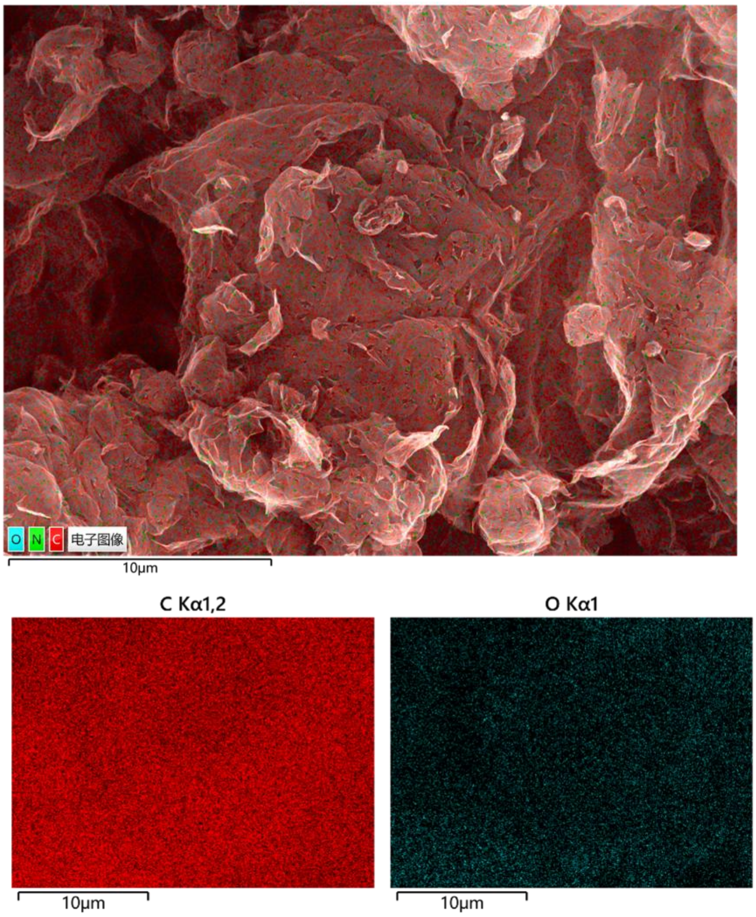

Supplementary Fig. 11. EDS mapping characterization of rGO. SEM-EDS mapping of rGO.

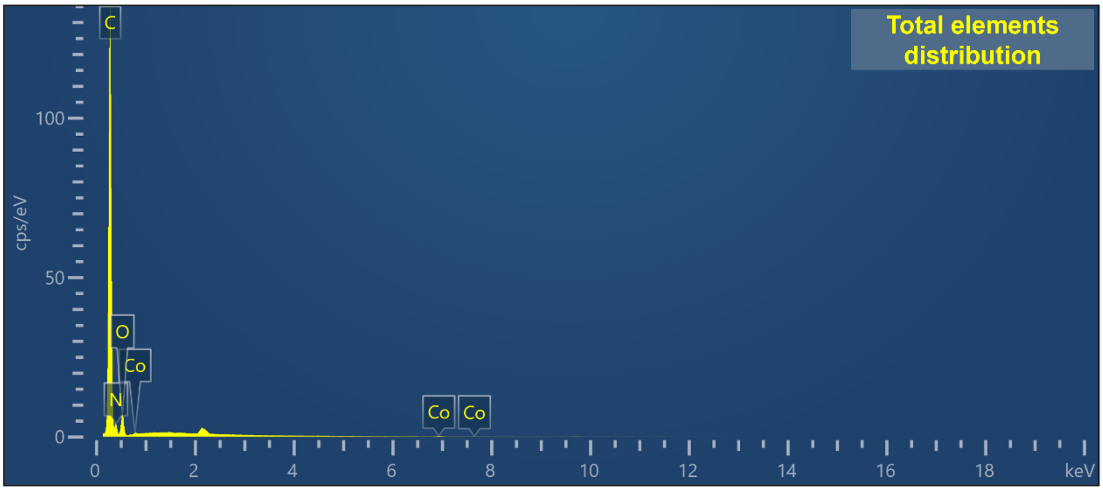

Supplementary Fig. 12. Elements contents in CoN_3C/rGO from EDS mapping. The signals of total elements distribution results about CoN_3C/rGO.

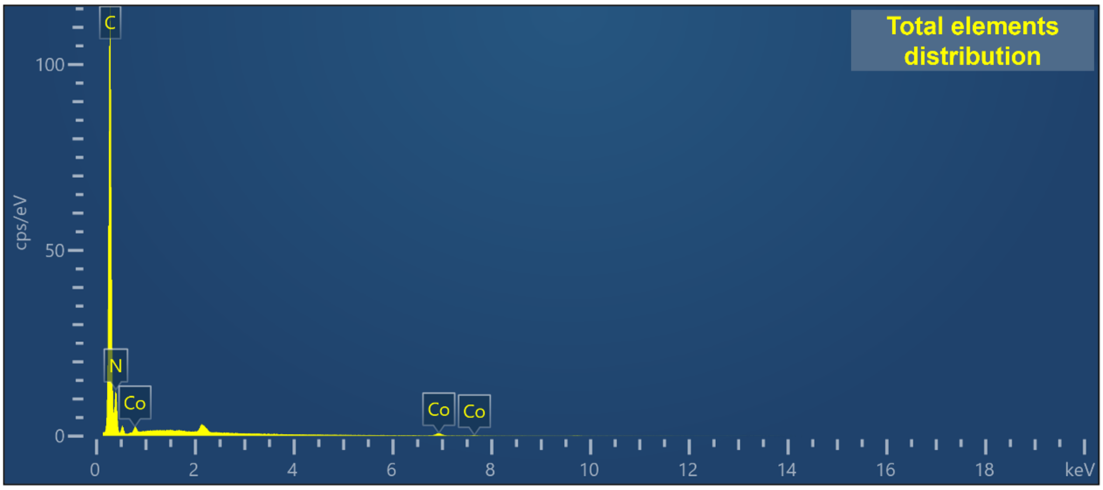

Supplementary Fig. 13. Elements contents in CoN_3C from EDS mapping. The signals of total elements distribution results about CoN_3C.

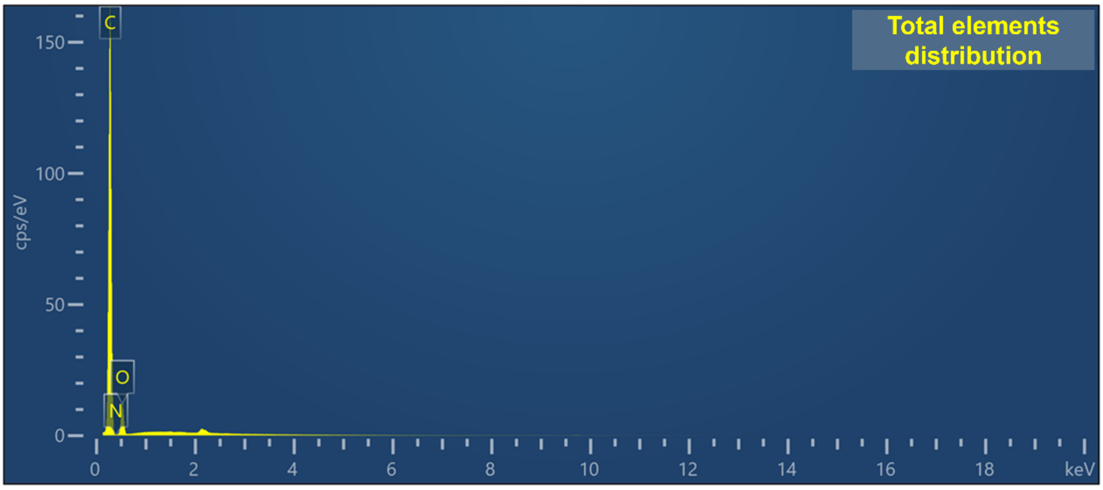

Supplementary Fig. 14. Elements contents in rGO from EDS mapping. The signals of total elements distribution results about rGO.

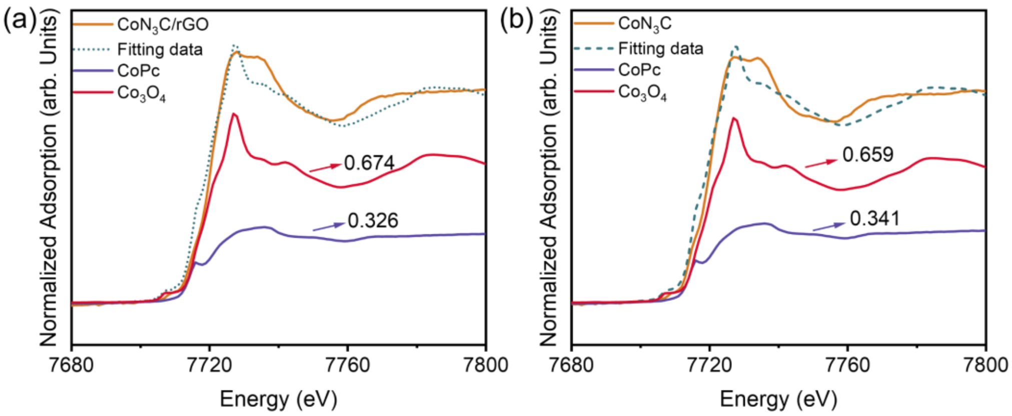

Supplementary Fig. 15. XANES LCF results. Linear combination fitting curves of (a) Co K-edge of CoN_3C/rGO. (b) Co K-edge of CoN_3C.

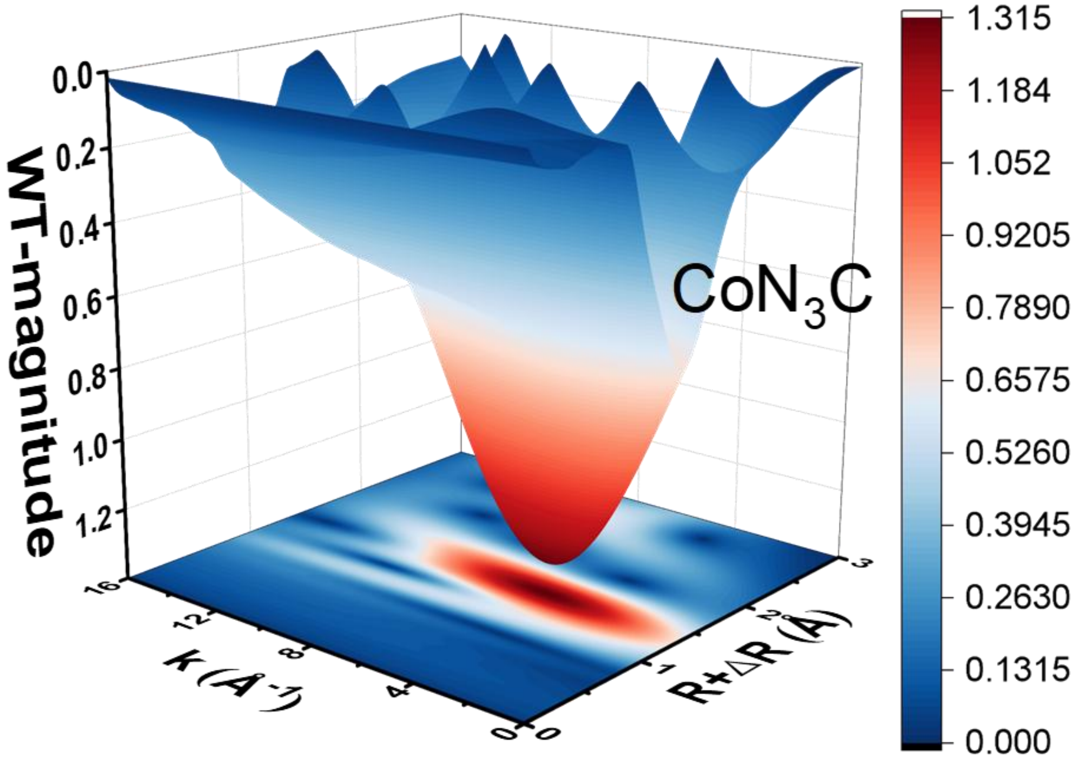

Supplementary Fig. 16. WT-EXAFS of catalysts. WT-EXAFS result of CoN_3C.

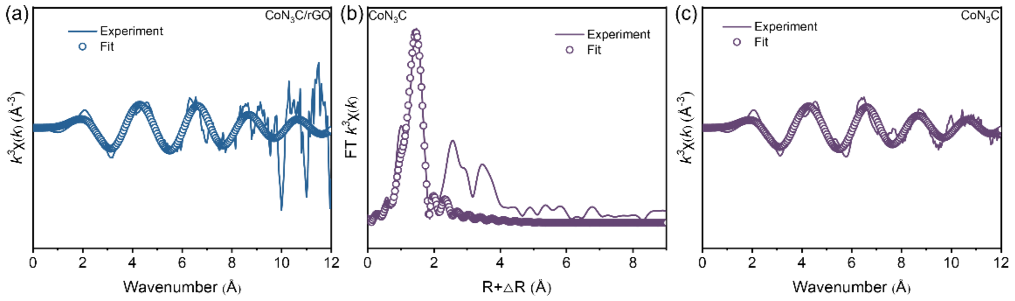

Supplementary Fig. 17. EXAFS characterization of catalysts. (a) EXAFS fitting analysis of CoN_3C/rGO in K space. EXAFS fitting analysis of CoN_3C in (b) R space and (c) K space.

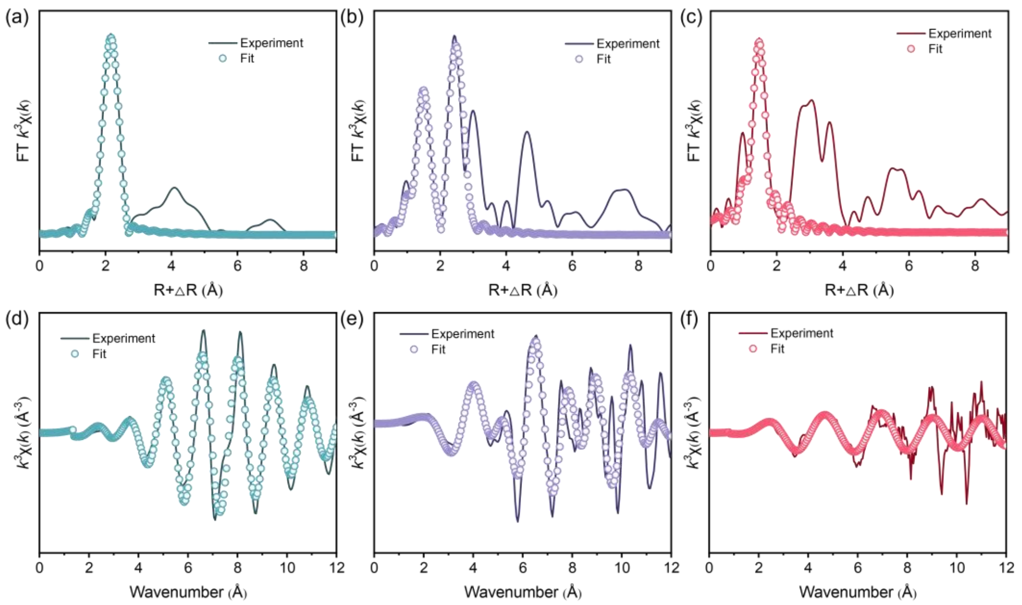

Supplementary Fig. 18. EXAFS characterization of reference samples. EXAFS fitting analysis of Co foil in (a) R space and (d) K space, Co_3O_4 in (b) R space and (e) K space, CoPc in (c) R space and (f) K space.

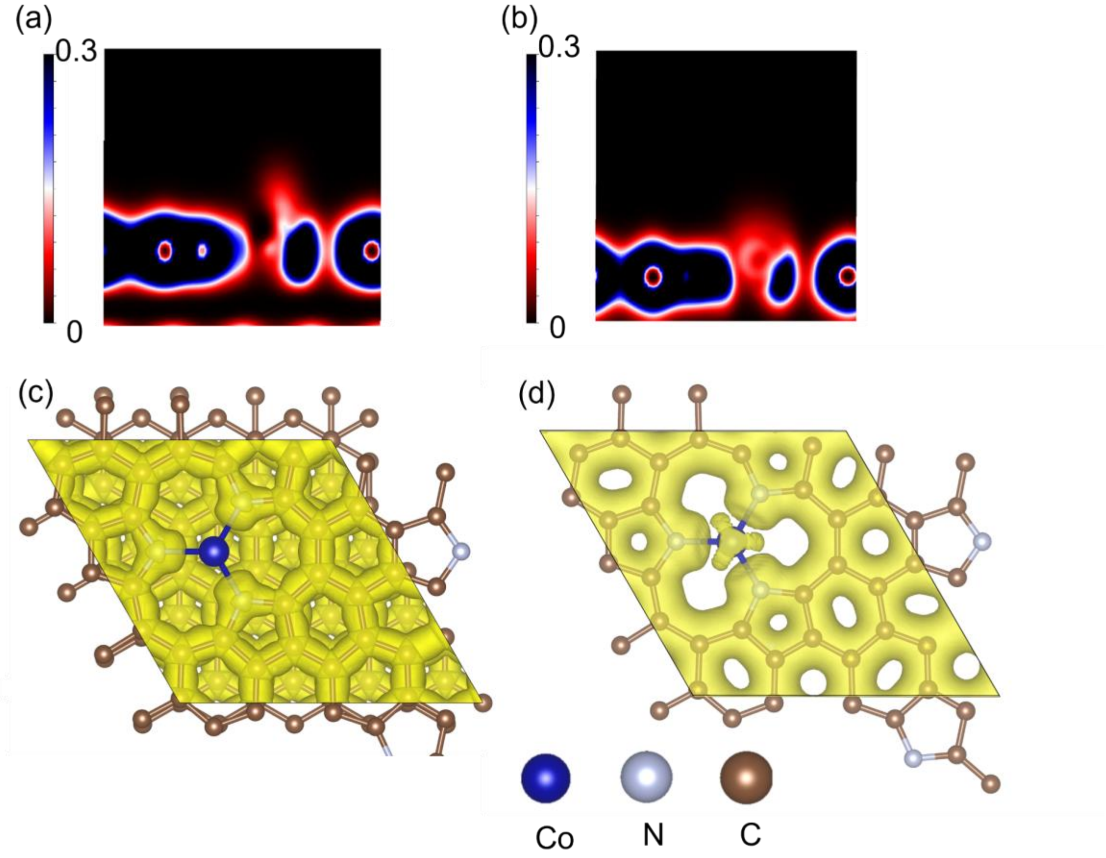

Supplementary Fig. 19. ELF results of catalysts. 2D cross section ELF images of (a) CoN_3C/rGO, (b) CoN_3C. 3D ELF images of (c) CoN_3C/rGO, (d) CoN_3C.

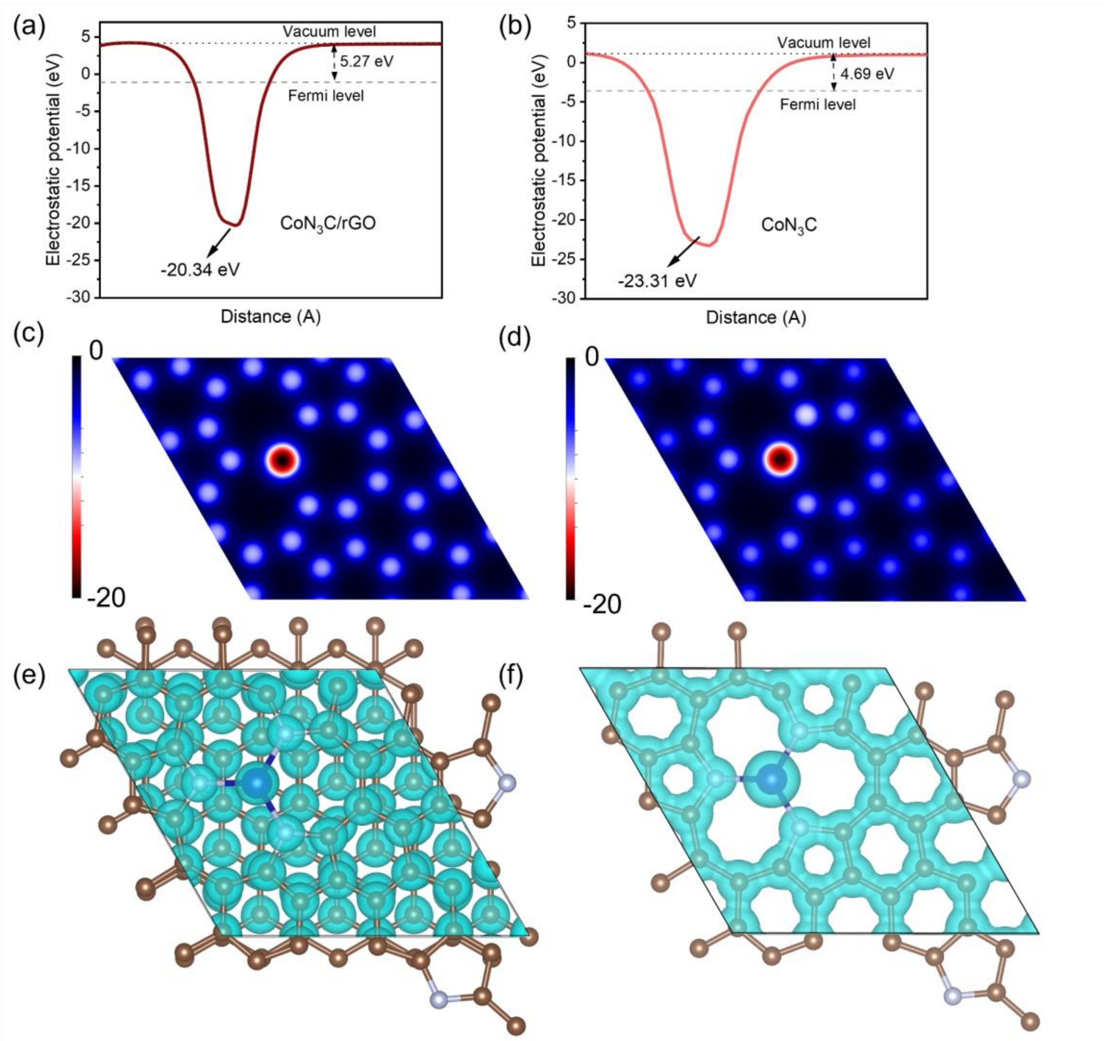

Supplementary Fig. 20. Work function and electrostatic potential results of catalysts. Work functions of (a) CoN_3C/rGO, (b) CoN_3C. 2D electrostatic potential images of (c) CoN_3C/rGO, (d) CoN_3C. 3D electrostatic potential images of (e) CoN_3C/rGO, (f) CoN_3C (The green area represents the equipotential surface where the electrostatic potential is -20 eV).

From the result of the electrostatic potential, the equipotential surface area where the electrostatic potential is -20 eV around the Co atom of CoN_3C/rGO is smaller than CoN_3C, indicating a lower electron density of Co in CoN_3C/rGO.

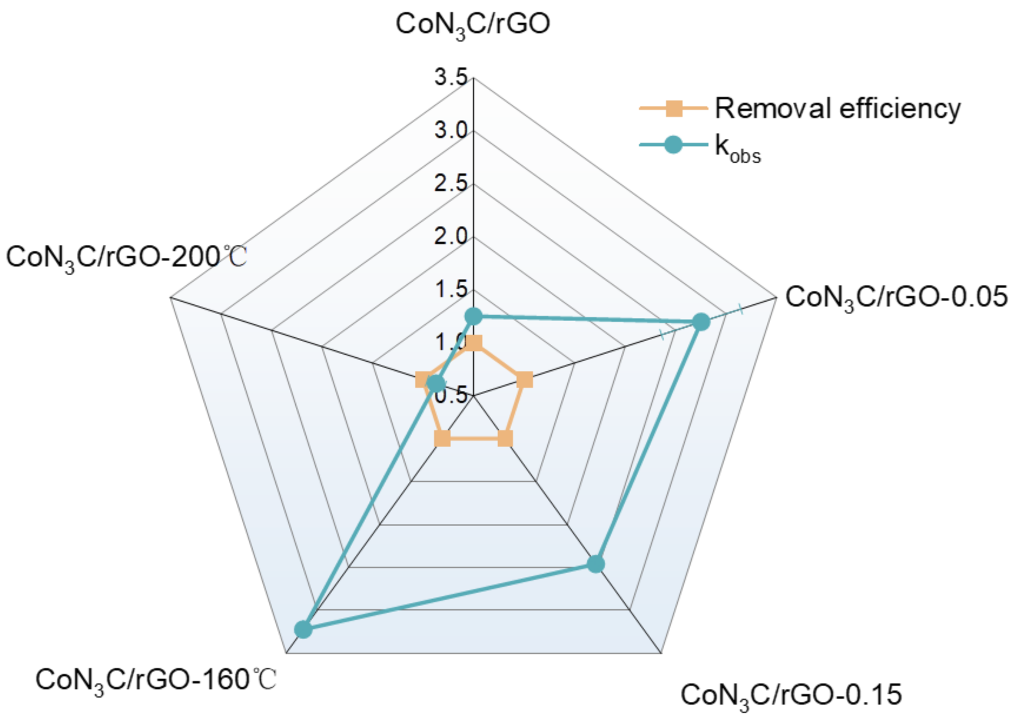

Supplementary Fig. 21. The catalytic performance of catalysts with different synthesis condition. The BPA degradation efficiency and k_{obs} with catalysts with different synthesis condition mediated PMS systems. Experiment conditions: [catalyst]= 0.05 g/L, [PMS]= 0.5 mM, [BPA]= 10 mg/L, pH= 6.0, room temperature if not otherwise specified. The error bars are standard deviation of three replicate tests (n= 3).

To construct the optimal island-sea architecture for efficient electron transfer, we systematically investigated the influence of synthesis parameters, specifically the hydrothermal temperature (160 °C, 200 °C) and rGO loading (0.05 g, 0.15 g), on the catalyst structure and PMS activation performance (Supplementary Figs. 21-22). Although all synthesized composites exhibited comparable initial degradation efficiencies, the sample prepared at 180 °C with 0.1 g rGO was identified as the optimal candidate, striking the best balance between cyclic stability, atom economy, and catalytic activity. It is worth noting that due to the amorphous nature of the CoN_3C phase and the flexible structure of rGO, no drastic morphological differences were

observed across these optimization ranges from TEM results (Supplementary Fig. 23). And the uniform distribution of C, N, O, Co can be obtained by EDS-mapping (Supplementary Fig. 24). This indicates that our island-sea assembly strategy is robust and capable of forming uniform heterostructures under a relatively wide range of synthesis conditions. Crucially, XPS characterization uncovers the electronic origin of this superiority: the optimal sample possesses the highest content of electron-donating pyrrolic N species, which not only serves as a robust anchor to prevent metal leaching (explaining the superior stability) but also induces an electron-rich state in Co centers (evidenced by the lowest Co 2*p* binding energy), thereby optimizing the electronic configuration of Co active sites (Supplementary Fig. 25).

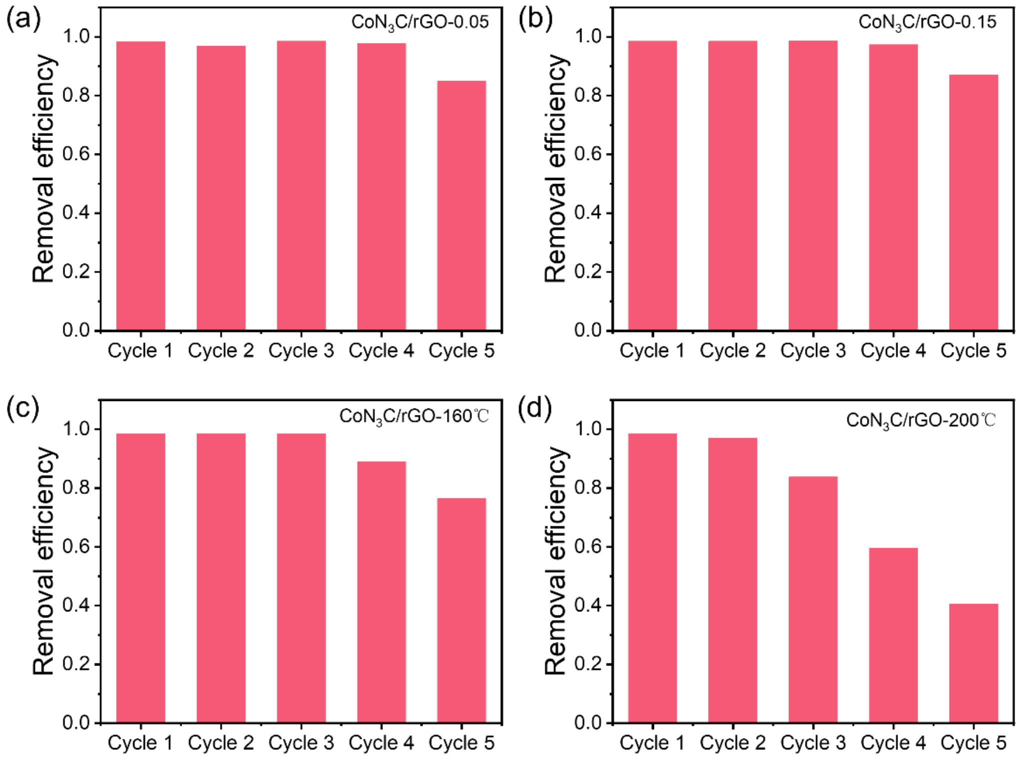

Supplementary Fig. 22. The stability of catalysts with different synthesis conditions. The cyclic performance for BPA removal of (a) CoN_3C/rGO-0.05g, (b) CoN_3C/rGO-0.15g, (c) CoN_3C/rGO-160 °C, (d) CoN_3C/rGO-200 °C.

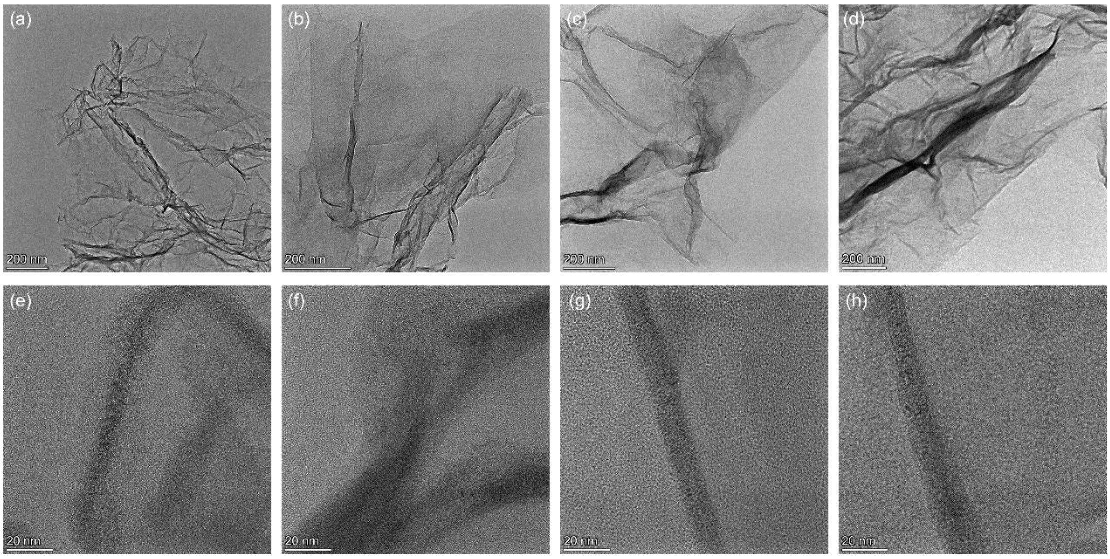

Supplementary Fig. 23. The morphologies of catalysts with different synthesis conditions. The TEM images of (a) CoN_3C/rGO-0.05g, (b) CoN_3C/rGO-0.15g, (c) CoN_3C/rGO-160 ℃, (d) CoN_3C/rGO-200 ℃; the HRTEM images of (e) CoN_3C/rGO-0.05g, (f) CoN_3C/rGO-0.15g, (g) CoN_3C/rGO-160 ℃, (h) CoN_3C/rGO-200 ℃.

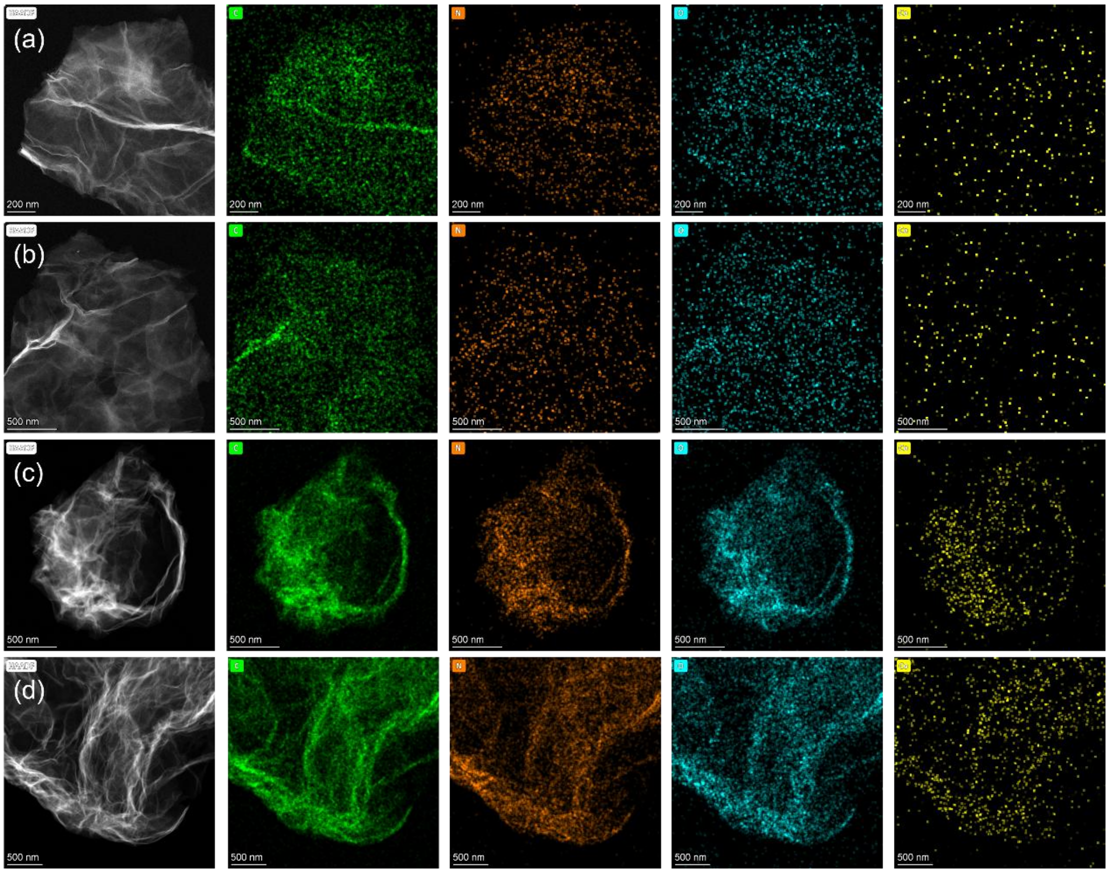

Supplementary Fig. 24. The elements distribution of catalysts with different synthesis conditions. The EDS mapping images of (a) CoN_3C/rGO-0.05g, (b) CoN_3C/rGO-0.15g, (c) CoN_3C/rGO-160 °C, (d) CoN_3C/rGO-200 °C.

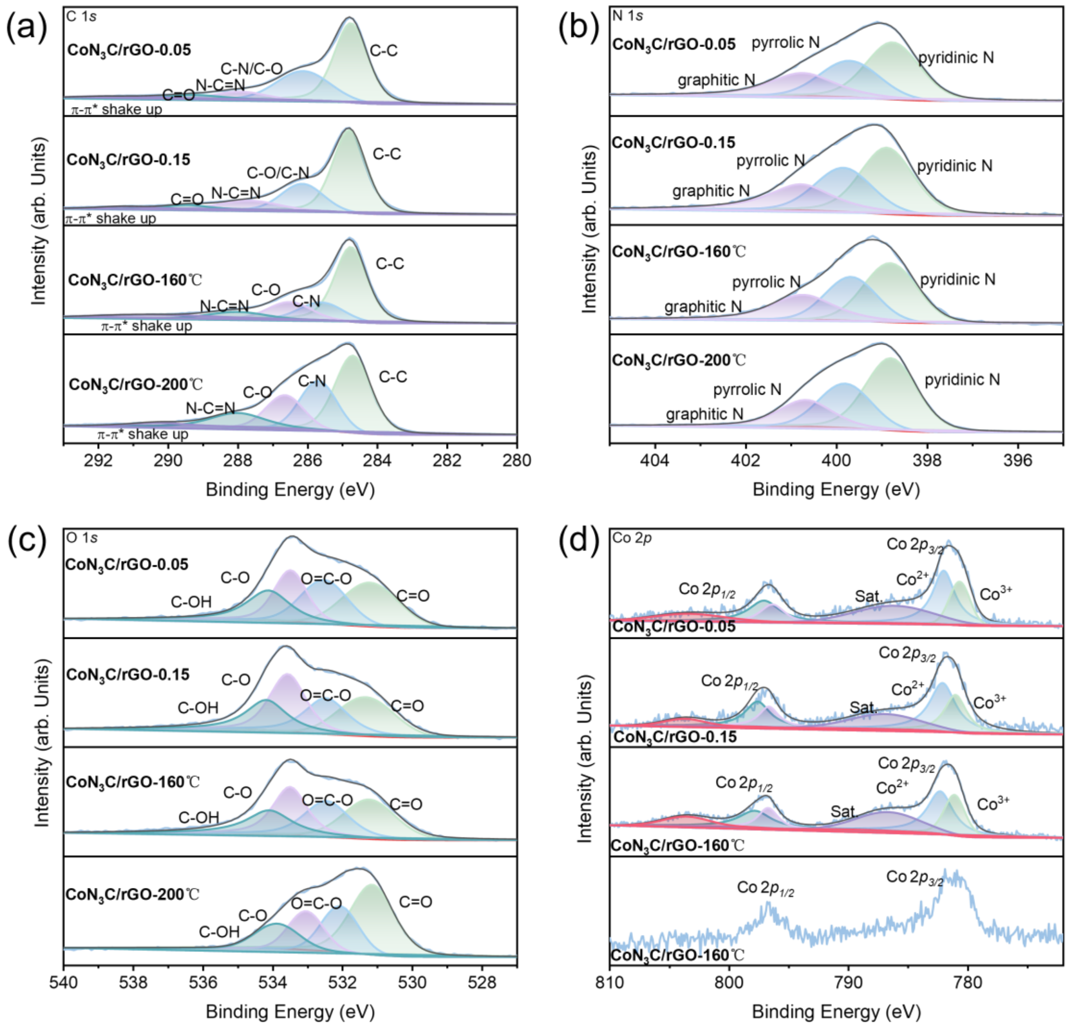

Supplementary Fig. 25. The chemical state of elements on catalysts with different synthesis conditions. The high-resolution XPS (a) C 1*s*, (b) N 1*s*, (c) O 1*s*, (d) Co 2*p* spectra of CoN_3C/rGO-0.05 g, CoN_3C/rGO-0.15 g, CoN_3C/rGO-160 ℃, and CoN_3C/rGO-200 ℃.

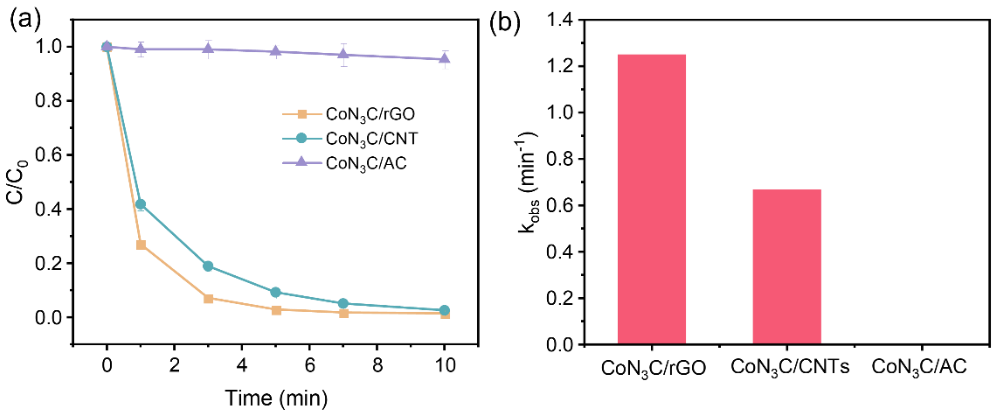

Supplementary Fig. 26. Effect of different sea carrieres. (a) The degradation curves, (b) k_{obs} of BPA in CoN_3C/rGO/PMS, CoN_3C/AC/PMS and CoN_3C/CNTs/PMS. Experiment conditions: [catalyst]= 0.050 g/L, [PMS]= 0.5 mM, [BPA]= 10 mg/L, pH=6.0, room temperature if not otherwise specified. The error bars are standard deviation of three replicate tests (n= 3).

To verify the universality of the island-sea strategy, CoN_3C islands were also anchored on activated carbon (AC) and carbon nanotubes (CNTs). As illustrated in Supplementary Fig. 26, the catalytic activity follows the order of CoN_3C/rGO > CoN_3C/CNTs > CoN_3C/AC. Compared with CoN_3C, XPS analysis reveals that CoN_3C/rGO possesses the minimal shift of Co 2*p* binding energy, indicating its highest electron density. Notably, the characteristic N-C=N skeleton signal was not resolved in the C 1*s* spectra of CoN_3C/AC and CoN_3C/CNTs. This is likely due to the insufficient electronic coupling between these supports and the CoN_3C islands, suggesting the interfacial electronic coupling is likely too weak to induce such a substantial shift, causing the N-C=N signal to remain overlapped with or masked by the C-N/C-O peaks (~285.8 and 286.3 eV)[18]. Consequently, the Co centers in CoN_3C/rGO maintain a more optimized, electron-rich environment, which is highly conducive to the fastest kinetics PMS activation. This preliminary exploration suggests that the structural and electronic properties of the "sea" carrier are pivotal to the performance.

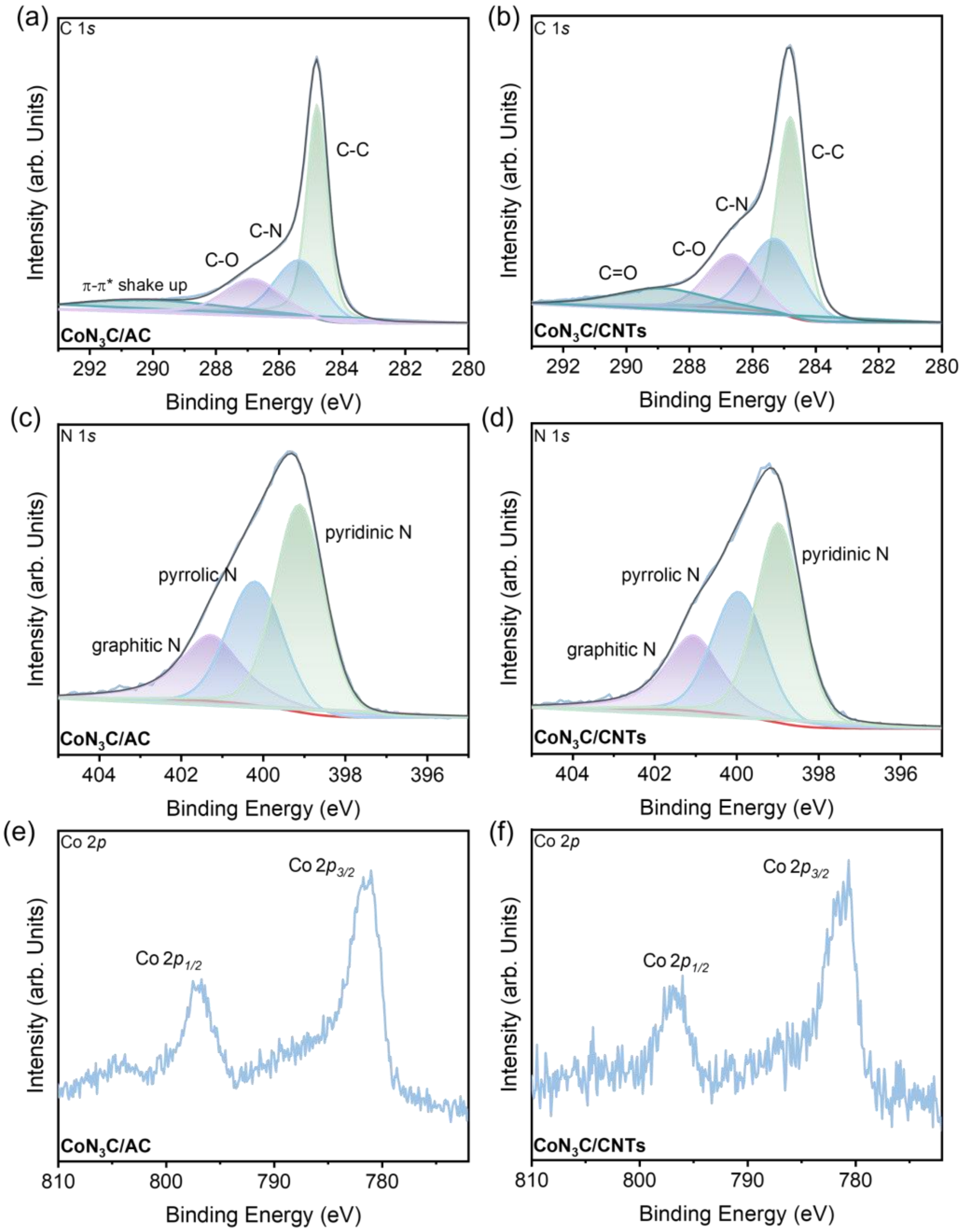

Supplementary Fig. 27. XPS characterization of catalysts with different sea carrieres. High-resolution XPS spectra of C 1*s* for (a) CoN_3C/AC, (b) CoN_3C/CNTs; N1*s* for (c) CoN_3C/AC, (d) CoN_3C/CNTs; Co 2*p* for (e) CoN_3C/AC, (f) CoN_3C/CNTs.

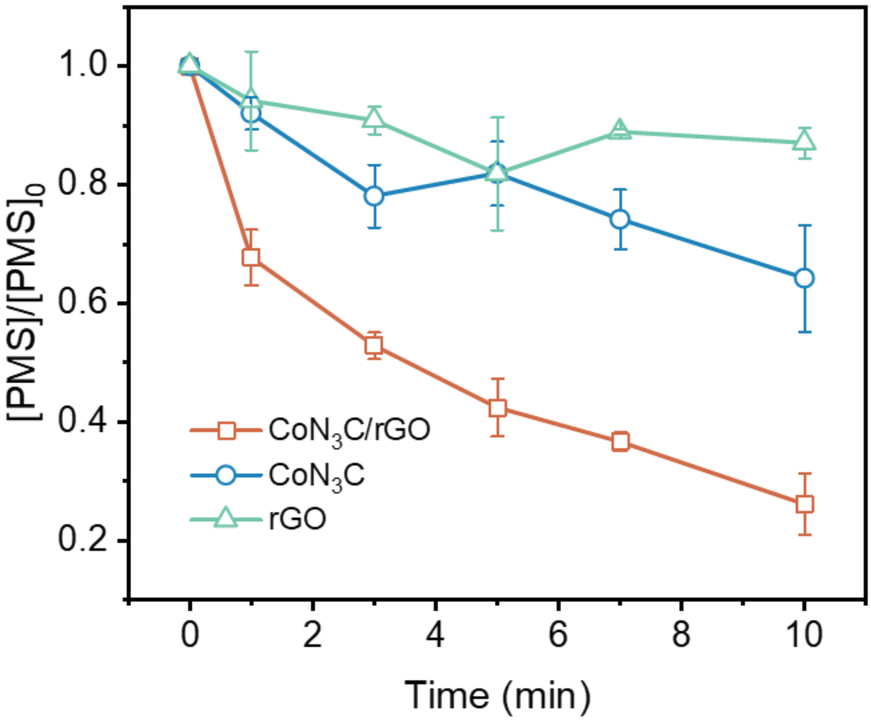

Supplementary Fig. 28. PMS utilization efficiency of different catalysts. PMS decomposition efficiency in CoN_3C/rGO/PMS, CoN_3C/PMS, and rGO/PMS systems. The error bars are standard deviation of three replicate tests (n= 3).

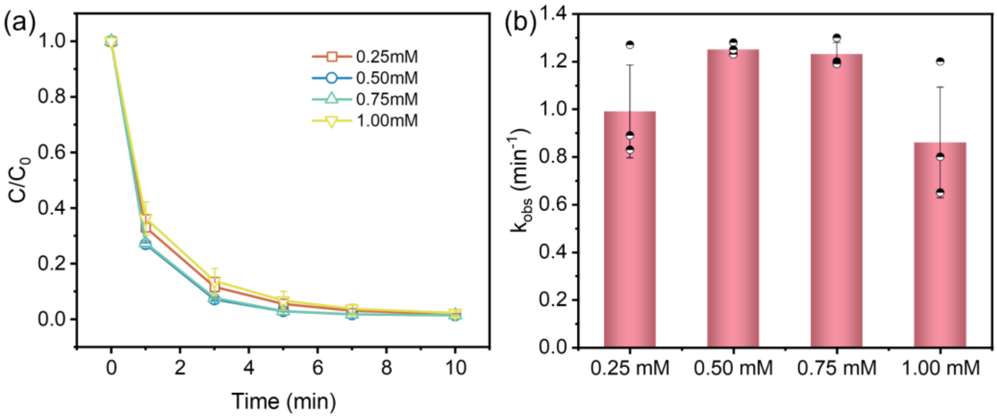

Supplementary Fig. 29. The effect of PMS concentration. (a) The degradation curves, (b) k_{obs} of BPA in CoN_3C/rGO/PMS with different PMS concentrations. Experiment conditions: [catalyst]= 0.05 g/L, [PMS]= 0.25, 0.50, 0.75, 1.00 mM, [BPA]= 10 mg/L, pH= 6.0, room temperature if not otherwise specified. The error bars are standard deviation of three replicate tests (n= 3).

The influence of PMS concentration was explored. In the range of 0.25 mM to 0.75 mM, with the increase of PMS concentration, k_{obs} showed a trend of rising and then stabilizing. Moreover, the BPA could be removed completely even in the low PMS concentration (0.25 mM). When the added PMS reached 1.00 mM, k_{obs} decreased, which might be due to the concentration of PMS beyond the maximum tolerance of the active site, and excess PMS would cause a self-scavenging effect for ROS.[19] Hence, a reasonable PMS concentration is conducive to ROS generation.

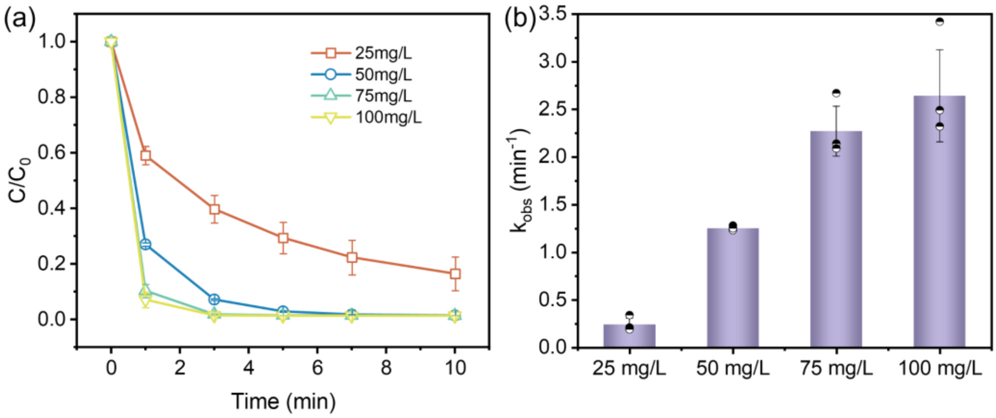

Supplementary Fig. 30. The effect of catalyst dosage. (a) The degradation curves, (b) k_{obs} of BPA in CoN_3C/rGO/PMS with different catalyst dosages. Experiment conditions: [catalyst]= 0.025, 0.050, 0.075, 0.100 g/L, [PMS]= 0.5 mM, [BPA]= 10 mg/L, pH= 6.0, room temperature if not otherwise specified. The error bars are standard deviation of three replicate tests (n= 3).

The catalyst dosage is also an important factor. With the rise of CoN_3C/rGO dosage from 25 mg/L to 75 mg/L, an obvious enhancement of k_{obs} and degradation efficiency could be observed, while this trend became slow after increasing to 100 mg/L, indicating that more catalyst would provide more active sites for PMS activation.

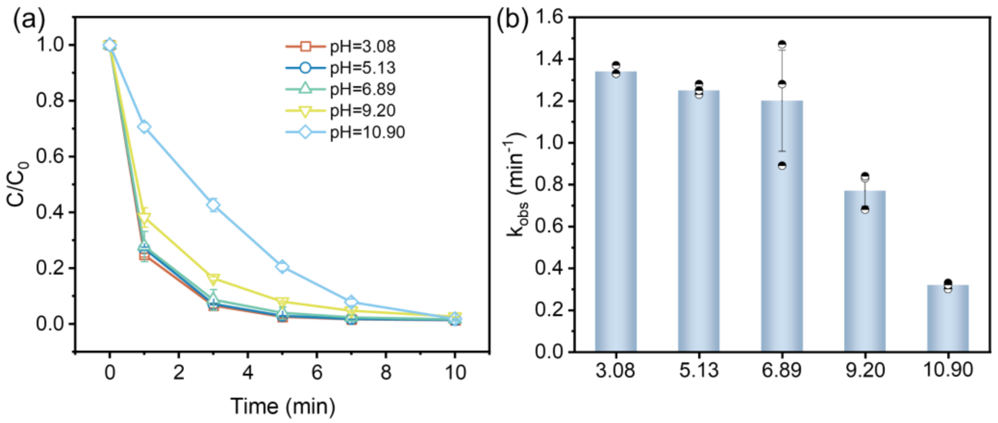

Supplementary Fig. 31. The effect of initial pH. (a) The degradation curves, (b) k_{obs} of BPA in CoN_3C/rGO/PMS with different initial pH. Experiment conditions: [catalyst]= 0.05 g/L, [PMS]= 0.5 mM, [BPA]= 10 mg/L, pH= 3.08, 5.13, 6.89, 9.20, 10.90, room temperature if not otherwise specified. The error bars are standard deviation of three replicate tests (n= 3).

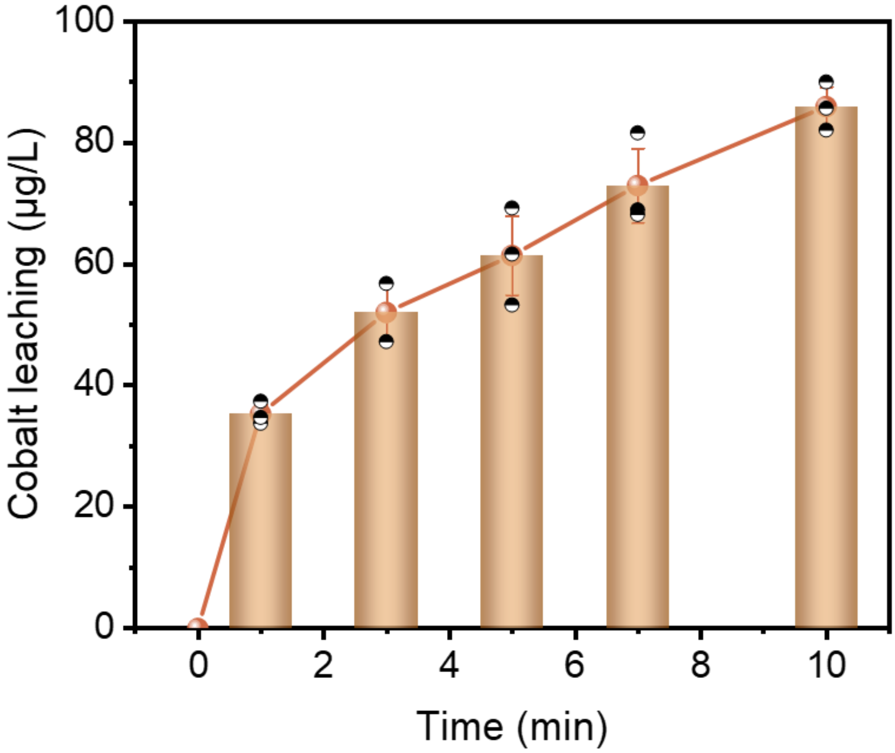

Supplementary Fig. 32. The metal leaching condition. The cobalt concentration within the degradation process. Experiment conditions: [catalyst]= 0.05 g/L, [PMS]= 0.5 mM, [BPA]= 10 mg/L, pH= 6.0, room temperature if not otherwise specified. The error bars are standard deviation of three replicate tests (n= 3).

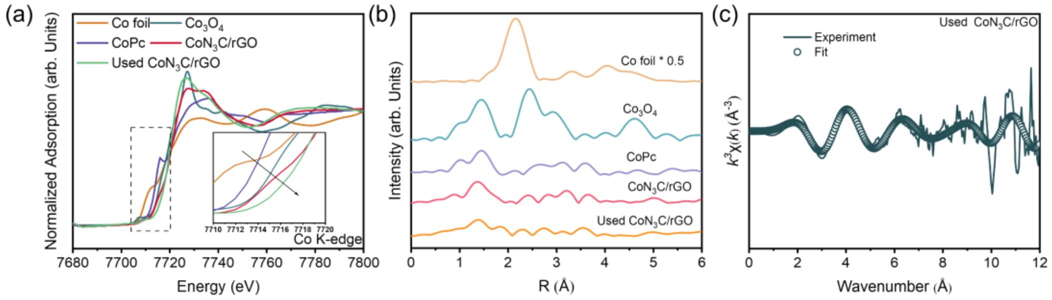

Supplementary Fig. 33. The coordination environment change after reaction of CoN3C/rGO. (a) Normalized Co k-edge XANES of CoN3C/rGO, used CoN3C/rGO and reference samples. (b) Fourier transform EXAFS of used CoN3C/rGO, CoN3C/rGO and reference samples. (c) EXAFS fitting analysis of used CoN3C/rGO in K space.

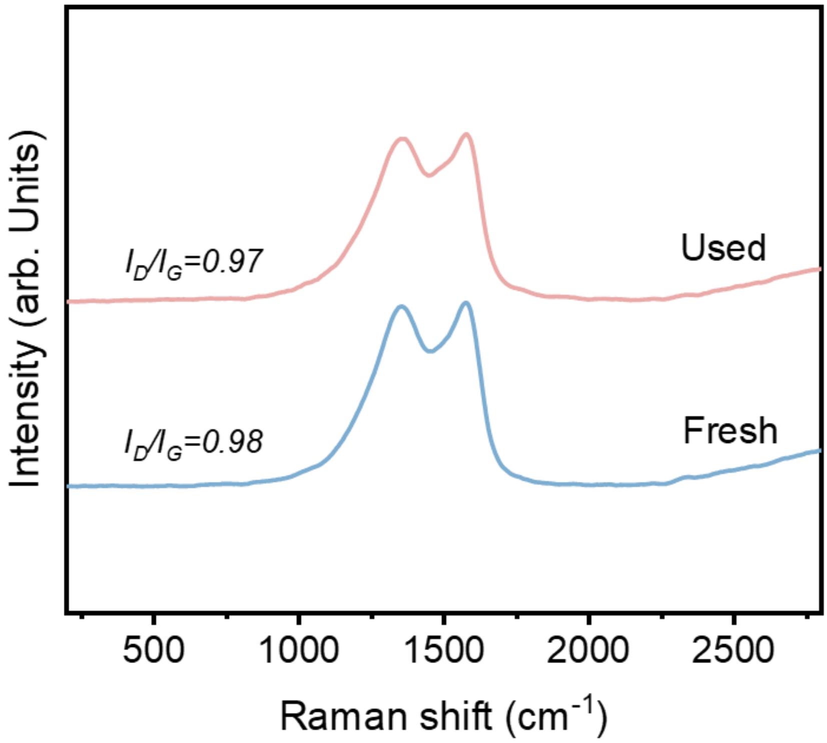

Supplementary Fig. 34. The change of catalyst after being used. The Raman spectra of fresh CoN_3C/rGO and used CoN_3C/rGO.

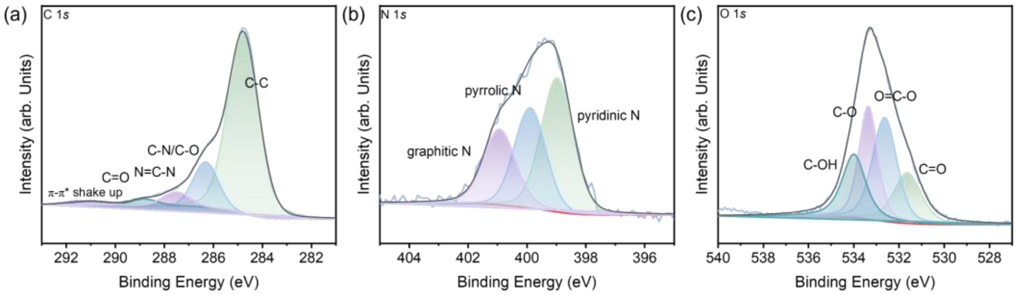

Supplementary Fig. 35. The chemical state of used CoN_3C/rGO. High-resolution XPS spectra of (a) C 1*s*, (b) N 1*s*, (c) O 1*s* for used CoN_3C/rGO.

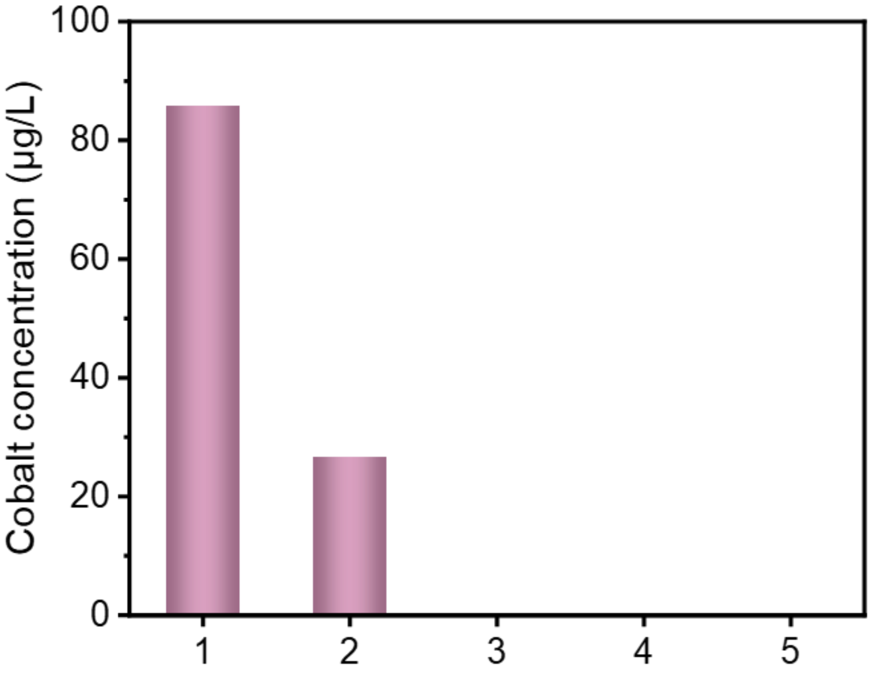

Supplementary Fig. 36. The stability of CoN_3C/rGO. The Co leaching concentrations after every cyclic experiment. Experiment conditions: [catalyst]= 0.05 g/L, [PMS]= 0.5 mM, [BPA]= 10 mg/L, pH= 6.0, room temperature if not otherwise specified.

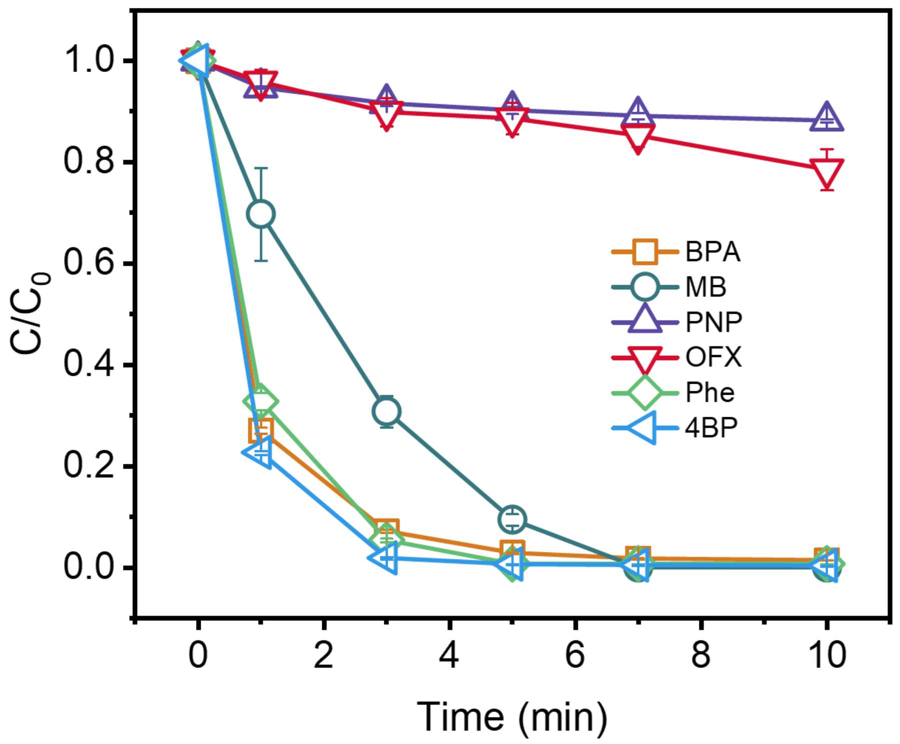

Supplementary Fig. 37. The degradation results of different pollutants. The degradation curves of different pollutants. Experiment conditions: [catalyst]= 0.05 g/L, [PMS]= 0.5 mM, [Pollutant]= 10 mg/L, pH= 6.0, room temperature if not otherwise specified. The error bars are standard deviation of three replicate tests (n= 3).

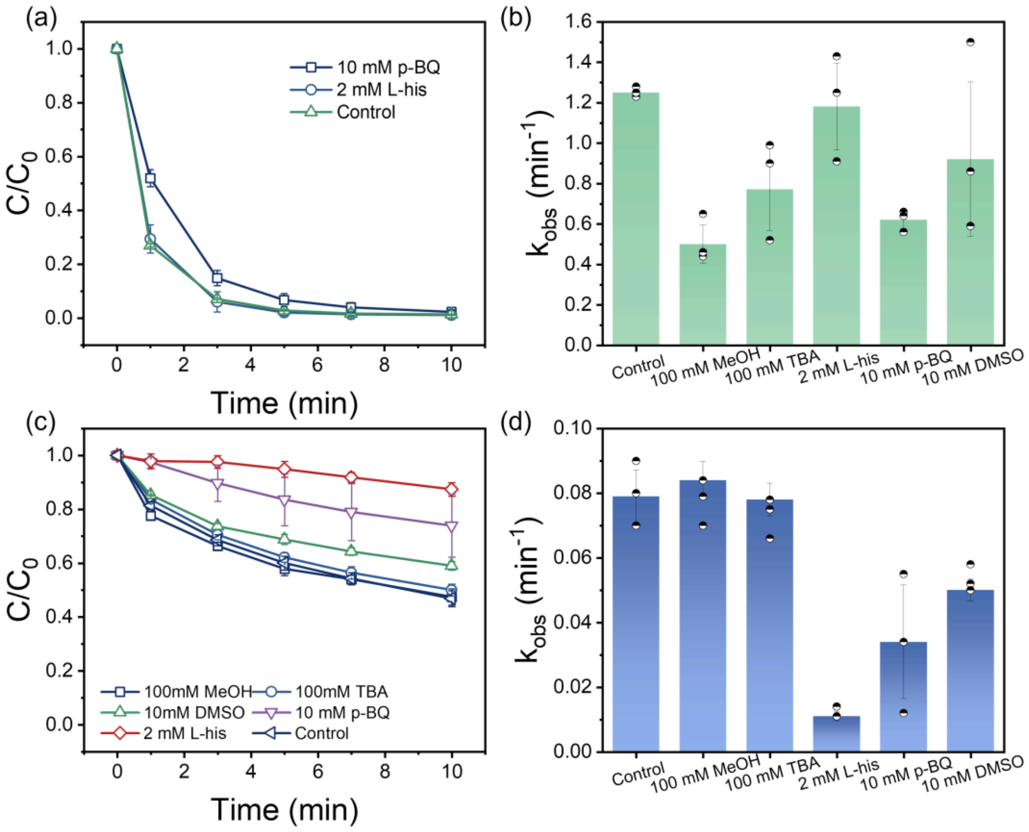

Supplementary Fig. 38. The quenching experiment results. The degradation curves of BPA degradation in (a) CoN_3C/rGO/PMS, (c) CoN_3C/PMS after adding quenching agents. The k_{obs} of BPA degradation in (b) CoN_3C/rGO/PMS, (d) CoN_3C/PMS after adding quenching agents. Experiment conditions: [catalyst]= 0.05 g/L, [PMS]= 0.5 mM, [BPA]= 10 mg/L, pH= 6.0, room temperature if not otherwise specified. The error bars are standard deviation of three replicate tests (n= 3).

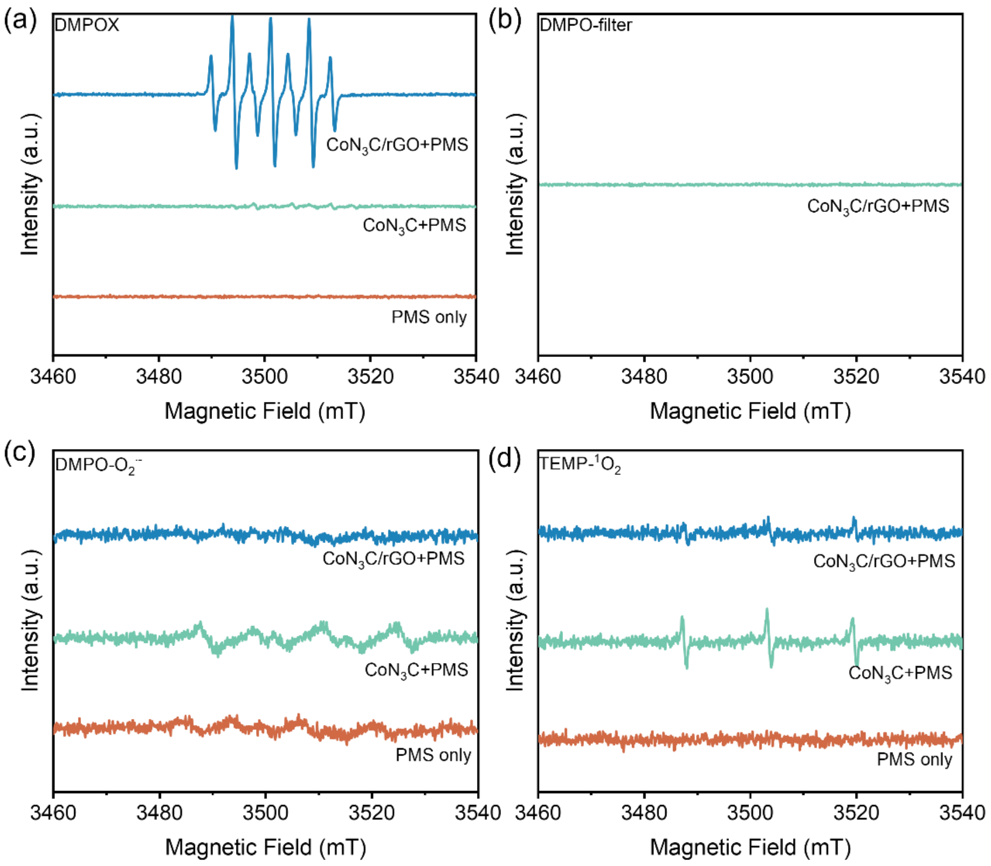

Supplementary Fig. 39. The EPR results. The EPR spectra of (a) DMPOX, (b) filtered DMPO-·OH and $SO_4^{\cdot-}$, (c) DMPO-$O_2^{\cdot-}$, (d) TEMP-$^1O_2$.

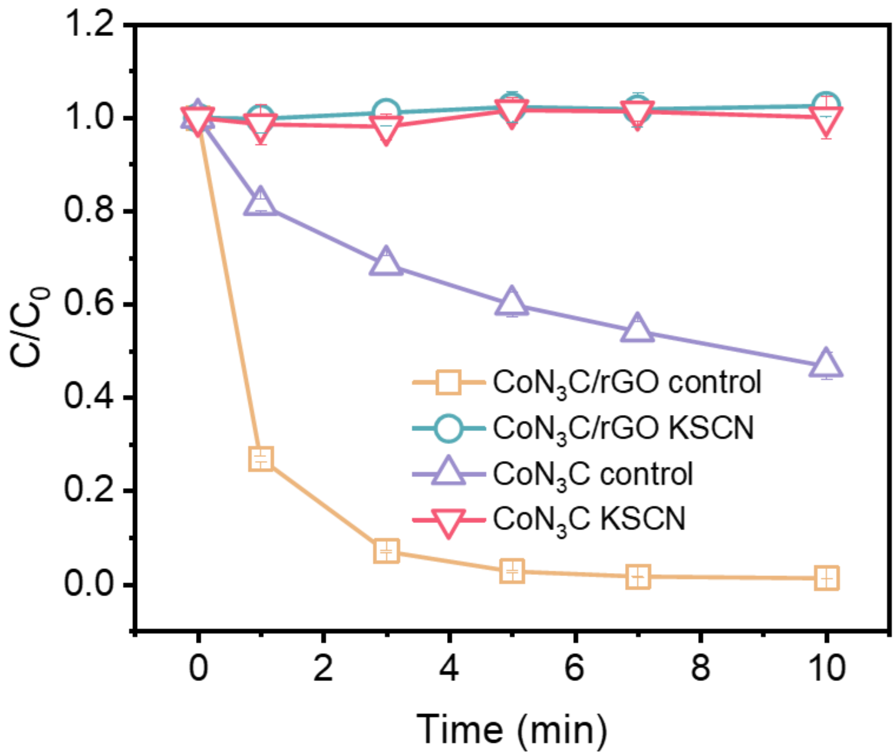

Supplementary Fig. 40. The investigation of active site. The BPA degradation curves under KSCN added to the $CoN_3C/rGO/PMS$ and CoN_3C/PMS system. Experiment conditions: [catalyst]= 0.05 g/L, [PMS]= 0.5 mM, [BPA]= 10 mg/L, pH= 6.0, room temperature if not otherwise specified. The error bars are standard deviation of three replicate tests (n= 3).

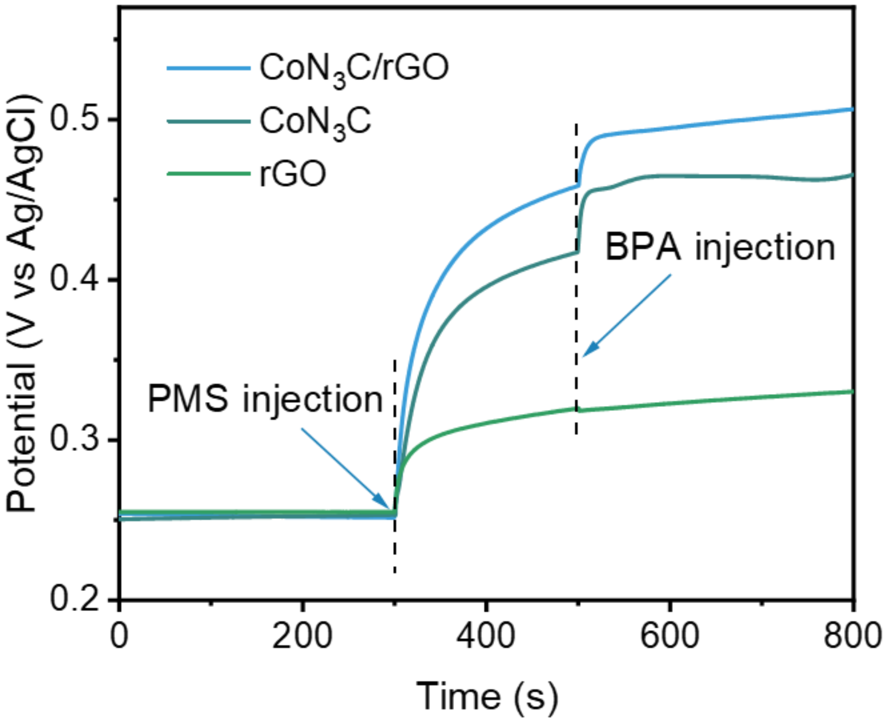

Supplementary Fig. 41. The OCP results. The OCP change of different catalysts upon PMS and BPA addition.

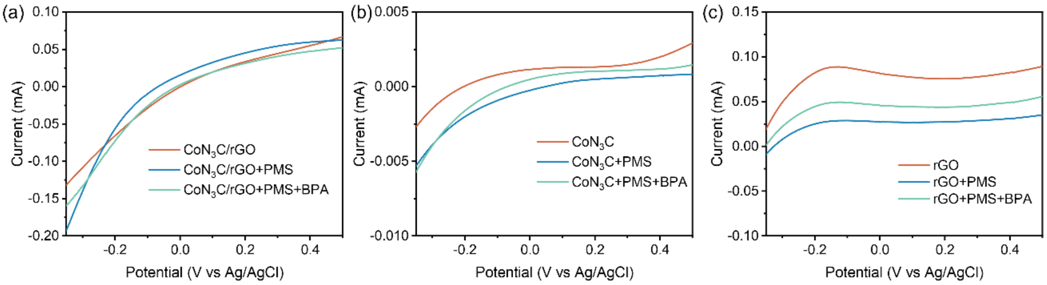

Supplementary Fig. 42. The LSV results. The LSV changed after adding PMS and BPA for (a) CoN_3C/rGO system, (b) CoN_3C system, and (c) rGO system.

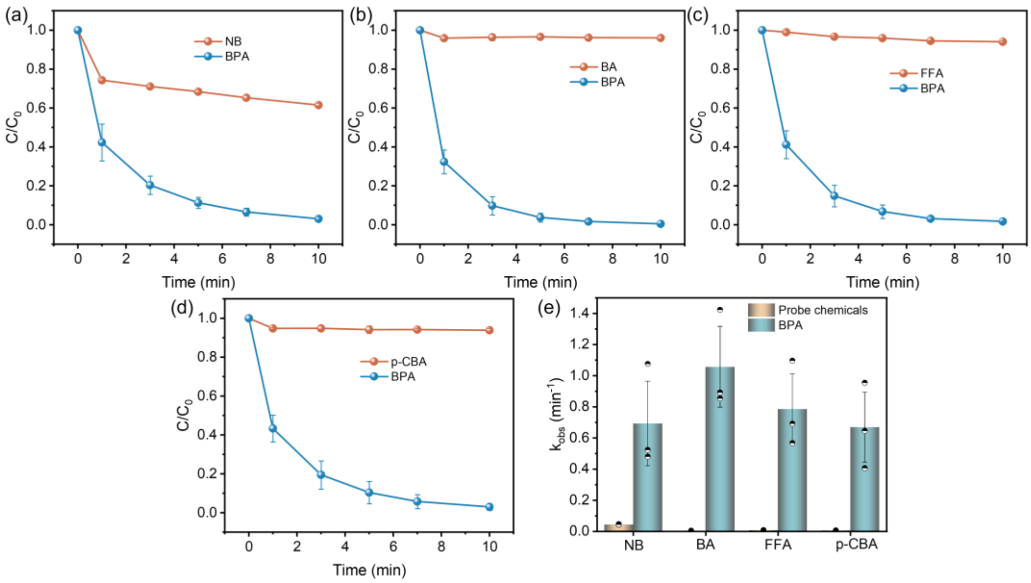

Supplementary Fig. 43. The ROS quantitative results of CoN3C/rGO/PMS. The concentration change of chemical probe (a) NB, (b) BA, (c) FFA, (d) p-CBA. (e) The k_{obs} of the chemical probe reaction. The error bars are standard deviation of three replicate tests (n= 3).

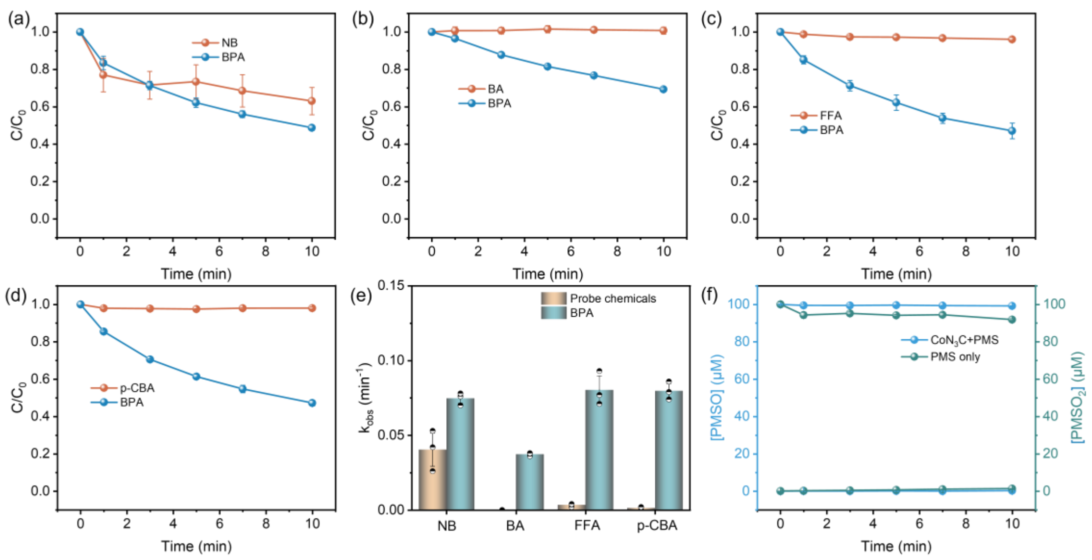

Supplementary Fig. 44. The ROS quantitative results of CoN3C/PMS. The concentration change of chemical probe (a) NB, (b) BA, (c) FFA, (d) p-CBA. (e) The k_{obs} of the chemical probe reaction. (f) The PMSO and $PMSO_2$ concentration change. The error bars are standard deviation of three replicate tests (n= 3).

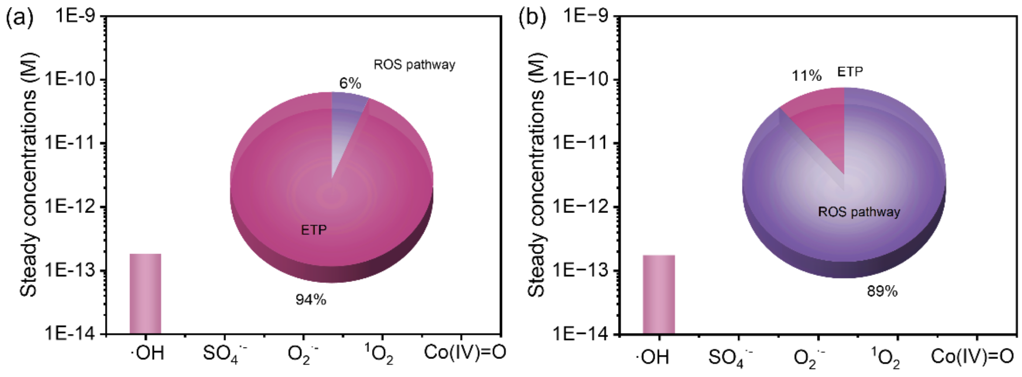

Supplementary Fig. 45. The contribution of various paths. The steady concentrations of the chemical probe and the contribution of various paths in (a) CoN_3C/rGO/PMS, (b) CoN_3C/PMS.

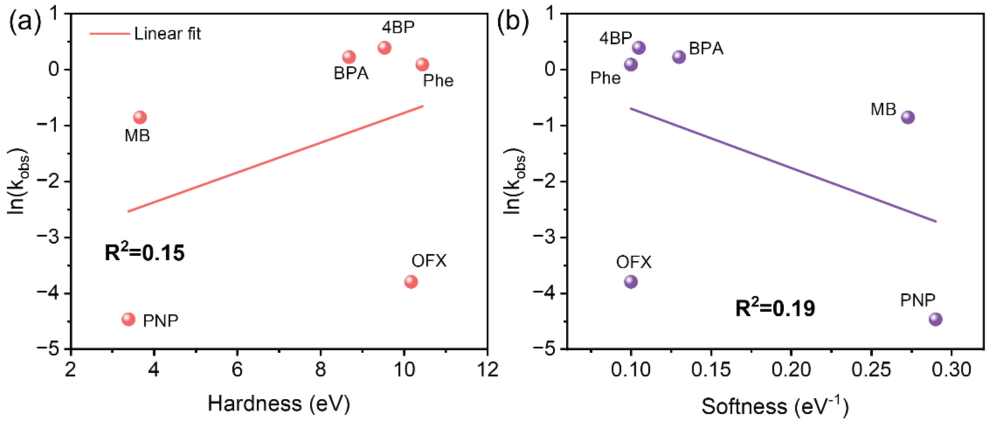

Supplementary Fig. 46. The relationship between ln(k_{obs}) and hardness/softness. The Linear fitting results between ln(k_{obs}) and (a) hardness, (b) softness. The error bars are standard deviation of three replicate tests (n= 3).

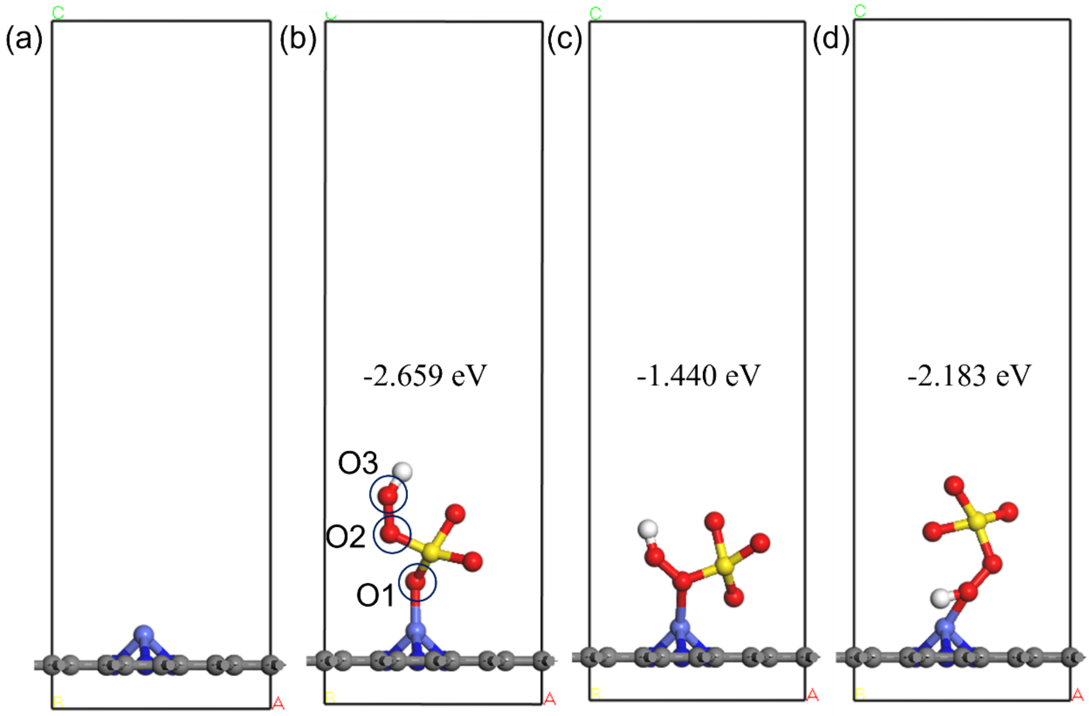

Supplementary Fig. 47. The optimized adsorption of PMS on CoN_3C. (a) The structure of CoN_3C. (b) The adsorption of PMS on CoN_3C by O1 site. (c) The adsorption of PMS on CoN_3C by O2 site. (d) The adsorption of PMS on CoN_3C by O3 site.

The O atom of PMS can be categorized to 3 types: (1) Three sulfonyl O atoms (O1 atom). (2) The peroxy O atom (O2 atom). (3) Hydroxyl O atom (O3 atom). When the PMS molecule adsorbs on Co site with O1 atom, the adsorption energy is the largest.

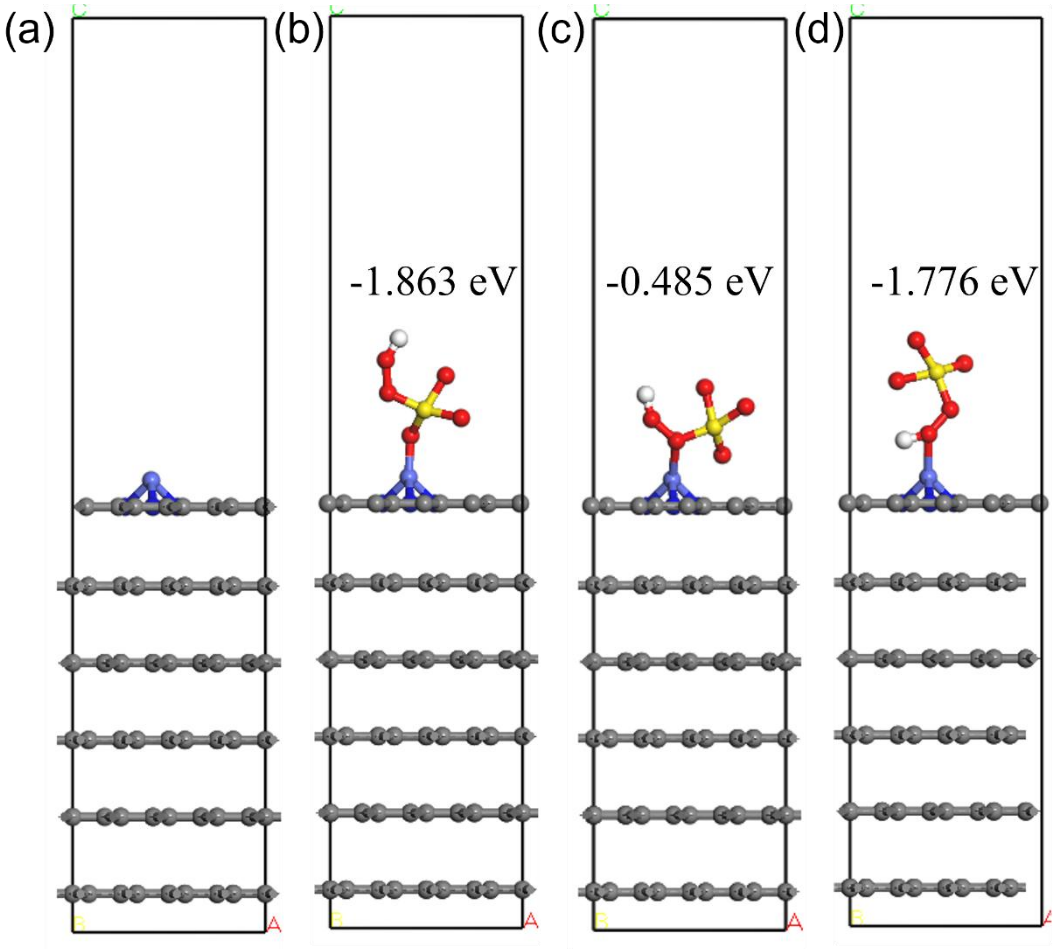

Supplementary Fig. 48. The optimized adsorption of PMS on CoN_3C/rGO. (a) The structure of CoN_3C/rGO. (b) The adsorption of PMS on CoN_3C/rGO by O1 site. (c) The adsorption of PMS on CoN_3C/rGO by O2 site. (d) The adsorption of PMS on CoN_3C/rGO by O3 site.

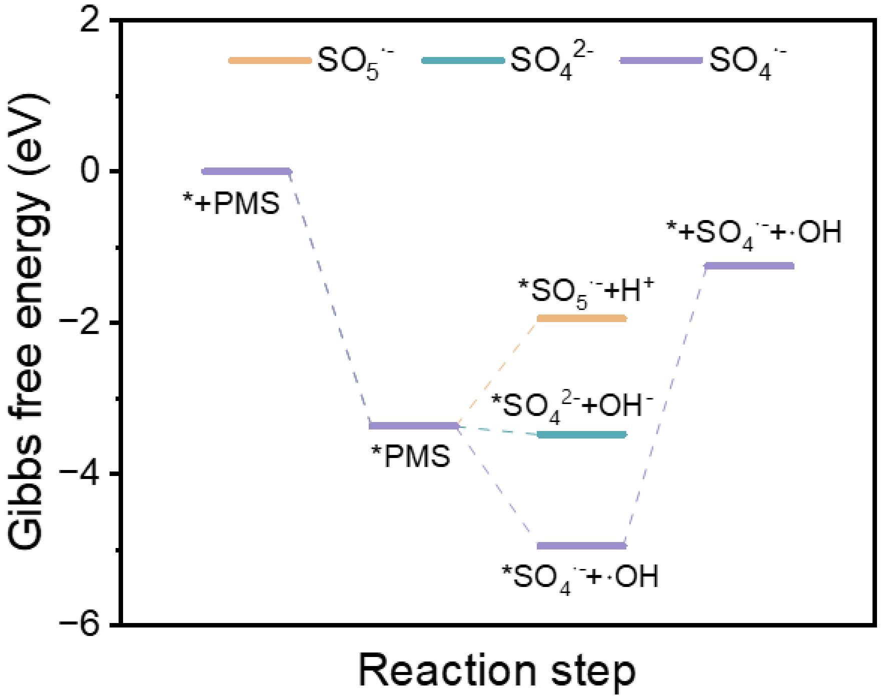

Supplementary Fig. 49. The thermodynamical trends of reaction between CoN_3C and PMS. Gibbs free energy of various CoN_3C/PMS reaction pathways.

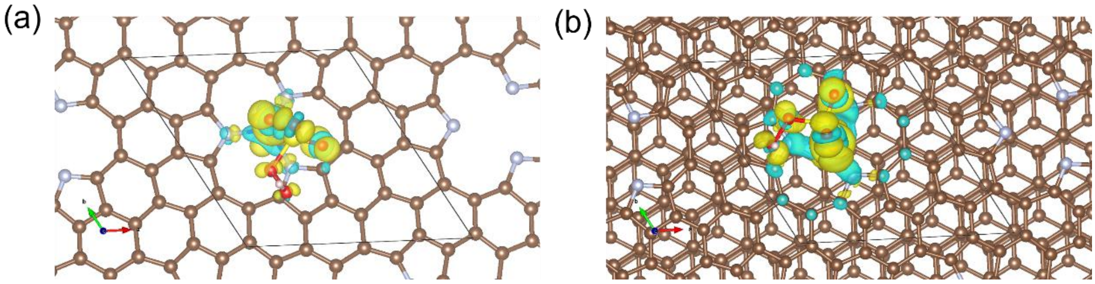

Supplementary Fig. 50. The differential charge density results. (a) The top images of the differential charge density between CoN_3C and PMS. (b) The top images of the differential charge density between CoN_3C/rGO and PMS.

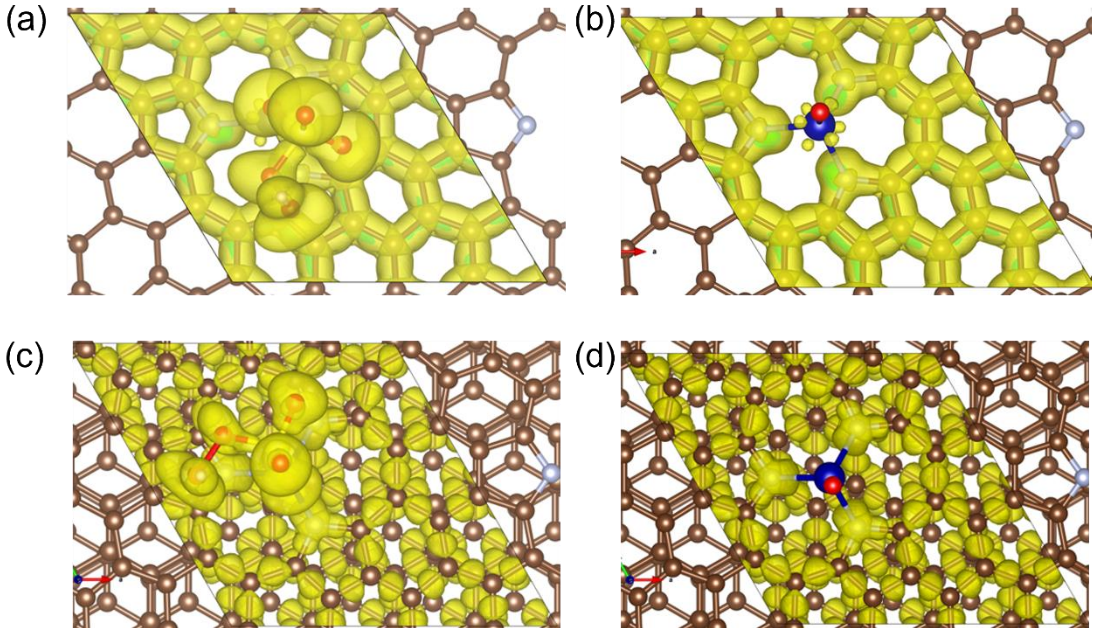

Supplementary Fig. 51. The ELF results of catalysts after reaction. (a, b) 3D ELF images of CoN_3C after PMS reaction under different cross-sections. (c, d) 3D ELF images of CoN_3C/rGO after PMS reaction under different cross-sections.

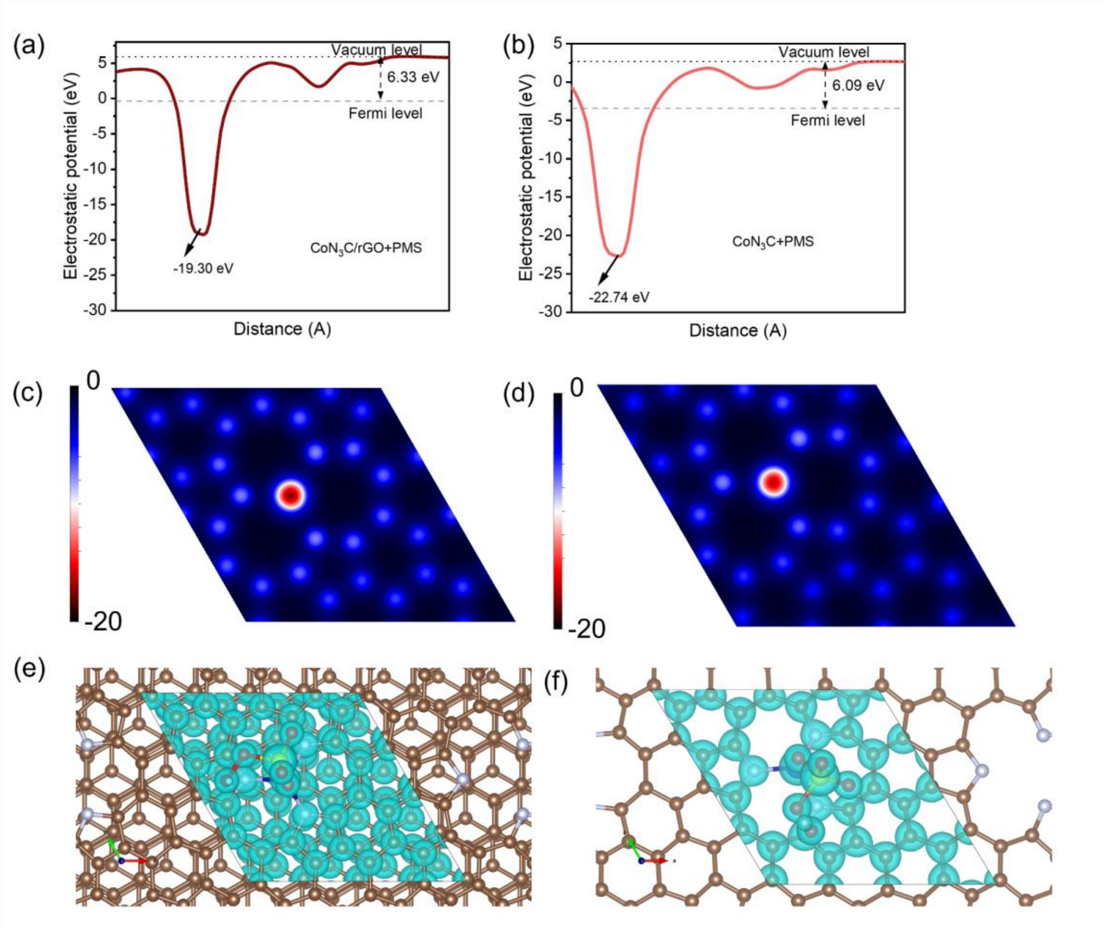

Supplementary Fig. 52. Work function and electrostatic potential results of catalysts after reaction. Work functions of (a) CoN_3C/rGO, (b) CoN_3C after PMS reaction. 2D electrostatic potential images of (c) CoN_3C/rGO, (d) CoN_3C after PMS reaction. 3D electrostatic potential images of (e) CoN_3C/rGO, (f) CoN_3C after PMS reaction (The green area represents the equipotential surface where the electrostatic potential is -20 eV).

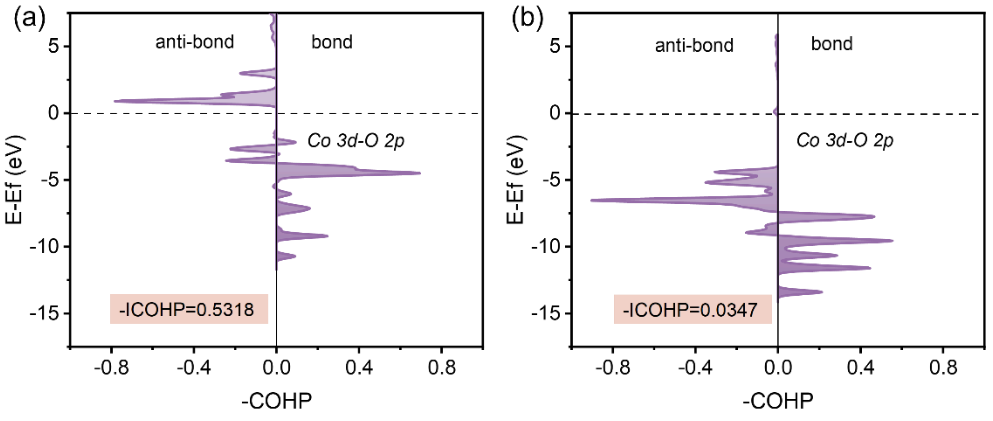

Supplementary Fig. 53. The COHP results. The COHP between Co 3*d* of (a) CoN_3C/rGO, (b) CoN_3C and O 2*p* of PMS.

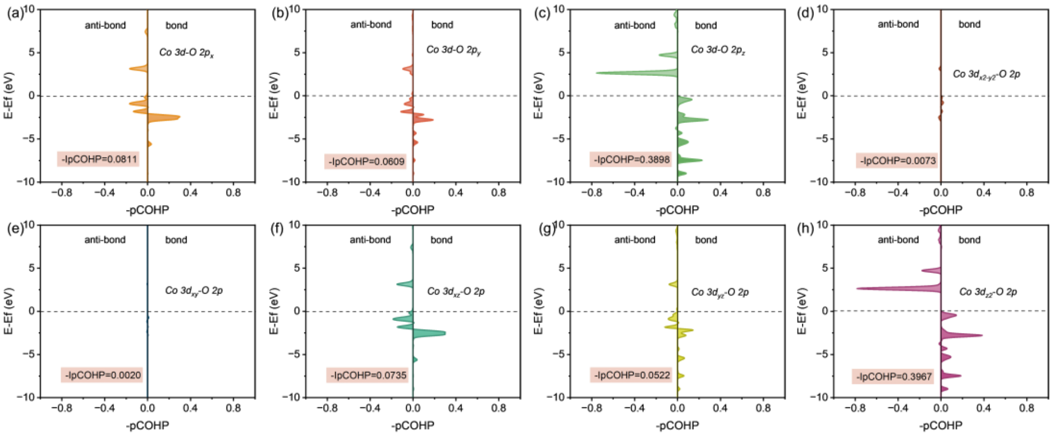

Supplementary Fig. 54. The COHP results of CoN_3C/rGO/PMS. The COHP of (a) Co 3*d*-O 2p_x, (b) Co 3*d*-O 2p_y, (c) Co 3*d*-O 2p_z, (d) Co 3$d_{x2\text{-}y2}$-O 2*p*, (e) Co 3d_{xy}-O 2*p*, (f) Co 3d_{xz}-O 2*p*, (g) Co 3d_{yz}-O 2*p*, (h) Co 3d_{z2}-O 2*p*.

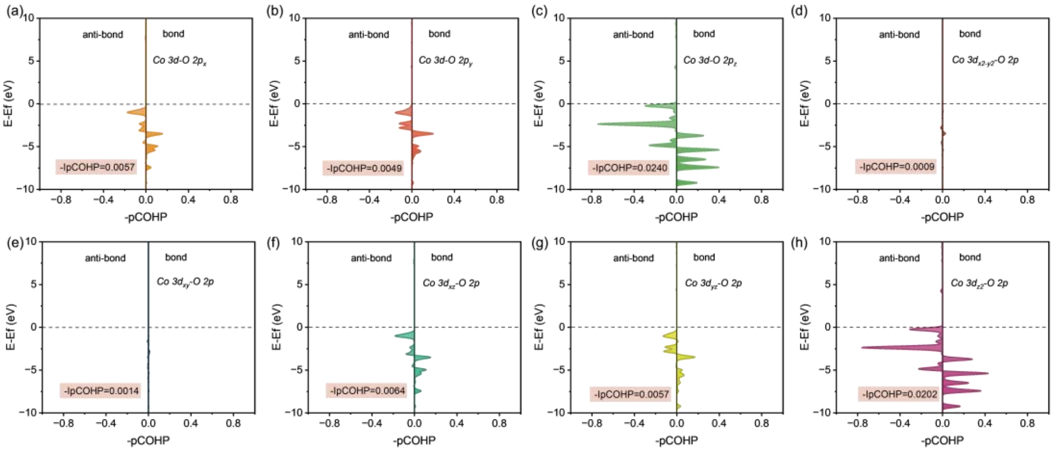

Supplementary Fig. 55. The COHP results of CoN3C/PMS. The COHP of (a) Co 3*d*-O 2p_x, (b) Co 3*d*-O 2p_y, (c) Co 3*d*-O 2p_z, (d) Co 3d_{x2-y2}-O 2*p*, (e) Co 3d_{xy}-O 2*p*, (f) Co 3d_{xz}-O 2*p*, (g) Co 3d_{yz}-O 2*p*, (h) Co 3d_{z2}-O 2*p*.

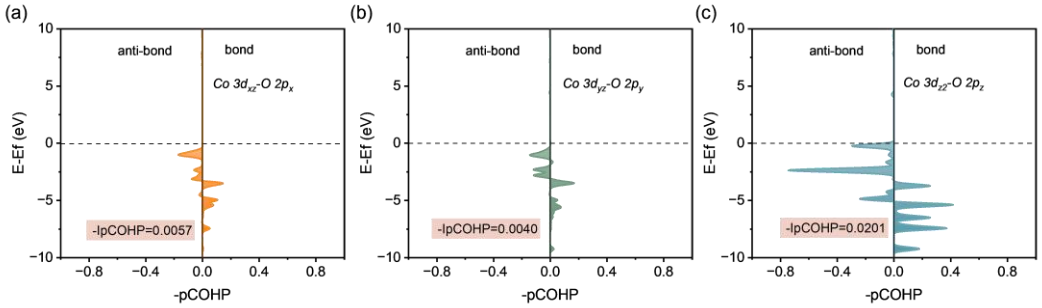

Supplementary Fig. 56. The COHP results of CoN3C/PMS. The COHP of (a) Co 3d_{xz}-O 2p_x, (b) Co 3d_{yz}-O 2p_y, (c) Co 3d_{z2}-O 2p_z.

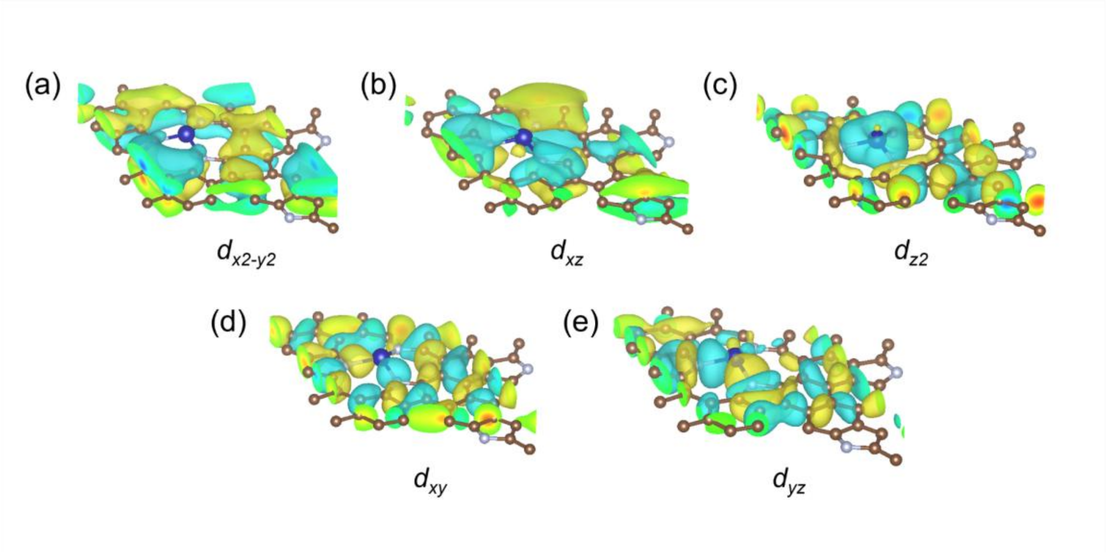

Supplementary Fig. 57. The electron cloud of *d* orbitals in CoN_3C. The wave function plot of (a) $d_{x2\text{-}y2}$, (b) d_{xz}, (c) d_{z2}, (d) d_{xy}, (e) d_{yz}.

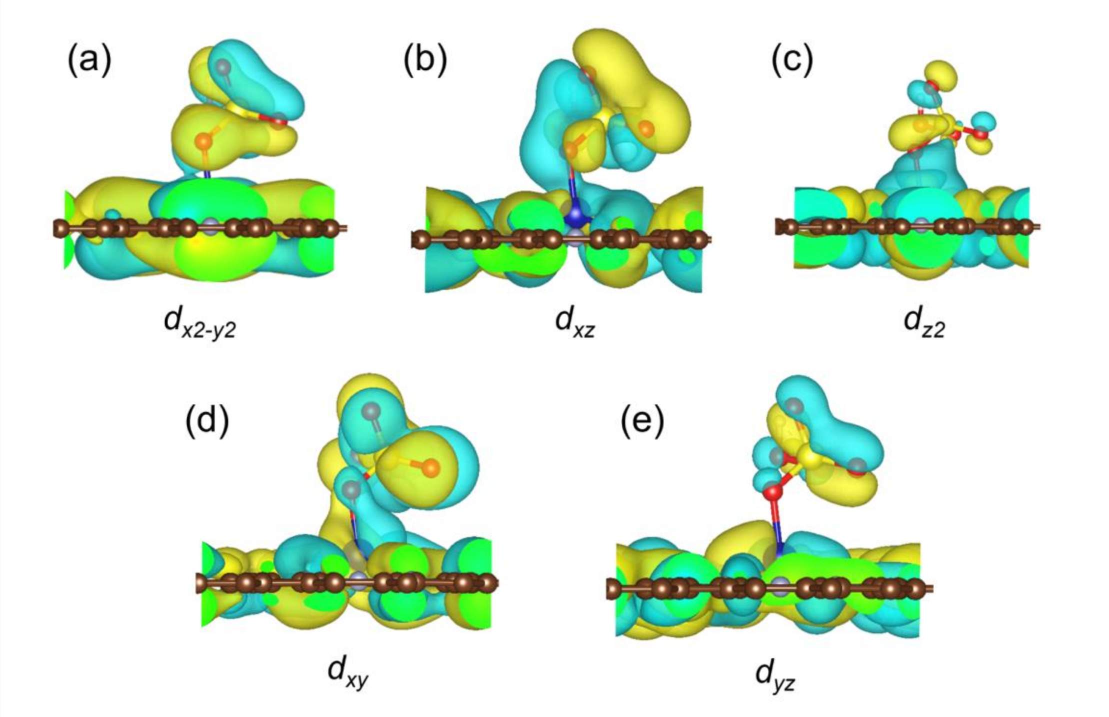

Supplementary Fig. 58. The electron cloud of *d* orbitals in CoN_3C/rGO/PMS. The wave function plot of (a) d_{x2-y2}, (b) d_{xz}, (c) d_{z2}, (d) d_{xy}, (e) d_{yz}.

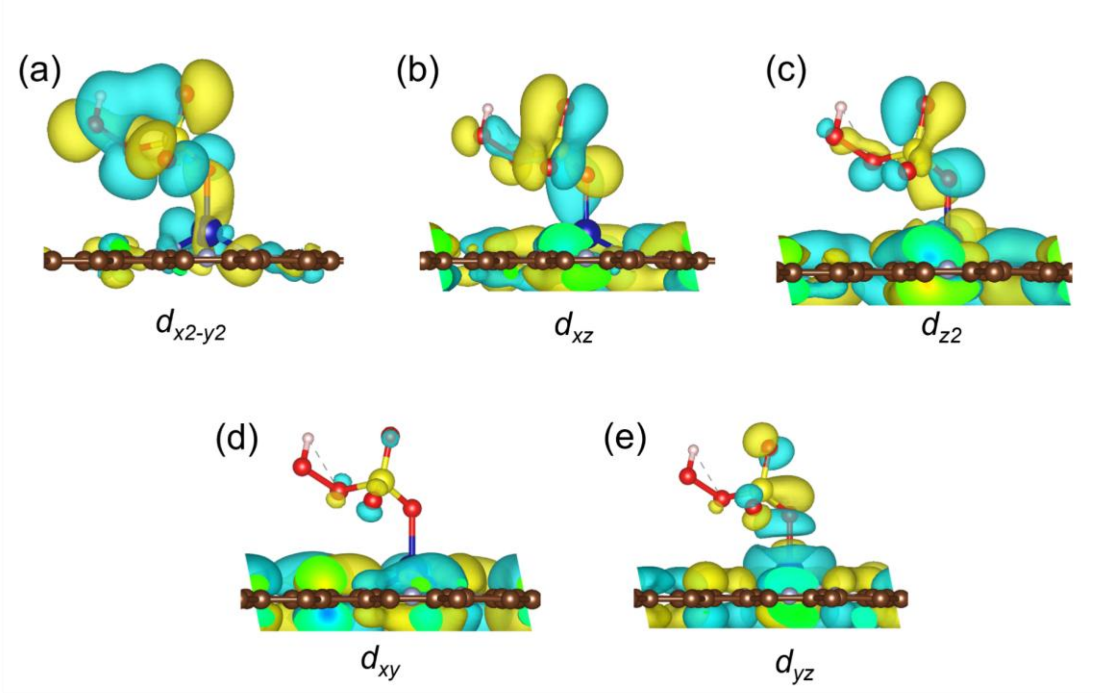

Supplementary Fig. 59. The electron cloud of *d* orbitals in CoN_3C/PMS. The wave function plot of (a) d_{x2-y2}, (b) d_{xz}, (c) d_{z2}, (d) d_{xy}, (e) d_{yz}.

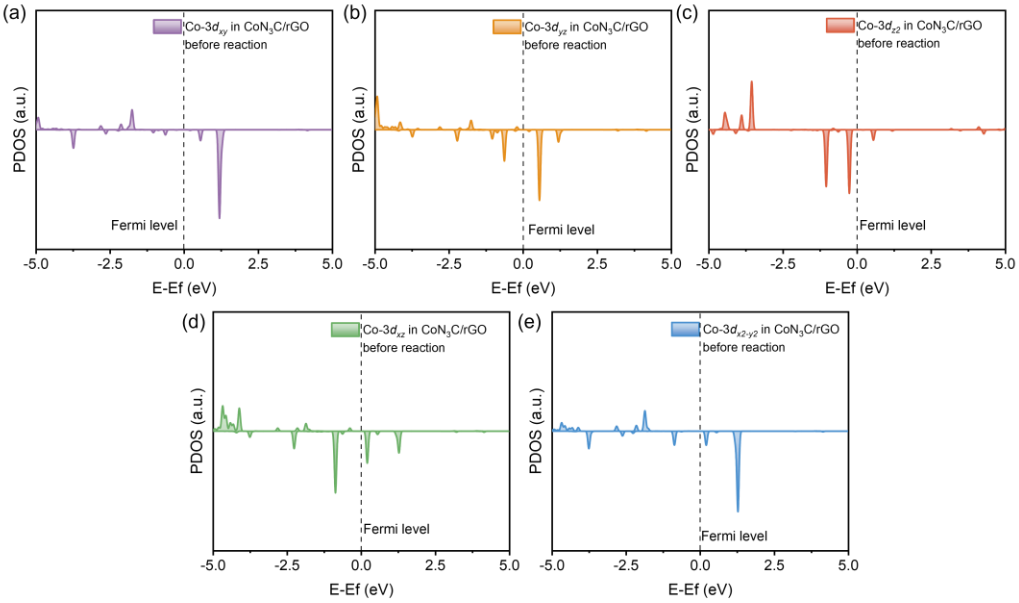

Supplementary Fig. 60. The PDOS results of CoN_3C/rGO. The PDOS of (a) Co 3d_{xy}, (b) Co 3d_{yz}, (c) Co 3d_{z2}, (d) Co 3d_{xz}, (e) Co 3$d_{x2\text{-}y2}$.

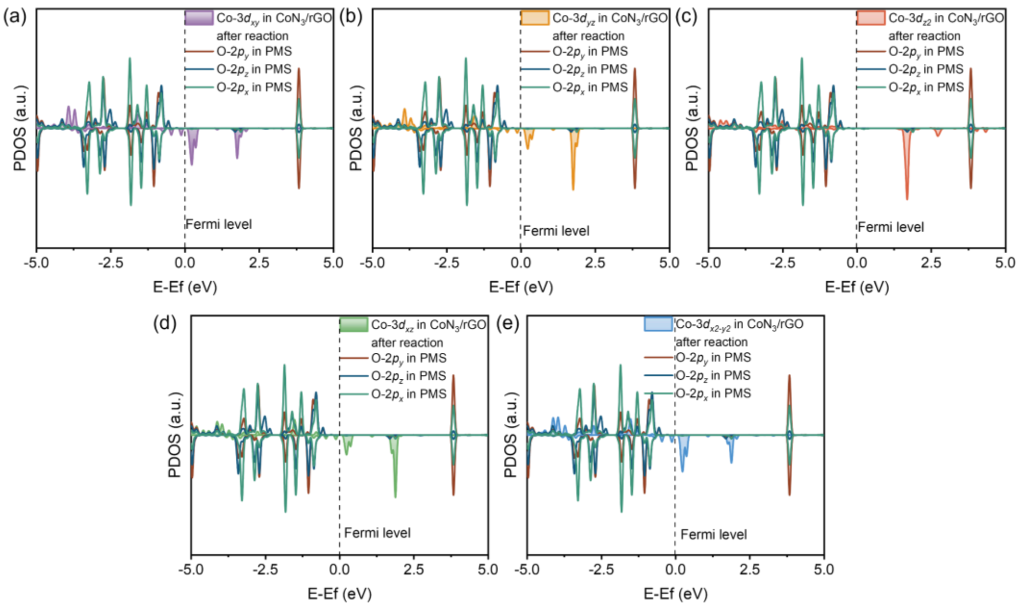

Supplementary Fig. 61. The PDOS results of CoN_3C/rGO after reaction. The PDOS of (a) Co 3d_{xy} and O 2p, (b) Co 3d_{yz} and O 2p, (c) Co 3d_{z2} and O 2p, (d) Co 3d_{xz} and O 2p, (e) Co 3$d_{x2\text{-}y2}$ and O 2p.

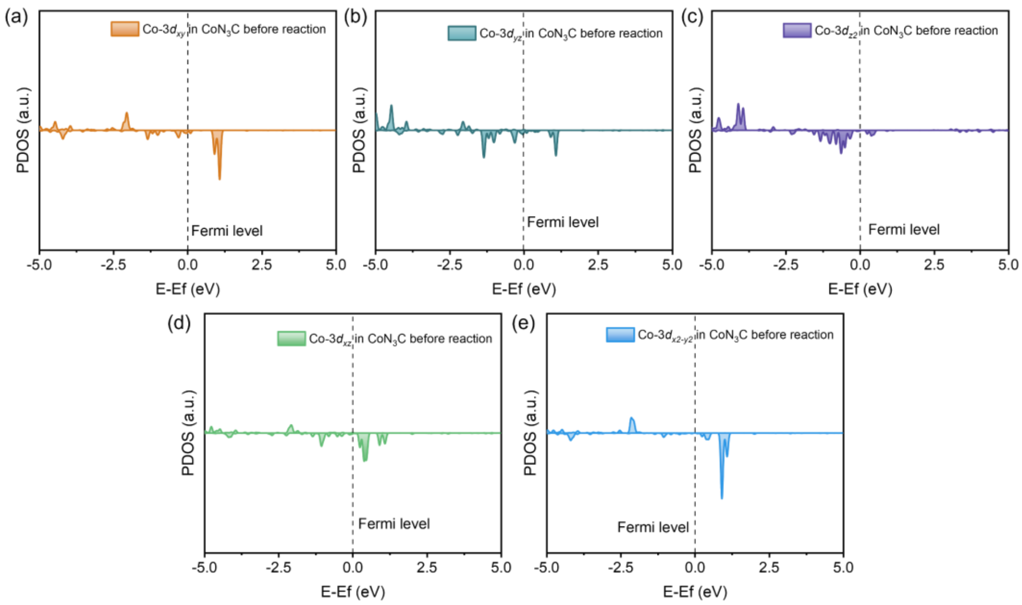

Supplementary Fig. 62. The PDOS results of CoN_3C. The PDOS of (a) Co 3d_{xy}, (b) Co 3d_{yz}, (c) Co 3d_{z2}, (d) Co 3d_{xz}, (e) Co 3$d_{x2\text{-}y2}$.

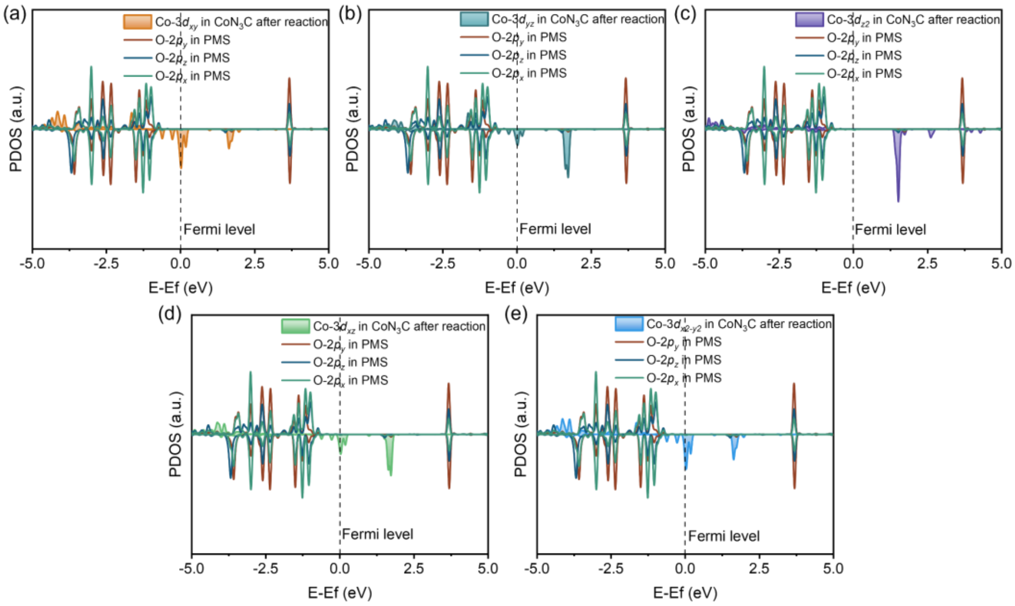

Supplementary Fig. 63. The PDOS results of CoN3C after reaction. The PDOS of (a) Co 3d_{xy} and O 2p, (b) Co 3d_{yz} and O 2p, (c) Co 3d_{z2} and O 2p, (d) Co 3d_{xz} and O 2p, (e) Co 3d_{x2-y2} and O 2p.

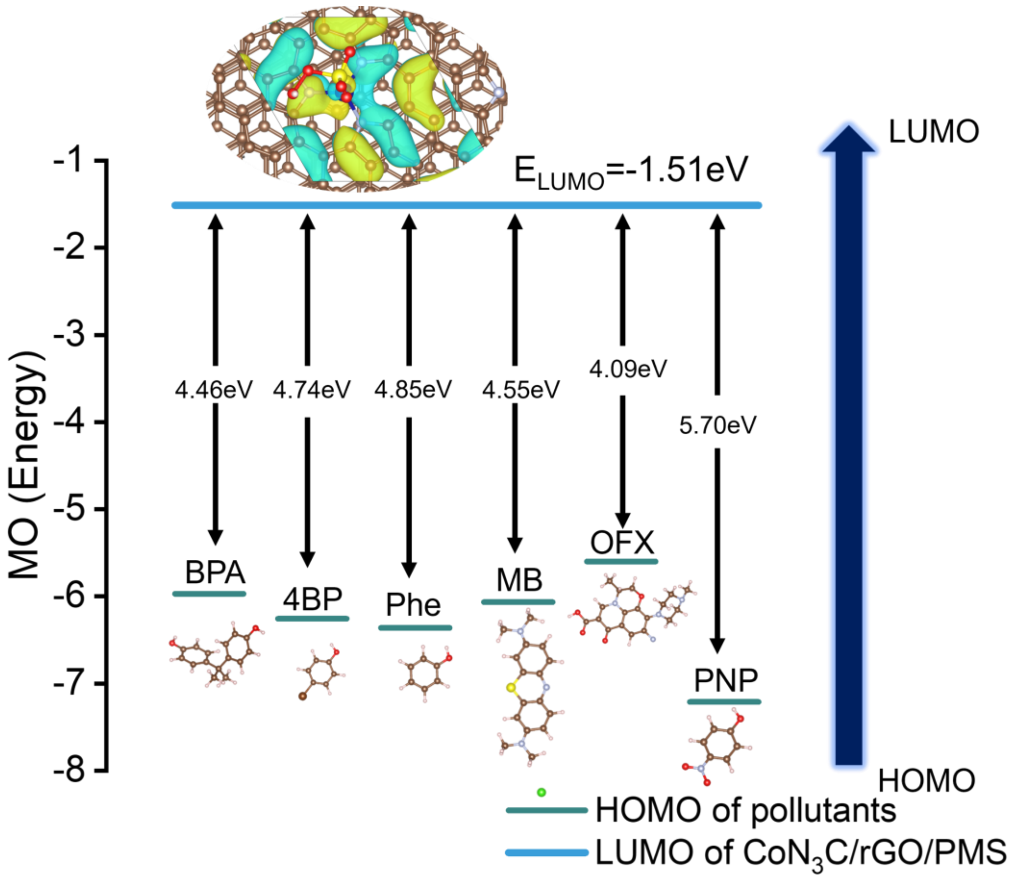

Supplementary Fig. 64. The pollutant-dependent selectivity from frontier orbital theory. Energy gap between HOMO of various pollutants and LUMO of CoN_3C/rGO/PMS.

The specific orbital/electronic interactions between CoN_3C/rGO/PMS system and the six tested contaminants was analyzed through Fukui function and HOMO-LUMO region (Supplementary Figs. 64-69, Supplementary Tables 11-14 and Supplementary Datas 1-2). In fact, the ETP process essentially involves the abstraction of electrons from the pollutant by the catalyst-PMS* complex. Therefore, the propensity of a pollutant to act as an electron donor governs its reactivity. The Fukui function exhibit sites most susceptible to nucleophilic attack, provides critical insights. Electron-rich compounds like Phe and 4-BP exhibit high f^- values on their hydroxyl oxygen and adjacent carbon atoms, indicating readily available electron density for transfer, leading to their rapid degradation. For BPA, although individual atomic f^- values are moderate, its two phenolic rings constitute an extended, delocalized π-system. Combined with its

high HOMO level, enables BPA to act as an exceptionally efficient and collective electron donor via the ETP pathway. In contrast, PNP, bearing a strong electron-withdrawing nitro group, displays a significantly lowered overall electron density and higher energy barrier for electron donation, resulting in its sluggish removal, which was verified by the evidence that it shows the lowest HOMO although it has similar maximum f^- value to Phe. For larger, more complex molecules like OFX and MB, while specific atoms (e.g., the piperazinyl N in OFX) show moderate f^- values, their overall reactivity is further hampered by steric hindrance and site distribution. Specifically, the electrophilic attack region and HOMO are mainly distributed on the rings within the OFX molecule. For MB, the similar regions are localized on the chloride counterion, rather than conjugated phenothiazine ring system of the cationic dye itself. This structure-activity relationship powerfully corroborates that the degradation is governed by an electron-transfer pathway rather than non-selective radical attack.

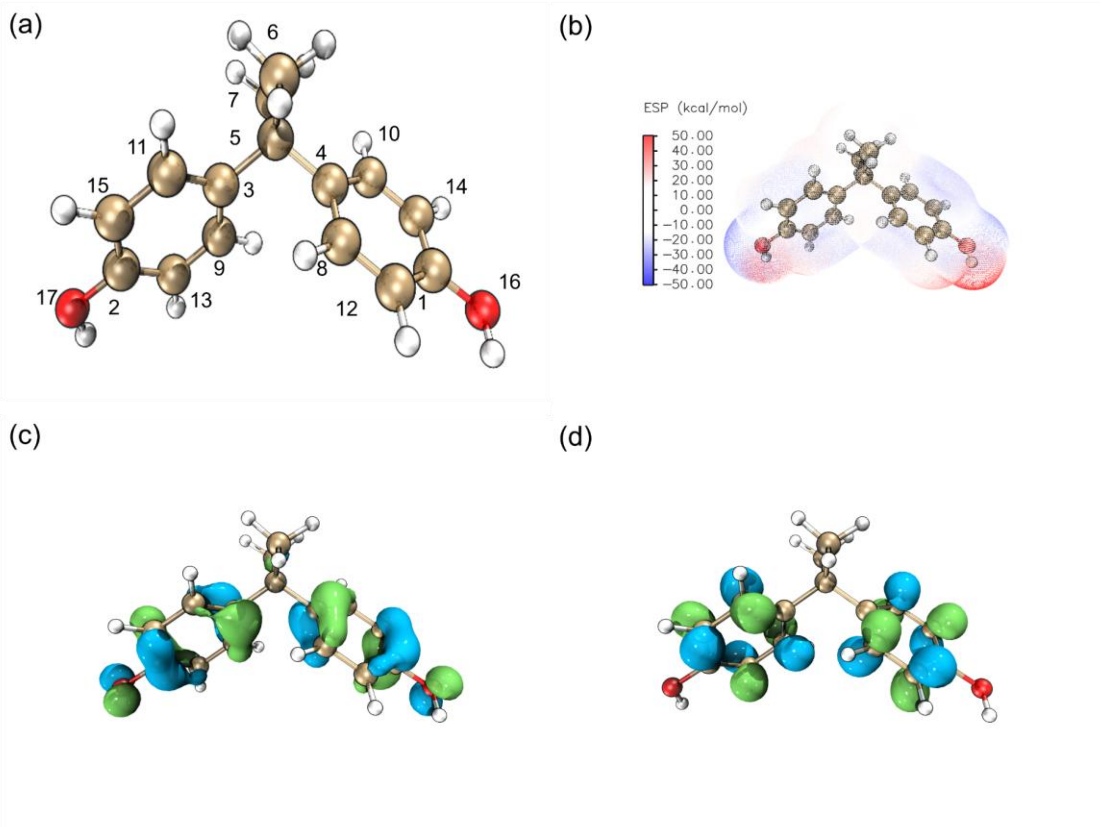

Supplementary Fig. 65. The molecular characteristics of BPA. (a) The molecular structure of BPA. (b) The electrostatic potential distribution of BPA. (c) The HOMO of BPA. (d) The LUMO of BPA.

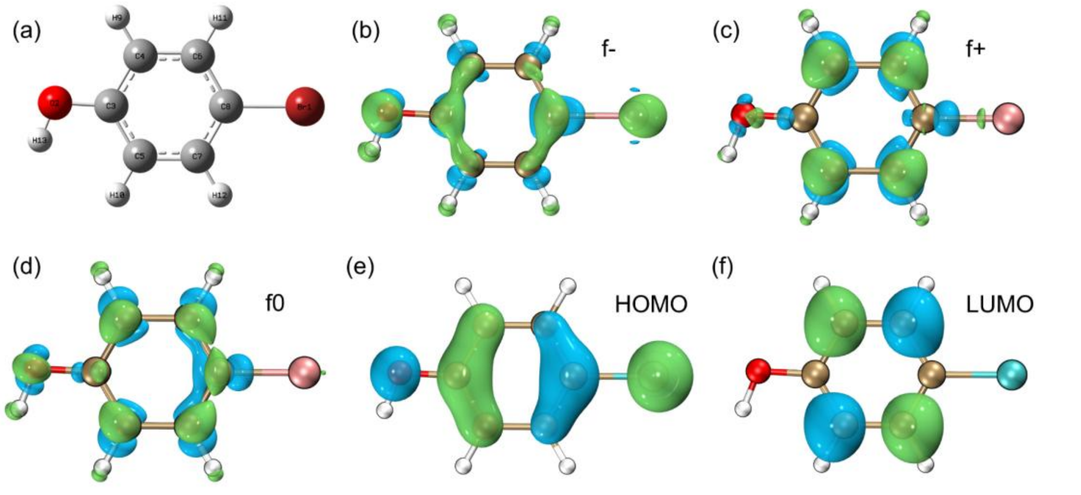

Supplementary Fig. 66. The structure and chemical properties of 4-BP. The (a) optimized chemical structure, (b) Fukui index (f^-) region, (c) Fukui index (f^+) region, (d) Fukui index (f^0) region, (e) HOMO region, (f) LUMO region of 4-BP.

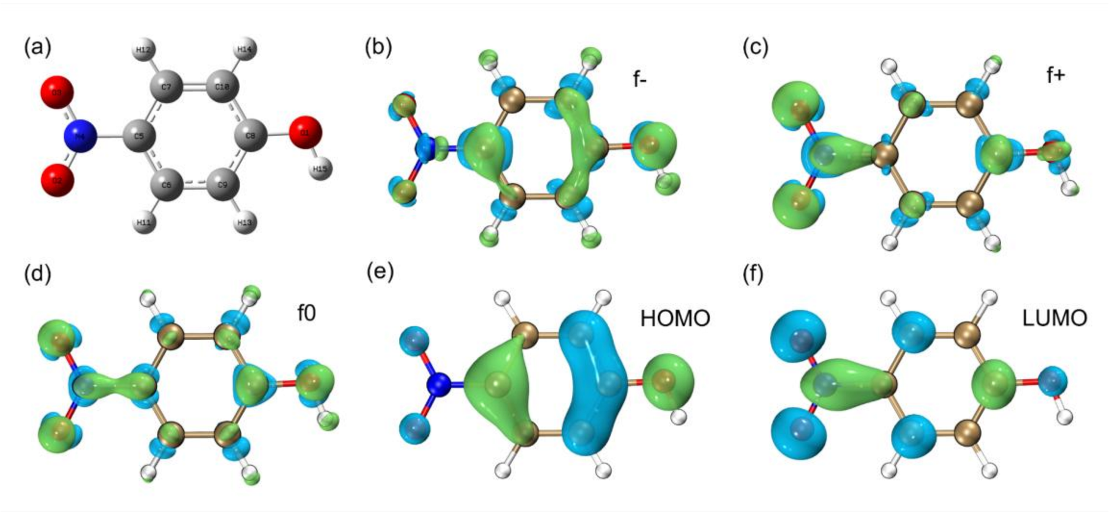

Supplementary Fig. 66. The structure and chemical properties of PNP. The (a) optimized chemical structure, (b) Fukui index (f^-) region, (c) Fukui index (f^+) region, (d) Fukui index (f^0) region, (e) HOMO region, (f) LUMO region of PNP.

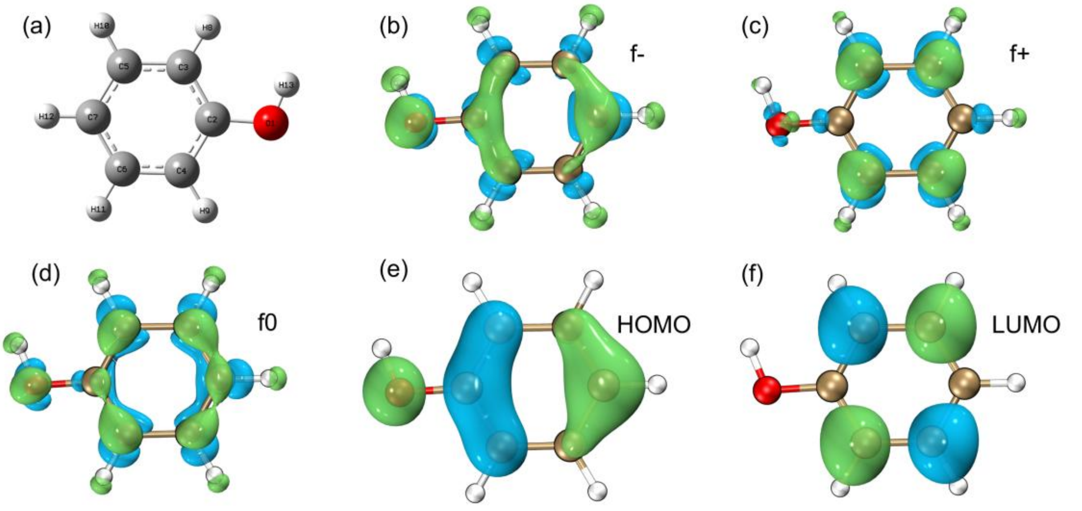

Supplementary Fig. 67. The structure and chemical properties of Phe. The (a) optimized chemical structure, (b) Fukui index (f^-) region, (c) Fukui index (f^+) region, (d) Fukui index (f^0) region, (e) HOMO region, (f) LUMO region of Phe.

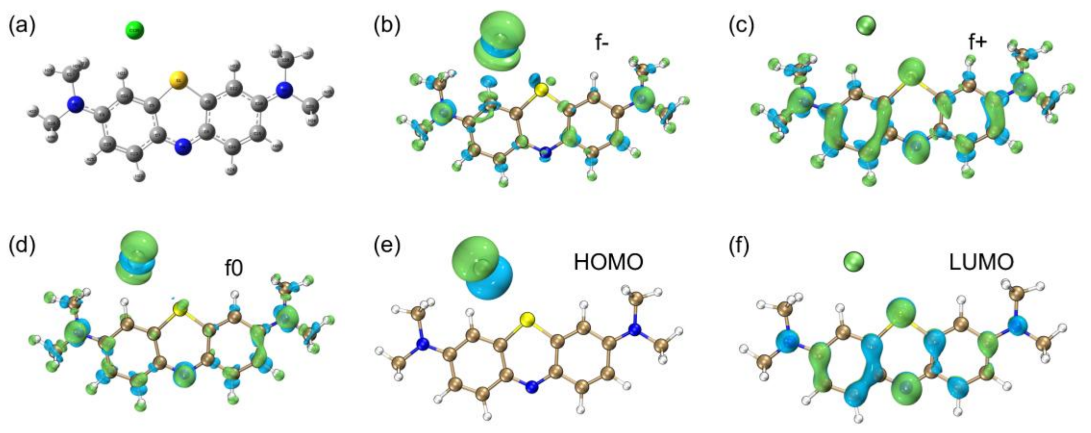

Supplementary Fig. 68. The structure and chemical properties of MB. The (a) optimized chemical structure, (b) Fukui index (f^-) region, (c) Fukui index (f^+) region, (d) Fukui index (f^0) region, (e) HOMO region, (f) LUMO region of MB.

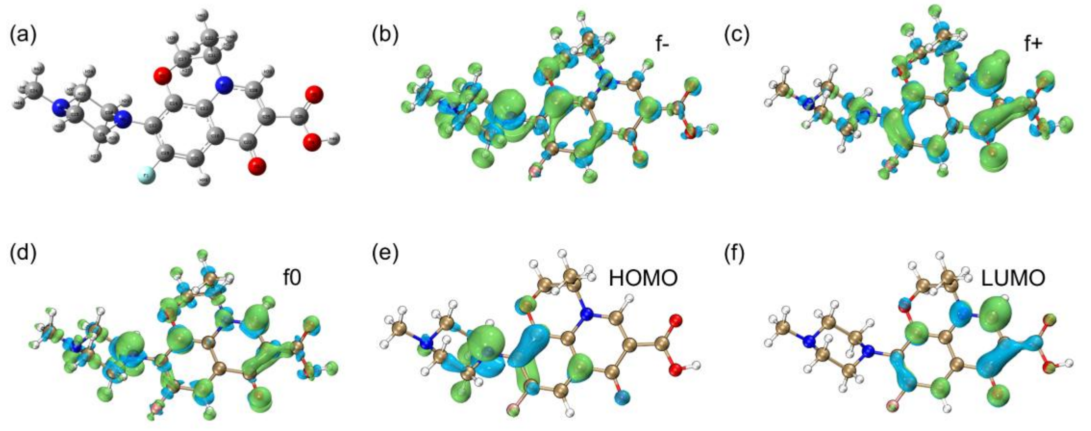

Supplementary Fig. 69. The structure and chemical properties of OFX. The (a) optimized chemical structure, (b) Fukui index (f^-) region, (c) Fukui index (f^+) region, (d) Fukui index (f^0) region, (e) HOMO region, (f) LUMO region of OFX.

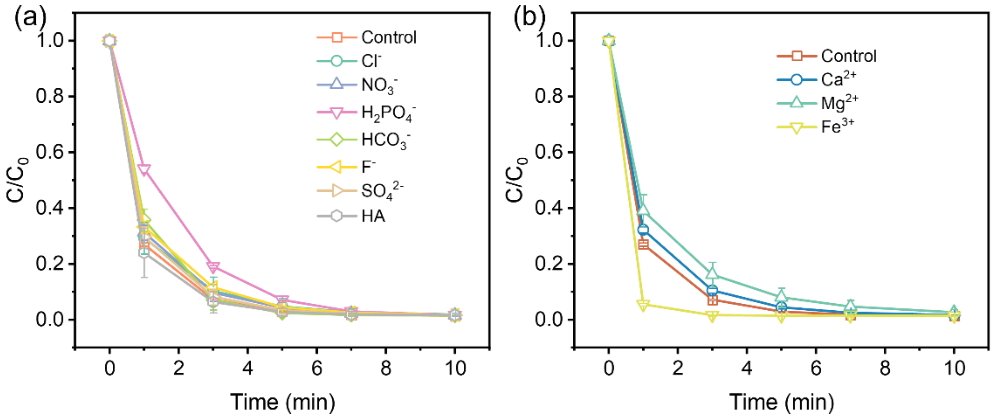

Supplementary Fig. 70. The effect of coexisting ions and humus. (a) The degradation curves of BPA under different coexisting anion and humus, (b) The degradation curves of BPA under different coexisting cations. Experiment conditions: [catalyst]= 0.05 g/L, [PMS]= 0.5 mM, [BPA]= 10 mg/L, pH= 6.00, [ion]= 10 mM, [humus]= 10 mg/L room temperature if not otherwise specified. The error bars are standard deviation of three replicate tests (n= 3).

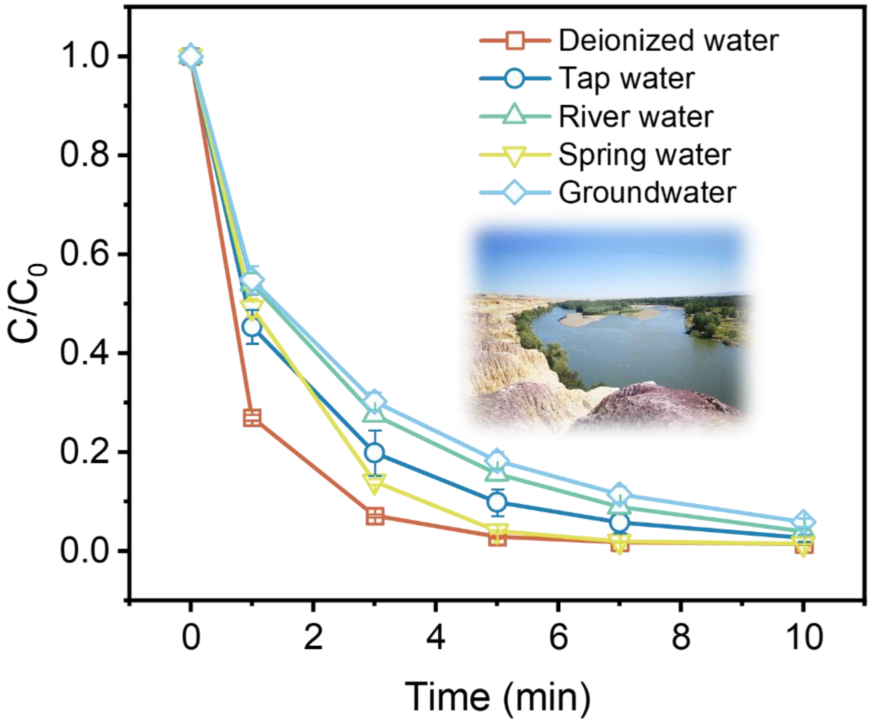

Supplementary Fig. 71. The effect of water matrix. The degradation of BPA under different real water matrices. Experiment conditions: [catalyst]= 0.05 g/L, [PMS]= 0.5 mM, [BPA]= 10 mg/L, pH= 6.00, room temperature if not otherwise specified. The error bars are standard deviation of three replicate tests (n= 3).

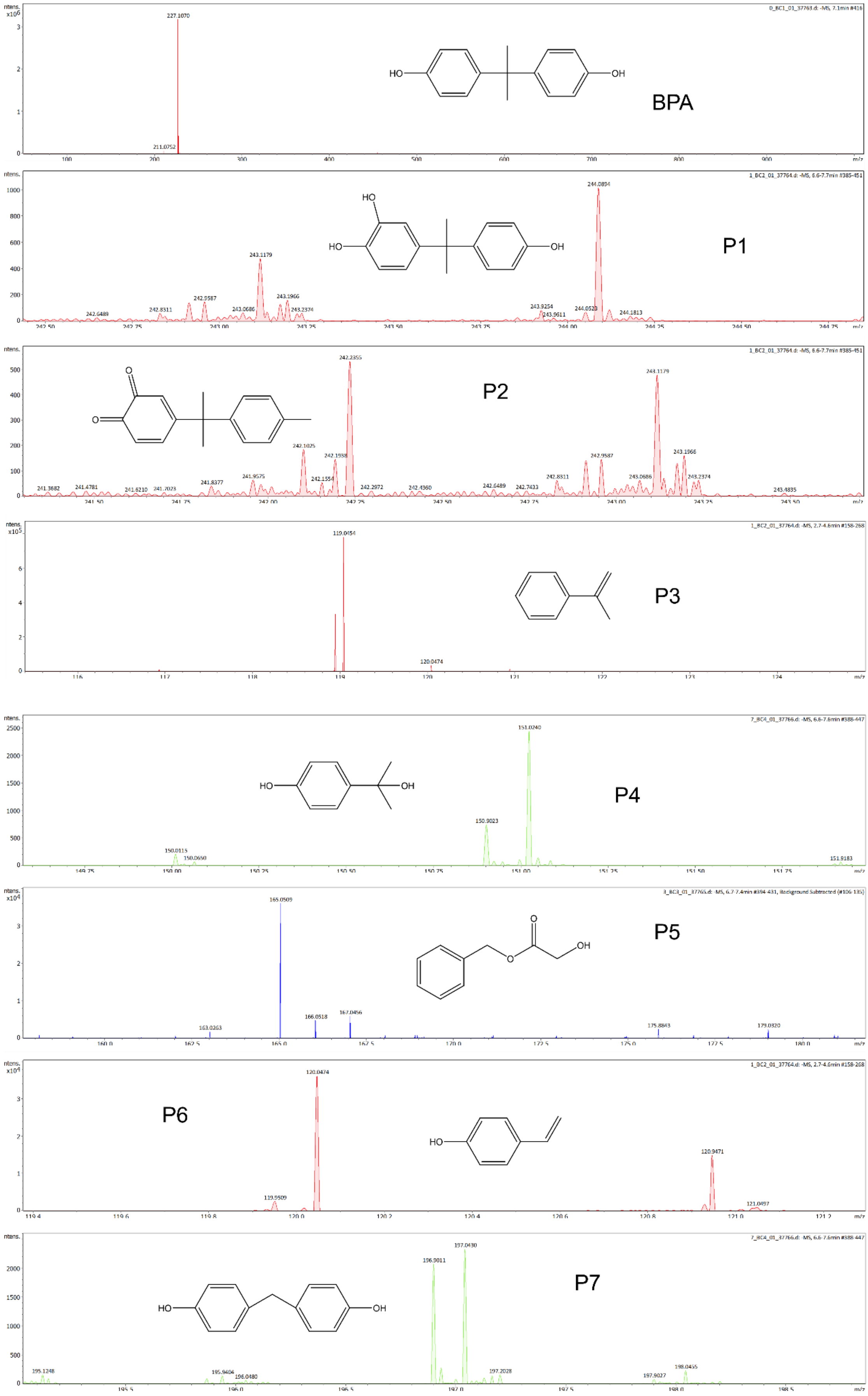
BPA
P1
P2
P3
P4
P5
P6
P7

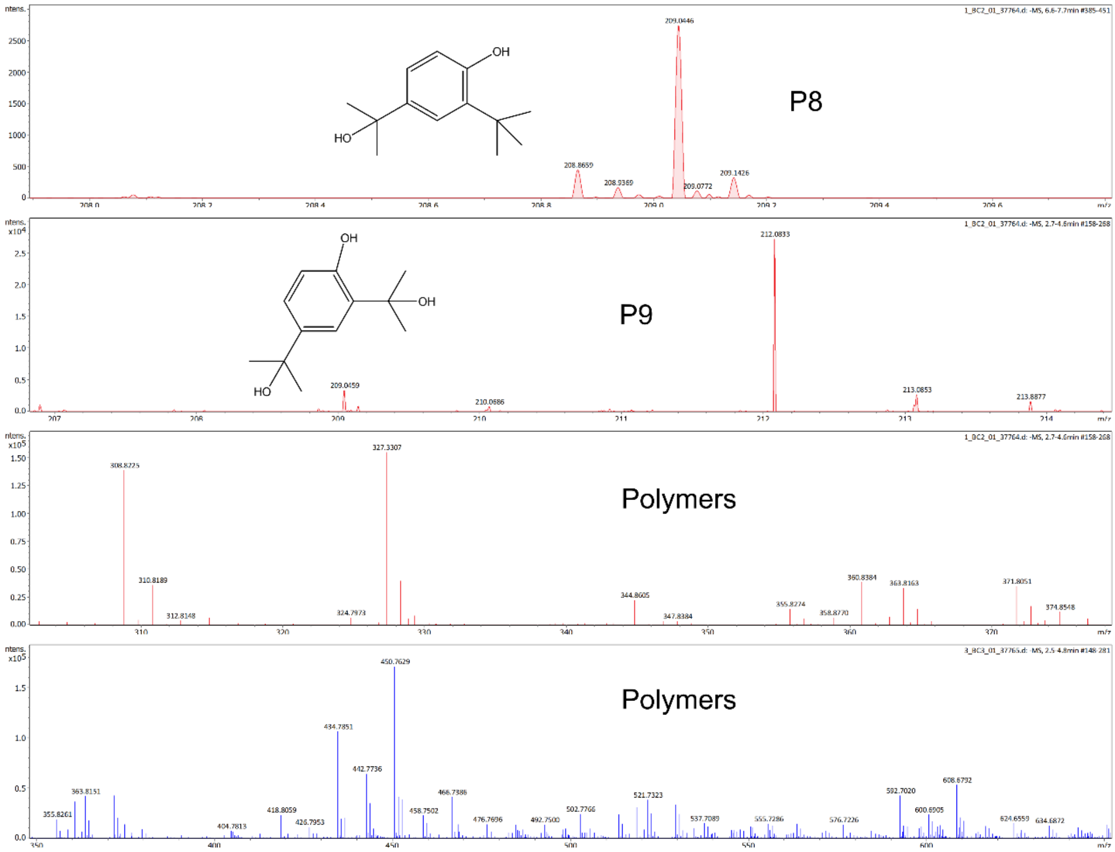

Supplementary Fig. 72. The results of UPLC-QTOF-MS. The detected degradation intermediate products of BPA.

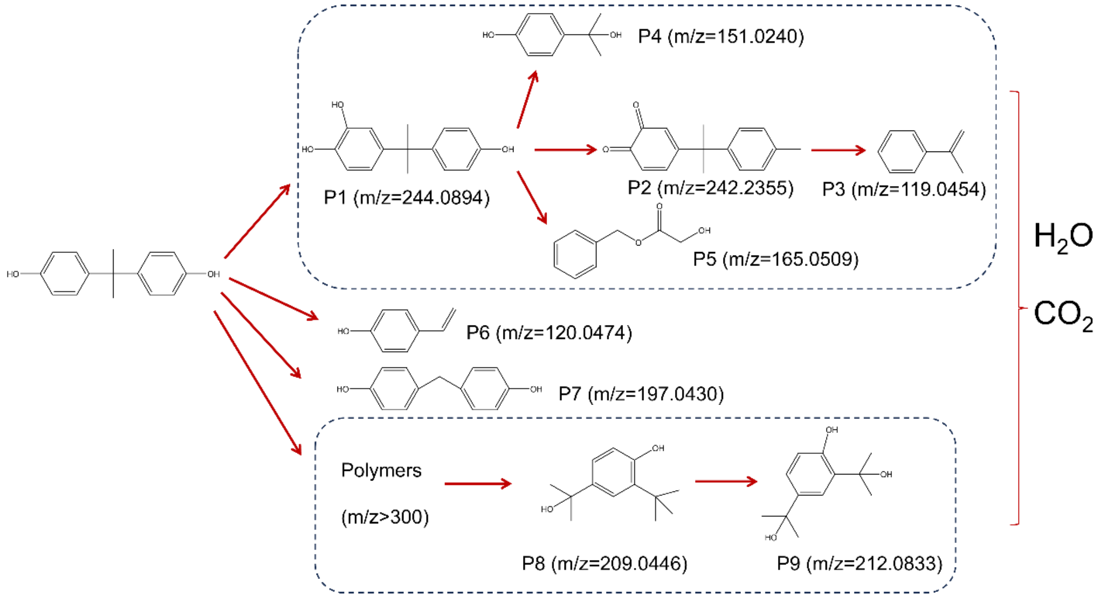

Supplementary Fig. 73. The possible degradation pathway of BPA. The possible degradation pathway and intermediate products of BPA in CoN_3C/rGO/PMS system.

In the ETP mechanism, as a substance to accept electrons, the CoN_3C/rGO-PMS* is more prone to undergo electrophilic attack with BPA. Therefore, the Fukui index was used to predict reaction sites (Supplementary Table 11). The most reactive sites, with the highest Fukui values, were found to be C4 ($f^- = 0.088$), C5 ($f^- = 0.089$), C1 ($f^- = 0.062$), C2($f^- = 0.062$) on the benzene ring, as well as O16($f^- = 0.077$) and O17($f^- = 0.077$) on the hydroxyl group, which are more possibly to react with CoN_3C/rGO-PMS*. Combining with UPLC-QTOF-MS results (Supplementary Fig. 72), four possible degradation pathways were obtained (Supplementary Fig. 73): (1) The CoN_3C/rGO-PMS* attacks sites on benzene rings to form hydroxyl addition BPA (P1), then carbonylation or the cleavage of the benzene ring and further oxidation occurs to generate P2-P5[20-22]. (2) The aromatic ring is directly broken to generate P6[23]. (3) The reactive species might attack the BPA molecule to produce P7 through the demethylation process[24]. (4) Some intermediate products will combine and form polymers through hydrogen atom transfer processes. Subsequently, these combined products will be attacked by the CoN_3C/rGO-PMS* and decompose into small molecules (P8, P9)[20]. Finally, these by-products would be minimized to H_2O and CO_2.

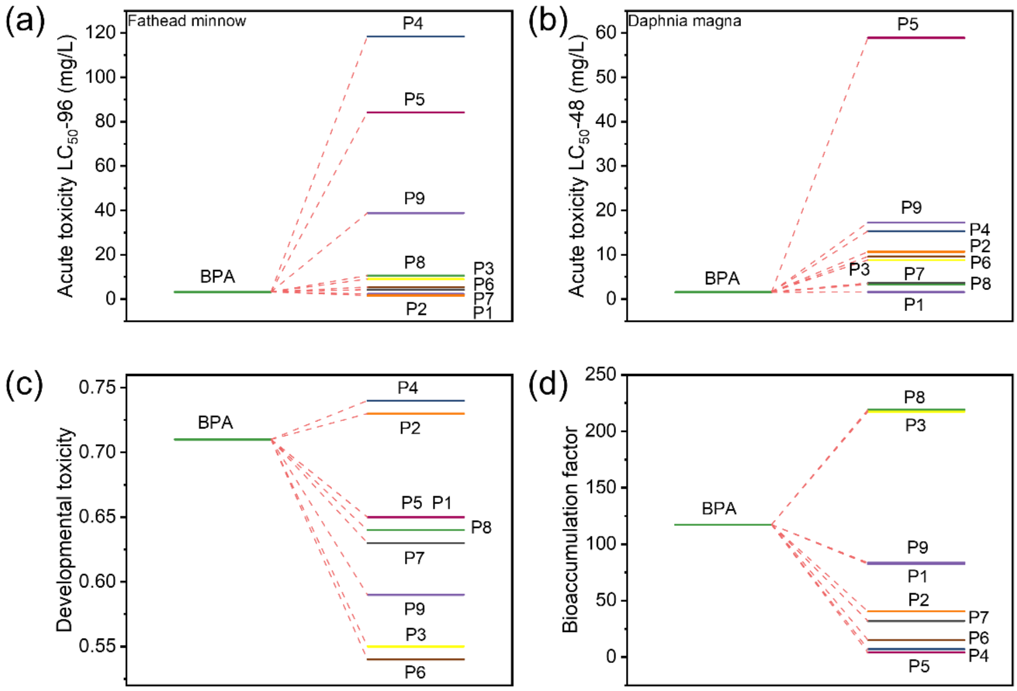

Supplementary Fig. 74. The predicted toxicity of BPA and its degradation intermediate products. (a) The acute toxicity (LC_{50}-96 for fathead minnow) of BPA and its degradation products. (b) The acute toxicity (LC_{50}-48 for Daphnia magna) of BPA and its degradation products. (c) The developmental toxicity of BPA and its degradation products. (d) The bioaccumulation factor of BPA and its degradation products.

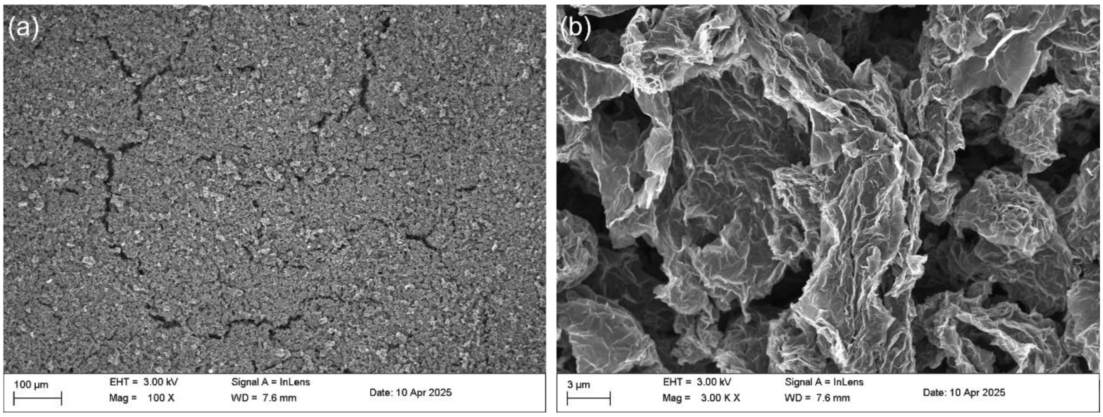

Supplementary Fig. 75. The microstructure of CoN_3C/rGO/PVDF membrane. The SEM images of CoN_3C/rGO/PVDF membrane under different magnifications (Mag). (a) Mag= 100×, (b) Mag= 3000×.

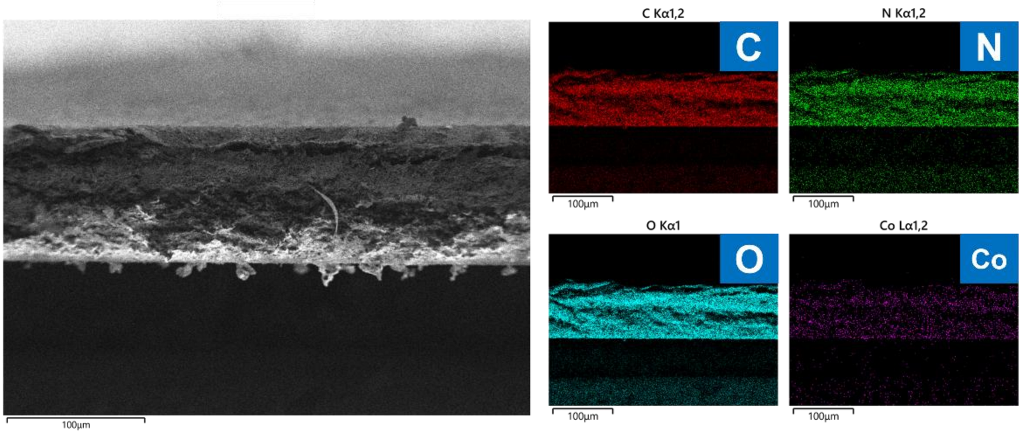

Supplementary Fig. 76. The elements distribution of CoN_3C/rGO/PVDF membrane. The SEM-EDS mapping of C, N, O, and Co on CoN_3C/rGO/PVDF.

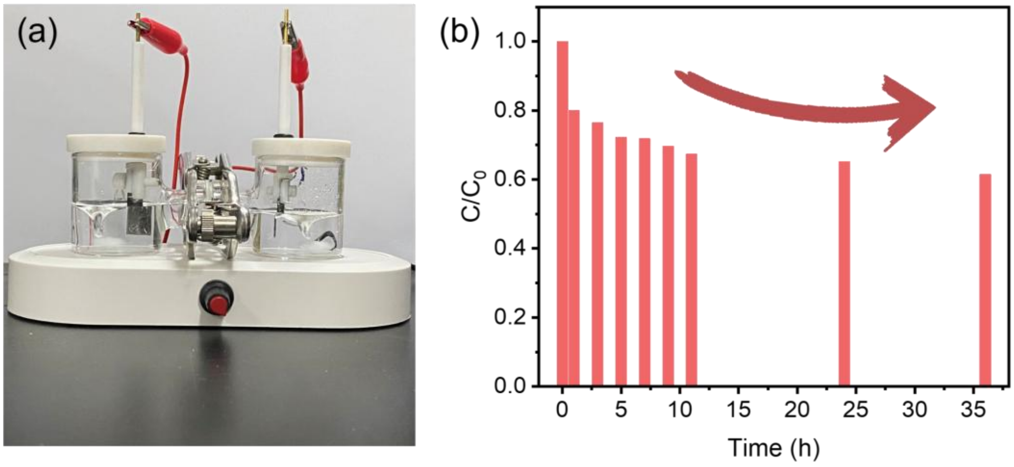

Supplementary Fig. 77. The performance of proof-of-concept dual-chamber device. (a) The image of dual-chamber device. (b) Degradation effect of BPA in dual-chamber device.

Supplementary Tables

Supplementary Table 1. HPLC conditions for organic compounds analysis

Organics	Water/MeOH	Water/MeCN	Wavelength (nm)	Flow rate (ml/min)
BPA	30/70		230	0.5
PNP	40/60		320	1
PMSO		80/20	230	0.7
$PMSO_2$		80/20	215	0.7
MB			665	
Phe		30/70	271	0.5
OFX		80(0.1% formic acid)/20	294	0.8
4-BP		30/70	230	0.8
NB		30/70	265	1
BA		50(0.1% formic acid)/50	228	1
FFA	90(0.1% phosphoric acid)/10		220	1
p-CBA	45(0.1% formic acid)/55		239	1

Supplementary Table 2. The second-order reaction rate between the probe molecule and various ROS.

Probe compounds	$k_{\cdot OH}$	$k_{SO_4^{\cdot-}}$	$k_{O_2^{\cdot-}}$	$k_{^1O_2}$	$k_{Co(IV)=O}$	Ref
Benzoic acid (BA)	1.2×10^{9}	5.9×10^{9}	/	/	/	25
Nitrobenzene (NB)	3.9×10^{9}	$<10^{9}$	/	/	/	25
Furfuryl alcohol (FFA)	1.5×10^{10}	1.3×10^{10}	3.5×10^{3}	1.2×10^{8}	/	25
p-chlorobenzoic-acid (pCBA)	1.5×10^{10}	1.3×10^{9}	8.6×10^{7}	1.4×10^{7}	/	25
Methyl phenyl sulfoxide (PMSO)	3.61×10^{9}	3.17×10^{8}	/	/	2×10^{6}	25

Supplementary Table 3. The elements content (%) of catalysts from XPS.

Elements / Catalysts	Co	N	C	O
CoN_3C/rGO	0.4	19.1	71.1	9.4
CoN_3C	0.4	31.0	61.3	7.3
rGO	/	/	88.0	12.0
Used CoN_3C/rGO	0.3	14.1	79.3	6.3

Supplementary Table 4. The elements content of CoN_3C/rGO from SEM-EDS mapping.

Elements	wt %
Co	0.3
N	15.0
C	75.9
O	8.8

Supplementary Table 5. The elements content of CoN_3C from SEM-EDS mapping.

Elements	wt %
Co	1.2
N	37.3
C	61.5

Supplementary Table 6. The elements content of rGO from SEM-EDS mapping.

Elements	wt %
N	0.0
C	87.0
O	13.0

Supplementary Table 7. The exact Co-loading content of catalysts.

Catalysts	CoN_3C/rGO	CoN_3C	Used CoN_3C/rGO
Co content (%)	1.25	1.97	0.80

Supplementary Table 8. EXAFS fitting parameters at the Co K-edge for samples.

Samples	Path	CN	R(Å)	σ^2(Å^2)	ΔE_0(eV)	R factor
Co foil	Co-Co	12	2.49	0.00882	7.273	0.0193
Co_3O_4	Co-O	3.4(0.32)	1.92	0.00300	0.040	0.0174
	Co-Co	6.1(1.52)	2.88	0.00781	-0.046	
CoPc	Co-N	1.9(0.21)	1.87	0.00200	-0.104	0.0199
CoN_3C/rGO	Co-N	2.9(0.20)	1.92	0.00700	-0.062	0.0116
CoN_3C	Co-N	2.7(0.19)	1.92	0.00700	-0.061	0.0139
Used CoN_3C/rGO	Co-N	2.7(0.26)	1.93	0.00300	-0.04671	0.0145
	Co-O	1.4(0.18)	2.10	0.00063	0.05788	

CN is the coordination number; R is the interatomic distance (the bond length between central atoms and surrounding coordination atoms); σ^2 is the Debye-Waller factor (a measure of thermal and static disorder in absorber-scatter distances); ΔE_0 is the edge-energy shift (the difference between the zero kinetic energy value of the sample and that of the theoretical model). R factor is used to evaluate the goodness of the fit. Error bounds (accuracies) that characterize the structural parameters obtained by EXAFS spectroscopy were estimated as CN ± 20%; R ± 2%; σ^2 ± 20%; ΔE_0 ± 20%; R factor ≤ 0.02.

Supplementary Table 9. The normalized k_{obs} of BPA degradation in some reported catalysts mediated PMS activation systems.

Catalyst	Catalyst dosage ($g\cdot L^{-1}$)	PMS concentration (mM)	BPA initial concentration ($mg\cdot L^{-1}$)	Normalized k ($min^{-1}\cdot g^{-1}\cdot L$)	Ref
NCM-0.6	0.08	0.15	20	1.56	26
Fe_{SA}-N-C	0.15	1.3	20	1.6	27
NOPC	0.2	0.15	10	1.5	28
SA-CoNC	0.1	0.5	10	0.3	29
FeOOH/Mt-TC-C	0.05	1.0	20	2.4	30
Fe_{SA}-N/C-20	0.15	1.3	20	1.78	31
NPC-1000	0.5	1.0	10	0.24	32
$CoFe_2O_4$	0.1	0.45	10	0.9	33
SA-FeN/C	0.1	0.5	10	6.2	34
Co(OH)F@MXenes	0.1	0.3	20	0.8	35
CuZn-NC	0.1	1.3	25	13.8	36
c-Cu_2O	0.2	1.0	10	0.14	37
Fe/O co-doped g-C_3N_4	0.1	1.0	10	12.0	38
Fe-N_2O_1/OCN	0.1	0.2	42.9	8.0	39
^{HT}NBC	0.2	1.0	22.8	3.1	40
Fe_{SA}-BNC	0.1	0.2	0.46	15.9	41
$La_{0.65}Sr_{0.35}MnO_3/Mn_3O_4$	0.1	3.3	10	1.7	42
Fe-N/O@C	0.1	1.3	10	1.09	43
Cu-NC-4	0.2	1.0	20	4.99	44
CoN_3C/rGO	0.05	0.5	10	25	This work
CoN_3C	0.05	0.5	10	1.58	This work

Supplementary Table 10. Relative parameters of different pollutants.

Pollutants	Electrophilicity index (eV)	Nucleophilicity index (eV)	Hardness (eV)	Softness (eV^{-1})	HOMO (eV)	LUMO (eV)
BPA	0.46	3.52	8.68	0.13	-5.97	-0.57
Phe	0.63	3.16	10.44	0.10	-6.36	-0.53
PNP	3.47	0.45	3.39	0.29	-7.21	-2.54
4BP	0.59	2.61	9.53	0.11	-5.60	-1.21
OFX	2.64	0.80	10.17	0.10	-5.78	-1.55
MB	0.88	2.05	3.67	0.27	-6.06	-3.64

Supplementary Table 11. Natural population analysis (NPA) charge group and condensed Fukui index for electrophilic attack (f^-) at B3LYP/6–31G(d) level.

ATOM	No	Charge(0) (e/Å^3)	Charge(-1) (e/Å^3)	Charge(+1) (e/Å^3)	f$^-$
C	1	0.321	0.319	0.383	0.062
C	2	0.321	0.319	0.383	0.062
C	3	-0.07	-0.063	-0.089	-0.019
C	4	-0.061	-0.046	0.027	0.088
C	5	-0.062	-0.046	0.027	0.089
C	6	-0.667	-0.657	-0.673	-0.006
C	7	-0.667	-0.657	-0.673	-0.006
C	8	-0.205	-0.283	-0.183	0.022
C	9	-0.205	-0.283	-0.183	0.022
C	10	-0.219	-0.3	-0.211	0.008
C	11	-0.219	-0.3	-0.211	0.008
C	12	-0.308	-0.392	-0.285	0.023
C	13	-0.308	-0.392	-0.285	0.023
C	14	-0.28	-0.366	-0.221	0.059
C	15	-0.28	-0.367	-0.221	0.059
O	16	-0.688	-0.711	-0.611	0.077
O	17	-0.688	-0.711	-0.611	0.077

Supplementary Table 12. Natural population analysis (NPA) charge group and condensed Fukui index for electrophilic attack (f^-) at B3LYP/6–31G(d) level of 4-BP.

ATOM	No	Charge(0) (e/Å^3)	Charge(-1) (e/Å^3)	Charge(+1) (e/Å^3)	f^-
Br	1	-0.054	-0.1793	0.2126	0.2666
O	2	-0.1841	-0.2262	-0.0682	0.116
C	3	0.0759	0.0273	0.1609	0.085
C	4	-0.0493	-0.1729	0.0222	0.0716
C	5	-0.0628	-0.1809	0.0052	0.068
C	6	-0.0383	-0.1544	0.0153	0.0536
C	7	-0.0409	-0.1611	0.0189	0.0598
C	8	-0.011	-0.0482	0.0717	0.0827
H	9	0.0505	-0.0116	0.0914	0.041
H	10	0.0418	-0.0185	0.0821	0.0403
H	11	0.0483	-0.0096	0.084	0.0357
H	12	0.0477	-0.0117	0.0845	0.0368
H	13	0.1763	0.1469	0.2193	0.043

Supplementary Table 13. Natural population analysis (NPA) charge group and condensed Fukui index for electrophilic attack (f^-) at B3LYP/6–31G(d) level of PNP.

**ATOM**	**No**	**Charge(0) (e/Å^3)**	**Charge(-1) (e/Å^3)**	**Charge(+1) (e/Å^3)**	**f^-**
O	1	-0.1693	-0.2279	-0.0304	0.1388
O	2	-0.2118	-0.3813	-0.1354	0.0764
O	3	-0.2101	-0.3803	-0.1364	0.0737
N	4	0.2458	0.1364	0.263	0.0171
C	5	0.0101	-0.0243	0.1248	0.1148
C	6	-0.0238	-0.0833	0.0347	0.0584
C	7	-0.0222	-0.078	0.0292	0.0514
C	8	0.0927	0.0143	0.1822	0.0895
C	9	-0.0585	-0.1047	0.0208	0.0792
C	10	-0.0452	-0.0933	0.0447	0.0898
H	11	0.0541	0.0201	0.091	0.0369
H	12	0.0545	0.0215	0.0905	0.0359
H	13	0.0459	0.0101	0.0895	0.0435
H	14	-0.2279	-0.0304	0.0998	0
H	15	-0.3813	-0.1354	0.1388	0.0586

Supplementary Table 14. Natural population analysis (NPA) charge group and condensed Fukui index for electrophilic attack (f^-) at B3LYP/6–31G(d) level of Phe.

ATOM	No	Charge(0) (e/Å^3)	Charge(-1) (e/Å^3)	Charge(+1) (e/Å^3)	f^-
O	1	-0.1889	-0.2338	-0.0456	0.1434
C	2	0.0733	0.0209	0.1729	0.0996
C	3	-0.071	-0.1916	0.0112	0.0822
C	4	-0.0579	-0.1833	0.0335	0.0913
C	5	-0.0392	-0.1685	0.0358	0.075
C	6	-0.0367	-0.163	0.0293	0.066
C	7	-0.0567	-0.1176	0.0873	0.1439
H	8	0.0361	-0.0276	0.083	0.0469
H	9	0.0448	-0.0206	0.0932	0.0485
H	10	0.0418	-0.0255	0.089	0.0472
H	11	0.0424	-0.0237	0.088	0.0457
H	12	0.0387	-0.0077	0.0975	0.0588
H	13	0.1731	0.1418	0.2247	0.0516

Supplementary Table 15. Input materials and energy required to treat 1 ton of BPA wastewater through three different systems.

Treatment of 1 ton of BPA wastewater	CoN_3C/rGO+PMS system	CoN_3C+PMS system	Co_3O_4 +PMS system
Reagents (g)			
$Co(NO_3)_2$•$6H_2O$	20	133	799
Formamide	16950	112988.7	0
Reduced Graphene Oxide	50	0	0
peroxymonosulfate	150	300	750
NaOH	0	0	27.28
H_2O	0	0	272.8
Energy (kWh)			
Power supply	63.15	63.15	61.95
Pumps (Fenton reactor)	3.6	3.6	3.6
Stirrer (Fenton reactor)	1.3	1.3	8.0

Supplementary References

1. Li, X. et al. UV-Induced Synthesis of Graphene Supported Iridium Catalyst with Multiple Active Sites for Overall Water Splitting. *Adv. Funct. Mater.* **34**, 2313530 (2024).
2. Liu, X. et al. Defects mediate the active N sites in N-doped carbon catalyst for efficiently catalyzing H_2S to element sulfur. *Appl. Catal. B: Environ.* **380**, 125718 (2026).
3. Shen, Z. et al. Sequential Catalysis of Defected-Carbon and Solid Catalyst in Li–O_2 Batteries. *J. Phys. Chem. C* **127**, 6239-6247 (2023).
4. Omar, H. et al. A review of synthesis graphene oxide from natural carbon based coconut waste by Hummer's method. *Mater. Today: Proc* **75**, 188-192 (2023).
5. Dwivedi, N. et al. Unusual High Hardness and Load-Dependent Mechanical Characteristics of Hydrogenated Carbon–Nitrogen Hybrid Films. *ACS Appl. Mater. Interfaces* **14**, 20220-20229 (2022).
6. Zheng, X. et al. Rape Pollen-Based Composite Sorbent with Thermo-Responsive and Photothermal Properties for Atmospheric Water Harvesting. *Adv. Funct. Mater.* **34**, 2407127 (2024).
7. Ma, X. & Cheng, H. Synergy of nitrogen vacancies and intercalation of carbon species for enhancing sunlight photocatalytic hydrogen production of carbon nitride. *Appl. Catal. B: Environ.* **314**, 121497 (2022).
8. Peng, G. et al. Nitrogen-Defective Polymeric Carbon Nitride Nanolayer Enabled Efficient Electrocatalytic Nitrogen Reduction with High Faradaic Efficiency. *Nano Lett.* **20**, 2879-2885 (2020).
9. Shan, X. et al. Ion-Regulated Water Activation in Plasmonic Aerogels for Highly Efficient and Salt-Resistive Solar Desalination. *Adv. Funct. Mater.*, e14279 (2025).
10. Chen, D. et al. Photosynergetic Electrochemical Synthesis of Graphene Oxide. *J. Am. Chem. Soc.* **142**, 6516-6520 (2020).
11. Gim, G. et al. Low-temperature hydrogenation of nanodiamond as a strategy to fabricate sp-hybridized nanocarbon as a high-performance persulfate activator. *Appl.*

Catal. B: Environ. **316**, 121589 (2022).

12. Shan, T. et al. Boosting H_2O_2 production over carboxymethyl cellulose modified g-C_3N_4 via hydrogen-bonding-assisted charge transfer. *Chem. Eng. J.* **478**, 147509 (2023).
13. Yang, C. et al. Potassium and chlorine co-tuned graphitic carbon nitride for organic pollutants photodegradation: Revealing the effects of cyano groups on O_2 evolution. *J. Catal.* **431**, 115396 (2024).
14. Kang, T.W. et al. An Ion-Channel-Restructured Zwitterionic Covalent Organic Framework Solid Electrolyte for All-Solid-State Lithium-Metal Batteries. *Adv. Mater.* **35**, 2301308 (2023).
15. Nguyen, K.G. et al. Engineering Nitrogen-Doped Carbon Quantum Dots: Tailoring Optical and Chemical Properties through Selection of Nitrogen Precursors. *Small* **20**, 2310587 (2024).
16. Ruan, L. et al. Rhodium nanoparticles in ZrO_2 on N-doped carbon leads to ultra-high catalytic selectivity and activity in nitroarene hydrogenation. *Appl. Catal. B: Environ.* **379** (2025).
17. Lei, G. et al. Highly Poison-Resistant Single-Atom Co–N_4 Active Sites with Superior Operational Stability over 460 h for H_2S Catalytic Oxidation. *Small* **17**, 2104939 (2021).
18. Kaulbersch, J., McGuigan, S., Timm, J., Maggard, P. & Marschall, R. Photocatalytic Activity and Stability of Carbon Nitride-Pyrite Composites. *ChemPhotoChem* **9**, e202400343 (2025).
19. Qin, F. et al. Catalyst-Free Photochemical Activation of Peroxymonosulfate in Xanthene-Rich Systems for Fenton-Like Synergistic Decontamination: Efficacy of Proton Transfer Process. *Angew. Chem. Int. Ed.* **62**, e202300256 (2023).
20. Dai, J. et al. Applying a novel advanced oxidation process of biochar activated periodate for the efficient degradation of bisphenol A: Two nonradical pathways. *Chem. Eng. J.* **453**, 139889 (2023).
21. Xu, M. et al. Chemical etching to regulation oxygen vacancies on Mn-Fe PBA for

highly efficient degradation of bisphenol A and acetaminophen. *J. Clean. Prod.* **377**, 134258 (2022).

22. Chen, Y. et al. WS_2-cocatalyzed peroxymonosulfate activation via an enhanced Fe(III)/Fe(II) cycle toward efficient organic pollutant degradation. *Chem. Eng. J.* **442**, 135961 (2022).
23. Jiang, X.-H. et al. Photodegradation of Organic Pollutants Coupled with Simultaneous Photocatalytic Evolution of Hydrogen Using Quantum-Dot-Modified g-C_3N_4 Catalysts under Visible-Light Irradiation. *ACS Sustainable Chem. Eng.* **6**, 12695-12705 (2018).
24. Zhang, X. et al. Insight to unprecedented catalytic activity of double-nitrogen defective metal-free catalyst: Key role of coal gangue. *Appl. Catal. B: Environ.* **263**, 118316 (2020).
25. Wu, Q.-Y., Yang, Z.-W., Wang, Z.-W. & Wang, W.-L. Oxygen doping of cobalt-single-atom coordination enhances peroxymonosulfate activation and high-valent cobalt–oxo species formation. *Proc. Natl. Acad. Sci. U. S. A.* **120**, e2219923120 (2023).
26. Chen, H. et al. Nitrogen-doped biochar/MnO_2 as an efficient PMS activator for synergistic BPA degradation via non-free radical pathways in the water. *J. Environ. Chem. Eng* **12**, 112446 (2024).
27. Li, Y. et al. Uniform N-coordinated single-atomic iron sites dispersed in porous carbon framework to activate PMS for efficient BPA degradation via high-valent iron-oxo species. *Chem. Eng. J.* **389**, 124382 (2020).
28. He, Y.-L. et al. Activating peroxymonosulfate by N and O co-doped porous carbon for efficient BPA degradation: A re-visit to the removal mechanism and the effects of surface unpaired electrons. *Appl. Catal. B: Environ.* **314**, 121390 (2022).
29. Wang, Q. et al. Degradation of bisphenol a using peroxymonosulfate activated by single-atomic cobalt catalysts: Different reactive species at acidic and alkaline pH. *Chem. Eng. J.* **439**, 135002 (2022).
30. Yang, S. et al. Efficient peroxymonosulfate activation and bisphenol A degradation

derived from mineral-carbon materials: Key role of double mineral-templates. *Appl. Catal. B: Environ.* **267**, 118701 (2020).

31. Yang, T., Fan, S., Li, Y. & Zhou, Q. Fe-N/C single-atom catalysts with high density of Fe-Nx sites toward peroxymonosulfate activation for high-efficient oxidation of bisphenol A: Electron-transfer mechanism. *Chem. Eng. J.* **419**, 129590 (2021).
32. Qu, G. et al. Synergistic activation of peroxymonosulfate by intrinsic defect and graphitic N of N, P co-doped carbon microspheres for BPA degradation. *Chem. Eng. J.* **475**, 145888 (2023).
33. Long, X. et al. Oxygen vacancies-enriched $CoFe_2O_4$ for peroxymonosulfate activation: The reactivity between radical-nonradical coupling way and bisphenol A. *J. Hazard. Mater.* **418**, 126357 (2021).
34. Ma, J. et al. Highly Efficient Degradation of Bisphenol A by Peroxymonosulfate Activation Using Bamboo Kraft Lignin Single-Atom Catalyst. *Small* **21**, 2409803 (2025).
35. Wang, F. et al. Facile fabricate of novel Co(OH)F@MXenes catalysts and their catalytic activity on bisphenol A by peroxymonosulfate activation: The reaction kinetics and mechanism. *Appl. Catal. B: Environ.* **262**, 118099 (2020).
36. Gan, D. et al. Atomically dispersed copper-zinc dual sites anchored on nitrogen-doped porous carbon toward peroxymonosulfate activation for degradation of various organic contaminants. *J. Colloid Interface Sci.* **673**, 756-764 (2024).
37. Li, H. et al. Structure-dependent catalysis of cuprous oxides in peroxymonosulfate activation via nonradical pathway with a high oxidation capacity. *J. Hazard. Mater.* **385** (2020).
38. Chen, F. et al. Efficient decontamination of organic pollutants under high salinity conditions by a nonradical peroxymonosulfate activation system. *Water Res.* **191**, 116799 (2021).
39. Deng, Y. et al. Highly effective activation of peroxymonosulfate via oxygen-coordinated single-atom iron for water decontamination. *Chem. Eng. J.* **485**, 149782 (2024).

40. Byambaa, B. et al. Synthesis of N-doped sludge biochar using the hydrothermal route-enabled carbonization method for the efficient degradation of organic pollutants by peroxymonosulfate activation. *Chem. Eng. J.* **456**, 141037 (2023).
41. Deng, Y., Zhou, Y., Song, Z. & Yang, X. Long-Range Electronically Polarized Fe-N_5 Catalysts Redirect Polymerization-Driven Phenolic Pollutant Removal Toward Sustainable Carbon Sequestration. *Angew. Chem. Int. Ed.*, e202509493 (2025).
42. Liang, P. et al. Improved catalytic activity in peroxymonosulfate activation over manganese-based perovskite oxides with acid treatment. *Chem. Eng. Sci.* **293**, 120058 (2024).
43. Su, Y., Wang, Y., Wan, J., Zuo, S. & Lin, Y. Mechanism of directed activation of peroxymonosulfate by Fe-N/O unsymmetrical coordination-modulated polarized electric field. *J. Colloid Interface Sci.* **664**, 779-789 (2024).
44. Chen, X. et al. Exploring the mechanism of electron transfer-mediated peroxymonosulfate activation over Cu-based catalyst for the selective decomposition of bisphenol A. *Chem. Eng. J.* **471**, 144774 (2023).
