# Supplementary material for: Breaking the activity-selectivity trade-off in Fenton-like catalysis by d-orbital modulation of single-atom sites within a nano-island-like structure
Source: Nat Commun. 2026 Jun 8;17:7293. doi: 10.1038/s41467-026-74072-2 (PMC13402592; doi:10.1038/s41467-026-74072-2)
Supplement: Supplementary file 2 — Description of Additional Supplementary Files [file 41467_2026_74072_MOESM2_ESM.pdf]

Description of Additional Supplementary Files

Supplementary Data 1: Natural population analysis (NPA) charge group and condensed Fukui index for electrophilic attack (f^{-}) at B3LYP/6–31G(d) level of MB.

Supplementary Data 2: Natural population analysis (NPA) charge group and condensed Fukui index for electrophilic attack (f^{-}) at B3LYP/6–31G(d) level of OFX.

Supplementary Data 3: Impact assessment for CoN_3C/rGO+PMS system.

Supplementary Data 4: Impact assessment for CoN_3C +PMS system.

Supplementary Data 5: Impact assessment for Co_3O_4 +PMS system.

Supplementary Data 6: Atomic coordinates of the optimized configurations of CoN_3C.

Supplementary Data 7: Atomic coordinates of the optimized configurations of CoN_3C/rGO.

Supplementary Data 8: Atomic coordinates of the optimized configurations of CoN_3C-PMS.

Supplementary Data 9: Atomic coordinates of the optimized configurations of CoN_3C/rGO-PMS.
