# Supplementary material for: Breaking the activity-selectivity trade-off in Fenton-like catalysis by d-orbital modulation of single-atom sites within a nano-island-like structure
Source: Nat Commun. 2026 Jun 8;17:7293. doi: 10.1038/s41467-026-74072-2 (PMC13402592; doi:10.1038/s41467-026-74072-2)
Supplement: Supplementary file 3 — Supplementary Data 1 [file 41467_2026_74072_MOESM3_ESM.docx]

**Supplementary Data 1.** Natural population analysis (NPA) charge group and condensed Fukui index for electrophilic attack (f ^–^) at B3LYP/6–31G(d) level of MB.

| **ATOM** | **No** | **Charge(0)**  **(e/Å^3^)** | **Charge(-1)**  **(e/Å^3^)** | **Charge(+1)**  **(e/Å^3^)** | **f^-^** |
| --- | --- | --- | --- | --- | --- |
| S | 1 | 0.1753 | 0.0833 | 0.1824 | 0.0071 |
| N | 2 | -0.0012 | -0.0407 | 0.0359 | 0.0371 |
| N | 3 | -0.0208 | -0.0503 | 0.0173 | 0.0381 |
| N | 4 | -0.1179 | -0.1958 | -0.0953 | 0.0226 |
| C | 5 | 0.0014 | -0.0219 | 0.0074 | 0.0061 |
| C | 6 | -0.0061 | -0.0302 | 0.0057 | 0.0117 |
| C | 7 | 0.0411 | 0.0146 | 0.0604 | 0.0192 |
| C | 8 | 0.0289 | 0.0116 | 0.0581 | 0.0292 |
| C | 9 | 0.0761 | 0.0377 | 0.0879 | 0.0118 |
| C | 10 | 0.0642 | 0.0276 | 0.083 | 0.0188 |
| C | 11 | -0.0696 | -0.0854 | -0.0526 | 0.017 |
| C | 12 | -0.0747 | -0.0911 | -0.0581 | 0.0166 |
| C | 13 | -0.0229 | -0.0642 | -0.0023 | 0.0206 |
| C | 14 | 0.0833 | 0.1824 | -0.0038 | 0.0575 |
| C | 15 | -0.0407 | 0.0359 | 0.0071 | 0.092 |
| C | 16 | -0.0503 | 0.0173 | 0.0371 | 0.0395 |
| C | 17 | -0.1958 | -0.0953 | 0.0381 | 0.0295 |
| C | 18 | -0.0219 | 0.0074 | 0.0226 | 0.0779 |
| C | 19 | -0.0302 | 0.0057 | 0.0061 | 0.0232 |
| C | 20 | 0.0146 | 0.0604 | 0.0117 | 0.0241 |
| H | 21 | 0.0116 | 0.0581 | 0.0192 | 0.0265 |
| H | 22 | 0.0377 | 0.0879 | 0.0292 | 0.0173 |
| H | 23 | 0.0276 | 0.083 | 0.0118 | 0.0385 |
| H | 24 | -0.0854 | -0.0526 | 0.0188 | 0.0365 |
| H | 25 | -0.0911 | -0.0581 | 0.017 | 0.0158 |
| H | 26 | -0.0642 | -0.0023 | 0.0166 | 0.0164 |
| H | 27 | 0.1824 | -0.0038 | 0.0206 | 0.0413 |
| H | 28 | 0.0359 | 0.0071 | 0.0575 | 0 |
| H | 29 | 0.0173 | 0.0371 | 0.092 | 0.0495 |
| H | 30 | -0.0953 | 0.0381 | 0.0395 | 0.0383 |
| H | 31 | 0.0074 | 0.0226 | 0.0295 | 0.0338 |
| H | 32 | 0.0057 | 0.0061 | 0.0779 | 0.0503 |
| H | 33 | 0.0604 | 0.0117 | 0.0232 | 0.0147 |
| H | 34 | 0.0581 | 0.0192 | 0.0241 | 0.0179 |
| H | 35 | 0.0879 | 0.0292 | 0.0265 | 0.0229 |
| H | 36 | 0.083 | 0.0118 | 0.0173 | 0.0233 |
| H | 37 | -0.0526 | 0.0188 | 0.0385 | 0.0251 |
| H | 38 | -0.0581 | 0.017 | 0.0365 | 0.0277 |
| Cl | 39 | -0.0023 | 0.0166 | 0.0158 | 0.0164 |
