# Supplementary material for: Breaking the activity-selectivity trade-off in Fenton-like catalysis by d-orbital modulation of single-atom sites within a nano-island-like structure
Source: Nat Commun. 2026 Jun 8;17:7293. doi: 10.1038/s41467-026-74072-2 (PMC13402592; doi:10.1038/s41467-026-74072-2)
Supplement: Supplementary file 4 — Supplementary Data 2 [file 41467_2026_74072_MOESM4_ESM.docx]

**Supplementary Data 2.** Natural population analysis (NPA) charge group and condensed Fukui index for electrophilic attack (f ^–^) at B3LYP/6–31G(d) level of OFX.

| **ATOM** | **No** | **Charge(0)**  **(e/Å^3^)** | **Charge(-1)**  **(e/Å^3^)** | **Charge(+1)**  **(e/Å^3^)** | **f^-^** |
| --- | --- | --- | --- | --- | --- |
| F | 1 | -0.0905 | -0.1223 | -0.0708 | 0.0197 |
| O | 2 | -0.117 | -0.1407 | -0.0892 | 0.0278 |
| O | 3 | -0.2756 | -0.3583 | -0.2385 | 0.0371 |
| O | 4 | -0.1596 | -0.1838 | -0.1461 | 0.0134 |
| O | 5 | -0.3046 | -0.3493 | -0.2713 | 0.0333 |
| N | 6 | -0.0671 | -0.0769 | 0.0318 | 0.0989 |
| N | 7 | -0.0005 | -0.0337 | 0.0138 | 0.0143 |
| N | 8 | -0.1002 | -0.1024 | -0.0602 | 0.04 |
| C | 9 | -0.01 | -0.0137 | 0.0085 | 0.0185 |
| C | 10 | -0.0085 | -0.0136 | 0.0091 | 0.0176 |
| C | 11 | 0.039 | 0.0317 | 0.0443 | 0.0053 |
| C | 12 | 0.0247 | -0.0145 | 0.0444 | 0.0197 |
| C | 13 | 0.0197 | 0.0113 | 0.0345 | 0.0148 |
| C | 14 | -0.1223 | -0.0708 | 0.0977 | 0.0549 |
| C | 15 | -0.1407 | -0.0892 | 0.0197 | 0.0318 |
| C | 16 | -0.3583 | -0.2385 | 0.0278 | 0.0318 |
| C | 17 | -0.1838 | -0.1461 | 0.0371 | 0.0237 |
| C | 18 | -0.3493 | -0.2713 | 0.0134 | 0.0826 |
| C | 19 | -0.0769 | 0.0318 | 0.0333 | 0.0447 |
| C | 20 | -0.0337 | 0.0138 | 0.0989 | 0.0098 |
| C | 21 | -0.1024 | -0.0602 | 0.0143 | 0.0333 |
| C | 22 | -0.0137 | 0.0085 | 0.04 | 0.0022 |
| C | 23 | -0.0136 | 0.0091 | 0.0185 | 0.0037 |
| C | 24 | 0.0317 | 0.0443 | 0.0176 | 0.0051 |
| C | 25 | -0.0145 | 0.0444 | 0.0053 | 0.0074 |
| C | 26 | 0.0113 | 0.0345 | 0.0197 | 0.0392 |
| H | 27 | -0.0708 | 0.0977 | 0.0148 | 0.0084 |
| H | 28 | -0.0892 | 0.0197 | 0.0549 | 0.0372 |
| H | 29 | -0.2385 | 0.0278 | 0.0318 | 0.0258 |
| H | 30 | -0.1461 | 0.0371 | 0.0237 | 0.0258 |
| H | 31 | -0.2713 | 0.0134 | 0.0826 | 0.0599 |
| H | 32 | 0.0318 | 0.0333 | 0.0242 | 0.0188 |
| H | 33 | 0.0138 | 0.0989 | 0.0447 | 0.039 |
| H | 34 | -0.0602 | 0.0143 | 0.0098 | 0.0544 |
| H | 35 | 0.0085 | 0.04 | 0.0333 | 0.0238 |
| H | 36 | 0.0091 | 0.0185 | 0.0022 | 0.0211 |
| H | 37 | 0.0443 | 0.0176 | 0.0037 | 0.0111 |
| H | 38 | 0.0444 | 0.0053 | 0.0051 | 0.0113 |
| H | 39 | 0.0345 | 0.0197 | 0.0074 | 0.0063 |
| H | 40 | 0.0977 | 0.0197 | 0.0197 | 0.0392 |
| H | 41 | 0.0197 | 0.0549 | 0.0148 | 0.0084 |
| H | 42 | 0.0278 | 0.0318 | 0.0372 | 0 |
| H | 43 | 0.0371 | 0.0237 | 0.0258 | 0.0121 |
| H | 44 | 0.0134 | 0.0826 | 0.0258 | -0.0041 |
| H | 45 | 0.0333 | 0.0242 | 0.0599 | 0.0456 |
| H | 46 | 0.0989 | 0.0447 | 0.0188 | 0.0108 |
