# Supplementary material for: Breaking the activity-selectivity trade-off in Fenton-like catalysis by d-orbital modulation of single-atom sites within a nano-island-like structure
Source: Nat Commun. 2026 Jun 8;17:7293. doi: 10.1038/s41467-026-74072-2 (PMC13402592; doi:10.1038/s41467-026-74072-2)
Supplement: Supplementary file 5 — Supplementary Data 3 [file 41467_2026_74072_MOESM5_ESM.docx]

**Supplementary Data 3.** Impact assessment for CoN_3_C/rGO+PMS system.

| Impact project | Unit | Total | Co(NO_3_)_2_*6H_2_O | Formamide | Reduced Graphene Oxide | peroxymonosulfate | Power supply | Pumps (Fenton reactor) | Stirrer (Fenton reactor) |
| --- | --- | --- | --- | --- | --- | --- | --- | --- | --- |
| Global warming | kg CO_2_ eq | 127.88624 | 0.61772569 | 82.233337 | 0.00197731 | 0.27904968 | 39.886328 | 3.5763582 | 1.2914627 |
| Stratospheric ozone depletion | kg CFC11 eq | 2.40E-05 | 4.05E-07 | 1.38E-05 | 6.78E-10 | 8.20E-08 | 8.69E-06 | 7.79E-07 | 2.81E-07 |
| Ionizing radiation | kBq Co-60 eq | 5.4431688 | 0.14258168 | 3.4669967 | 0.00010696 | 0.01768546 | 1.6182972 | 0.14510261 | 0.05239817 |
| Ozone formation, Human health | kg NO_x_ eq | 0.26581176 | 0.00133821 | 0.14161342 | 6.79E-06 | 0.00068731 | 0.10887828 | 0.00976244 | 0.00352532 |
| Fine particulate matter formation | kg PM2.5 eq | 0.17332632 | 0.00138557 | 0.10202562 | 4.69E-06 | 0.00072958 | 0.0616562 | 0.00552833 | 0.00199634 |
| Ozone formation, Terrestrial ecosystems | kg NO_x_ eq | 0.2767378 | 0.00141534 | 0.15172986 | 6.91E-06 | 0.00071581 | 0.10950555 | 0.00981868 | 0.00354563 |
| Terrestrial acidification | kg SO_2_ eq | 0.40005201 | 0.00376988 | 0.2369192 | 8.64E-06 | 0.00186648 | 0.14035816 | 0.01258504 | 0.0045446 |
| Freshwater eutrophication | kg P eq | 0.04661523 | 0.00034339 | 0.02873204 | 9.11E-07 | 0.00014523 | 0.01550178 | 0.00138995 | 0.00050193 |
| Marine eutrophication | kg N eq | 0.00220488 | 5.34E-05 | 0.00158145 | 4.66E-08 | 1.06E-05 | 0.00049857 | 4.47E-05 | 1.61E-05 |
| Terrestrial ecotoxicity | kg 1,4-DCB | 1118.5354 | 24.545244 | 974.2476 | 0.01318374 | 3.6734117 | 103.43275 | 9.2741696 | 3.3490057 |
| Freshwater ecotoxicity | kg 1,4-DCB | 4.6405962 | 0.09937589 | 2.4318047 | 4.72E-05 | 0.04198106 | 1.8425218 | 0.16520743 | 0.05965824 |
| Marine ecotoxicity | kg 1,4-DCB | 6.8534314 | 0.13912916 | 3.9492295 | 7.63E-05 | 0.0563396 | 2.4140416 | 0.21645205 | 0.07816324 |
| Human carcinogenic toxicity | kg 1,4-DCB | 16.280304 | 0.15075798 | 10.642229 | 0.00034508 | 0.08337105 | 4.8158624 | 0.43180834 | 0.15593079 |
| Human non-carcinogenic toxicity | kg 1,4-DCB | 111.85574 | 2.7270856 | 63.182876 | 0.00160515 | 0.84620211 | 40.192756 | 3.6038337 | 1.3013844 |
| Land use | m^2^a crop eq | 2.3365524 | 0.02344259 | 1.5535157 | 5.85E-05 | 0.0082276 | 0.66958973 | 0.06003793 | 0.02168037 |
| Mineral resource scarcity | kg Cu eq | 0.3974886 | 0.16463294 | 0.16286874 | 0.01010782 | 0.00270642 | 0.05095412 | 0.00456874 | 0.00164982 |
| Fossil resource scarcity | kg oil eq | 39.485621 | 0.18048208 | 30.15405 | 0.00045492 | 0.0772285 | 8.0865091 | 0.72506682 | 0.26182969 |
| Water consumption | m^3^ | 0.99325892 | 0.0966371 | 0.76458094 | 8.15E-06 | 0.00685195 | 0.11156512 | 0.01000335 | 0.00361232 |
