# Supplementary material for: Breaking the activity-selectivity trade-off in Fenton-like catalysis by d-orbital modulation of single-atom sites within a nano-island-like structure
Source: Nat Commun. 2026 Jun 8;17:7293. doi: 10.1038/s41467-026-74072-2 (PMC13402592; doi:10.1038/s41467-026-74072-2)
Supplement: Supplementary file 6 — Supplementary Data 4 [file 41467_2026_74072_MOESM6_ESM.docx]

**Supplementary Table 19.** Impact assessment for CoN_3_C +PMS system.

| Impact project | Unit | Total | Co(NO_3_)_2_*6H_2_O | Formamide | peroxymonosulfate | Power supply | Pumps (Fenton reactor) | Stirrer (Fenton reactor) |
| --- | --- | --- | --- | --- | --- | --- | --- | --- |
| Global warming | kg CO_2_ eq | 758.19063 | 4.1078758 | 666.05289 | 0.55809936 | 82.60394 | 3.5763582 | 1.2914627 |
| Stratospheric ozone depletion | kg CFC11 eq | 0.00013355 | 2.69E-06 | 0.00011164 | 1.64E-07 | 1.80E-05 | 7.79E-07 | 2.81E-07 |
| Ionizing radiation | kBq Co-60 eq | 32.613615 | 0.94816814 | 28.081108 | 0.03537093 | 3.3514673 | 0.14510261 | 0.05239817 |
| Ozone formation, Human health | kg NO_x_ eq | 1.3960514 | 0.00889908 | 1.1470048 | 0.00137461 | 0.22548515 | 0.00976244 | 0.00352532 |
| Fine particulate matter formation | kg PM2.5 eq | 0.97224829 | 0.00921403 | 0.82636143 | 0.00145915 | 0.127689 | 0.00552833 | 0.00199634 |
| Ozone formation, Terrestrial ecosystems | kg NO_x_ eq | 1.4799356 | 0.00941204 | 1.2289434 | 0.00143163 | 0.22678423 | 0.00981868 | 0.00354563 |
| Terrestrial acidification | kg SO_2_ eq | 2.2555503 | 0.02506973 | 1.9189385 | 0.00373296 | 0.29067949 | 0.01258504 | 0.0045446 |
| Freshwater eutrophication | kg P eq | 0.26928638 | 0.00228353 | 0.23271657 | 0.00029047 | 0.03210394 | 0.00138995 | 0.00050193 |
| Marine eutrophication | kg N eq | 0.01427872 | 0.00035519 | 0.01280906 | 2.11E-05 | 0.00103252 | 4.47E-05 | 1.61E-05 |
| Terrestrial ecotoxicity | kg 1,4-DCB | 8288.369 | 163.22588 | 7890.9656 | 7.3468234 | 214.20756 | 9.2741696 | 3.3490057 |
| Freshwater ecotoxicity | kg 1,4-DCB | 24.48203 | 0.66084964 | 19.69652 | 0.08396213 | 3.8158327 | 0.16520743 | 0.05965824 |
| Marine ecotoxicity | kg 1,4-DCB | 38.31892 | 0.92520891 | 31.986975 | 0.1126792 | 4.999441 | 0.21645205 | 0.07816324 |
| Human carcinogenic toxicity | kg 1,4-DCB | 97.927841 | 1.0025406 | 86.197246 | 0.16674209 | 9.9735731 | 0.43180834 | 0.15593079 |
| Human non-carcinogenic toxicity | kg 1,4-DCB | 619.72405 | 18.135119 | 511.75276 | 1.6924042 | 83.238547 | 3.6038337 | 1.3013844 |
| Land use | m^2^a crop eq | 14.223551 | 0.1558932 | 12.582775 | 0.01645519 | 1.3867095 | 0.06003793 | 0.02168037 |
| Mineral resource scarcity | kg Cu eq | 2.5311288 | 1.094809 | 1.3191632 | 0.00541284 | 0.10552516 | 0.00456874 | 0.00164982 |
| Fossil resource scarcity | kg oil eq | 263.32277 | 1.2002058 | 244.23418 | 0.15445701 | 16.747029 | 0.72506682 | 0.26182969 |
| Water consumption | m^3^ | 7.0937661 | 0.64263673 | 6.1927603 | 0.01370389 | 0.23104957 | 0.01000335 | 0.00361232 |
