# Supplementary material for: Breaking the activity-selectivity trade-off in Fenton-like catalysis by d-orbital modulation of single-atom sites within a nano-island-like structure
Source: Nat Commun. 2026 Jun 8;17:7293. doi: 10.1038/s41467-026-74072-2 (PMC13402592; doi:10.1038/s41467-026-74072-2)
Supplement: Supplementary file 7 — Supplementary Data 5 [file 41467_2026_74072_MOESM7_ESM.docx]

**Supplementary Data 5.** Impact assessment for Co_3_O_4_ +PMS system.

| Impact project | Unit | Total | Co(NO_3_)_2_*6H_2_O | peroxymonosulfate | NaOH | H_2_O | Power supply | Pumps (Fenton reactor) | Stirrer (Fenton reactor) |
| --- | --- | --- | --- | --- | --- | --- | --- | --- | --- |
| Global warming | kg CO_2_ eq | 185.77793 | 30.855398 | 1.3952484 | 41.57575 | 0.14066362 | 100.28704 | 3.5763582 | 7.9474626 |
| Stratospheric ozone depletion | kg CFC11 eq | 6.17E-05 | 2.02E-05 | 4.10E-07 | 1.66E-05 | 9.94E-08 | 2.18E-05 | 7.79E-07 | 1.73E-06 |
| Ionizing radiation | kBq Co-60 eq | 14.90546 | 7.1219547 | 0.08842732 | 3.1506909 | 0.00791495 | 4.0689191 | 0.14510261 | 0.32245025 |
| Ozone formation, Human health | kg NO_x_ eq | 0.48318296 | 0.06684346 | 0.00343653 | 0.10736668 | 0.00032459 | 0.27375498 | 0.00976244 | 0.0216943 |
| Fine particulate matter formation | kg PM2.5 eq | 0.32715693 | 0.06920916 | 0.00364788 | 0.0811173 | 0.00034559 | 0.15502351 | 0.00552833 | 0.01228517 |
| Ozone formation, Terrestrial ecosystems | kg NO_x_ eq | 0.49147794 | 0.07069644 | 0.00357907 | 0.10989698 | 0.00033534 | 0.27533215 | 0.00981868 | 0.02181929 |
| Terrestrial acidification | kg SO_2_ eq | 0.73930555 | 0.18830574 | 0.0093324 | 0.14734989 | 0.00086019 | 0.35290552 | 0.01258504 | 0.02796676 |
| Freshwater eutrophication | kg P eq | 0.08289325 | 0.01715222 | 0.00072617 | 0.02149029 | 6.94E-05 | 0.03897646 | 0.00138995 | 0.00308877 |
| Marine eutrophication | kg N eq | 0.00572937 | 0.0026679 | 5.28E-05 | 0.00160667 | 4.44E-06 | 0.00125356 | 4.47E-05 | 9.93E-05 |
| Terrestrial ecotoxicity | kg 1,4-DCB | 1819.1838 | 1226.035 | 18.367059 | 283.03014 | 1.8049939 | 260.06317 | 9.2741696 | 20.609266 |
| Freshwater ecotoxicity | kg 1,4-DCB | 12.508827 | 4.9638255 | 0.20990531 | 2.149968 | 0.02010147 | 4.6326917 | 0.16520743 | 0.36712762 |
| Marine ecotoxicity | kg 1,4-DCB | 17.047777 | 6.9495015 | 0.281698 | 3.0223795 | 0.02706479 | 6.0696762 | 0.21645205 | 0.48100455 |
| Human carcinogenic toxicity | kg 1,4-DCB | 29.622313 | 7.5303612 | 0.41685524 | 8.128844 | 0.04624471 | 12.108625 | 0.43180834 | 0.95957408 |
| Human non-carcinogenic toxicity | kg 1,4-DCB | 305.84887 | 136.21793 | 4.2310106 | 52.320871 | 0.40920834 | 101.0575 | 3.6038337 | 8.0085192 |
| Land use | m^2^a crop eq | 4.1577671 | 1.1709572 | 0.04113798 | 1.0645112 | 0.00414155 | 1.6835637 | 0.06003793 | 0.13341763 |
| Mineral resource scarcity | kg Cu eq | 8.5161211 | 8.2234152 | 0.01353211 | 0.13473755 | 0.00159968 | 0.12811503 | 0.00456874 | 0.01015275 |
| Fossil resource scarcity | kg oil eq | 42.47426 | 9.01508 | 0.38614252 | 10.370427 | 0.03420195 | 20.332082 | 0.72506682 | 1.6112596 |
| Water consumption | m^3^ | 5.9814613 | 4.8270233 | 0.03425973 | 0.52474312 | 0.28269161 | 0.28051057 | 0.01000335 | 0.02222966 |
