# Supplementary material for: Breaking the activity-selectivity trade-off in Fenton-like catalysis by d-orbital modulation of single-atom sites within a nano-island-like structure
Source: Nat Commun. 2026 Jun 8;17:7293. doi: 10.1038/s41467-026-74072-2 (PMC13402592; doi:10.1038/s41467-026-74072-2)
Supplement: Supplementary file 12 — Transparent Peer Review file [file 41467_2026_74072_MOESM12_ESM.pdf]

nature portfolio

Peer Review File

Breaking the activity-selectivity trade-off in Fenton-like catalysis by d-orbital modulation of single-atom sites within a nano-island-like structure

Corresponding Author: Professor Liang Tang

This file contains all reviewer reports in order by version, followed by all author rebuttals in order by version.

Version 0:

Reviewer comments:

Reviewer #1

(Remarks to the Author)
This manuscript reported a strategy to guide the activation pathway of peroxymonosulfate (PMS) by regulating the electronic occupation state of Co single atom through an "island-sea" structure. This work represents a valuable contribution in breaking the traditional trade-off between activity and selectivity in Fenton-like reaction. Overall, the work is novel, the mechanism is clear, and the catalyst and method demonstrate great potential in water treatment. Thus, I recommend this manuscript to Nature Communications. Before the final acceptance, some minor revisions are required.
1. Why are reduced graphene oxide and carbon nitride chosen as the "sea" and "island"? What are the advantages of these materials? The explanation should be added.
2. The author mentioned that "more defects were generated in CoN3C/rGO" according to the Raman spectra results. So what did the roles of these defects play in catalysis? Will they influence the activity or selectivity of the catalysts?
3. N species are important in Fenton-like reactions. CoN3C/rGO has more types of N species than CoN3C. Why? How is their influence on the performance of the catalysts?
4. Fig.2c: the activity comparison of various catalysts is specifically for BPA degradation. The authors need to make it clear in the manuscript rather than in SI.
5. What are the concentrations of various ROS in the CoN3C/PMS system, and what are their contribution ratios? These data are crucial for mechanisms discussion.
6. The authors should compare the free energy between the free radical pathway and ETP.
7. The authors are suggested to determine the chemical states and the contents of C and N elements in the used CoN3C/rGO, and evaluate the stability of the catalysts.
8. More experiments, such as the screening experiment, should be provided to identify the dominant active sites.

Reviewer #2

(Remarks to the Author)
The manuscript addresses the critical challenge of precisely regulating the direct electron transfer (ETP) pathway during peroxymonosulfate (PMS) activation. A "nano-island" Co single-atom catalyst (CoN_3C/rGO) is designed and synthesized, where carbon nitride serves as "islands" and reduced graphene oxide (rGO) as the "sea". This synergistic "island-sea" structure achieves the simultaneous enhancement of activity and selectivity in the Fenton-like system. Focusing on the core demand of advanced oxidation water treatment technology, the study features an innovative design concept and a comprehensive experimental setup. It combines multiple characterization techniques (XRD, TEM/HAADF-STEM, XAFS, XPS etc.), performance evaluations, and density functional theory (DFT) calculations to thoroughly elucidate the molecular mechanism underlying the regulation of the ETP pathway by the "island-sea" structure from both experimental and theoretical perspectives. The research outcomes exhibit remarkable advantages in catalytic activity, selectivity, stability, and practical application potential, providing crucial theoretical support and technical references for the directional regulation of reactive oxygen species (ROS) in advanced oxidation processes (AOPs). This work holds significant academic value and promising application prospects. My comments are as follows:
(1) The manuscript reports the ETP pathway contribution rate as "approximately 94%" and "as high as ~92.5%". Although this discrepancy may stem from different calculation methods or experimental conditions, the underlying reasons are not clearly explained, which may confuse readers. It is recommended to explicitly clarify the calculation methods for the two sets of data (e.g., indirect calculation based on quenching experiments vs. direct calculation via electrochemical tests) and verify data reliability through repeated experiments. If the difference arises from varying experimental conditions, detailed

information on how specific parameters (e.g., catalyst concentration, PMS concentration) affect the results should be provided, and the core data representation should be standardized.
(2) The influence of carbon nitride "island" size (e.g., particle size variation under different hydrothermal conditions) and rGO "sea" loading on catalyst structure and performance has not been investigated. This makes it impossible to determine the optimal parameter range of the "island-sea" structure or quantify the contributions of "island" size and "sea" loading to electron transfer efficiency and orbital regulation efficacy. It is suggested to supplement experiments on the optimization of "island-sea" structural parameters and carrier comparison: Prepare carbon nitride "islands" of different sizes by adjusting hydrothermal temperature, reaction time, and other parameters; characterize particle size distribution via TEM; and identify the optimal "island" size range through catalytic performance tests. Investigate the effect of rGO loading (e.g., 0.05 g, 0.1 g, 0.2 g) on electronic structure (XPS valence band, UPS) and catalytic performance.
(3) The influence of different "sea" carriers (e.g., carbon nanotubes, porous carbon) on the electronic structure and catalytic performance of CoN3C has not been compared. This fails to fully demonstrate the unique advantages of rGO as the "sea" carrier and weakens the argument for the universality of the "island-sea" structural design strategy. It is recommended to select carbon nanotubes and porous carbon as alternative "sea" carriers, synthesize CoN3C/carbon nanotube and CoN3C/porous carbon catalysts, and compare their activity and selectivity in PMS activation and BPA degradation.
(4) The continuous-flow experiment only reports 36-hour operation results. Long-term stability data (e.g., over 100 hours) are lacking, which is insufficient to verify the wear resistance, active site retention rate, and long-term leaching risk of the catalyst membrane coating. Supplementary long-term stability tests are recommended.
(5) The manuscript does not specify the PMS dosing method (e.g., one-time addition vs. continuous supplementation) or the influence of water flow rate on degradation efficiency in the continuous-flow system, nor does it provide membrane flux data. This hinders future engineering scaling. It is suggested to supplement relevant information.
(6) In the XAFS analysis, although the Co-N3 coordination configuration is confirmed by EXAFS fitting, the original k-space data and fitting residual plots are not provided, making it difficult to evaluate fitting quality. For the XANES spectra, the Co valence state is reported to be between +2 and +3, but linear combination fitting has not been used to quantitatively analyze the Co2+/Co3+ ratio or the effect of rGO introduction on Co valence. It is recommended to: Supplement the original k-space XAFS data and fitting residual plots. Perform LCF on XANES spectra to quantify the Co2+/Co3+ ratio and clarify the influence of rGO on Co valence state.
(7) In the DFT calculations, it is recommended to clarify whether solvent effects on PMS adsorption energy and electron transfer efficiency are considered. The Gibbs free energy barrier for the ETP process has not been calculated, which prevents further verification of the "island-sea" structure’s promotional effect on the ETP pathway from a thermodynamic perspective. It is suggested to: Supplement the calculation of the Gibbs free energy barrier for the ETP process to verify the "island-sea" structure’s thermodynamic advantage. Provide charge density difference maps and orbital overlap integral data to more intuitively demonstrate the directional hybridization between the Co dz2 orbital and the PMS p orbital.

Reviewer #3

(Remarks to the Author)
The work by Y. Chen and co-workers (NCOMMS-25-87289) tries to explain how to overcome the persistent trade-off between activity and selectivity in Fenton-type catalysis, particularly in peroxymonosulfate (PMS) activation. The authors designed a single-atom cobalt catalyst with a “nano-island” structure (called CoN_3C/rGO) to direct PMS activation toward the desired direct Electron Transfer Pathway (ETP) process, a non-radical mechanism known for its high selectivity and resistance to matrix interference. The authors hypothesize that the catalyst consists of single cobalt atoms anchored on islands of carbon nitride (CoN_3C) supported by a layer of reduced graphene oxide (rGO), which acts as a "sea". The "island-sea" architecture exploits the synergistic effect to precisely modulate the electrons in the cobalt d-orbital. Theoretical analyses show that rGO elevates the center of the Co d-band and makes the electronic state of the d-orbital more concentrated and intense near the Fermi level. However, a clear experimental characterization, to support the predictions made by the DFT simulation, is lacking. The XPS technique, for example, partially used in the study, could have provided several useful insights if the Co2p and valence band regions had also been analyzed. The latter, although we all know that UPS is the best technique for valence band analysis, could have as well provided information on the Co3d, O2p, and N2p orbitals (the latter would have provided interesting information also on the hybridization of nitrogen, sigma and π bonds, etc.) and on the position of the valence band maximum with respect to the Fermi level, to directly verify the presence or absence of "occupied" states in its vicinity.
What emerges from this work is undoubtedly a huge amount of data acquired using various techniques, some complementary, which, however, have not been adequately commented on or analyzed. Furthermore, the reader struggles to follow the work's logical thread, as the different sections appear disjointed. Fewer analyses could have been performed, but more time and precision could have been devoted to presenting the results. At the same time, the manuscript contains numerous errors and omissions, making the writing and subsequent editing rather careless. This is unfortunate, given the amount of work done. To give a few more explicit examples:
• If you use ETP acronym, then you should write instead "direct electron transfer pathway (ETP)", otherwise you should use the acronym DET (direct electron transfer);
• When authors stated “Here, we utilize a cobalt single atom catalyst anchored on carbon nitride supported by reduced graphene oxide (CoN3C/rGO) to activate PMS”, then experimental evidence of this connection should be added. XAS measurements have not demonstrated it precisely and unequivocally, for instance. “In the X-ray absorption near-edge structure (XANES) spectroscopy, the adsorption of CoN3C/rGO and CoN3C is very near to Co3O4, indicating the valence of Co in two catalysts is between +2 and +3, while rGO further decreases the electron density of Co site (Fig. 1g). The extended X-ray absorption fine structure (EXAFS) spectra reveals that no Co-Co interaction exists in CoN3/rGO and CoN3C, while obvious Co-N can be observed at 1.4-1.6 Å, further demonstrating the single atom form of Co”. If the authors hypothesized a direct Co-N bond, why didn't they use XPS to confirm this hypothesis? The literature contains several articles that refer to this type of bond and the associated chemical shifts, which are distinguishable (“Atomically dispersed

cobalt on carbon nitride for peroxymonosulfate activation: Switchable catalysis enabled by light irradiation" https://doi.org/10.1016/j.cej.2022.137277; "Regulating Second-Shell Coordination in Cobalt Single-Atom Catalysts toward Highly Selective Hydrogenation" https://doi.org/10.1021/acsnano.4c05637; "Cobalt/nitrogen doped porous carbon as catalysts for efficient oxygen reduction reaction: Towards hybrid enzymatic biofuel cells" https://doi.org/10.1016/j.electacta.2021.138791). What the authors demonstrated with XAS measurements is that the average oxidation state of Co was between +2 and +3, but this does not allow us to distinguish between a "doping" inclusion or a simple functionalization, with Co bound to existing functionalities on both rGO and/or -N3C, for example;
• if authors state that there are species related to N-oxidised in the N1s region, which is the counterpart in O1s region? Where is the chemical shift related to this N-O bond?
• peak assignments in the C1s and N1s regions are inconclusive and in some cases incorrect. For example, for the N-C=N bond, it is initially positioned at around 286 eV for the CoN3C/rGO sample, then shifts, for no apparent reason, to around 288.5 eV for the CoN3C sample. In some cases, the FWHM of some components is on the order of around 1.5-2 eV, while in others it reaches almost 4 eV, for no apparent reason, as these peaks are always related to real bonds and not satellites, loss or shake-up features. The N1s region also presents several inconsistencies: N-pyridine is initially positioned at around 398.5 eV (CoN3C/rGO), then shifts to around 397.8 eV (CoN3C), with no explanation for the shift. And what about the * peaks, both in C1s and N1s regions, which have been suggested by other techniques results?
• "Density function theory (DFT)", should be "functional";
• Which is the precursor used for the Co: Co(NO3)2•6H2O or CoCl2? The first has been mentioned in the sample preparation procedure, while the second is reported in fig. 1-a;
• Co signal is not clearly detectable in all the EDS maps presented in the manuscript or SI: which is the amount of Co in these samples. What about survey spectra from XPS analysis or EDS?;
• Please do not use terms like "exceptional" ("Exceptional resistance to matrix interference…") or "obvious" ("According to Raman spectra, the obvious D band…"), they are a bit out of place in a scientific text.
I suggest the authors to read the following articles, to understand how to display data and to correlate results: "A review of the innovations in metal- and carbon-based catalysts explored for heterogeneous peroxymonosulfate (PMS) activation, with focus on radical vs. non-radical degradation pathways of organic contaminants" (https://doi.org/10.1016/j.cej.2020.127957), "Advancements on Single-Atom Catalysts-Mediated Persulfate Activation: Generating Reactive Species for Contaminants Elimination in Water" (https://doi.org/10.3390/molecules29235696), or "Transition Metal-Nitrogen-Carbon Single-Atom Catalysts Enhanced CO2 Electroreduction Reaction: A Review." (https://doi.org/10.1002/cssc.202401557).
Although the ETP demonstrated a contribution of approximately 94% and was verified by a two-cell electron transfer experiment, the suggestion in the introduction that the ETP avoids directly introducing PMS into the wastewater (e.g., using a salt bridge device) to circumvent the environmental concern arising from SO42−, has not been fully implemented in the final continuous application (which still involves the addition of PMS for treatment). Furthermore, the study demonstrated that the system has variable selectivity for different contaminants (e.g., BPA, ofloxacin, phenol, etc.), and established a structure-activity relationship (QSAR) indicating that electron-donating organic contaminants are more readily degraded. However, detailed mechanistic analysis (such as intermediate degradation pathways) was provided only for bisphenol A (BPA), leaving specific orbital/electronic interactions with the other tested contaminants less explored.
Finally, the conclusions presented are not sufficiently supported by the data and analyses in their current form. It is difficult to recommend publication of this manuscript in Nature Communications in this form. The article as a whole needs to be revised, first to correct the numerous inaccuracies and errors already cited, and then to restructure the presentation of the results, with more in-depth commentary on the results, in order to make the proposed theses irrefutable.

Version 1:

Reviewer comments:

Reviewer #1

(Remarks to the Author)
The authors addressed all comments. The paper has been improved and is ready for publication.

Reviewer #2

(Remarks to the Author)
The authors have provided comprehensive and convincing responses to each of my comments. All the necessary experiments, characterizations, data analyses, and discussions have been properly supplemented and revised. The scientific quality and presentation of the manuscript are now satisfactory. I recommend to accept the manuscript.

Reviewer #3

(Remarks to the Author)
Dear authors, first of all, I thank you for the extensive work you have done to address my requests and those of my fellow reviewers, filling the gaps and uncertainties identified.

Regarding the implementation of the new section I requested, I still have some concerns regarding the XPS measurements.

First, I kindly ask you to switch from the "line + symbol" representation for the fit curves to simple lines, since the chosen circles almost entirely cover the "raw" signal of the experimental data and the quality of the fits is difficult to assess.
Regarding the Co2p regions, however, I believe the reported fits are flawed in terms of the choice of the number of curves to

use to fit the components due to the two oxidation states +2 and +3, which involve several satellites and multiple splitting (have a look at this paper for an example about Co2p correct deconvolution procedure: Applied Surface Science 257 (2011) 2717–2730). Furthermore, especially for images in SI 6 and 27, the signal quality of the Co2p regions is so poor, with a very low signal-to-noise ratio, that you are essentially trying to fit the noise itself. Furthermore, judging by the position and relative intensities of the different components, no constraint appears to have been applied on the area ratio of the Co2p3/2 and Co2p1/2 components, as the areas of the latter do not appear to be in the 1:2 ratio, as one would expect. At this point, I would recommend fitting only those curves where it makes sense to interpret a clear and clean signal and ignoring regions where noise dominates the experimental signal. Finally, in Table 3 of the supplementary material, I would round the relative atomic concentration values to the first decimal place and verify that the total for each sample is equal to 100% and not 100.01% as in some rows of the table.
The work cannot therefore be published in its current state, as it still requires minor revisions.

Version 2:

Reviewer comments:

Reviewer #3

(Remarks to the Author)
I am satisfied with the revisions undertaken by the authors. The manuscript has benefited substantially from the changes implemented, and the authors have carefully and convincingly addressed the concerns previously raised. As a result, the overall quality, clarity, and impact of the work have improved markedly. In its current form, I consider the manuscript to meet the high standards of the journal and I am pleased to recommend it for publication.

Open Access This Peer Review File is licensed under a Creative Commons Attribution 4.0 International License, which permits use, sharing, adaptation, distribution and reproduction in any medium or format, as long as you give appropriate credit to the original author(s) and the source, provide a link to the Creative Commons license, and indicate if changes were made.
In cases where reviewers are anonymous, credit should be given to 'Anonymous Referee' and the source.
The images or other third party material in this Peer Review File are included in the article's Creative Commons license, unless indicated otherwise in a credit line to the material. If material is not included in the article's Creative Commons license and your intended use is not permitted by statutory regulation or exceeds the permitted use, you will need to obtain permission directly from the copyright holder.
To view a copy of this license, visit https://creativecommons.org/licenses/by/4.0/

Responses to the Reviewers' Comments

We thank the editor and all reviewers for their time spend making their constructive remarks and useful suggestions, which has significantly raised the quality of the manuscript. We have read all the comments and recommendations carefully, and tried our best to revise the manuscript accordingly. Each suggested revision and comment were responded point by point. The comments are in **black**, the responses are in **blue**, and the corresponding revised texts in the revised manuscript and revised Supplementary information are in **red**. All line and pages mentioned below are corresponding to **unmarked revised manuscript** and **unmarked revised Supplementary information**.

Reviewer #1:

This manuscript reported a strategy to guide the activation pathway of peroxymonosulfate (PMS) by regulating the electronic occupation state of Co single atom through an "island-sea" structure. This work represents a valuable contribution in breaking the traditional trade-off between activity and selectivity in Fenton-like reaction. Overall, the work is novel, the mechanism is clear, and the catalyst and method demonstrate great potential in water treatment. Thus, I recommend this manuscript to Nature Communications. Before the final acceptance, some minor revisions are required.

Response: We sincerely thank the reviewers for their time and valuable comments on our manuscript. We have diligently addressed all the points raised in a point-by-point manner below. We believe that the revisions made in response to these insightful suggestions have significantly strengthened the quality of the manuscript.

Comment 1. Why are reduced graphene oxide and carbon nitride chosen as the "sea" and "island"? What are the advantages of these materials? The explanation should be added.

Response: Thank you for your professional questions. We chose rGO as the "sea" and

carbon nitride as the "island" based on their unique physicochemical properties and the design strategy of synergy. rGO possesses high electrical conductivity and a delocalized π-electron system derived from its sp^2 carbon network. This enables it to efficiently accept, store, and transport electrons. In our design, the rGO "sea" engages in long-range electronic interactions with the Co single atoms residing on the "islands" via this delocalized π-electron system. This macroscopically modulates the electronic structure of the microscopic atomic centers, serving as the foundation for achieving the high selectivity. The enormous specific surface area of rGO enabling the high-density and uniform loading of "islands". Meanwhile, its layered structure is easily modifiable. The characteristic data in the manuscript indicate that after compositing with CoN_3C, the interlayer spacing of rGO decreased and it was further reduced. This optimization enhances its electron transport properties and strengthens the interfacial coupling with the "islands".

For carbon nitride "island", the pyridine nitrogen and other groups richly present in it can provide strong coordination sites for transition metal atom such as Co, forming a stable and definite Co-N coordination structure. This effectively prevents the migration and agglomeration of metal atoms during the reaction process, ensuring the stability of the single-atom catalyst.

We have added the related explanation to **Introduction** in the revised **manuscript**. Detailed information can be found below (**Line 80-85, Page 2**):

Due to the well-defined nitrogen-rich motifs that can firmly anchor transition metal atoms via coordination, creating stable and atomically dispersed active sites, carbon nitride has potential to be the "island"[22]. Meanwhile, the advantages that superior electrical conductivity, large specific surface area, and a delocalized π-electron system that can potentially modulate the electronic structure of the metal centers over long range makes reduced graphene oxide (rGO) would be a candidate of "sea"[23, 24].

Comment 2. The author mentioned that "more defects were generated in CoN_3C/rGO" according to the Raman spectra results. So what did the roles of these defects play in catalysis? Will they influence the activity or selectivity of the catalysts?

Response: Thank you for your valuable questions. We apologize for ignoring the explanation of this phenomenon in the manuscript. The presence of defects in the graphene lattice makes graphene an ideal support to anchor and stabilize noble metal nanoparticles, clusters, single atoms according to previous reports (please see: *Adv. Funct. Mater. 34, 2313530 (2024)*). Furthermore, the defects in carbon materials have been investigated that can alter the local electronic structure of the carbon lattice and create active sites and enhance the catalytic activity (please see: *Appl. Catal. B: Environ. 380 (2026); J. Phys. Chem. C 127, 6239-6247 (2023)*). However, the exceptional selectivity toward the ETP pathway is primarily governed by the orbital-level modulation of the Co centers induced by the island-sea architecture, as revealed by subsequent mechanistic studies. Detailed information added to the revised **Supplementary information** can be found below (**Page 5**):

The defect level of materials represented by the peak intensity ratio (I_D/I_G) indicate that more defects were generated in CoN_3C/rGO (0.98 vs 0.89 and 0.92), which can support more anchoring sites, and regulate the electronic structure of active site, enhancing the catalytic activity[1-3].

Comment 3. N species are important in Fenton-like reactions. CoN_3C/rGO has more types of N species than CoN_3C. Why? How is their influence on the performance of the catalysts?

Response: Thank you for your valuable and professional questions. We fully agree that species N plays a crucial role in regulating the electronic structure and catalytic performance of the catalyst. The phenomenon that CoN_3C/rGO possesses a more abundant variety of N species than CoN_3C is evidence of the “sea-island” structure we designed generating interfacial interactions during the synthesis process. The additional graphitic N and oxidized N demonstrate that the N element not only exists on the “island” but also successfully modifies the “sea” (rGO), forming a chemically bonded interface rather than only a physical mixture (please see *Nat. Commun. 16 (2025); Chin. Chem. Lett., 111903 (2025)*). Moreover, these newly formed N species have played a positive role in promoting the outstanding performance of the catalyst. For example, the

emergence of graphitic N enhances the adsorption ability and electron transfer capability of the rGO support, which would facilitate the delocalization of π-electrons from the rGO "sea" to the "island" (please see *ACS ES&T Water 2, 817-829 (2022); J. Mater. Chem. A 10, 15543-15553 (2022); Chem. Eng. J. 466, 143110 (2023)*).

The related information has been added to the revised **Supplementary information** as below (**Page 8**):

The N 1s spectra further corroborate the specific coordination environment. The spectrum of CoN_3C/rGO can be deconvoluted into three distinct peaks at 398.7 eV, 399.5 eV, and 401.0 eV, corresponding to pyridinic N, Co-N/pyrrolic N, and Graphitic N, respectively[1-4]. More importantly, higher ratio of pyrrolic N than pyridinic N exists in CoN_3C/rGO, while the opposite data is exhibited for CoN_3C. This suggests that the strong interfacial interaction with rGO induces the transformation of some rigid six-membered pyridinic rings into more flexible, electron-rich five-membered pyrrolic defects.

Comment 4. Fig.2c: the activity comparison of various catalysts is specifically for BPA degradation. The authors need to make it clear in the manuscript rather than in SI.

Response: Thank you for your valuable suggestions. We agree that specifying the target pollutant is crucial for a fair and accurate comparison of catalytic activities. Accordingly, we have revised the description of Fig. 2c in the main text to explicitly state that the performance comparison is based on the reaction rate constants (k_{obs}) for BPA degradation. The related information has been added to the revised **manuscript** as below (**Line 187-189, Page 4**):

As evidenced by normalized k_{obs}, CoN_3C/rGO exhibits superior activity for BPA degradation compared to most of the previously reported single atom catalysts and other catalysts (Fig. 2c and Supplementary Table 9).

Comment 5. What are the concentrations of various ROS in the CoN_3C/PMS system, and what are their contribution ratios? These data are crucial for mechanisms discussion.

Response: Thank you for your valuable and professional suggestions. We completely

agree with your opinion that the analysis of the concentrations of various ROS in the CoN_3C/PMS system. Hence, we conducted ROS quantitative experiment by the same method with CoN_3C/rGO/PMS system. From the result (**Figures R1-R2,** which has been as the **Supplementary Figs. 44-45** to the revised **Supplementary information**), the ETP only contributed 11% for BPA degradation in the CoN_3C/PMS system, indicating the significant regulation role of rGO sea. Related description has been added to the revised **manuscript** as below (**Line 259-262, Page 6**):

According to the result, the contribution rate of ETP achieved around 94%, demonstrating its dominant pathway for BPA degradation in CoN_3C/rGO/PMS. While for the CoN_3C/PMS system, this contribution decreases to only about 11%, further confirming the significant regulation role of rGO sea.

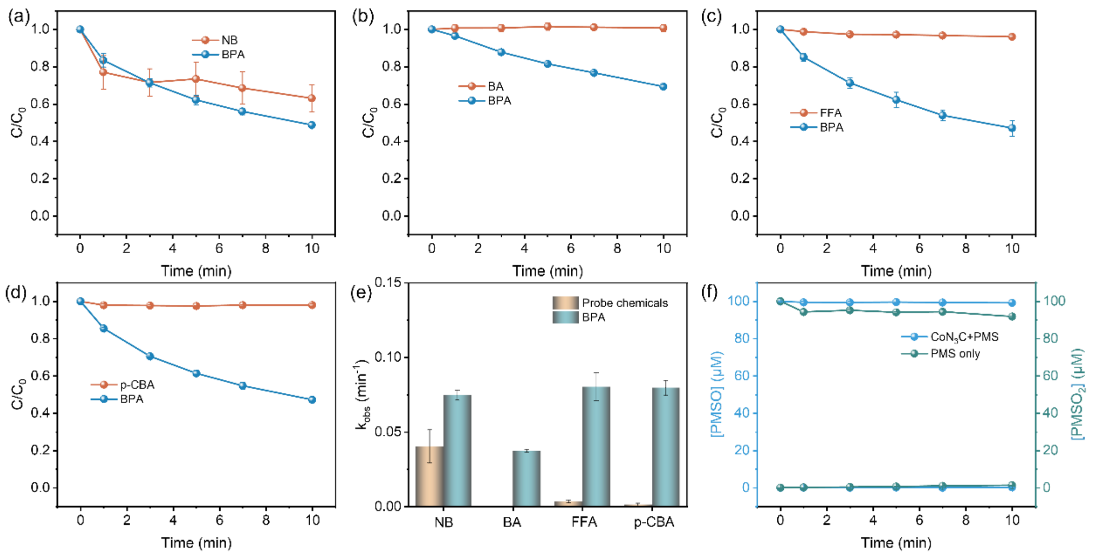

Figure R1. The ROS quantitative results of CoN_3C/PMS. The concentration change of chemical probe (a) NB, (b) BA, (c) FFA, (d) p-CBA. (e) The k_{obs} of the chemical probe reaction. (f) The PMSO and $PMSO_2$ concentration change.

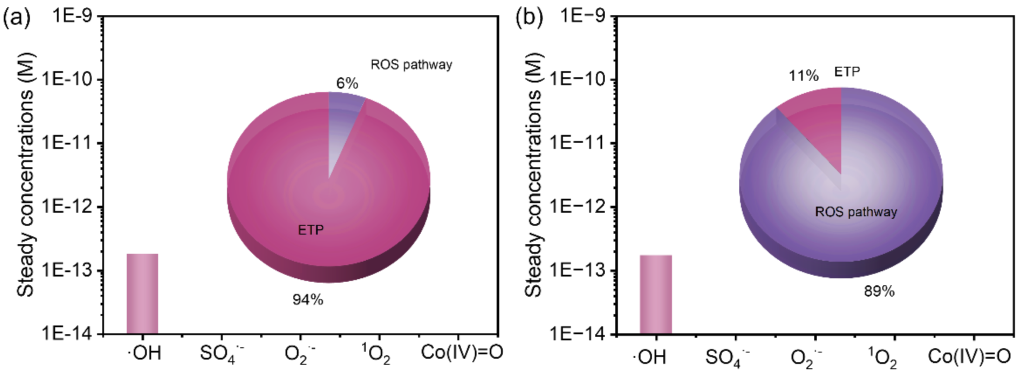

Figure R2. The contribution of various paths. The steady concentrations of the chemical probe and the contribution rate of various paths in (a) CoN_3C/rGO/PMS, (b) CoN_3C/PMS.

Comment 6. The authors should compare the free energy between the free radical pathway and ETP.

Response: Thank you for your valuable and professional suggestions. We completely agree with your opinion that the comparison of various pathways' thermodynamic trends. Hence, we calculated the Gibbs free energy profiles for PMS activation on both CoN_3C and CoN_3C/rGO to compare the difference of various pathway (**Figure R3,** which has been as the **Fig. 4b** in the revised **manuscript** and **Supplementary Fig. 49** in the revised **Supplementary information**). As shown in **Figure R3**, the formation of ·OH is thermodynamically favorable for both systems, which explains why trace ROS were detected in both cases experimentally. However, a critical difference lies in the adsorption energy (-2.659 eV of CoN_3C vs. -1.863 eV of CoN_3C/rGO), which is also confirmed by the first step in the Gibbs free energy results. For the CoN_3C, the overly strong interaction suggests an aggressive activation of the PMS molecule. This intense adsorption likely induces excessive electron back-donation into the O–O antibonding orbitals, causing the cleavage of the peroxide bond to generate radicals. This aligns with the experimental observation that the ROS pathway dominates (>89%) in CoN_3C/PMS. In contrast, the moderated adsorption strength on CoN_3C/rGO is pivotal for the mechanism switch. The weaker binding prevents the immediate rupture

of the O–O bond, preserving the PMS molecule in a metastable activated state. Coupled with the high conductivity of the rGO network, this configuration facilitates a rapid ETP process, rather than chemical bond breaking. Furthermore, the weaker adsorption favors the timely desorption of reduced species, preventing surface poisoning and accelerating the catalytic turnover. The related information has been added to the revised **manuscript** as below (**Line 275-280, Page 6**):

Combined with thermodynamic analysis provided by Gibbs free energy calculations that while radical generation is thermodynamically feasible for both systems, the dominant reaction pathway is different. For CoN$_3$C, the diffuse orbital results in an overly strong adsorption, inducing excessive O–O bond stretching and breaking (radical pathway). Conversely, CoN$_3$C/rGO features an optimized, moderate adsorption energy, which preserves the PMS molecular integrity (Supplementary Fig. 49)[32, 33].

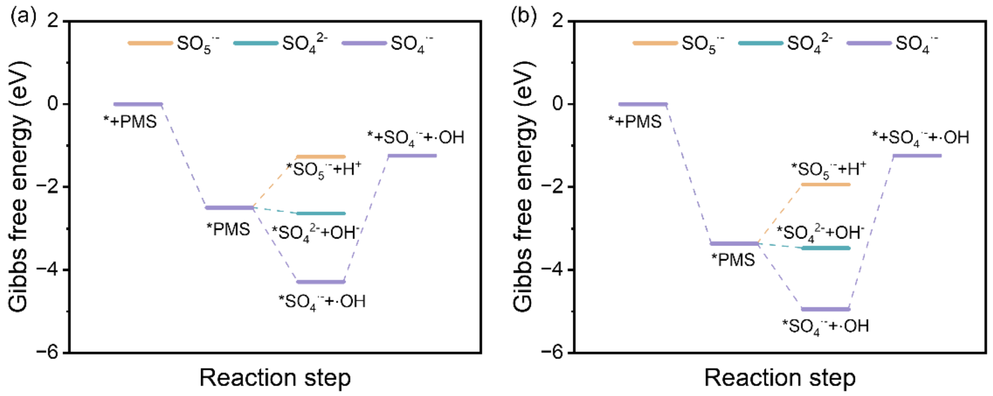

Figrue R3. The thermodynamic trends of reaction between catalysts and PMS. The Gibbs free energy change of vairous pathway in (a) CoN$_3$C/rGO/PMS, (b) CoN$_3$C/PMS.

Comment 7. The authors are suggested to determine the chemical states and the contents of C and N elements in the used CoN$_3$C/rGO, and evaluate the stability of the catalysts.

Response: Thank you for your professional suggestions. We have conducted XPS analysis on the used CoN$_3$C/rGO catalyst to investigate its chemical stability. As summarized in **Figure R4** (as the **Supplementary Fig. 35** in the revised

Supplementary information) and **Tables R1-R2** (as the **Supplementary Table 3** and **Supplementary Table 7** in the revised **Supplementary information**), the high-resolution XPS spectra reveal negligible shifts in the binding energies of C 1s and N 1s after the reaction. Specifically, the N 1s peak corresponding to pyridinic N/Co-N (~399.0 eV) remains stable, confirming that the atomic $Co\text{-}N_3$ coordination environment is well-preserved. The C 1s peak at 284.8 eV also confirms the structural integrity of the rGO support. The AAS results show that the Co loading decreased from 1.25 wt% to 0.8 wt% after the stability test. This reduction is likely attributed to the leaching of some loosely bound or unstable Co species during the initial stage of the reaction. However, the subsequent stable catalytic performance indicates that the core $Co\text{-}N_x$ active sites remain robust. Regarding the XPS results, the increase in C content (from 71.08% to 79.37%) and the corresponding decrease in N and Co atomic percentages are primarily due to the accumulation of carbonaceous reaction intermediates on the catalyst surface, which covers the active sites and attenuates the signals of other elements. Furthermore, XAFS was used to explore the coordination environment change of active site (**Figure R5**, which has been as the **Fig. 2f** in the revised **manuscript** and **Supplementary Figs. 33** in the revised **Supplementary information**). The $Co\text{-}N_3$ structure was remained and one O atom from PMS was adsorbed to the Co site according to the results. These results demonstrate that while some mass loss occurs, the chemical environment of the active sites in CoN_3C/rGO is highly stable, which is the key to its long-term stability. The related information has been added to the revised **manuscript** as below (**Line 200-207, Pages 4-5**):

The AAS results show that the Co loading decreased from 1.25 wt% to 0.8 wt% after the stability test, which is likely attributed to the leaching of some loosely bound or unstable Co species during the initial stage of the reaction (Supplementary Table 7). However, the subsequent stable catalytic performance indicates that the core Co-Nx active sites remain robust. The EXAFS and Raman spectra of the used CoN_3C*/rGO reveal that no significant changes in the coordination environment, graphitization degree and defect density of the catalyst (Fig. 2f and Supplementary Figs. 33-34). The*

high-resolution XPS spectra reveal negligible shifts in the binding energies of C 1s and N 1s after the reaction, confirming the stability of catalyst structure (Supplementary Fig. 35).

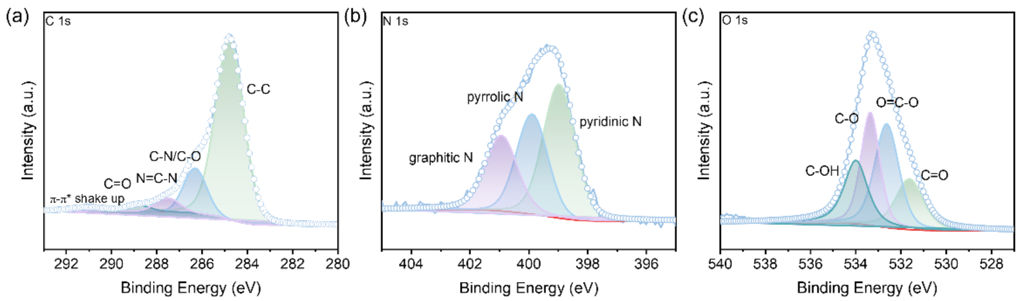

Figure R4. The chemical state of used CoN3C/rGO. High-resolution XPS spectra of (a) C 1s, (b) N 1s, (c) O 1s for used CoN3C/rGO.

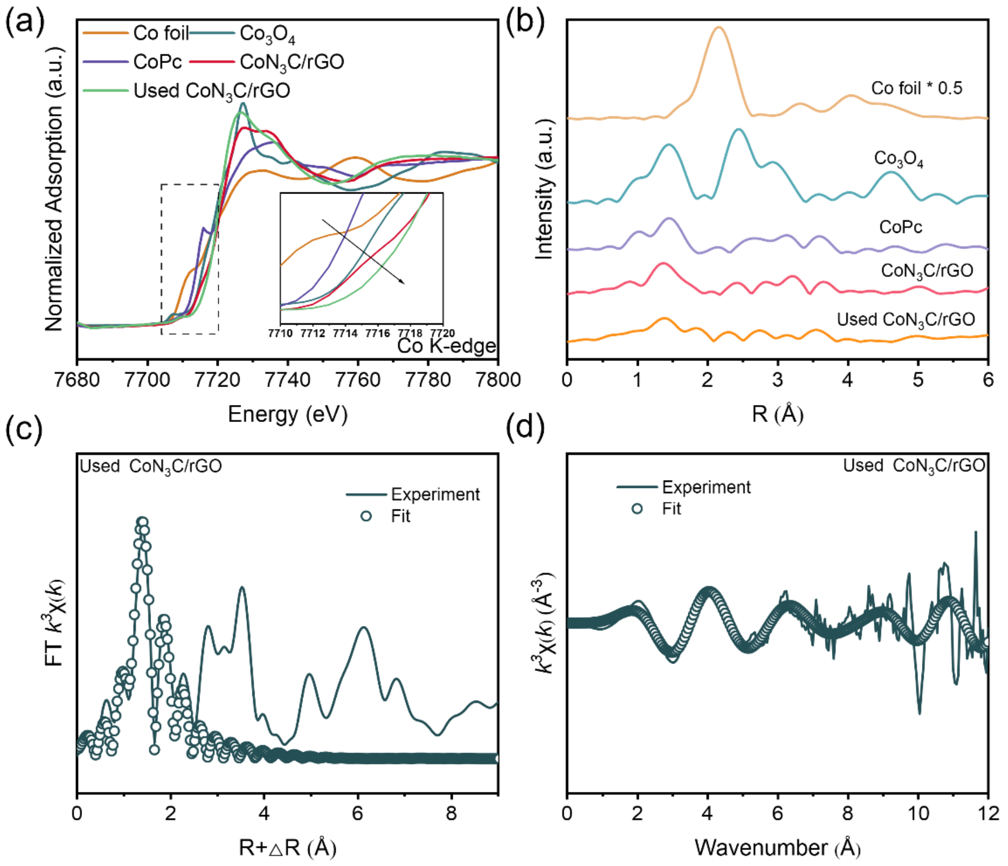

Figure R5. The coordination environment change of used CoN3C/rGO. (a) Normalized Co k-edge XANES of CoN3C/rGO, used CoN3C/rGO and reference samples. (b) Fourier transform EXAFS of used CoN3C/rGO, CoN3C/rGO and reference samples. (c) EXAFS fitting analysis of used CoN3C/rGO in R space. (d) EXAFS fitting

analysis of used CoN_3C/rGO in K space.

Table R1. The exact Co-loading content of catalysts.

Catalysts	CoN_3C/rGO	CoN_3C	Used CoN_3C/rGO
Co content %	1.25	1.97	0.80

Table R2. The elements content of catalysts from XPS.

Elements / Catalysts	Co	N	C	O
CoN_3C/rGO	0.44	19.09	71.08	9.4
CoN_3C	0.41	30.96	61.34	7.3
rGO	/	/	88.01	12
Used CoN_3C/rGO	0.26	14.06	79.37	6.32

Comment 8. More experiments, such as the screening experiment, should be provided to identify the dominant active sites.

Response: We appreciate your professional and valuable suggestion. In the revised manuscript, we added the result of screening experiment by using the KSCN as the screening agent and investigated the core role of Co as the active site. As clearly shown in **Figure R6**, the presence of KSCN inhibit the BPA degradation completely, indicating as the active site, Co atom plays the main role in the activation of PMS. And **Figure R6** were added into the revised **Supplementary information** as **Supplementary Fig. 40** to clarify our conclusion. Detailed discussion in the revised **manuscript** can be found below (**Line 232-234, Page 5**):

The presence of KSCN, a representative cobalt complexing agent, inhibit the BPA degradation completely, indicating Co atom is the active site in the activation of PMS.

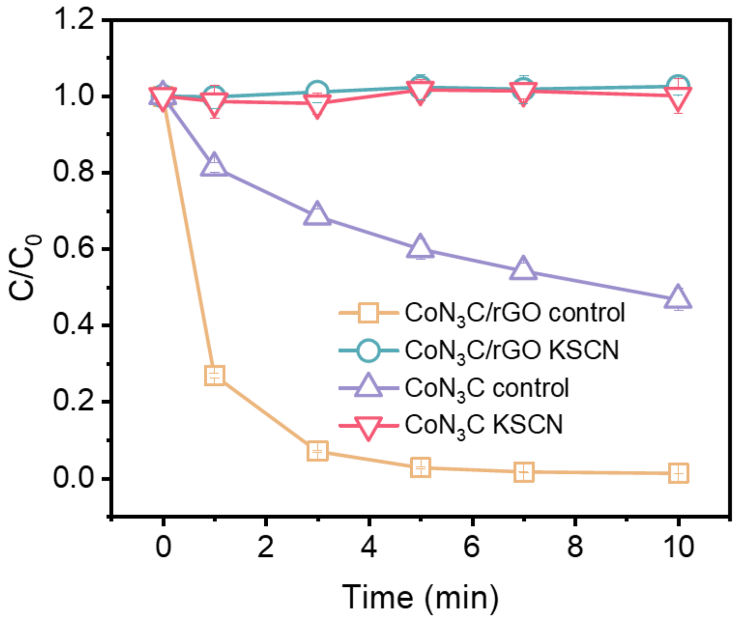

Figure R6. The investigation of active site. The BPA degradation curves under KSCN added to the CoN_3C/rGO/PMS and CoN_3C/PMS system.

Reviewer #2:

The manuscript addresses the critical challenge of precisely regulating the direct electron transfer (ETP) pathway during peroxymonosulfate (PMS) activation. A "nano-island" Co single-atom catalyst (CoN_3C/rGO) is designed and synthesized, where carbon nitride serves as "islands" and reduced graphene oxide (rGO) as the "sea". This synergistic "island-sea" structure achieves the simultaneous enhancement of activity and selectivity in the Fenton-like system. Focusing on the core demand of advanced oxidation water treatment technology, the study features an innovative design concept and a comprehensive experimental setup. It combines multiple characterization techniques (XRD, TEM/HAADF-STEM, XAFS, XPS etc.), performance evaluations, and density functional theory (DFT) calculations to thoroughly elucidate the molecular mechanism underlying the regulation of the ETP pathway by the "island-sea" structure from both experimental and theoretical perspectives. The research outcomes exhibit remarkable advantages in catalytic activity, selectivity, stability, and practical application potential, providing crucial theoretical support and technical references for the directional regulation of reactive oxygen species (ROS) in advanced oxidation processes (AOPs). This work holds significant academic value and promising application prospects. My comments are as follows:

Response: We are truly grateful for your positive and insightful evaluation of our manuscript. All the specific points and suggestions raised have been addressed in detail below. We believe the manuscript has been strengthened as a result. And we sincerely appreciate your time and support.

Comment 1. The manuscript reports the ETP pathway contribution rate as "approximately 94%" and "as high as ~92.5%". Although this discrepancy may stem from different calculation methods or experimental conditions, the underlying reasons are not clearly explained, which may confuse readers. It is recommended to explicitly clarify the calculation methods for the two sets of data (e.g., indirect calculation based on quenching experiments vs. direct calculation via electrochemical tests) and verify

data reliability through repeated experiments. If the difference arises from varying experimental conditions, detailed information on how specific parameters (e.g., catalyst concentration, PMS concentration) affect the results should be provided, and the core data representation should be standardized.

Response: Thank you for your meticulous review. We thank you for your careful review. We acknowledge that this discrepancy resulted from an oversight during our final manuscript check. The corrected contribution rate of the ETP pathway is 94%, not 92.5%. Specific calculation method has been transferred from Supplementary Information to manuscript. We sincerely apologize for this error. Detailed information added to the revised **manuscript** can be found below (**Line 451-455, Page 10**):

The contribution of ·OH and the direct electron transfer pathway were obtained by the following equations:

$$R_{\cdot OH}=\frac{k_{\cdot OH,\ BPA}[\cdot OH]_{ss}}{k_{obs,\ BPA}} \quad (4)$$

$$R_{ETP}=1-R_{\cdot OH} \quad (5)$$

The $k_{\cdot OH,\ BPA}$ *is the second-order reaction rate constants between ·OH and BPA* $(6.9\times10^{9}\ M^{-1}\ s^{-1})$.

Comment 2. The influence of carbon nitride "island" size (e.g., particle size variation under different hydrothermal conditions) and rGO "sea" loading on catalyst structure and performance has not been investigated. This makes it impossible to determine the optimal parameter range of the "island-sea" structure or quantify the contributions of "island" size and "sea" loading to electron transfer efficiency and orbital regulation efficacy. It is suggested to supplement experiments on the optimization of "island-sea" structural parameters and carrier comparison: Prepare carbon nitride "islands" of different sizes by adjusting hydrothermal temperature, reaction time, and other parameters; characterize particle size distribution via TEM; and identify the optimal "island" size range through catalytic performance tests. Investigate the effect of rGO loading (e.g., 0.05 g, 0.1 g, 0.2 g) on electronic structure (XPS valence band, UPS) and catalytic performance.

Response: Thank you for your valuable suggestions. We fully agree that the dimensions

of the CoN_3C "islands" and the loading of the rGO "sea" are critical to the electronic regulation and catalytic efficacy. Accordingly, we have supplemented systematic experiments to investigate the influence of hydrothermal temperature (160 °C, 180 °C, and 200 °C) and rGO loading (0.05 g, 0.1 g, and 0.15 g) on the catalyst structure and performance. As shown in the newly added **Figure R7** (as **Supplementary Fig. 21** added to the revised **Supplementary information**), catalysts synthesized at 0.05g rGO, 0.15g rGO, 160 °C and 200 °C both achieved 100% BPA removal within 10 minutes, with some samples even exhibiting a higher k_{obs} than the 0.1g rGO/180 °C sample. While the specific yield of the composite catalyst at 0.05 g rGO loading is relatively low, which constrains its potential for large-scale production. And 0.15 g rGO provides high conductivity, it involves excessive raw material consumption while no significant reactivity enhancement. Moreover, stability tests revealed that the samples synthesized at other conditions suffered from significant performance decay, with the BPA removal rate dropping below 90% after only 2-4 cycles (**Figure R8**, as **Supplementary Fig. 22** added to the revised **Supplementary information**). In contrast, the optimized catalyst maintained superior stability even at 5 cycles. We attribute this to the fact that 0.1g rGO and 180 °C provides the optimal balance of crystallinity and anchoring strength for the CoN_3C islands on the rGO surface. Under other conditions, the islands may not be fully stabilized, leading to leaching or structural collapse during recycling. To further analyze the effect of synthesis condition to morphology and electronic structure, TEM and XPS were conducted (**Figures R9-R11**, as **Supplementary Figs. 23-25** added to the revised **Supplementary information**). It is worth noting that due to the amorphous nature of the CoN_3C phase and the flexible structure of rGO, no drastic morphological differences were observed across these optimization ranges. This indicates that our "island-sea" assembly strategy is robust and capable of forming uniform heterostructures under a relatively wide range of synthesis conditions. From the XPS spectra, the optimal catalyst (CoN_3C/rGO, 0.1g rGO/180 °C) exhibits the highest proportion of pyrrolic N (47.47%) compared to the samples synthesized at 0.05g rGO (28.87%), 0.15g rGO (29.75%), 160 °C (30.11%), and 200 °C (28.84%). Since pyrrolic

N sites act as strong anchors for Co atoms within the carbon defects, their high abundance at 180 °C signifies the formation of the most robust coordination structure. In contrast, the lower pyrrolic N content at other conditions leads to weaker metal-support interactions. Therefore, the maximization of pyrrolic N directly correlates with the superior structural stability observed in the optimal sample. Additionally, the optimized catalyst exhibits the lowest Co 2p binding energy (~780.06 eV) compared to other samples, indicating a higher electron density on the Co atoms, which can facilitate the electron transfer.

We acknowledge that the current optimization is a preliminary exploration into the complex "island-sea" assembly. While we have successfully identified the optimal synthesis window to balance activity and stability, we recognize that the precise control over amorphous nanomaterials remains a challenge. In our future work, we will focus on developing more advanced synthesis strategies to achieve finer regulation and deeper insight of the island size and distribution.

Related information added to the revised **manuscript** (**Line 164-168, Page 4**):

Systematic optimization of the hydrothermal conditions and rGO loading revealed that the CoN_3C/rGO catalyst prepared at 180 °C with 0.1 g rGO exhibits optimal PMS activation performance (Supplementary Figs. 21-27). This optimal synthesis window maximizes the formation of electron-rich pyrrolic N defects and creates the most electron-donating Co centers, as evidenced by XPS, which underpins its superior activity and stability.

Other detailed information added to the revised **Supplementary information** (**Pages 25-26**):

To construct the optimal "island-sea" architecture for efficient electron transfer, we systematically investigated the influence of synthesis parameters, specifically the hydrothermal temperature (160 °C, 200 °C) and rGO loading (0.05 g, 0.15 g), on the catalyst structure and PMS activation performance (Supplementary Figs. 21-22). Although all synthesized composites exhibited comparable initial degradation efficiencies, the sample prepared at 180 °C with 0.1 g rGO was identified as the optimal

candidate, striking the best balance between cyclic stability, atom economy, and catalytic activity. It is worth noting that due to the amorphous nature of the CoN_3C phase and the flexible structure of rGO, no drastic morphological differences were observed across these optimization ranges from TEM results (Supplementary Figs. 23). And the uniform distribution of C, N, O, Co can be obtained by EDS-mapping (Supplementary Figs. 24). *This indicates that our "island-sea" assembly strategy is robust and capable of forming uniform heterostructures under a relatively wide range of synthesis conditions. Crucially, XPS characterization uncovers the electronic origin of this superiority: the optimal sample possesses the highest content of electron-donating pyrrolic N species, which not only serves as a robust anchor to prevent metal leaching (explaining the superior stability) but also induces an electron-rich state in Co centers (evidenced by the lowest Co 2p binding energy), thereby optimizing the electronic configuration of Co active sites (Supplementary Figs. 25).*

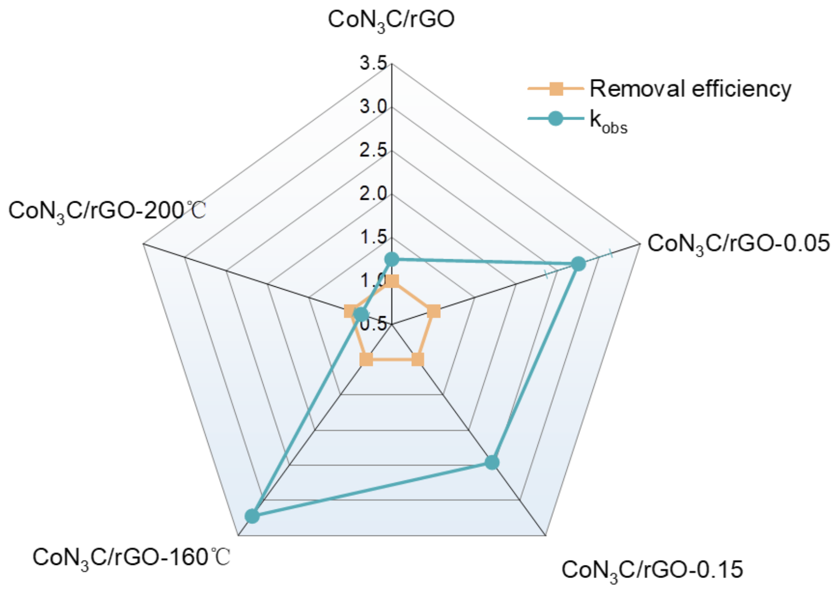

Figure R7. The catalytic performance of catalysts with different synthesis condition. The BPA degradation efficiency and k_{obs} with catalysts with different synthesis condition mediated PMS systems.

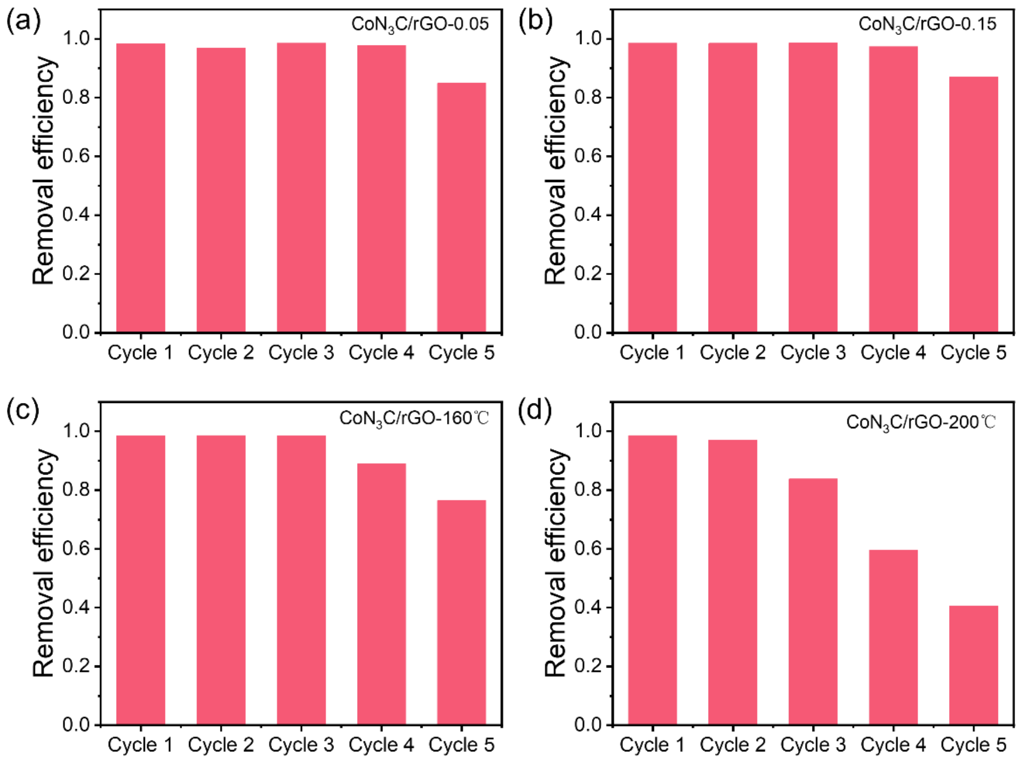

Figure R8. The stability of catalysts with different synthesis condition. The cyclic performance for BPA removal of (a) CoN_3C/rGO-0.05g, (b) CoN_3C/rGO-0.15g, (c) CoN_3C/rGO-160 °C, (d) CoN_3C/rGO-200 °C.

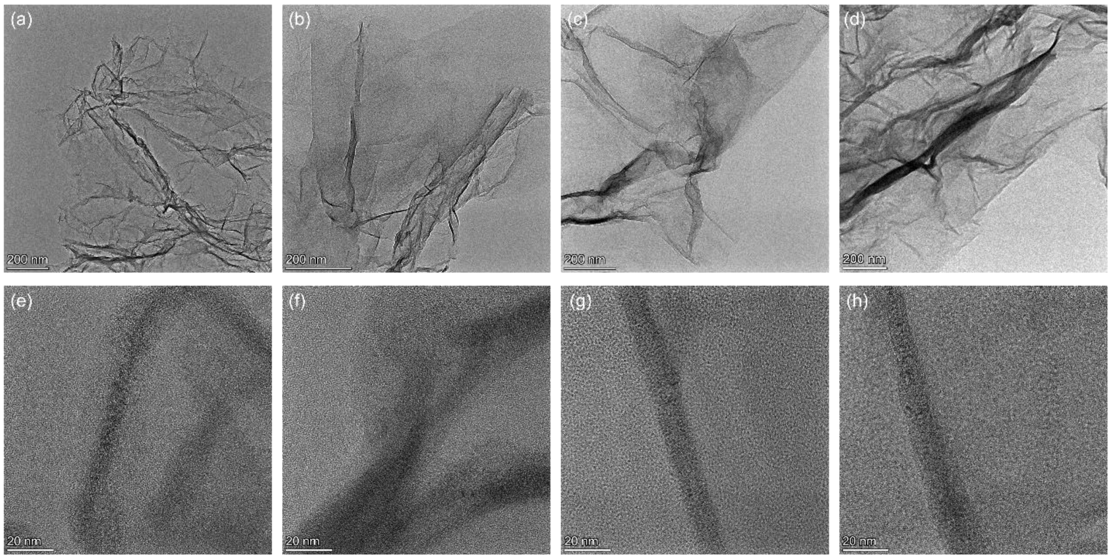

Figure R9. The morphologies of catalysts with different synthesis condition. The

TEM images of (a) CoN_3C/rGO-0.05g, (b) CoN_3C/rGO-0.15g, (c) CoN_3C/rGO-160 °C, (d) CoN_3C/rGO-200 °C; the HRTEM images of (e) CoN_3C/rGO-0.05g, (f) CoN_3C/rGO-0.15g, (g) CoN_3C/rGO-160 °C, (h) CoN_3C/rGO-200 °C.

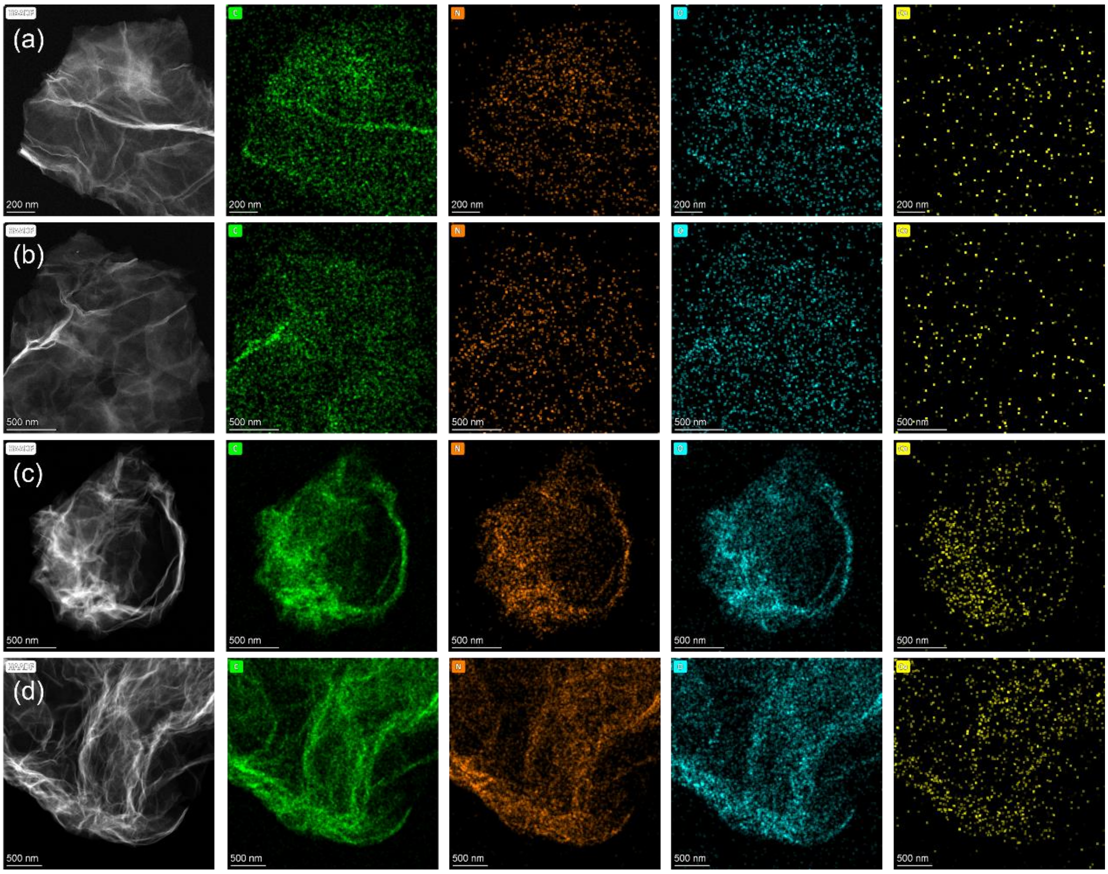

Figure R10. The elements distribution of catalysts with different synthesis condition. The EDS mapping images of (a) CoN_3C/rGO-0.05g, (b) CoN_3C/rGO-0.15g, (c) CoN_3C/rGO-160 °C, (d) CoN_3C/rGO-200 °C.

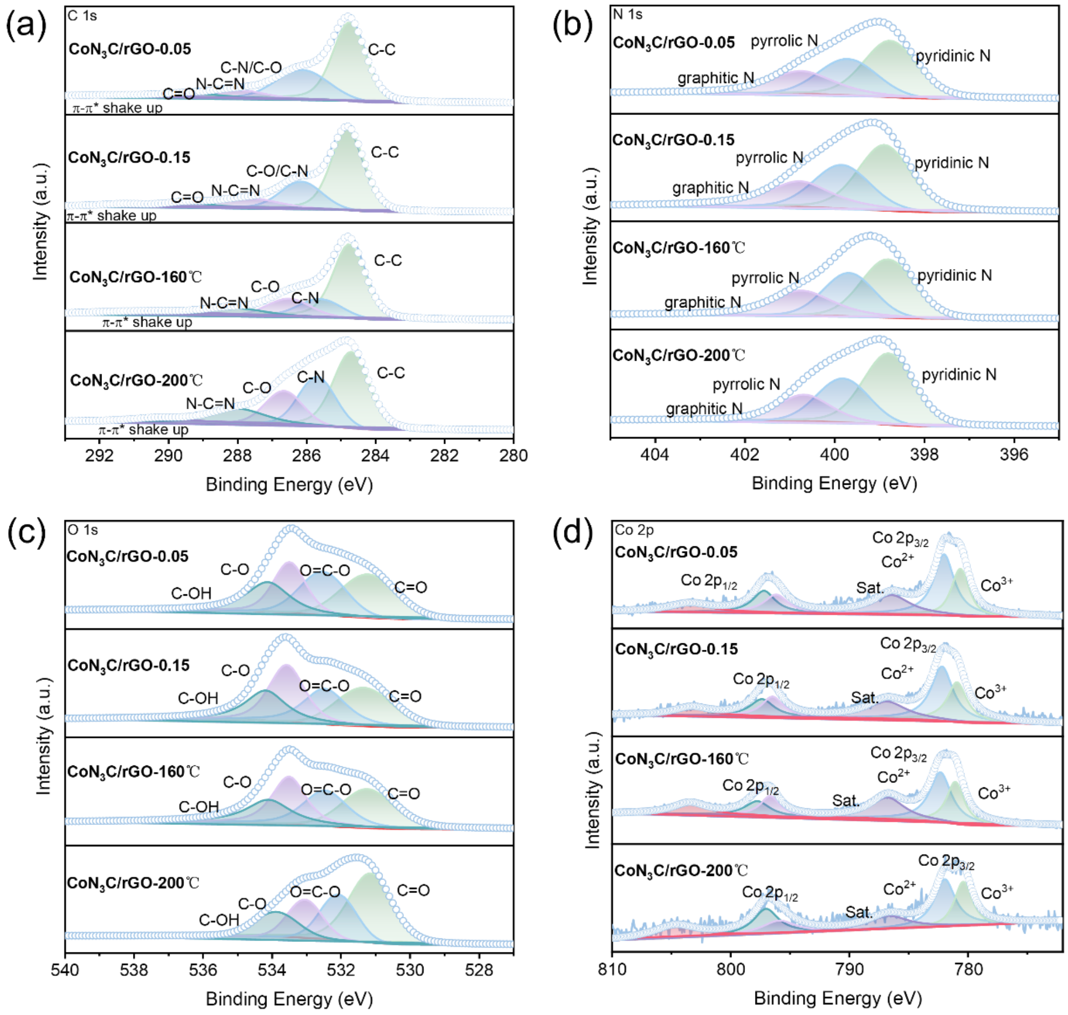

Figure R11. The chemical state of elements on catalysts with different synthesis condition. The high-resolution XPS (a) C 1s, (b) N 1s, (c) O 1s, (d) Co 2p spectra of CoN_3C/rGO-0.05g, CoN_3C/rGO-0.15g, CoN_3C/rGO-160 °C, and CoN_3C/rGO-200 °C.

Comment 3. The influence of different "sea" carriers (e.g., carbon nanotubes, porous carbon) on the electronic structure and catalytic performance of CoN_3C has not been compared. This fails to fully demonstrate the unique advantages of rGO as the "sea" carrier and weakens the argument for the universality of the "island-sea" structural design strategy. It is recommended to select carbon nanotubes and porous carbon as alternative "sea" carriers, synthesize CoN_3C/carbon nanotube and CoN_3C/porous carbon catalysts, and compare their activity and selectivity in PMS activation and BPA degradation.

Response: Thank you for your valuable and professional comment. We agree with your advice that the influence of different sea carriers should be compared. Hence, we synthesized two other catalysts, which utilized the active carbon (CoN_3C/AC) and carbon nanotubes (CoN_3C/CNTs) as the "sea", respectively. These two materials exhibited different activity in BPA removal. As shown in **Figure R12** (as the **Supplementary Fig. 26** in the revised **Supplementary information**), For CoN_3C/CNTs, its k_{obs} is about 2 times lower than CoN_3C/rGO even though it also can degrade BPA within 10 mins. While the CoN_3C/AC nearly no PMS activation effect. The XPS was conducted to complete an initial analysis of the reasons (**Figure R13,** which has been as the **Supplementary Fig. 27** added to the revised **Supplementary information**). The Co $2p_{3/2}$ and $2p_{1/2}$ binding energy of CoN_3C/rGO (~781.0 eV and 796.5 eV) is lower than those of CoN_3C/AC (~781.5 eV and 797.0 eV) and CoN_3C/CNTs (~781.6 eV and 796.9 eV). This suggests that the rGO network provides the most effective electron injection into the Co active centers, optimizing their electronic configuration for PMS activation. Additionally, the appearance of the N-C=N peak at 287.4 eV in the CoN_3C/rGO is the result of a significant negative binding energy shift (~0.9 eV), which is induced by the strong electron-donating effect of the 2D rGO network, effectively separates the N-C=N signal from the dominant carbon background. In the AC and CNTs systems, the interfacial electronic coupling is likely too weak to induce such a substantial shift, causing the N-C=N signal to remain overlapped with or masked by the C-N/C-O peaks (~285.8 and 286.3 eV) (please see *ChemPhotoChem 9, e202400343 (2025)*). Given the complexity of carrier-guest interactions, we plan to conduct more in-depth investigations, such as density functional theory (DFT) simulations on different carbon curvatures and experimental X-ray absorption fine structure (XAFS) comparisons in our future work. Detailed information added to the revised **Supplementary information** can be found below (**Page 31**):

To verify the universality of the "island-sea" strategy, CoN_3C islands were also anchored on activated carbon (AC) and carbon nanotubes (CNTs). As illustrated in Supplementary Fig. 26, the catalytic activity follows the order of CoN_3C/rGO >

CoN_3C/CNTs > CoN_3C/AC. Compared with CoN_3C, XPS analysis reveals that CoN_3C/rGO possesses the minimal shift of Co 2p binding energy, indicating its highest electron density. Notably, the characteristic N-C=N skeleton signal was not resolved in the C 1s spectra of CoN_3C/AC and CoN_3C/CNTs. This is likely due to the insufficient electronic coupling between these supports and the CoN_3C islands, suggesting the interfacial electronic coupling is likely too weak to induce such a substantial shift, causing the N-C=N signal to remain overlapped with or masked by the C-N/C-O peaks (~285.8 and 286.3 eV). Consequently, the Co centers in CoN_3C/rGO maintain a more optimized, electron-rich environment, which is highly conducive to the fastest kinetics PMS activation. This preliminary exploration suggests that the structural and electronic properties of the "sea" carrier are pivotal to the performance.

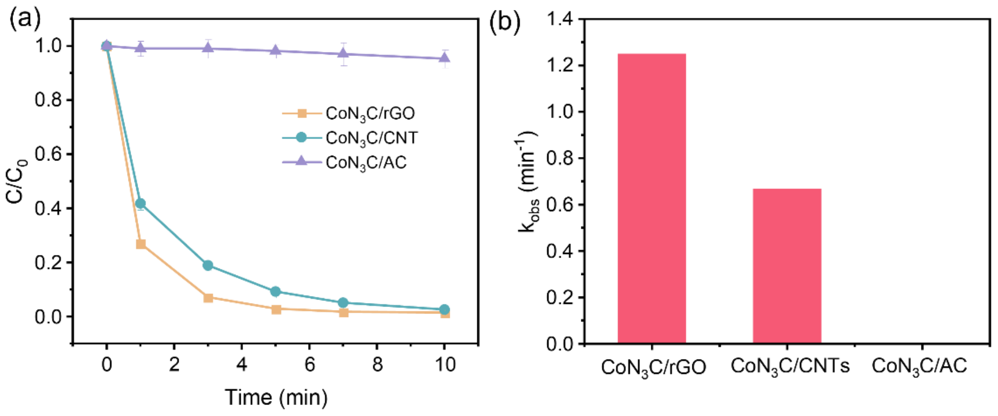

Figrue R12. Effect of different sea carrieres. (a) The degradation curves, (b) k_{obs} of BPA in CoN_3C/rGO/PMS, CoN_3C/AC/PMS and CoN_3C/CNTs/PMS. (Experiment conditions: [catalyst]= 0.050 g/L, [PMS]= 0.5 mM, [BPA]= 10 mg/L, pH= 6.0, room temperature if not otherwise specified)

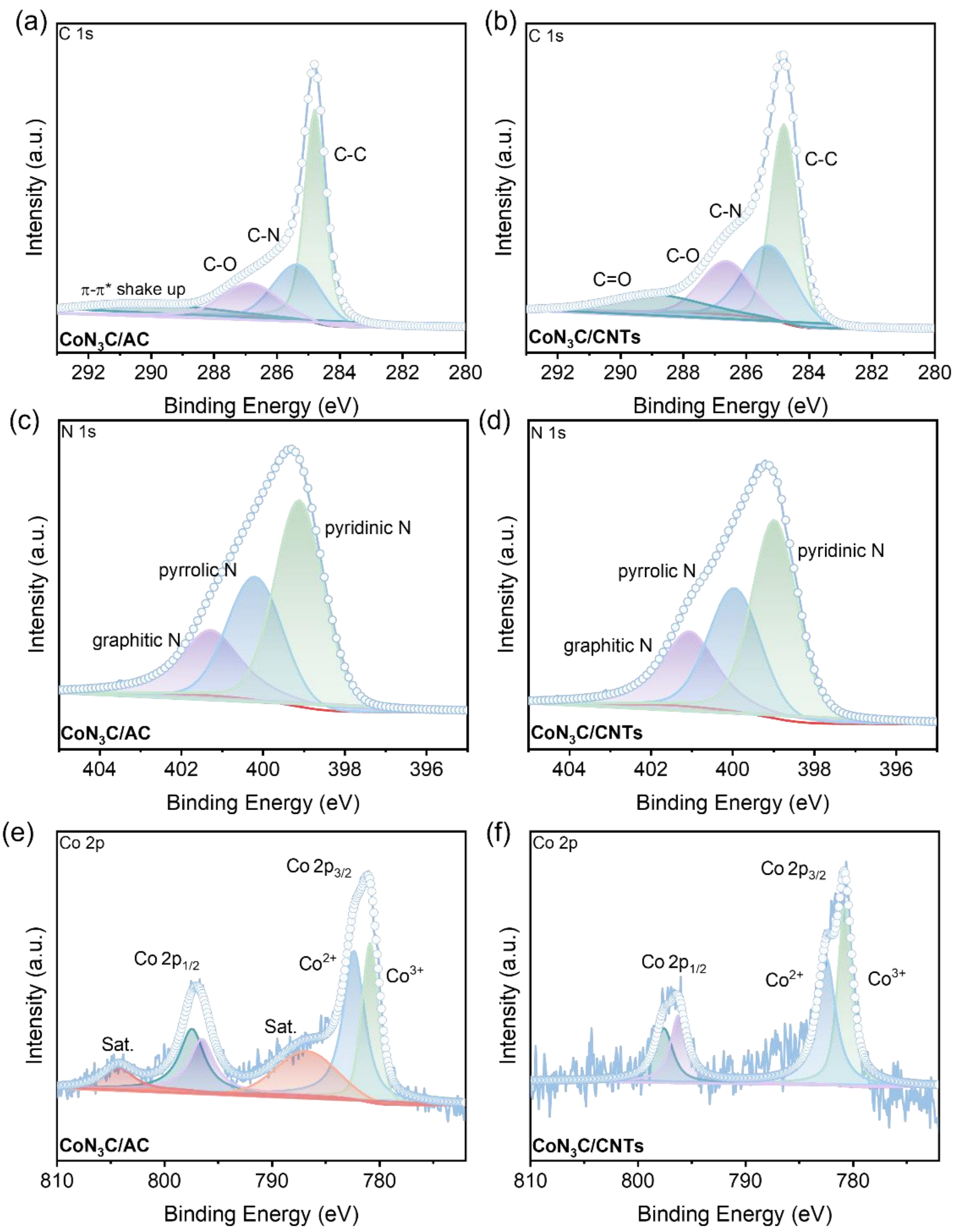

Figrue R13. XPS characterization of catalysts with different sea carrieres. High-resolution XPS spectra of C 1s for (a) CoN_3C/AC, (b) CoN_3C/CNTs; N1s for (c) CoN_3C/AC, (d) CoN_3C/CNTs; Co 2p for (e) CoN_3C/AC, (f) CoN_3C/CNTs.

Comment 4. The continuous-flow experiment only reports 36-hour operation results. Long-term stability data (e.g., over 100 hours) are lacking, which is insufficient to verify the wear resistance, active site retention rate, and long-term leaching risk of the catalyst membrane coating. Supplementary long-term stability tests are recommended.

Response: Thank you for your valuable and professional suggestions. To supplementary the long-term stability data, we conducted a longer continuous flow test. The entire running time achieved 100 h. During the whole process, the CoN_3C/rGO/PVDF catalytic membrane exhibited the outstanding activity and stability under three kinds of water matrix (ultrapure water, groundwater, and surface water) (**Figure R14**, as **Fig. 5f** in the revised **manuscript**). The results show that BPA in three water matrices could be almost completely removed during the entire 100-hour operation period with the stable water flux. Moreover, Co leaching concentration remained at an eco-friendly level (< 1 mg/L) in the whole process. The related information has been added to the revised **manuscript** as below (**Line 357-361, Page 8**):

Ultrapure water, groundwater, and surface water were used as typical water matrices to be treated by a continuous flow reactor at a flow rate of 1.0 mL/min (Fig. 5f). The results show that BPA in three water matrices could be removed > 90% during the entire 100-hour operation period with stable water flux. Moreover, Co leaching concentration remained at an eco-friendly level (< 1 mg/L) in the whole process.

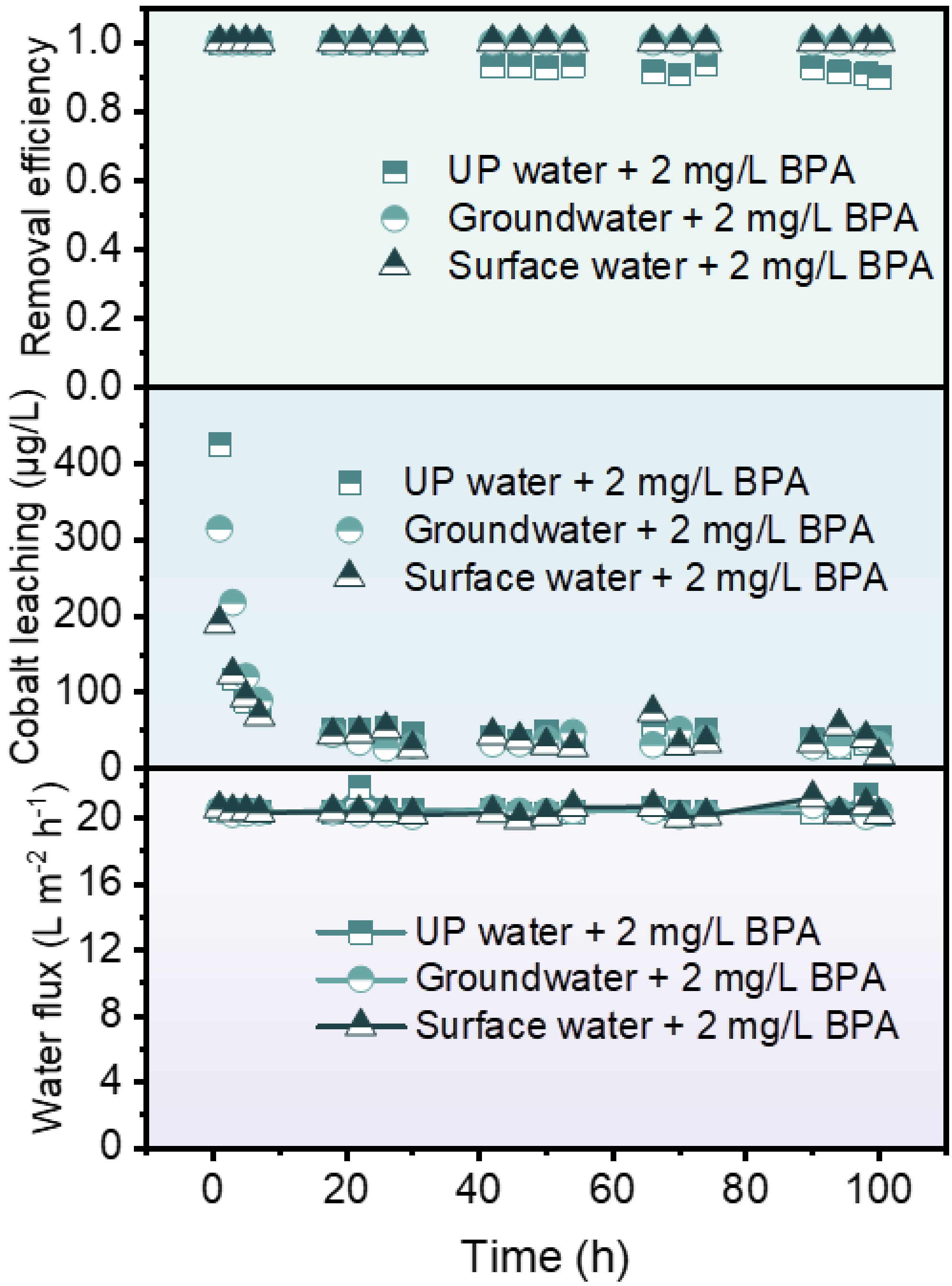

Figrue R14. The continuous flow results. BPA removal performance in CoN_3C/rGO/PVDF system under different water matrices.

Comment 5. The manuscript does not specify the PMS dosing method (e.g., one-time addition vs. continuous supplementation) or the influence of water flow rate on degradation efficiency in the continuous-flow system, nor does it provide membrane

flux data. This hinders future engineering scaling. It is suggested to supplement relevant information.

Response: Thank you for your valuable suggestions. I apologize for our neglect in this matter. The one-time addition method was conducted within the whole continuous-flow process. Moreover, water flow information was added in the revised **manuscript** and **Figure R14** (as **Fig. 5f** in the revised **manuscript**). The related description in the revised **manuscript** can be seen as below (**Line 357-361, Page 8**):

Ultrapure water, groundwater, and surface water were used as typical water matrices to be treated by a continuous flow reactor at a flow rate of 1.0 mL/min (Fig. 5f). The results show that BPA in three water matrices could be removed > 90% during the entire 100-hour operation period with stable water flux. Moreover, Co leaching concentration remained at an eco-friendly level (< 1 mg/L) in the whole process.

Comment 6. In the XAFS analysis, although the Co-N3 coordination configuration is confirmed by EXAFS fitting, the original k-space data and fitting residual plots are not provided, making it difficult to evaluate fitting quality. For the XANES spectra, the Co valence state is reported to be between +2 and +3, but linear combination fitting has not been used to quantitatively analyze the Co^{2+}/Co^{3+} ratio or the effect of rGO introduction on Co valence. It is recommended to: Supplement the original k-space XAFS data and fitting residual plots. Perform LCF on XANES spectra to quantify the Co^{2+}/Co^{3+} ratio and clarify the influence of rGO on Co valence state.

Response: Thank you for your valuable and professional suggestions. We apologize for our neglect in this matter and we agree with the necessity of k space data. Actually, we have shown the original k-space data and fitting residual plots in **Supplementary Fig. 17.** Here, we once again present this figure to you (**Figure R15**). To analyze the Co^{2+}/Co^{3+} ratio of CoN_3C/rGO and CoN_3C, the LCF was conducted and we have added the **Figure R16** to the **Supplementary information** as the **Supplementary Fig. 15**. According to the results, the Co^{2+}/Co^{3+} ratio of CoN_3C/rGO is 0.55/0.45, and 0.56/0.44 for CoN_3C, indicating that the introduction of the rGO sea did not drastically alter the average oxidation state of Co. This suggests that the significant performance

enhancement is not primarily due to a shift in Co valence. Instead, the key role of rGO is to modulate the local electronic structure and orbital occupation of the Co sites, as evidenced by the upshifted d-band center and the sharpened d_{z2} orbital states near the Fermi level revealed by DFT calculations. This orbital-level modulation underpins the simultaneous improvement in activity and selectivity. Detailed discussion in the revised **manuscript** can be found below (**Line 145-148, Page 3**):

The specific valence state is shown in linear combination fitting results, which the proportion of Co^{2+}/Co^{3+} of CoN_3C/rGO (0.55/0.45) and CoN_3C (0.56/0.44) is similar, indicating that the introduction of the rGO sea did not drastically alter the average oxidation state of Co (Supplementary Fig. 15).

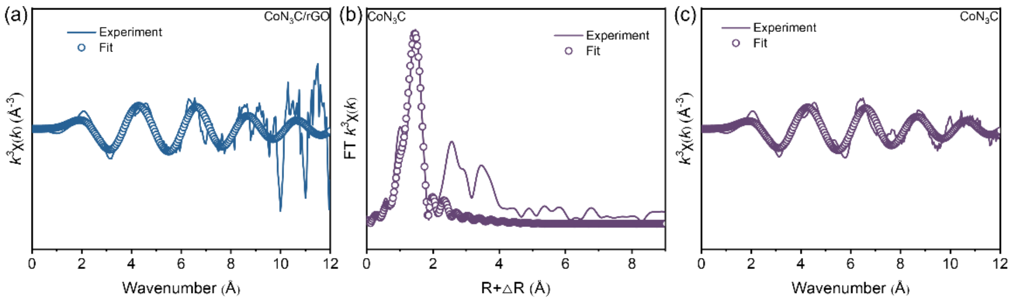

Figrue R15. EXAFS characterization of catalysts. (a) EXAFS fitting analysis of CoN_3C/rGO in K space. EXAFS fitting analysis of CoN_3C in (b) R space and (c) K space.

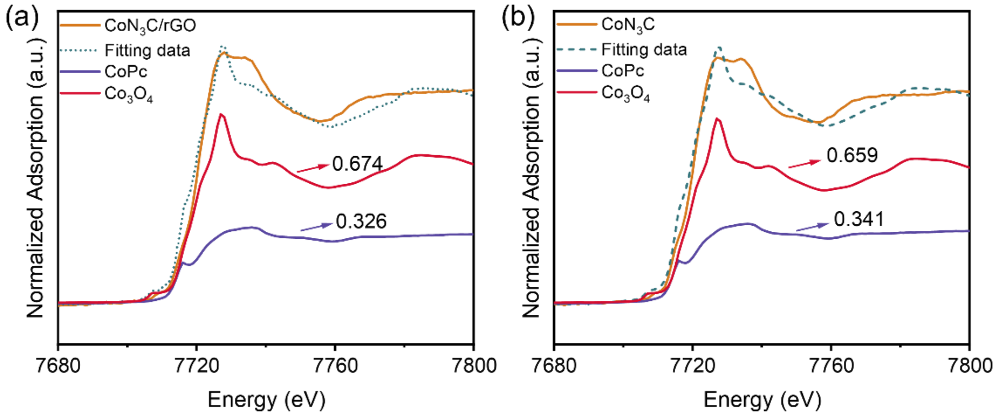

Figrue R16. XANES LCF results. Linear combination fitting curves of (a) Co K-edge of CoN_3C/rGO. (b) Co K-edge of CoN_3C.

Comment 7. In the DFT calculations, it is recommended to clarify whether solvent effects on PMS adsorption energy and electron transfer efficiency are considered. The Gibbs free energy barrier for the ETP process has not been calculated, which prevents further verification of the "island-sea" structure's promotional effect on the ETP pathway from a thermodynamic perspective. It is suggested to: Supplement the calculation of the Gibbs free energy barrier for the ETP process to verify the "island-sea" structure's thermodynamic advantage. Provide charge density difference maps and orbital overlap integral data to more intuitively demonstrate the directional hybridization between the Co dz2 orbital and the PMS p orbital.

Response: We sincerely appreciate the reviewer's professional advice regarding the DFT calculations and the mechanism verification. Firstly, we confirm that all DFT calculations were performed in the vacuum condition to isolate the intrinsic electronic modulation induced by the rGO substrate, and we added this point to the manuscript. In the process of mechanistic exploration in catalysis, employing a vacuum model to understand the fundamental electronic processes and orbital interactions at surfaces is a common and acceptable practice in computational catalysis. Many mechanistic studies on single-atom catalysts for PMS activation have also adopted similar simplifications, successfully revealing the core mechanisms (please see: *Nat. Commun. 16, 2376 (2025); Nat. Commun. 16, 115 (2025)*).

We calculated the Gibbs free energy profiles for PMS activation on both CoN_3C and CoN_3C/rGO to compare the difference of various pathway from the thermodynamic insight. As shown in **Figure R17** (as the **Fig. 4b** in the revised **manuscript** and **Supplementary Fig. 49** in the revised **Supplementary information**), the formation of ·OH is thermodynamically favorable for both systems, which explains why trace ROS were detected in both cases experimentally. However, a critical difference lies in the adsorption energy (-2.659 eV of CoN_3C vs. -1.863 eV of CoN_3C/rGO), which is also confirmed by the first step in the Gibbs free energy results. For the CoN_3C, the overly strong interaction suggests an aggressive activation of the PMS molecule. This intense adsorption likely induces excessive electron back-donation into the O–O

antibonding orbitals, causing the cleavage of the peroxide bond to generate radicals. This aligns with the experimental observation that the ROS pathway dominates (>89%) in CoN_3C/PMS. In contrast, the moderated adsorption strength on CoN_3C/rGO is pivotal for the mechanism switch. The weaker binding prevents the immediate break of the O–O bond, preserving the PMS molecule in a metastable activated state. Coupled with the high conductivity of the rGO network, this configuration facilitates a rapid ETP process, rather than chemical bond breaking. Furthermore, the weaker adsorption favors the timely desorption of reduced species, preventing surface poisoning and accelerating the catalytic turnover.

To further demonstrate the directional hybridization, we calculated the spatial distribution of the Co 3d orbitals by wave function (**Figures R18-R21**, as **Fig. 4e** in the revised **manuscript** and **Supplementary Figs. 57-59** in the revised **Supplementary information**). For the d_{z2} orbital electron of CoN_3C, the electron cloud exhibits a diffuse and radially expanded character, indicating a strong delocalization of electron density towards the surrounding planar ligands. In contrast, upon introducing the rGO sea (CoN_3C/rGO), the d_{z2} orbital becomes significantly contracted in the radial direction and elongated along the z-axis. This morphological transformation suggests that the “island-sea” interaction suppresses the random hybridization of d_{z2} orbital electron and re-orients the electron density to be highly concentrated perpendicular to the catalyst plane.

Furthermore, the DFT results are further corroborated by the Ultraviolet Photoelectron Spectroscopy (UPS) measurements. As shown in **Figure R22** (as the **Fig. 4h** in the revised **manuscript**), a narrower peak with higher intensity near the Fermi level appears at CoN_3C/rGO sample compared with CoN_3C, explaining the DOS results. Additionally, the valence band maximum (VBM) position of CoN_3C/rGO (0.46 eV) is closer to Fermi level than CoN_3C (0.66 eV), correlated with higher d band center of CoN_3C/rGO. This electronic reconfiguration enhances the orbital hybridization between the Co active sites and PMS molecules, thereby kinetically accelerating the ETP.

The related information added to the revised **manuscript** can be found as below (**Line 275-280, Page 6; Line 306-311, Page 7; Line 321-324, Page 7**):

Combined with thermodynamic analysis provided by Gibbs free energy calculations that while radical generation is thermodynamically feasible for both systems, the dominant reaction pathway is different. For CoN_3C*, the diffuse orbital results in an overly strong adsorption, inducing excessive O–O bond stretching and breaking (radical pathway) (Fig. 4b). Conversely,* CoN_3C*/rGO features an optimized, moderate adsorption energy, which preserves the PMS molecular integrity (Supplementary Fig. 49)*[32, 33].

To visualize this difference, wave function plots reveals that the Co d_{z2} *orbital in* CoN_3C*/rGO exhibits a radially contracted and axially elongated shape, which maximizes the directional orbital overlap with the axial PMS ligand, suppressing random hybridization (Fig. 4e, f and Supplementary Figs. 57-59). While the* CoN_3C *displays a diffuse and radially expanded electron cloud, limiting the effective interfacial coupling. This suggests that the "island-sea" architecture facilitates the formation of a more robust catalyst-PMS* complex favoring the ETP.*

The above results were also confirmed by Ultraviolet Photoelectron Spectroscopy (UPS) (Fig. 4h). Compared with CoN_3C*, the occupied states near the* E_F *become narrower and more significant for* CoN_3C*/rGO. And the upshift of the valence band maximum (VBM) towards the* E_F *suggests an elevation of the d-band center (0.46 eV for* CoN_3C*/rGO vs. 0.66 eV for* CoN_3C*).*

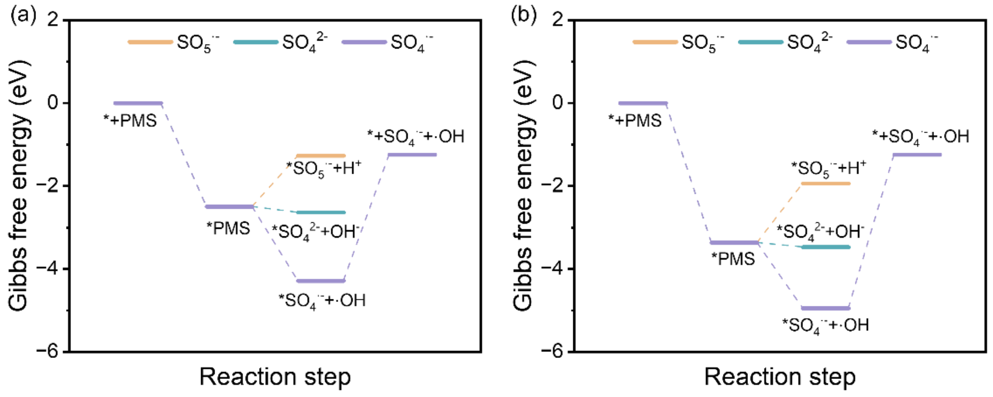

Figrue R17. The thermodynamic trends of reaction between catalysts and PMS. The Gibbs free energy change of vairous pathway in (a) CoN_3C/rGO/PMS, (b) CoN_3C/PMS.

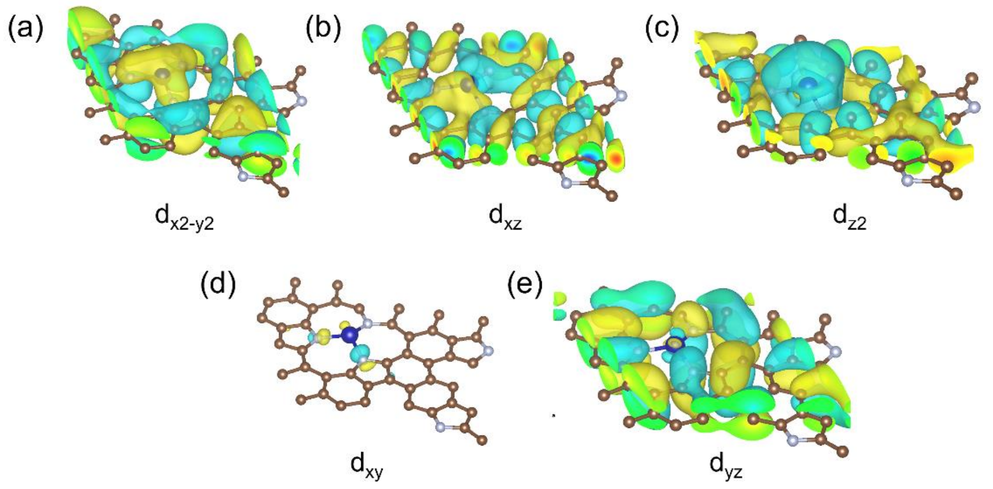

Figrue R18. The d electron cloud distribution of CoN_3C/rGO. The wave function of (a) d_{x2-y2}, (b) d_{xz}, (c) d_{z2}, (d) d_{xy}, (e) d_{yz} in CoN_3C/rGO.

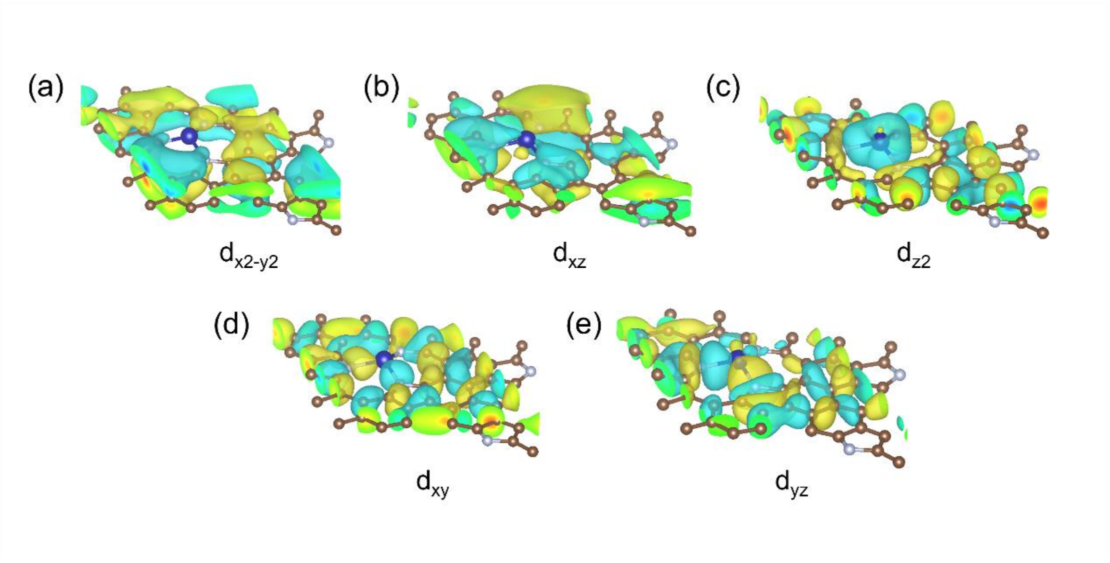

Figrue R19. The d electron cloud distribution of CoN$_3$C. The wave function of (a) d_{x2-y2}, (b) d_{xz}, (c) d_{z2}, (d) d_{xy}, (e) d_{yz} in CoN$_3$C.

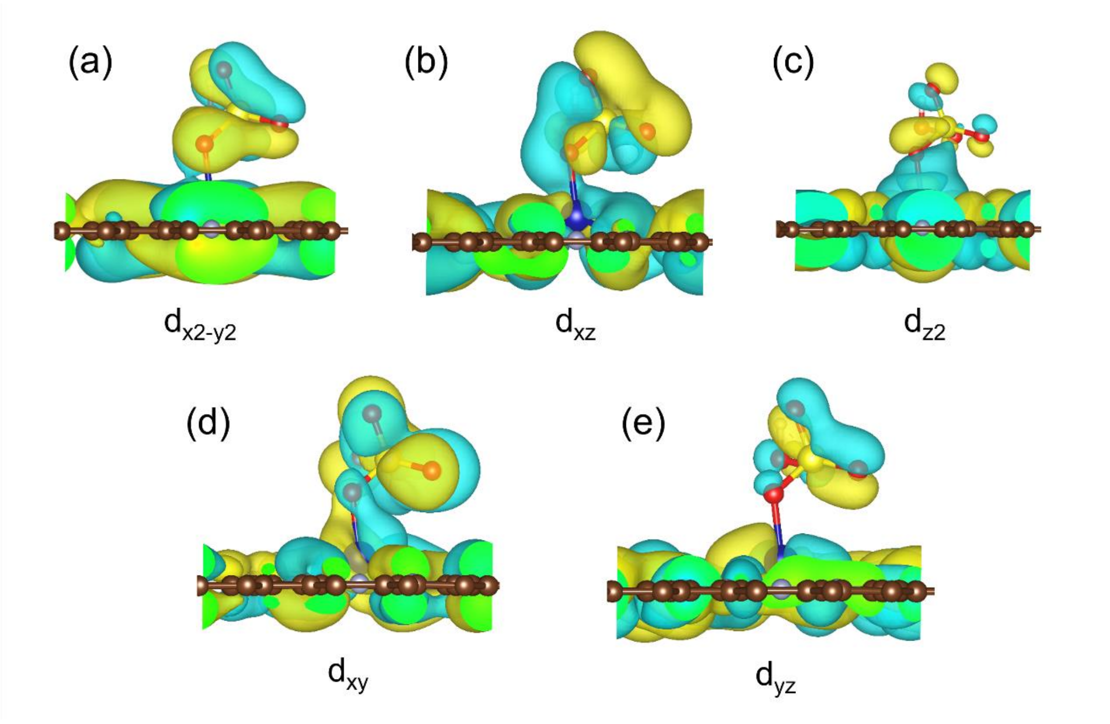

Figrue R20. The d electron cloud distribution of CoN$_3$C/rGO-PMS. The wave function of (a) d_{x2-y2}, (b) d_{xz}, (c) d_{z2}, (d) d_{xy}, (e) d_{yz} in CoN$_3$C/rGO-PMS.

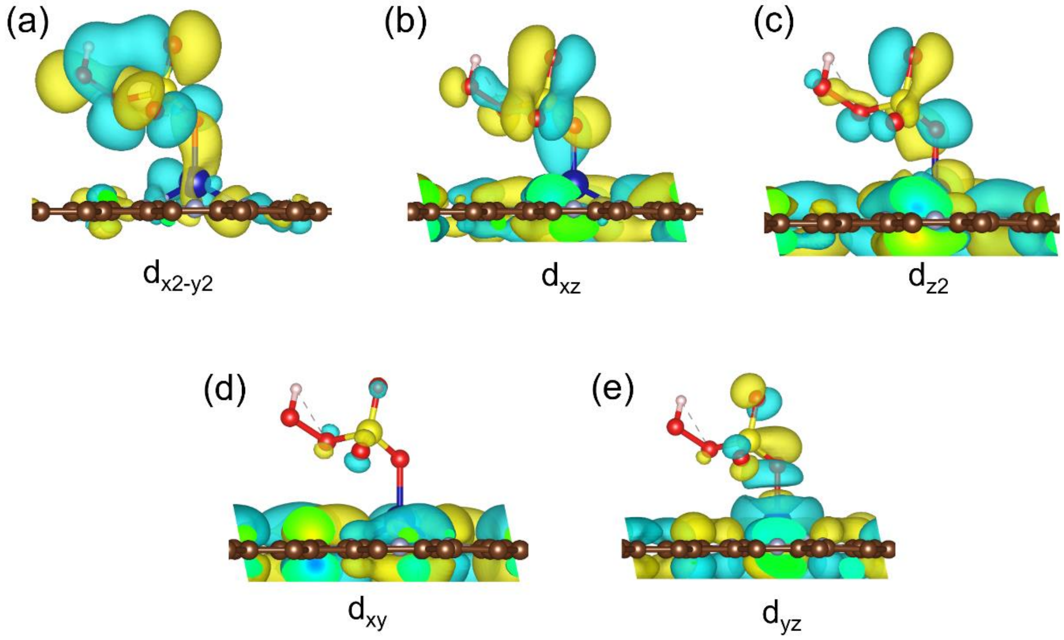

Figrue R21. The d electron cloud distribution of CoN_3C-PMS. The wave function of (a) d_{x2-y2}, (b) d_{xz}, (c) d_{z2}, (d) d_{xy}, (e) d_{yz} in CoN_3C-PMS.

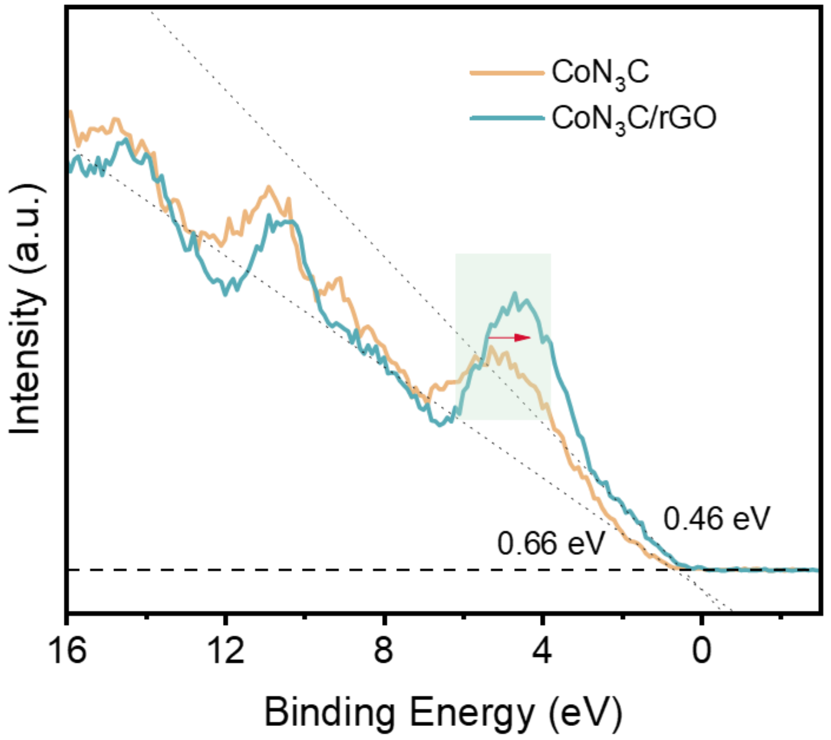

Figure R22. The valence band and d electron occupancy state information of catalysts. The UPS spectra of CoN_3C and CoN_3C/rGO.

Reviewer #3:

The work by Y. Chen and co-workers (NCOMMS-25-87289) tries to explain how to overcome the persistent trade-off between activity and selectivity in Fenton-type catalysis, particularly in peroxymonosulfate (PMS) activation. The authors designed a single-atom cobalt catalyst with a "nano-island" structure (called CoN_3C/rGO) to direct PMS activation toward the desired direct Electron Transfer Pathway (ETP) process, a non-radical mechanism known for its high selectivity and resistance to matrix interference. The authors hypothesize that the catalyst consists of single cobalt atoms anchored on islands of carbon nitride (CoN_3C) supported by a layer of reduced graphene oxide (rGO), which acts as a "sea". The "island-sea" architecture exploits the synergistic effect to precisely modulate the electrons in the cobalt d-orbital. Theoretical analyses show that rGO elevates the center of the Co d-band and makes the electronic state of the d-orbital more concentrated and intense near the Fermi level. However, a clear experimental characterization, to support the predictions made by the DFT simulation, is lacking. The XPS technique, for example, partially used in the study, could have provided several useful insights if the Co2p and valence band regions had also been analyzed. The latter, although we all know that UPS is the best technique for valence band analysis, could have as well provided information on the Co3d, O2p, and N2p orbitals (the latter would have provided interesting information also on the hybridization of nitrogen, sigma and π bonds, etc.) and on the position of the valence band maximum with respect to the Fermi level, to directly verify the presence or absence of "occupied" states in its vicinity.

What emerges from this work is undoubtedly a huge amount of data acquired using various techniques, some complementary, which, however, have not been adequately commented on or analyzed. Furthermore, the reader struggles to follow the work's logical thread, as the different sections appear disjointed. Fewer analyses could have been performed, but more time and precision could have been devoted to presenting the results. At the same time, the manuscript contains numerous errors and omissions, making the writing and subsequent editing rather careless. This is unfortunate, given

the amount of work done. To give a few more explicit examples:

Response: Thank you for taking the time to review our manuscript and providing us with your insightful comments. We greatly appreciate your efforts in helping us enhance the quality of our work. We agree with your constructive criticisms regarding the disjointed logical flow, the lack of experimental verification for electronic states, and the careless errors in the original submission. In the revised manuscript and revised Supplementary information, we did our best to deeply analyze the data, and reorganized the relationship between the data and the results. For example, we specifically strengthened the discussion to correlate the "island-sea" structural features directly with the observed catalytic selectivity and activity. To experimentally support the DFT predictions regarding d-orbital modulation, we have performed Ultraviolet Photoelectron Spectroscopy (UPS) and XPS analyses as suggested. We have expanded the discussion on existing characteristic and DFT data to interpret the physical significance of the “island-sea” interaction, rather than merely describing the phenomena. Meanwhile, we have carefully corrected the errors and omissions pointed out by the reviewer and polished the language to ensure the manuscript is accurate and readable. We believe these substantial revisions have addressed the core concerns and significantly elevated the quality of this work.

Comment 1. If you use ETP acronym, then you should write instead "direct electron transfer pathway (ETP)", otherwise you should use the acronym DET (direct electron transfer).

Response: Thank you for your valuable suggestions. We apologize for our neglect in this matter. We have corrected the “direct electron transfer (ETP)” to “direct electron transfer pathway (ETP)” in the manuscript.

Comment 2. When authors stated “Here, we utilize a cobalt single atom catalyst anchored on carbon nitride supported by reduced graphene oxide (CoN_3C/rGO) to activate PMS”, then experimental evidence of this connection should be added. XAS measurements have not demonstrated it precisely and unequivocally, for instance. “In

the X-ray absorption near-edge structure (XANES) spectroscopy, the adsorption of CoN_3C/rGO and CoN_3C is very near to Co_3O_4, indicating the valence of Co in two catalysts is between +2 and +3, while rGO further decreases the electron density of Co site (Fig. 1g). The extended X-ray absorption fine structure (EXAFS) spectra reveals that no Co-Co interaction exists in CoN_3/rGO and CoN_3C, while obvious Co-N can be observed at 1.4-1.6 Å, further demonstrating the single atom form of Co". If the authors hypothesized a direct Co-N bond, why didn't they use XPS to confirm this hypothesis? The literature contains several articles that refer to this type of bond and the associated chemical shifts, which are distinguishable ("Atomically dispersed cobalt on carbon nitride for peroxymonosulfate activation: Switchable catalysis enabled by light irradiation" https://doi.org/10.1016/j.cej.2022.137277; "Regulating Second-Shell Coordination in Cobalt Single-Atom Catalysts toward Highly Selective Hydrogenation" https://doi.org/10.1021/acsnano.4c05637; "Cobalt/nitrogen doped porous carbon as catalysts for efficient oxygen reduction reaction: Towards hybrid enzymatic biofuel cells" https://doi.org/10.1016/j.electacta.2021.138791). What the authors demonstrated with XAS measurements is that the average oxidation state of Co was between +2 and +3, but this does not allow us to distinguish between a "doping" inclusion or a simple functionalization, with Co bound to existing functionalities on both rGO and/or $-N_3C$, for example;

Response: Thank you for your professional comments. In our original manuscript, the peak at 399.5 eV was assigned solely to pyrrolic N. We agree with your advice that, considering the coordination chemistry of cobalt single atoms, this specific binding energy region should also reflect the contribution of Co-N coordination bonds. As pointed out by the reviewer and the recommended literature (*Electrochim. Acta, 389, 138791 (2021)*), the Co-N typically exhibits a binding energy around 399.1 eV, which is closely adjacent to the standard position of pyrrolic N. Due to the relatively low cobalt loading in CoN_3C/rGO catalyst, the signal for Co-N coordination is partially superimposed under the pyrrolic N peak envelope. And this phenomenon has been reported (please see: *Small 17, 2104939 (2021); J. Am. Chem. Soc. 145, 8052-8063*

(2023)). This XPS assignment is fully consistent with our EXAFS analysis, where a prominent scattering path at 1.4-1.6 Å (characteristic of the Co-N first shell) was observed. The absence of Co-Co interactions further supports that Co is chemically coordinated as single atoms rather than physically adsorbed clusters. More importantly, wavelet transform (WT) EXAFS shows a peak at about 5 Å in k space, which is also ascribed to Co-N, which is also exhibited in WT-EXAFS results of CoPc (A chemical substance has structure that Co center coordinated with N). The re-fitting N1s spectra, EXAFS and WT-EXAFS results are shown in **Figure R23**.

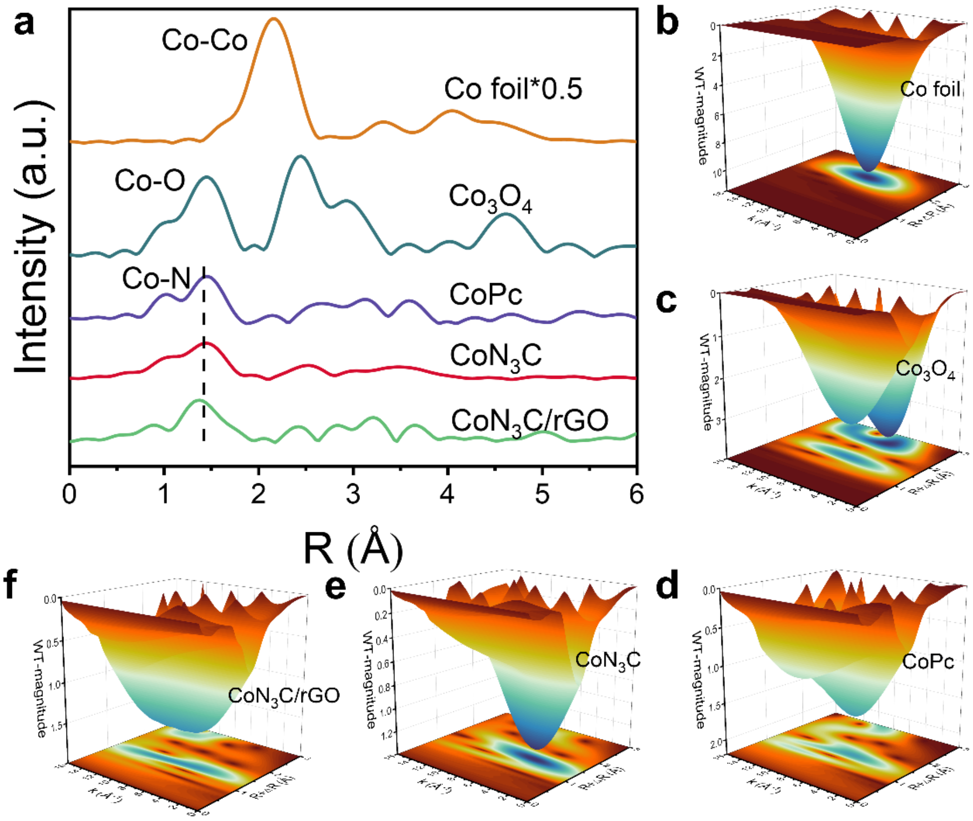

Figure R23. The coordination environment of catalysts. a Fourier transform EXAFS of CoN_3C/rGO, CoN_3C and reference samples. The Wavelet transforms of **b** Co foil, **c** Co_3O_4, **d** CoPc, **e** CoN_3C and **f** CoN_3C/rGO.

Comment 3. if authors state that there are species related to N-oxidized in the N1s region, which is the counterpart in O1s region? Where is the chemical shift related to this N-O bond?

Response: Thank you for your valuable question. This comment prompted us to rigorously re-examine the N 1s peak assignments (Details can be seen in the next response and **Figures R25-R26**). Upon re-fitting the N 1s spectra with strict constraints, we found that the previous assignment of oxidized N species was unreasonable. The revised fitting results show that the N species in the CoN_3C/rGO composite are composed of pyridinic N (398.7 eV), Co-N/pyrrolic N (399.5 eV), and graphitic N (401.0 eV). No peak related to N-oxides (>402 eV) was detected. Consequently, the absence of oxidized N species in both N 1s and O 1s spectra confirms the chemical consistency of new results.

Comment 4. peak assignments in the C1s and N1s regions are inconclusive and in some cases incorrect. For example, for the N-C=N bond, it is initially positioned at around 286 eV for the CoN_3C/rGO sample, then shifts, for no apparent reason, to around 288.5 eV for the CoN_3C sample. In some cases, the FWHM of some components is on the order of around 1.5-2 eV, while in others it reaches almost 4 eV, for no apparent reason, as these peaks are always related to real bonds and not satellites, loss or shake-up features. The N1s region also presents several inconsistencies: N-pyridine is initially positioned at around 398.5 eV (CoN_3C/rGO), then shifts to around 397.8 eV (CoN_3C), with no explanation for the shift. And what about the $\pi-\pi^*$ peaks, both in C1s and N1s regions, which have been suggested by other techniques results?

Response: Thank you for your valuable suggestions. We fully accept that the original peak assignments and fitting parameters were inaccurate and inconsistent. To address these valid concerns, we have completely re-processed and re-fitted the high-resolution C 1s, N 1s, O 1s and Co 2p spectra for all samples (CoN_3C/rGO, CoN_3C, rGO), strictly adhering to standard binding energy references and physical constraints. The new fitting results are shown in **Figures R24-27**, which would been added to the revised **Supplementary information** as **Supplementary Figs. 3-6**. The corrections mainly include: 1) In the original manuscript, the peaks at around 286 eV were wrongly assigned to N-C=N, while these peaks are now correctly assigned to C-N or C-O species. Meanwhile, the N-C=N was represented by the peak at 287.4eV in CoN_3C/rGO and

288.3 eV in CoN_3C. 2) The FWHM for all primary chemical states in C 1s, N 1s, O 1s spectra was constrained to a reasonable range of 1.2 – 1.8 eV. 3) We have added the π–π* shake-up satellite peaks in the revised fitting results. For CoN_3C/rGO, the satellite peak is fitted at 290.1 eV. For rGO, the satellite peak is fitted at 290.5 eV. 4) We carefully analyzed the shifts between CoN_3C and CoN_3C/rGO to provide a reasonable explanation. The negative shift of N-C=N in C 1s (288.3 eV for CoN_3C vs. 287.4 eV for CoN_3C/rGO) and positive shifts of pyridinic N in N 1s (398 eV for CoN_3C vs. 398.7 eV for CoN_3C/rGO) and Co 2p provide robust evidence for the electron transfer from the rGO substrate to the CoN_3C islands. This confirms the strong electronic modulation effect of the "island-sea" architecture, consistent with our DFT results. Detailed discussion in the revised **manuscript** can be found below (**Line 112-119, Page 3**):

The electronic states of the prepared catalysts were analyzed by X-ray photoelectron spectra (XPS) (Supplementary Figs. 3-6 and Supplementary Table 3). More pronounced N-C=N peak appears at CoN_3C/rGO than CoN_3C, indicating the existence of rGO anchors and disperses the CoN_3C "islands", which maximizes the exposure of the N-C=N coordination network to the surface. Compared with CoN_3C, the negative shift in the N-C=N skeleton and positive shifts in the Co 2p and pyridinic N of CoN_3C/rGO suggests an electron injection from the conductive rGO "sea" to the electron-deficient carbon nitride "islands", confirming a significant interfacial electronic coupling.

Other detailed discussion in the revised **Supplementary information** can be found below (**Pages 7-8**):

The high-resolution C 1s spectra provide detailed insights into the structural evolution of the carbon matrix. For the CoN_3C, two peaks at 283.8 eV and 284.8 eV are assigned to the graphitized sp^2 C=C framework and sp^3 C-C species, respectively[11]. The N-C=N peak, representing the triazine/heptazine heterocyclic skeleton, is identified at 288.3 eV[12]. In contrast, the N-C=N peak in CoN_3C/rGO appears at 287.4 eV[13]. This significant negative shift indicates an increased electron density on the carbon atoms in the vicinity of the Co-N_3 centers.

The N 1s spectra further corroborate the specific coordination environment. The spectrum of CoN3C/rGO can be deconvoluted into three distinct peaks at 398.7 eV, 399.5 eV, and 401.0 eV, corresponding to pyridinic N, Co-N/pyrrolic N, and Graphitic N, respectively[14-17]. More importantly, higher ratio of pyrrolic N than pyridinic N exists in CoN3C/rGO, while the opposite data is exhibited for CoN3C. This suggests that the strong interfacial interaction with rGO induces the transformation of some rigid six-membered pyridinic rings into more flexible, electron-rich five-membered pyrrolic defects.

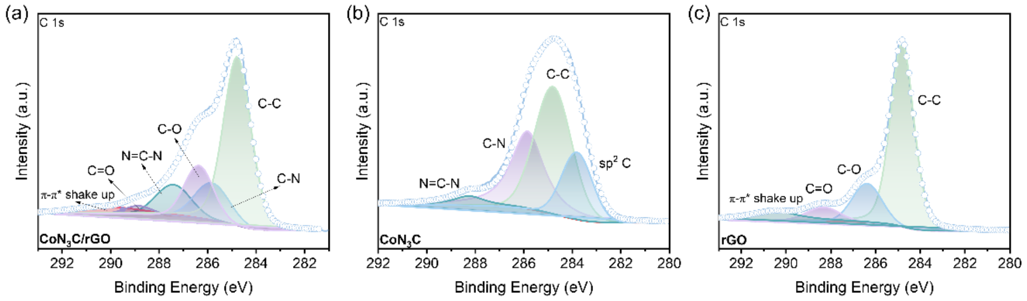

Figure R24. XPS characterization of catalysts. High-resolution XPS spectra of C 1s for (a) CoN3C/rGO, (b) CoN3C, (c) rGO.

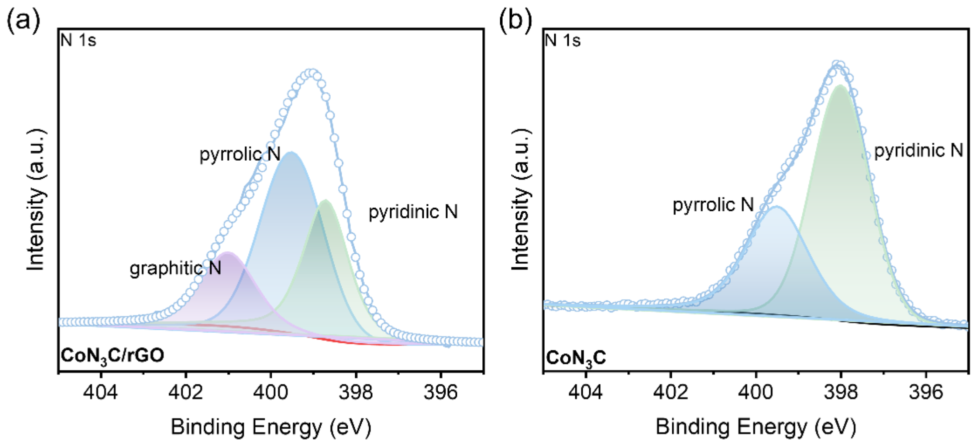

Figure R25. XPS characterization of catalysts. High-resolution XPS spectra of N 1s for (a) CoN3C/rGO, (b) CoN3C.

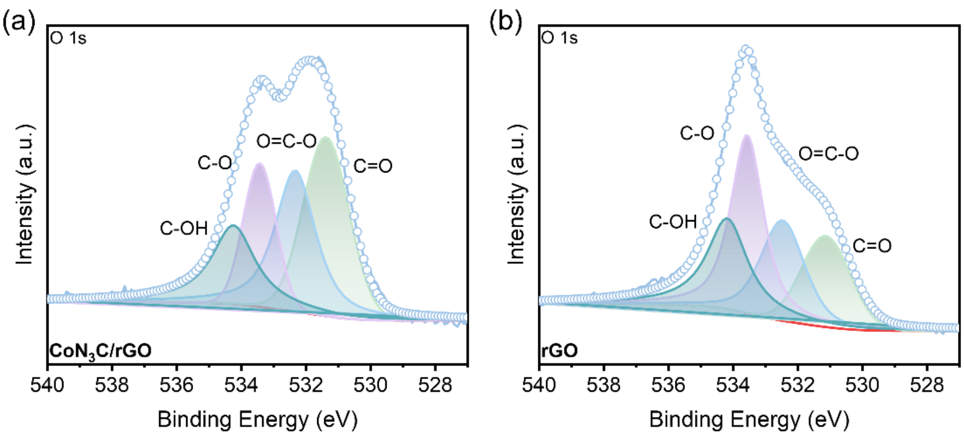

Figure R26. XPS characterization of catalysts. High-resolution XPS spectra of O 1s for (a) CoN_3C/rGO, (b) rGO.

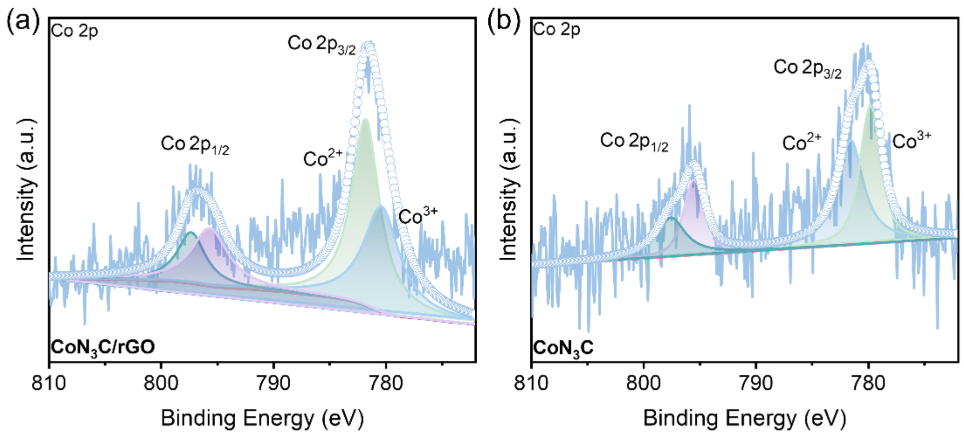

Figure R27. XPS characterization of catalysts. High-resolution XPS spectra of Co 2p for (a) CoN_3C/rGO, (b) CoN_3C.

Comment 5. "Density function theory (DFT)", should be "functional"

Response: Thank you for your valuable suggestions. I apologize for our neglect in this matter. We have corrected the "Density function theory (DFT)" to "Density functional theory (DFT)" in the revised **manuscript**.

Comment 6. Which is the precursor used for the Co: $Co(NO_3)_2 \cdot 6H_2O$ or $CoCl_2$? The first has been mentioned in the sample preparation procedure, while the second is reported in fig. 1-a;

Response: Thank you for your valuable suggestions. We apologize for this mistake. The correct precursor is $Co(NO_3)_2{\bullet}6H_2O$. We have corrected it in the revised **manuscript Fig. 1a**. The corresponding figure is shown below (**Figure R28**):

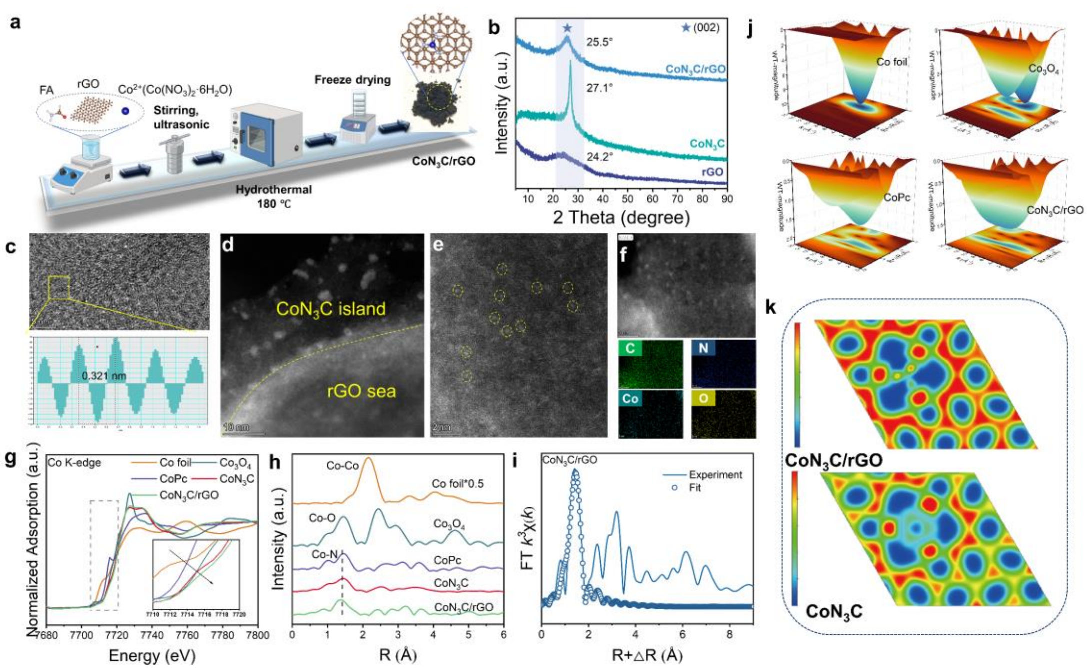

Figure R28. Synthesis and microscopic structures of catalysts. a Synthesis procedure of CoN_3C/rGO. **b** XRD pattern of different catalysts. **c** Interlayer spacing of CoN_3C island. **d, e** AC-HAADF-STEM images of CoN_3C/rGO. **f** EDS-mapping of CoN_3C/rGO. **g** Normalized Co k-edge XANES of CoN_3C/rGO, CoN_3C and reference samples. **h** Fourier transform EXAFS of CoN_3C/rGO, CoN_3C and reference samples. **i** Co k-edge EXAFS fitting analysis of CoN_3C/rGO in R space. **j** Wavelet transforms of Co foil, Co_3O_4, CoPc, and CoN_3C/rGO. **k** The two-dimensional electron localization functions of CoN_3C/rGO and CoN_3C.

Comment 7. Co signal is not clearly detectable in all the EDS maps presented in the manuscript or SI: which is the amount of Co in these samples. What about survey spectra from XPS analysis or EDS?

Response: Thank you for your professional questions. Actually, we have detected the amount of Co in CoN_3C/rGO and CoN_3C according to the atomic adsorption spectroscopy (AAS, GGX-920, Haiguang), and shown related data (1.25% for

CoN_3C/rGO; 1.97% for CoN_3C) in the **Supplementary Table 7**. Here, we once again present this table to you (**Table R3**). Additionally, we apologize for ignoring the data detected data from EDS mapping and XPS, which are shown here as the **Figures R29-R31 and Tables R4-R7.** We have added them to the revised **Supplementary information** as the **Supplementary Figs. 12-14** and **Supplementary Tables 3-6**. Due to the differences in the area and depth of the detection, the element contents measured by these two techniques are different. Detailed discussion in the revised **manuscript** can be found below (**Line 112-113, Page 3**; **Line 128-130, Page 3**):

More chemical species and surface elements content of the prepared catalysts were analyzed by XPS (Supplementary Figs. 3-6 and Supplementary Table 3).

SEM- energy dispersive spectroscopy (EDS) mapping indicates the homogeneous distribution of C, N, O, Co and low content of Co in catalysts (Supplementary Figs. 9-14 and Supplementary Tables 4-6).

Table R3. The exact Co-loading content of catalysts.

Catalysts	CoN_3C/rGO	CoN_3C	Used CoN_3C/rGO
Co content %	1.25	1.97	0.80

Table R4. The elements content of catalysts from XPS.

Elements / Catalysts	Co	N	C	O
CoN_3C/rGO	0.44	19.09	71.08	9.4
CoN_3C	0.41	30.96	61.34	7.3
rGO	/	/	88.01	12
Used CoN_3C/rGO	0.26	14.06	79.37	6.32

Table R5. The elements content of CoN_3C/rGO from SEM-EDS mapping.

Elements	wt %
Co	0.30
N	14.96
C	75.96
O	8.79

Table R6. The elements content of CoN_3C from SEM-EDS mapping.

Elements	wt %
Co	1.16
N	37.30
C	61.55

Table R7. The elements content of rGO from SEM-EDS mapping.

Elements	wt %
N	0.00
C	86.96
O	13.04

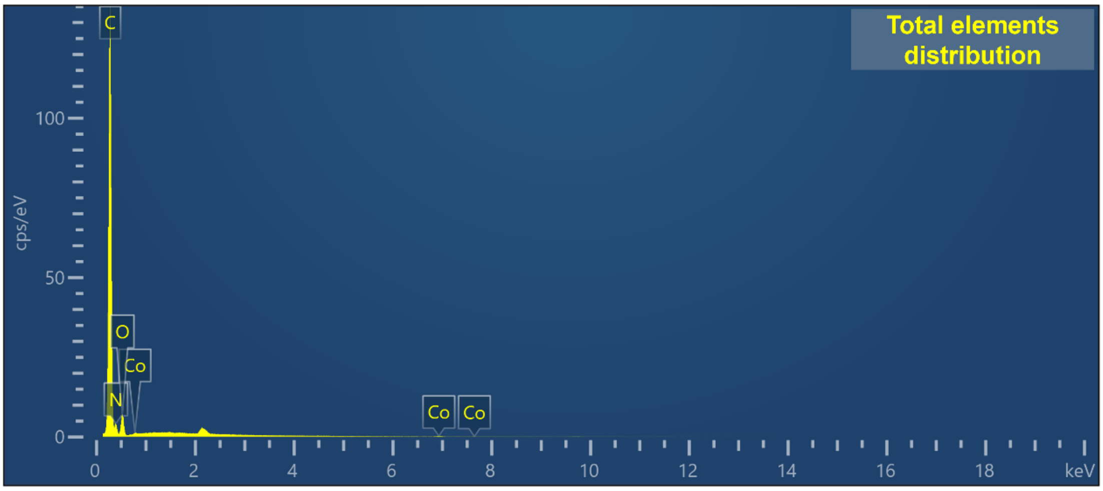

Figure R29. Elements contents in CoN_3C/rGO from EDS mapping. The signals of total elements distribution results about CoN_3C/rGO.

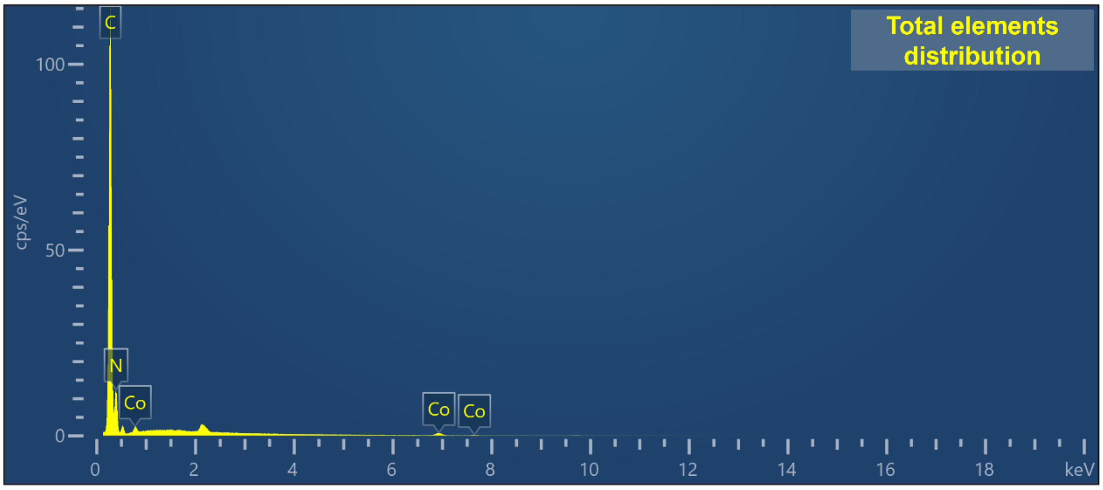

Figure R30. Elements contents in CoN_3C from EDS mapping. The signals of total elements distribution results about CoN_3C.

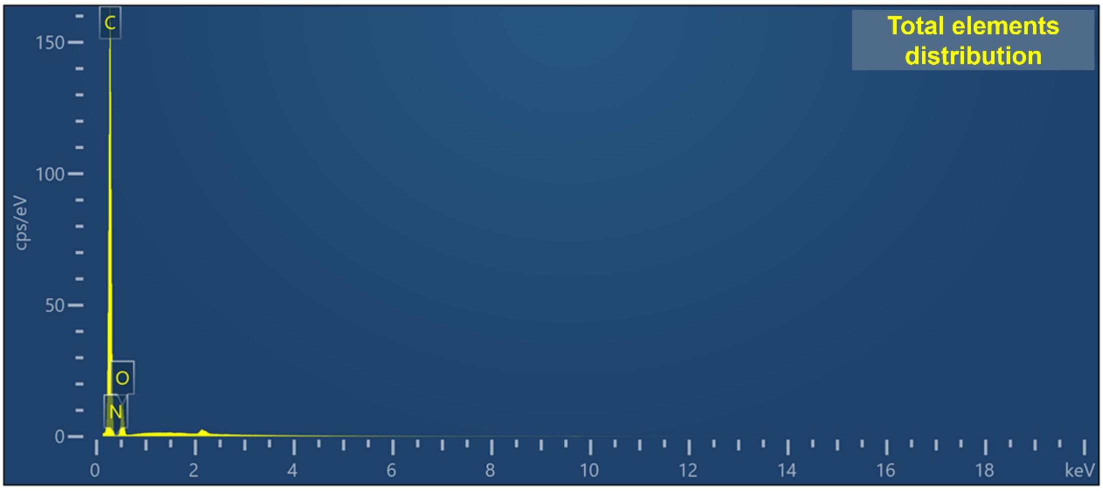

Figure R31. Elements contents in rGO from EDS mapping. The signals of total elements distribution results about rGO.

Comment 8. Please do not use terms like "exceptional" ("Exceptional resistance to matrix interference…") or "obvious" ("According to Raman spectra, the obvious D band…"), they are a bit out of place in a scientific text.

Response: Thank you for your valuable and professional suggestions. We carefully revised the related terms in the revised **manuscript** and **revised Supplementary information**. The corresponding revision can be found below:

Line 42-43, Page 1 in the revised **manuscript**, we corrected "Exceptional resistance to matrix interference (e.g., anions, humic acid matter), higher oxidant utilization efficiency, and low damage to catalyst." to "Superior resistance to matrix interference (e.g., anions, humic acid matter), higher oxidant utilization efficiency, and low damage to catalyst."

Line 92-94, Page 2 in the revised **manuscript**, we corrected "The synthesized CoN_3C/rGO can achieve significantly superior PMS activation performance compared to CoN_3C and most of the previously reported catalysts." to "The synthesized CoN_3C/rGO can achieve robust PMS activation performance compared to CoN_3C and most of the previously reported catalysts.".

Line 97-98, Page 2 in the revised **manuscript**, we corrected "These advantages provide an excellent degradation efficiency for electron-donating contaminants in

CoN_3C/rGO/PMS system." to "These advantages provide a remarkable degradation efficiency for electron-donating contaminants in CoN_3C/rGO/PMS system.".

Page 5 in the revised **Supplementary information**, we corrected "According to Raman spectra, the obvious D band (disorder induced phonon mode) and G band (graphite band) of CoN_3C/rGO, CoN_3C, and rGO are obtained at 1352 cm^{-1} and 1580 cm^{-1}." to "According to Raman spectra, the characteristic D band (disorder induced phonon mode) and G band (graphite band) of CoN_3C/rGO, CoN_3C, and rGO are obtained at 1352 cm^{-1} and 1580 cm^{-1}.".

Line 185, Page 4 in the revised **manuscript**, we corrected "Hence, sole CoN_3C or rGO would cause more oxidant waste." to "Hence, sole CoN_3C or rGO would cause higher oxidant waste.".

Line 191-193, Page 4 in the revised **manuscript**, we corrected "In the range of 3.0-11.0, almost 100% removal efficiency could be achieved within 10 min through CoN_3C/rGO activating PMS, even though the k_{obs} decreased in the strong base condition, exhibiting a wide pH adaptability (Fig. 2d and Supplementary Fig. 19)" to "In the range of 3.0-11.0, almost 100% removal efficiency could be achieved within 10 min through CoN_3C/rGO activating PMS, even though the k_{obs} decreased in the strong base condition, exhibiting a broad pH adaptability (Fig. 2d and Supplementary Fig. 31)".

Line 253-255, Page 6 in the revised **manuscript**, we corrected "Moreover, the potential and current variations in the CoN_3C/rGO/PMS system were larger than those observed in CoN_3C/PMS, and negligible changes were detected in the rGO/PMS system, indicating stronger electron transfer interaction existed due to the "nano-island-like" structure." to "Moreover, the potential and current variations in the CoN_3C/rGO/PMS system were larger than those observed in CoN_3C/PMS, and negligible changes were detected in the rGO/PMS system, indicating enhanced electron transfer interaction existed due to the "nano-island-like" structure.".

Line 290-291, Page 6 in the revised **manuscript**, we corrected "While for CoN_3C, electrons form highly localized regions, which lead to stronger electron mobility for CoN_3C/rGO, indicating the rGO sea not only supply electron to CoN_3C island, but also

enhances the mobility of electrons according to its π delocalization network.” to “While for CoN_3C, electrons form highly localized regions, which lead to enhanced electron mobility for CoN_3C/rGO.”.

Line 336-337, Page 7 in the revised **manuscript**, we corrected “The nonradical dominant pathway of ETP in CoN_3C/rGO/PMS system shows outstanding application potential.” to “The nonradical dominant pathway of ETP in CoN_3C/rGO/PMS system shows highly promising application potential.”.

Line 342-343, Page 7 in the revised **manuscript**, we corrected “Therefore, CoN_3C/rGO has outstanding resistance against common aqueous interfering substances.” to “Therefore, CoN_3C/rGO maintains high resistance against common aqueous interfering substances.”.

Line 387-388, Page 8 in the revised **manuscript**, we corrected “Additionally, this catalyst exhibits 94% selectivity to generate the ETP path, which can obtain excellent degradation efficiency for various electron-rich contaminants.” to “Additionally, this catalyst exhibits 94% selectivity to generate the ETP path, which can obtain robust degradation efficiency for various electron-rich contaminants.”.

Comment 9. I suggest the authors to read the following articles, to understand how to display data and to correlate results: "A review of the innovations in metal- and carbon-based catalysts explored for heterogeneous peroxymonosulfate (PMS) activation, with focus on radical vs. non-radical degradation pathways of organic contaminants" (https://doi.org/10.1016/j.cej.2020.127957), “Advancements on Single-Atom Catalysts-Mediated Persulfate Activation: Generating Reactive Species for Contaminants Elimination in Water” (https://doi.org/10.3390/molecules29235696), or “Transition Metal-Nitrogen-Carbon Single-Atom Catalysts Enhanced CO2 Electroreduction Reaction: A Review.” (https://doi.org/10.1002/cssc.202401557).

Response: Thank you for your valuable suggestions and references. We have carefully read all references you provided. Most data and correlate results were reanalyzed and rewritten in the revised manuscript and revised Supplementary information. For example, some revisions are listed below:

Line 105-109, Page 3 in the revised **manuscript**, we corrected "The XRD pattern of CoN_3C shows a sharp peak at 2θ= 27.1°, corresponding to carbon nitride. And a wide peak at 2θ= 24.2° appears at rGO (Fig. 1b). In contrast, a peak at 2θ= 25.5° was observed in CoN_3C/rGO, indicating the new interface was formed between the CoN_3C island and rGO sea, and the interlayer spacing of rGO decreased after composite. Meanwhile, the original highly crystalline structure of CoN_3C was disrupted and interfaced with rGO in a small-sized and low-stacked manner." to " The X-ray diffraction (XRD) patterns reveal a critical structural evolution upon compositing (Fig. 1b). Unlike the physical mixture, the CoN_3C/rGO composite exhibits a distinct diffraction peak at 2θ= 25.5°, which sits between the characteristic peaks of rGO (24.2°) and CoN_3C (27.1°), suggesting the original highly crystalline structure of CoN_3C was disrupted and interfaced with rGO in a small-sized and low-stacked manner.".

Line 121-128, Page 3 in the revised **manuscript**, we corrected "In the SEM and TEM images, clear graphene structures are illustrated in rGO and CoN_3C/rGO samples, while CoN_3C shows an irregular shape Furthermore, SEM reveals that CoN_3C/rGO exhibits more obvious wrinkling and curling. This might be due to the composite destruction of the originally continuous and extended sp^2 carbon network of rGO by the CoN_3C islands on the rGO surface, resulting in local stress and structural defects." to "In the scanning electron microscopic (SEM) and transmission electron microscopy (TEM) images, the pristine rGO presents a smooth and sheet-like structure, limiting mass transfer (Supplementary Figs. 7-8). Conversely, the pure CoN_3C exhibits an irregular, and aggregated morphology, typical of bulk carbon nitride, which severely buries the active Co sites. Upon hydrothermal process, the bulky CoN_3C aggregates completely disappear, replaced by ultra-thin "nano-island" uniformly dispersed on the rGO surface. This indicates that the rGO substrate effectively exfoliates and anchors the CoN_3C precursors, preventing their self-aggregation. And these rigid islands induce significant wrinkling of the rGO sheets.".

Line 109-112, Page 3 in the revised **manuscript**, **and Pages 5-6** in the revised **Supplementary information**, we corrected "According to Raman spectra, the obvious

D band (disorder induced phonon mode) and G band (graphite band) of CoN_3C/rGO, CoN_3C, and rGO are obtained at 1352 cm^{-1} and 1580 cm^{-1} (Supplementary Fig. 6). The peak intensity ratio (I_D/I_G) is used to represent the defect level of materials. The results indicate that more defects were generated in CoN_3C/rGO (0.98 vs 0.89 and 0.92). For the FTIR spectra, the peak of C-OH stretching vibrations (1220 cm^{-1}) is present in three materials (Supplementary Fig. 7). The characteristics of C-N (1370-1420 cm^{-1}) and C=N (1610-1680cm^{-1}) are observed in CoN_3C/rGO and CoN_3. While a peak at 1750 cm^{-1} corresponding to C=O of rGO disappeared after compositing with CoN_3C to form CoN_3C/rGO, indicating that rGO was further reduced under hydrothermal conditions, which possibly led to the reduction of layer spacing" to "The results of Raman spectra and Fourier-transform infrared (FTIR) further demonstrate the formation of "nano-island" interface and the resulting local structural changes from the perspectives of chemical bond and defect level (Supplementary Figs. 1-2).", "According to Raman spectra, the charateristic D band (disorder induced phonon mode) and G band (graphite band) of CoN_3C/rGO, CoN_3C, and rGO are obtained at 1352 cm^{-1} and 1580 cm^{-1}. The defect level of materials represented by the peak intensity ratio (I_D/I_G) indicate that more defects were generated in CoN_3C/rGO (0.98 vs 0.89 and 0.92), which can support more anchoring sites, and regulate the electronic structure of active site, enhancing the catalytic activity", and "For the FTIR spectra, the peak of C-OH stretching vibrations (1220 cm^{-1}) is present in three materials. The characteristics of C-N (1370-1420 cm^{-1}) and C=N (1610-1680cm^{-1}) are observed in CoN_3C/rGO and CoN_3, confirming the CoN_3C island maintain their frameworks. While a peak at 1750 cm^{-1} corresponding to C=O of rGO disappeared after compositing with CoN_3C to form CoN_3C/rGO, indicating that rGO was further reduced under hydrothermal conditions, which restore its graphitic conjugated π-electron network".

Line 119-119, Page 3 in the revised **manuscript**, **and Pages 7-8** in the revised **Supplementary information**, we corrected "More chemical species information was analyzed by XPS. The C 1s of CoN_3C/rGO can be fitted to five peaks, ascribing to C-C/C=C (284.8 eV), C-N (285.4eV), N-C=N (287.9 eV), C=O (286.9 eV), and C-O

(286.0 eV), respectively (Supplementary Fig. 8). Four species, including pyridinic N (398.7 eV), pyrrolic N (399.5 eV), graphitic N (400.6 eV), and oxidized N (401.2 eV) existed in the N 1s spectra of CoN_3C/rGO, more than only pyridinic N and pyrrolic N in CoN_3C (Supplementary Fig. 9). The introduction of rGO sea provides more carbon substrate, allowing N to be doped into the lattice of rGO, further enhancing the electron transfer ability of support. For O 1s, rGO and CoN_3C/rGO show the same O species, including C=O (531.5 eV), C-O (533.0 eV), and C-OH (534.0 eV) (Supplementary Fig. 10). While the composite process decreased the O content, which also indicates that the hydrothermal method further reduced the rGO." to "The electronic states and surface elements content of the prepared catalysts were analyzed by X-ray photoelectron spectra (XPS) (Supplementary Figs. 3-6 and Supplementary Table 3). More pronounced N-C=N peak appears at CoN_3C/rGO than CoN_3C, indicating the existence of rGO anchors and disperses the CoN_3C "islands", which maximizes the exposure of the N-C=N coordination network to the surface. Compared with CoN_3C, the negative shift in the N-C=N skeleton and positive shifts in the Co 2p and pyridinic N of CoN_3C/rGO suggests an electron injection from the conductive rGO "sea" to the electron-deficient carbon nitride "islands", confirming a significant interfacial electronic coupling.", "The high resolution C 1s spectra provide detailed insights into the structural evolution of the carbon matrix. For the CoN_3C, two peaks at 283.8 eV and 284.8 eV are assigned to the graphitized sp^2 C=C framework and sp^3 C-C species, respectively. The N-C=N peak, representing the triazine/heptazine heterocyclic skeleton, is identified at 288.3 eV. In contrast, the N-C=N peak in CoN_3C/rGO appears at 287.4 eV. This significant negative shift indicates an increased electron density on the carbon atoms in the vicinity of the Co-N_3 centers.", "The N 1s spectra further corroborate the specific coordination environment. The spectrum of CoN_3C/rGO can be deconvoluted into three distinct peaks at 398.7 eV, 399.5 eV, and 401.0 eV, corresponding to pyridinic N, Co-N/pyrrolic N, and Graphitic N, respectively. More importantly, higher ratio of pyrrolic N than pyridinic N exists in CoN_3C/rGO, while the opposite data is exhibited for CoN_3C. This suggests that the strong interfacial interaction with rGO induces the transformation of

some rigid six-membered pyridinic rings into more flexible, electron-rich five-membered pyrrolic defects."

Line 289-294, Page 6 in the revised **manuscript**, we corrected "After forming the complex catalyst-PMS*, the degree of electron delocalization around Co atom in CoN_3C/rGO increases. While for CoN_3C, electrons form highly localized regions, which lead to stronger electron mobility for CoN_3C/rGO, indicating the rGO sea not only supply electron to CoN_3C island, but also enhances the mobility of electrons according to its π delocalization network." to "Upon the formation of catalyst-PMS* complex, the Co center in CoN_3C/rGO exhibits a significantly increased degree of electron delocalization. While for CoN_3C, electrons form highly localized regions, which lead to enhanced electron mobility for CoN_3C/rGO. This transition from localization to delocalization suggests that the rGO "sea" does not merely act as a passive support, its extended π-conjugated network actively participates in charge redistribution, thereby lowering the energy barrier for electron transport from the Co "island" to the adsorbed PMS.".

And there are also some other detailed revisions can be seen in the revised **manuscript** and revised **Supplementary information**.

More importantly, we conducted UPS to obtain valence band and d electron occupancy state information, further confirming our DFT results. As shown in **Figure R32** (as the **Fig. 4h** in the revised **manuscript**), a narrower peak with higher intensity near the Fermi level appears at CoN_3C/rGO sample compared with CoN_3C, explaining the DOS results. Additionally, the valence band maximum (VBM) position of CoN_3C/rGO (0.46 eV) is closer to Fermi level than CoN_3C (0.66 eV), correlated with higher d band center of CoN_3C/rGO. This electronic reconfiguration enhances the orbital hybridization between the Co active sites and PMS molecules, thereby kinetically accelerating the ETP. Related description has been added to manuscript as below (**Line 321-324, Page 7**):

The above results were also confirmed by Ultraviolet Photoelectron Spectroscopy (UPS) (Fig. 4h). Compared with CoN_3C, the occupied states near the E_F become narrower

and more significant for CoN_3C/rGO. And the upshift of the valence band maximum (VBM) towards the E_F suggests an elevation of the d-band center (0.46 eV for CoN_3C/rGO vs. 0.66 eV for CoN_3C).

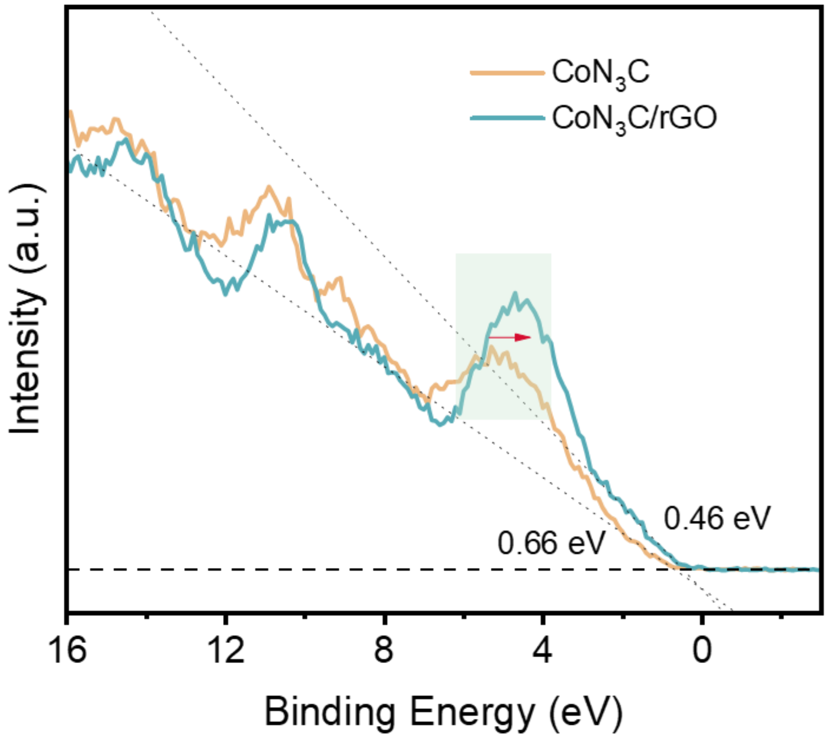

Figure R32. The valence band and d electron occupancy state information of catalysts. The UPS spetra of CoN_3C and CoN_3C/rGO.

Comment 10. Although the ETP demonstrated a contribution of approximately 94% and was verified by a two-cell electron transfer experiment, the suggestion in the introduction that the ETP avoids directly introducing PMS into the wastewater (e.g., using a salt bridge device) to circumvent the environmental concern arising from SO_4^{2-}, has not been fully implemented in the final continuous application (which still involves the addition of PMS for treatment). Furthermore, the study demonstrated that the system has variable selectivity for different contaminants (e.g., BPA, ofloxacin, phenol, etc.), and established a structure-activity relationship (QSAR) indicating that electron-donating organic contaminants are more readily degraded. However, detailed mechanistic analysis (such as intermediate degradation pathways) was provided only

for bisphenol A (BPA), leaving specific orbital/electronic interactions with the other tested contaminants less explored.

Response: Thank you for your professional and valuable suggestions. For suggest that "the suggestion in the introduction that the ETP avoids directly introducing PMS into the wastewater" mentioned in the Introduction. We conducted the degradation experiment in a dual-chamber separated device (**Figure R33**, as the **Supplementary Fig. 77** in the revised **Supplementary information**). In this setup, BPA and PMS were physically separated by a proton exchange membrane (PEM), and the optimal CoN_3C/rGO catalyst was loaded onto the electrodes connected by an external circuit. We acknowledge that the reaction kinetics in this macroscopically separated device are significantly slower than in the direct-contact batch system. This kinetic drop is scientifically highly reasonable and is governed by macroscopic engineering limitations rather than the intrinsic catalyst activity. The catalytic process shifted from a 3D suspension (homogeneous powder dispersion) to a 2D planar electrode configuration, drastically reducing the active solid-liquid interface area and making macroscopic diffusion the rate-determining step. The internal resistance of the PEM and the external circuit inevitably dissipate the thermodynamic driving force for the long-distance electron transfer.

Additionally, to provide specific orbital/electronic interactions between CoN_3C/rGO/PMS system and the other tested contaminants, we analyzed the orbital/electronic structure of these contaminants molecules through Fukui function and HOMO-LUMO region (**Figures R34-R38**, as **Supplementary Figs. 66-69** in the revised **Supplementary information**). The Fukui function data has been shown as **Tables R8-R12** (as **Supplementary Tables. 12-16** in the revised **Supplementary information**). In fact, the ETP process essentially involves the abstraction of electrons from the pollutant by the catalyst-PMS* complex. Therefore, the propensity of a pollutant to act as an electron donor governs its reactivity. We used the Fukui function analysis to identify sites most susceptible to nucleophilic attack, provides critical insights. Electron-rich compounds like Phe and 4-BP exhibit high f^- values on their

hydroxyl oxygen and adjacent carbon atoms, indicating readily available electron density for transfer, leading to their rapid degradation. For BPA, although individual atomic f^- values are moderate, its two phenolic rings constitute an extended, delocalized π-system. Combined with its high HOMO level, enables BPA to act as an exceptionally efficient and collective electron donor via the ETP pathway. In contrast, PNP, bearing a strong electron-withdrawing nitro group, displays a significantly lowered overall electron density and higher energy barrier for electron donation, resulting in its sluggish removal, which was verified by the evidence that it shows the lowest HOMO although it has similar maximum f^- value to Phe. For larger, more complex molecules like OFX and MB, while specific atoms (e.g., the piperazinyl N in OFX) show moderate f^- values, their overall reactivity is further hampered by steric hindrance and site distribution. Specifically, the electrophilic attack region and HOMO are mainly distributed on the rings within the OFX molecule. For MB, the similar regions are localized on the chloride counterion, rather than conjugated phenothiazine ring system of the cationic dye itself. This structure-activity relationship powerfully corroborates that the degradation is governed by an electron-transfer pathway rather than non-selective radical attack. Detailed discussion was added to the revised **manuscript**, which can be found below (**Line 361-366, Page 8**):

A proof-of-concept dual-chamber device successfully achieved ~40% BPA removal about 36 h via long-distance electron transfer, fundamentally eliminating the risk of SO_4^{2-} secondary pollution in the treated water (Supplementary Fig. 77). Although the macroscopic degradation kinetics are inherently limited by the mass transfer resistance and ohmic drop of the decoupled setup, this result robustly validates the practical environmental feasibility of the ETP mechanism.

Other detailed discussion in the revised **Supplementary information** can be found below (**Pages 69-70**):

The specific orbital/electronic interactions between CoN_3C/rGO/PMS system and the six tested contaminants were analyzed through Fukui function and HOMO-LUMO

region (Supplementary Figs. 64-69 and Supplementary Tables 11-16). In fact, the ETP process essentially involves the abstraction of electrons from the pollutant by the catalyst-PMS complex. Therefore, the propensity of a pollutant to act as an electron donor governs its reactivity. The Fukui function exhibit sites most susceptible to nucleophilic attack, provides critical insights. Electron-rich compounds like Phe and 4-BP exhibit high f^- values on their hydroxyl oxygen and adjacent carbon atoms, indicating readily available electron density for transfer, leading to their rapid degradation. For BPA, although individual atomic f^- values are moderate, its two phenolic rings constitute an extended, delocalized π-system. Combined with its high HOMO level, enables BPA to act as an exceptionally efficient and collective electron donor via the ETP pathway. In contrast, PNP, bearing a strong electron-withdrawing nitro group, displays a significantly lowered overall electron density and higher energy barrier for electron donation, resulting in its sluggish removal, which was verified by the evidence that it shows the lowest HOMO although it has similar maximum f^- value to Phe. For larger, more complex molecules like OFX and MB, while specific atoms (e.g., the piperazinyl N in OFX) show moderate f^- values, their overall reactivity is further hampered by steric hindrance and site distribution. Specifically, the electrophilic attack region and HOMO are mainly distributed on the rings within the OFX molecule. For MB, the similar regions are localized on the chloride counterion, rather than conjugated phenothiazine ring system of the cationic dye itself. This structure-activity relationship powerfully corroborates that the degradation is governed by an electron-transfer pathway rather than non-selective radical attack.*

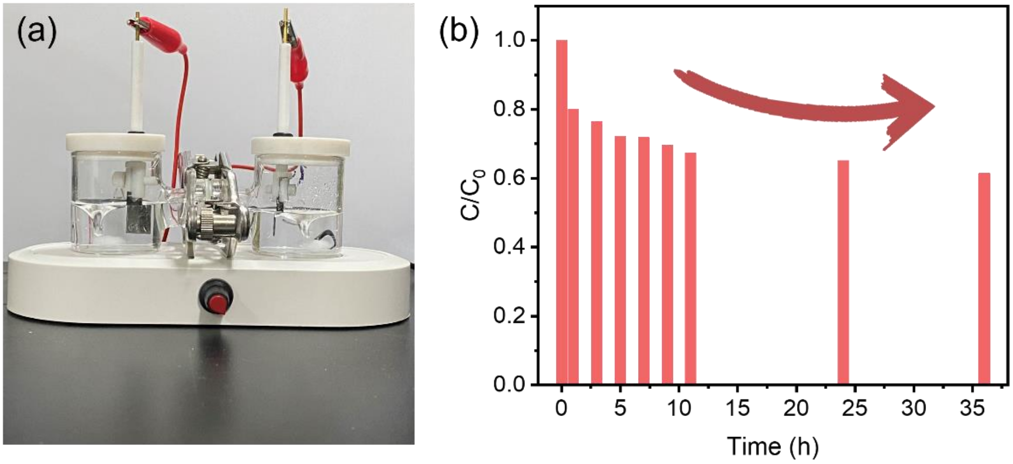

Figure R33. The performance of proof-of-concept dual-chamber device. (a) The image of dual-chamber device. (b) Degradation effect of BPA in dual-chamber device.

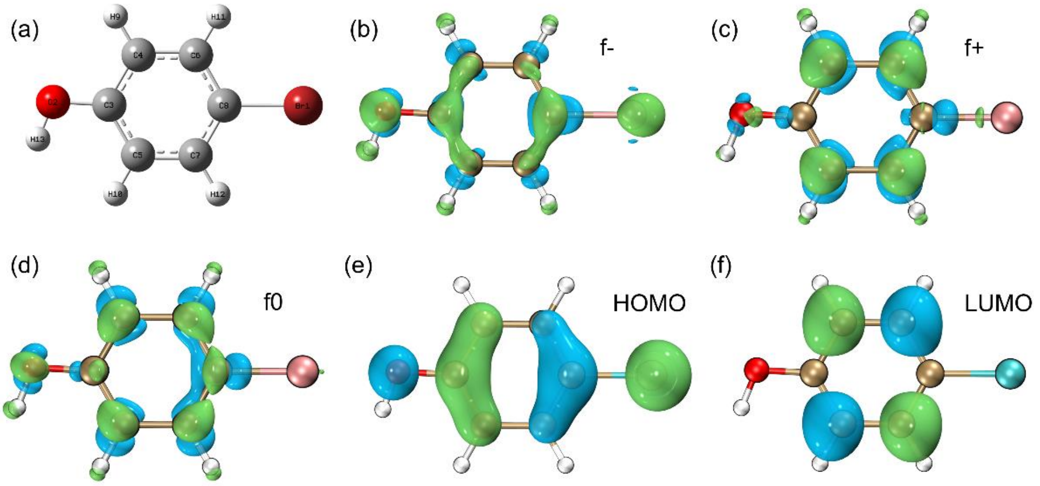

Figure R34. The structure and chemical properties of 4-BP. The (a) optimized chemical structure, (b) Fukui index (f^-) region, (c) Fukui index (f^+) region, (d) Fukui index (f^0) region, (e) HOMO region, (f) LUMO region of 4-BP.

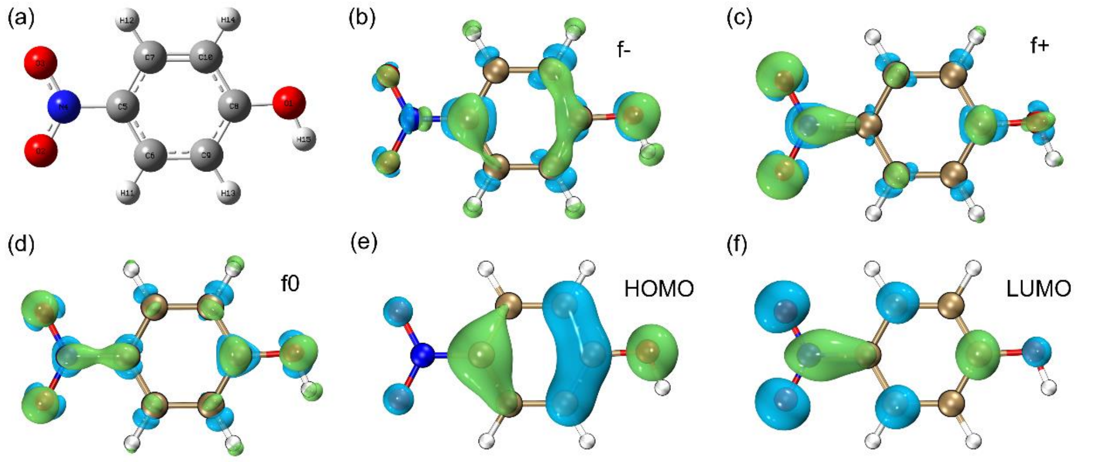

Figure R35. The structure and chemical properties of PNP. The (a) optimized chemical structure, (b) Fukui index (f^-) region, (c) Fukui index (f^+) region, (d) Fukui index (f^0) region, (e) HOMO region, (f) LUMO region of PNP.

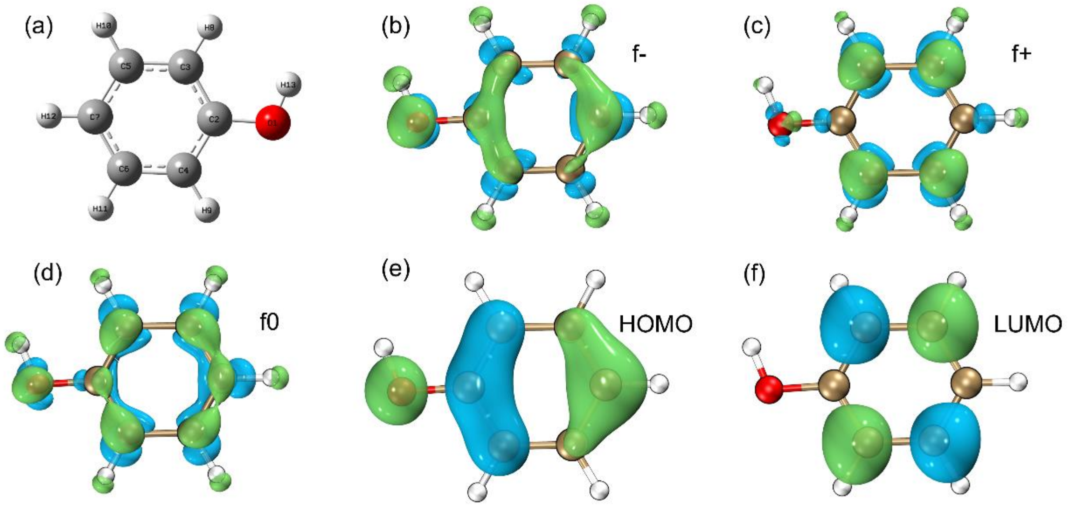

Figure R36. The structure and chemical properties of Phe. The (a) optimized chemical structure, (b) Fukui index (f^-) region, (c) Fukui index (f^+) region, (d) Fukui index (f^0) region, (e) HOMO region, (f) LUMO region of Phe.

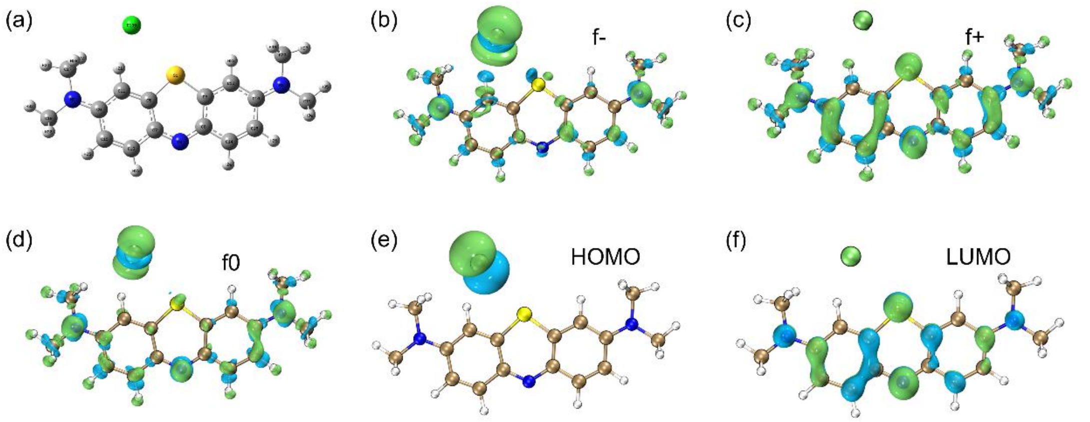

Figure R37. The structure and chemical properties of MB. The (a) optimized chemical structure, (b) Fukui index (f^-) region, (c) Fukui index (f^+) region, (d) Fukui index (f^0) region, (e) HOMO region, (f) LUMO region of MB.

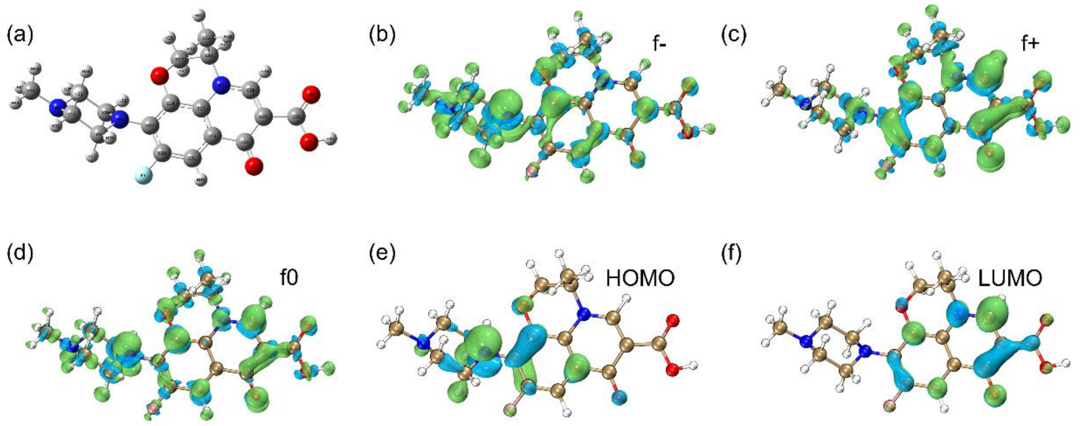

Figure R38. The structure and chemical properties of OFX. The (a) optimized chemical structure, (b) Fukui index (f^-) region, (c) Fukui index (f^+) region, (d) Fukui index (f^0) region, (e) HOMO region, (f) LUMO region of OFX.

Table R8. Natural population analysis (NPA) charge group and condensed Fukui index for electrophilic attack (f^-) at B3LYP/6–31G(d) level of 4-BP.

ATOM	No	Charge(0) (e/Å^3)	Charge(-1) (e/Å^3)	Charge(+1) (e/Å^3)	f^-
Br	1	-0.054	-0.1793	0.2126	0.2666
O	2	-0.1841	-0.2262	-0.0682	0.116
C	3	0.0759	0.0273	0.1609	0.085
C	4	-0.0493	-0.1729	0.0222	0.0716

C	5	-0.0628	-0.1809	0.0052	0.068
C	6	-0.0383	-0.1544	0.0153	0.0536
C	7	-0.0409	-0.1611	0.0189	0.0598
C	8	-0.011	-0.0482	0.0717	0.0827
H	9	0.0505	-0.0116	0.0914	0.041
H	10	0.0418	-0.0185	0.0821	0.0403
H	11	0.0483	-0.0096	0.084	0.0357
H	12	0.0477	-0.0117	0.0845	0.0368
H	13	0.1763	0.1469	0.2193	0.043

Table R9. Natural population analysis (NPA) charge group and condensed Fukui index for electrophilic attack (f^-) at B3LYP/6–31G(d) level of PNP.

ATOM	No	Charge(0) (e/Å^3)	Charge(-1) (e/Å^3)	Charge(+1) (e/Å^3)	f^-
O	1	-0.1693	-0.2279	-0.0304	0.1388
O	2	-0.2118	-0.3813	-0.1354	0.0764
O	3	-0.2101	-0.3803	-0.1364	0.0737
N	4	0.2458	0.1364	0.263	0.0171
C	5	0.0101	-0.0243	0.1248	0.1148
C	6	-0.0238	-0.0833	0.0347	0.0584
C	7	-0.0222	-0.078	0.0292	0.0514
C	8	0.0927	0.0143	0.1822	0.0895
C	9	-0.0585	-0.1047	0.0208	0.0792
C	10	-0.0452	-0.0933	0.0447	0.0898
H	11	0.0541	0.0201	0.091	0.0369
H	12	0.0545	0.0215	0.0905	0.0359
H	13	0.0459	0.0101	0.0895	0.0435
H	14	-0.2279	-0.0304	0.0998	0
H	15	-0.3813	-0.1354	0.1388	0.0586

Table R10. Natural population analysis (NPA) charge group and condensed Fukui index for electrophilic attack (f^{-}) at B3LYP/6–31G(d) level of MB.

ATOM	No	Charge(0) (e/Å^3)	Charge(-1) (e/Å^3)	Charge(+1) (e/Å^3)	f^-
S	1	0.1753	0.0833	0.1824	0.0071
N	2	-0.0012	-0.0407	0.0359	0.0371
N	3	-0.0208	-0.0503	0.0173	0.0381
N	4	-0.1179	-0.1958	-0.0953	0.0226
C	5	0.0014	-0.0219	0.0074	0.0061
C	6	-0.0061	-0.0302	0.0057	0.0117
C	7	0.0411	0.0146	0.0604	0.0192
C	8	0.0289	0.0116	0.0581	0.0292
C	9	0.0761	0.0377	0.0879	0.0118
C	10	0.0642	0.0276	0.083	0.0188
C	11	-0.0696	-0.0854	-0.0526	0.017
C	12	-0.0747	-0.0911	-0.0581	0.0166
C	13	-0.0229	-0.0642	-0.0023	0.0206
C	14	0.0833	0.1824	-0.0038	0.0575
C	15	-0.0407	0.0359	0.0071	0.092
C	16	-0.0503	0.0173	0.0371	0.0395
C	17	-0.1958	-0.0953	0.0381	0.0295
C	18	-0.0219	0.0074	0.0226	0.0779
C	19	-0.0302	0.0057	0.0061	0.0232
C	20	0.0146	0.0604	0.0117	0.0241
H	21	0.0116	0.0581	0.0192	0.0265
H	22	0.0377	0.0879	0.0292	0.0173
H	23	0.0276	0.083	0.0118	0.0385
H	24	-0.0854	-0.0526	0.0188	0.0365
H	25	-0.0911	-0.0581	0.017	0.0158
H	26	-0.0642	-0.0023	0.0166	0.0164
H	27	0.1824	-0.0038	0.0206	0.0413
H	28	0.0359	0.0071	0.0575	0
H	29	0.0173	0.0371	0.092	0.0495
H	30	-0.0953	0.0381	0.0395	0.0383
H	31	0.0074	0.0226	0.0295	0.0338
H	32	0.0057	0.0061	0.0779	0.0503
H	33	0.0604	0.0117	0.0232	0.0147
H	34	0.0581	0.0192	0.0241	0.0179
H	35	0.0879	0.0292	0.0265	0.0229
H	36	0.083	0.0118	0.0173	0.0233
H	37	-0.0526	0.0188	0.0385	0.0251
H	38	-0.0581	0.017	0.0365	0.0277
Cl	39	-0.0023	0.0166	0.0158	0.0164

Table R11. Natural population analysis (NPA) charge group and condensed Fukui index for electrophilic attack (f^-) at B3LYP/6–31G(d) level of Phe.

ATOM	No	Charge(0) (e/Å^3)	Charge(-1) (e/Å^3)	Charge(+1) (e/Å^3)	f^-
O	1	-0.1889	-0.2338	-0.0456	0.1434
C	2	0.0733	0.0209	0.1729	0.0996
C	3	-0.071	-0.1916	0.0112	0.0822
C	4	-0.0579	-0.1833	0.0335	0.0913
C	5	-0.0392	-0.1685	0.0358	0.075
C	6	-0.0367	-0.163	0.0293	0.066
C	7	-0.0567	-0.1176	0.0873	0.1439
H	8	0.0361	-0.0276	0.083	0.0469
H	9	0.0448	-0.0206	0.0932	0.0485
H	10	0.0418	-0.0255	0.089	0.0472
H	11	0.0424	-0.0237	0.088	0.0457
H	12	0.0387	-0.0077	0.0975	0.0588
H	13	0.1731	0.1418	0.2247	0.0516

Table R12. Natural population analysis (NPA) charge group and condensed Fukui index for electrophilic attack (f^-) at B3LYP/6–31G(d) level of OFX.

ATOM	No	Charge(0) (e/Å^3)	Charge(-1) (e/Å^3)	Charge(+1) (e/Å^3)	f^-
F	1	-0.0905	-0.1223	-0.0708	0.0197
O	2	-0.117	-0.1407	-0.0892	0.0278
O	3	-0.2756	-0.3583	-0.2385	0.0371
O	4	-0.1596	-0.1838	-0.1461	0.0134
O	5	-0.3046	-0.3493	-0.2713	0.0333
N	6	-0.0671	-0.0769	0.0318	0.0989
N	7	-0.0005	-0.0337	0.0138	0.0143
N	8	-0.1002	-0.1024	-0.0602	0.04
C	9	-0.01	-0.0137	0.0085	0.0185
C	10	-0.0085	-0.0136	0.0091	0.0176
C	11	0.039	0.0317	0.0443	0.0053
C	12	0.0247	-0.0145	0.0444	0.0197
C	13	0.0197	0.0113	0.0345	0.0148
C	14	-0.1223	-0.0708	0.0977	0.0549
C	15	-0.1407	-0.0892	0.0197	0.0318
C	16	-0.3583	-0.2385	0.0278	0.0318
C	17	-0.1838	-0.1461	0.0371	0.0237
C	18	-0.3493	-0.2713	0.0134	0.0826
C	19	-0.0769	0.0318	0.0333	0.0447
C	20	-0.0337	0.0138	0.0989	0.0098
C	21	-0.1024	-0.0602	0.0143	0.0333
C	22	-0.0137	0.0085	0.04	0.0022
C	23	-0.0136	0.0091	0.0185	0.0037
C	24	0.0317	0.0443	0.0176	0.0051
C	25	-0.0145	0.0444	0.0053	0.0074
C	26	0.0113	0.0345	0.0197	0.0392
H	27	-0.0708	0.0977	0.0148	0.0084
H	28	-0.0892	0.0197	0.0549	0.0372
H	29	-0.2385	0.0278	0.0318	0.0258
H	30	-0.1461	0.0371	0.0237	0.0258
H	31	-0.2713	0.0134	0.0826	0.0599
H	32	0.0318	0.0333	0.0242	0.0188
H	33	0.0138	0.0989	0.0447	0.039
H	34	-0.0602	0.0143	0.0098	0.0544
H	35	0.0085	0.04	0.0333	0.0238
H	36	0.0091	0.0185	0.0022	0.0211
H	37	0.0443	0.0176	0.0037	0.0111
H	38	0.0444	0.0053	0.0051	0.0113
H	39	0.0345	0.0197	0.0074	0.0063
H	40	0.0977	0.0197	0.0197	0.0392

H	41	0.0197	0.0549	0.0148	0.0084
H	42	0.0278	0.0318	0.0372	0
H	43	0.0371	0.0237	0.0258	0.0121
H	44	0.0134	0.0826	0.0258	-0.0041
H	45	0.0333	0.0242	0.0599	0.0456
H	46	0.0989	0.0447	0.0188	0.0108

Responses to the Reviewers' Comments

We thank the editor and all reviewers for their time spend making their constructive remarks and useful suggestions, which has significantly raised the quality of the manuscript. We have read all the comments and recommendations carefully, and tried our best to revise the manuscript accordingly. Each suggested revision and comment were responded point by point. The comments are in **black**, the responses are in **blue**, and the corresponding revised texts in the revised manuscript and revised Supplementary information are in **red**.

Reviewer #1:

The authors addressed all comments. The paper has been improved and is ready for publication.

Response: We gratefully appreciate for your approval of our revised content. Thank you for your valuable comments to improve the quality of our manuscript.

Reviewer #2:

The authors have provided comprehensive and convincing responses to each of my comments. All the necessary experiments, characterizations, data analyses, and discussions have been properly supplemented and revised. The scientific quality and presentation of the manuscript are now satisfactory. I recommend to accept the manuscript.

Response: We sincerely thank you for your valuable feedback that has significantly improved the quality of our manuscript. Thank you again for your recognition of our work.

Reviewer #3:

Dear authors, first of all, I thank you for the extensive work you have done to address my requests and those of my fellow reviewers, filling the gaps and uncertainties identified.

Regarding the implementation of the new section I requested, I still have some concerns regarding the XPS measurements.

First, I kindly ask you to switch from the "line + symbol" representation for the fit curves to simple lines, since the chosen circles almost entirely cover the "raw" signal of the experimental data and the quality of the fits is difficult to assess. Regarding the Co2p regions, however, I believe the reported fits are flawed in terms of the choice of the number of curves to use to fit the components due to the two oxidation states +2 and +3, which involve several satellites and multiple splitting (have a look at this paper for an example about Co2p correct deconvolution procedure: Applied Surface Science 257 (2011) 2717 – 2730). Furthermore, especially for images in SI 6 and 27, the signal quality of the Co2p regions is so poor, with a very low signal-to-noise ratio, that you are essentially trying to fit the noise itself. Furthermore, judging by the position and relative intensities of the different components, no constraint appears to have been applied on the area ratio of the Co2p3/2 and Co2p1/2 components, as the areas of the latter do not appear to be in the 1:2 ratio, as one would expect. At this point, I would recommend fitting only those curves where it makes sense to interpret a clear and clean signal and ignoring regions where noise dominates the experimental signal. Finally, in Table 3 of the supplementary material, I would round the relative atomic concentration values to the first decimal place and verify that the total for each sample is equal to 100% and not 100.01% as in some rows of the table.

The work cannot therefore be published in its current state, as it still requires minor revisions.

Response: We gratefully appreciate for your careful concern and constructive suggestions throughout the review process. We fully agree with your highly professional comments, which have significantly improved the rigorousness of our

characterization results. We have carefully revised the XPS figures and data processing methods according to your instructions.

Following your suggestion, we have changed the plotting style of all XPS figures in the revised **Supplementary information** (**Figure R1**, as the **Supplementary Fig. 3**; **Figure R2**, as the **Supplementary Fig. 4**; **Figure R3**, as the **Supplementary Fig. 5**; **Figure R4**, as the **Supplementary Fig. 6**; **Figure R5**, as the **Supplementary Fig. 25**; **Figure R6**, as the **Supplementary Fig. 27**; **Figure R7**, as the **Supplementary Fig. 35**). The experimental "raw" data are now exhibited clearly without being covered, while all the individual component fit curves and the cumulative fit lines are plotted as simple solid lines without symbols.

We sincerely thank the reviewer for pointing out the highly valuable reference (*Applied Surface Science 257 (2011) 2717–2730*). We realize that our previous fitting lacked thermodynamic constraints and oversimplified the multiplet splitting. In the revised manuscript, we have re-fitted the high-quality Co 2p spectra strictly following the procedure described in the recommended paper. And we strictly ensure the area ratio of the Co $2p_{3/2}$ to Co $2p_{1/2}$ components are fixed at exactly 2:1 (**Figure R5**). Additionally, the Co loading in some samples was extremely low, leading to a very poor signal-to-noise ratio. Following your advice, we have removed the multi-component fits for these noisy spectra (**Figures R4-R6**). We now only present the raw signal to indicate the existence of Co species.

Finally, we have revised **Supplementary Table 3** in the revised **Supplementary information** as you can see in the **Table R1**. All relative atomic concentration values have been rounded to the first decimal place. Furthermore, we have manually verified every row to ensure that the sum of the atomic concentrations exactly equals 100.0%.

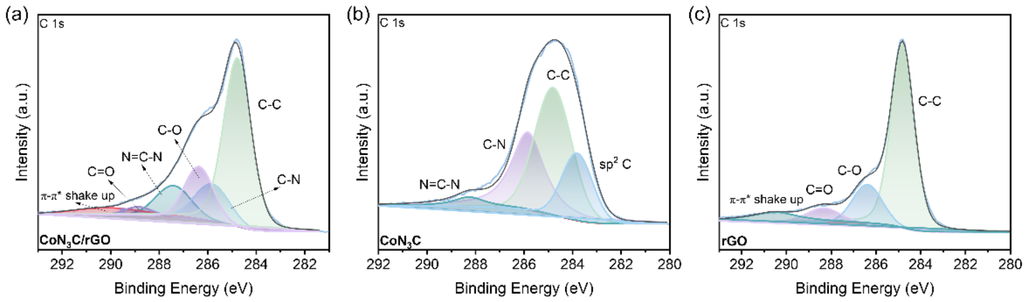

Figure R1. XPS characterization of catalysts. High-resolution XPS spectra of C 1s for (a) CoN_3C/rGO, (b) CoN_3C, (c) rGO.

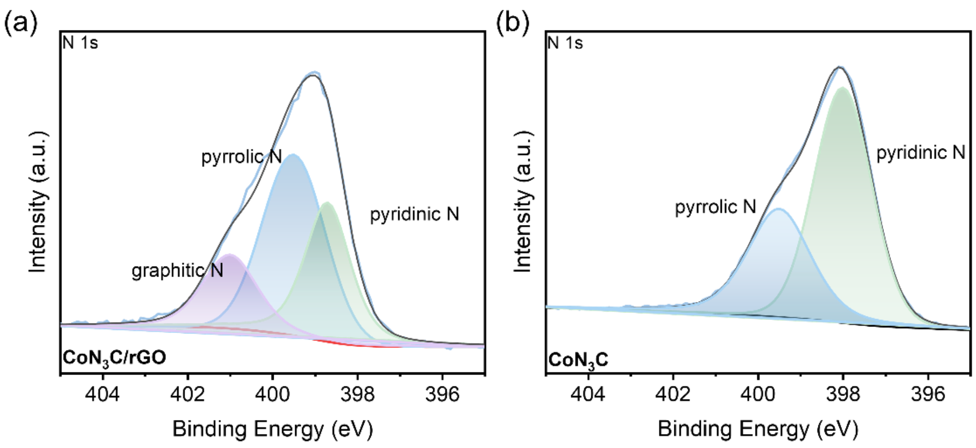

Figure R2. XPS characterization of catalysts. High-resolution XPS spectra of N 1s for (a) CoN_3C/rGO, (b) CoN_3C.

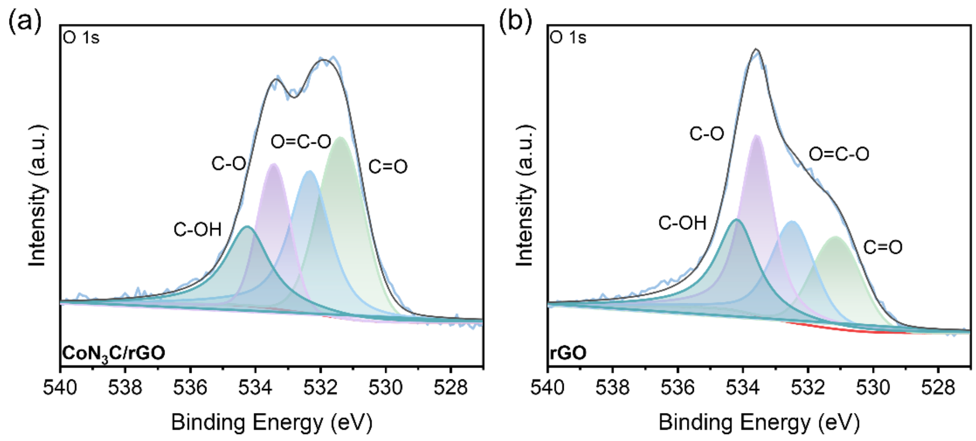

Figure R3. XPS characterization of catalysts. High-resolution XPS spectra of O 1s for (a) CoN_3C/rGO, (b) rGO.

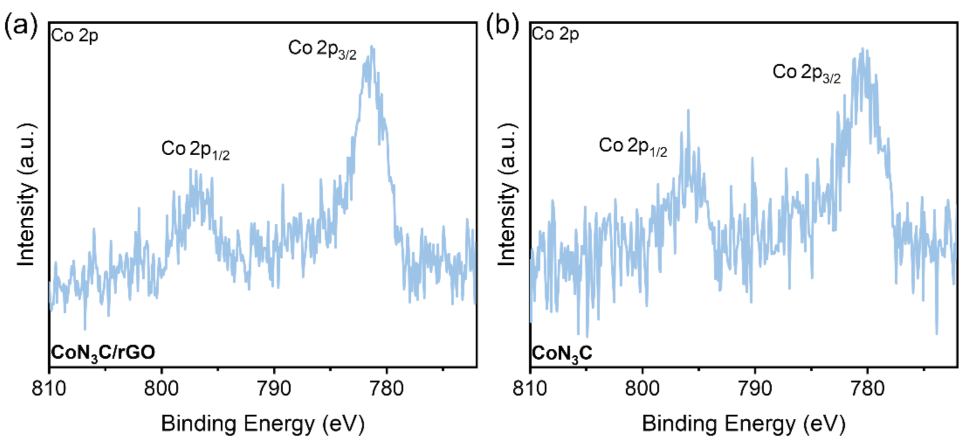

Figure R4. XPS characterization of catalysts. High-resolution XPS spectra of Co 2p for (a) CoN_3C/rGO, (b) CoN_3C.

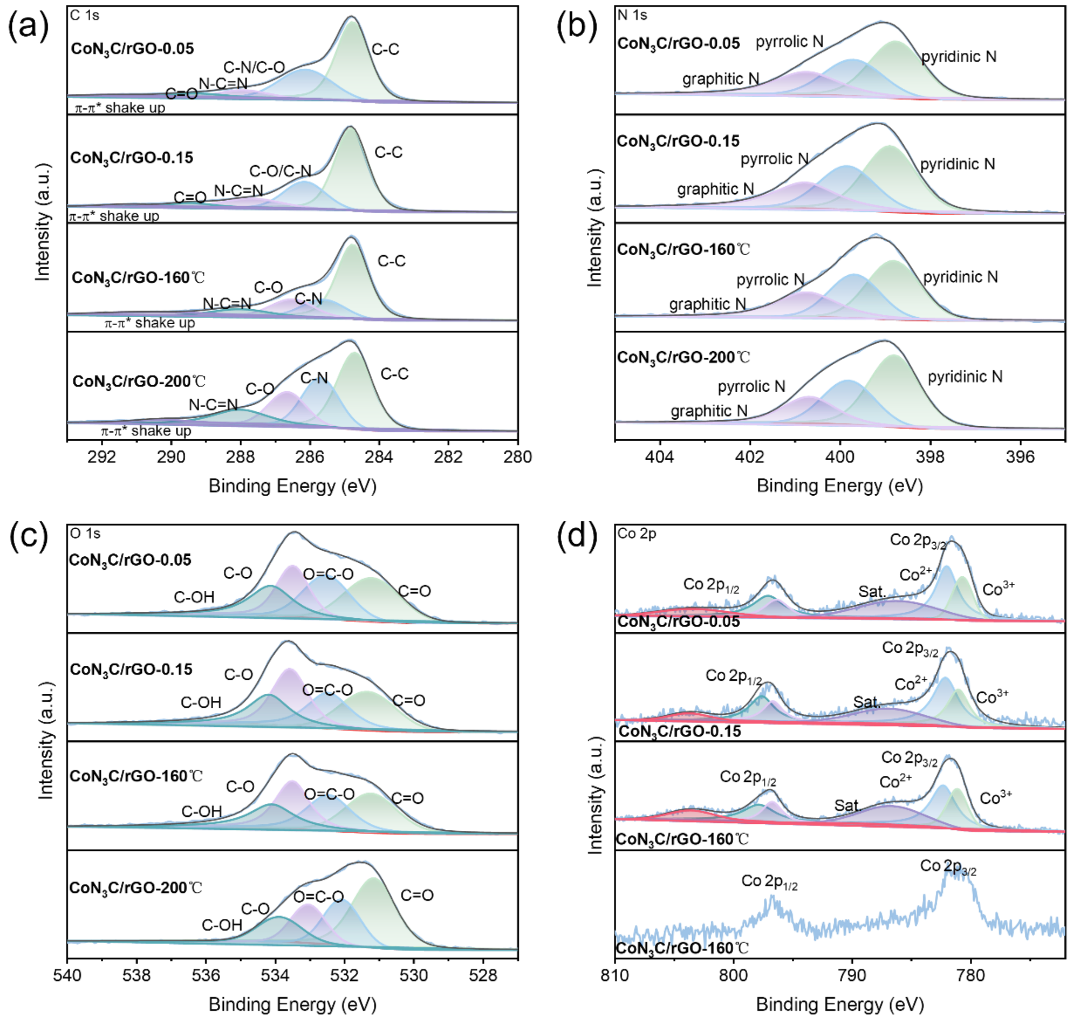

Figure R5. The chemical state of elements on catalysts with different synthesis condition. The high-resolution XPS (a) C 1s, (b) N 1s, (c) O 1s, (d) Co 2p spectra of CoN_3C/rGO-0.05g, CoN_3C/rGO-0.15g, CoN_3C/rGO-160 °C, and CoN_3C/rGO-200 °C.

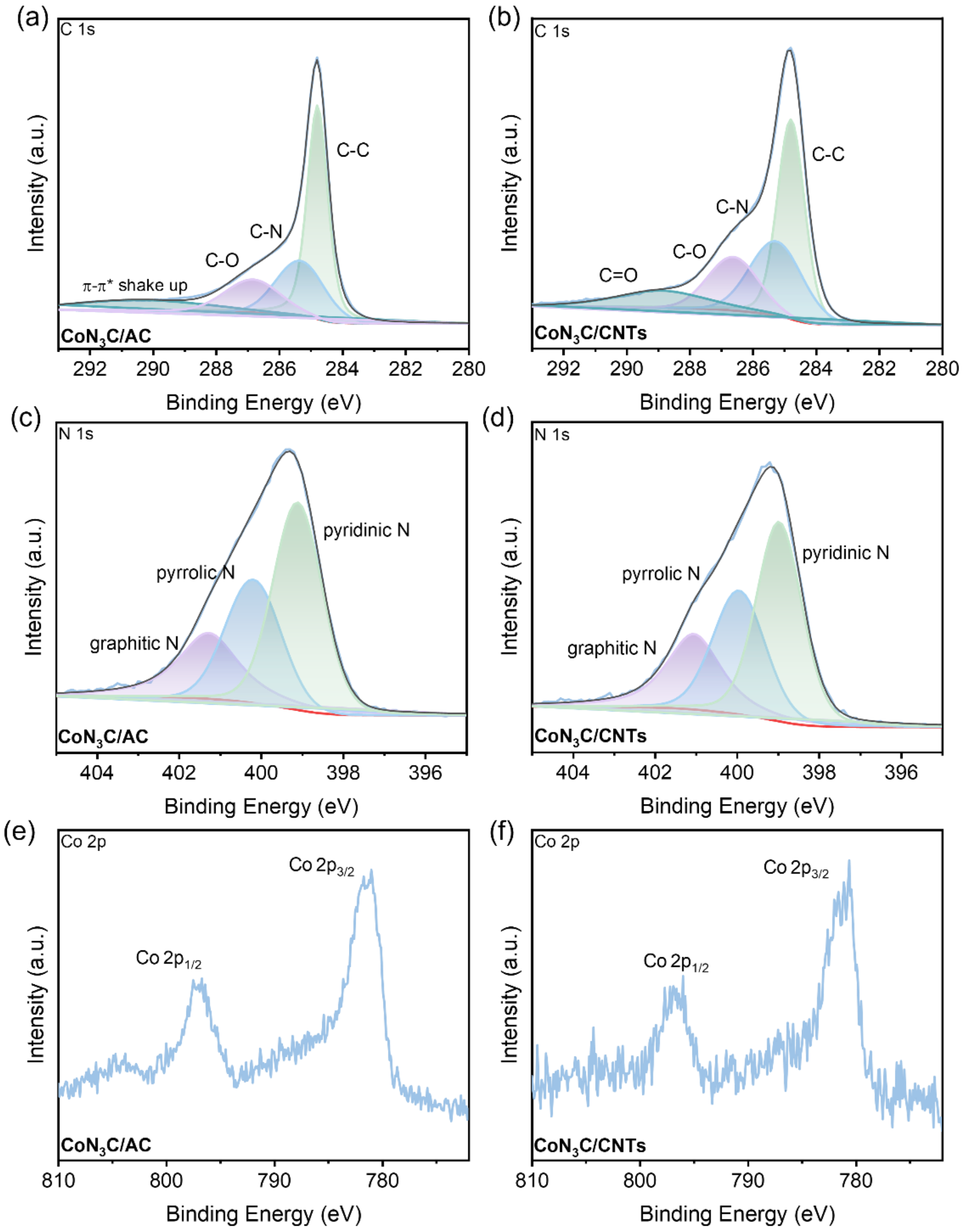

Figure R6. XPS characterization of catalysts with different sea carrieres. High-resolution XPS spectra of C 1s for (a) CoN_3C/AC, (b) CoN_3C/CNTs; N1s for (c) CoN_3C/AC, (d) CoN_3C/CNTs; Co 2p for (e) CoN_3C/AC, (f) CoN_3C/CNTs.

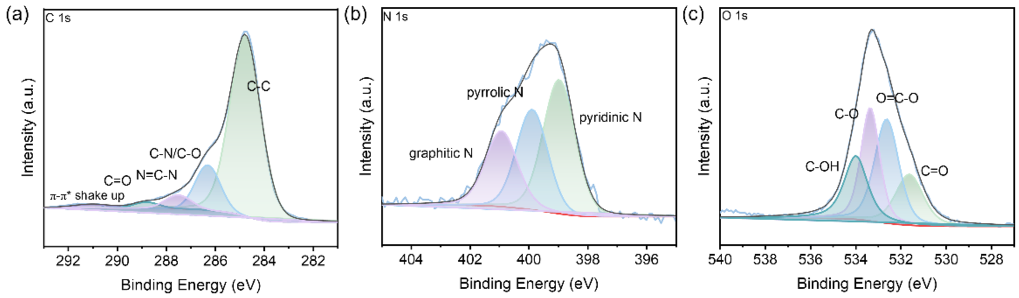

Figure R7. The chemical state of used CoN_3C/rGO. High-resolution XPS spectra of (a) C 1s, (b) N 1s, (c) O 1s for used CoN_3C/rGO.

Table R1. The elements content (%) of catalysts from XPS.

Catalysts \ Elements	Co	N	C	O
CoN_3C/rGO	0.4	19.1	71.1	9.4
CoN_3C	0.4	31.0	61.3	7.3
rGO	/	/	88.0	12.0
Used CoN_3C/rGO	0.3	14.1	79.3	6.3

Responses to the Reviewers' Comments

We thank the editor and all reviewers for their time spend making their constructive remarks and useful suggestions, which has significantly raised the quality of the manuscript. We have read all the comments and recommendations carefully, and tried our best to revise the manuscript accordingly. Each suggested revision and comment were responded point by point. The comments are in **black**, the responses are in **blue**.

Reviewer #3:

I am satisfied with the revisions undertaken by the authors. The manuscript has benefited substantially from the changes implemented, and the authors have carefully and convincingly addressed the concerns previously raised. As a result, the overall quality, clarity, and impact of the work have improved markedly. In its current form, I consider the manuscript to meet the high standards of the journal and I am pleased to recommend it for publication.

Response: We are happy that you can satisfy with our work. We gratefully appreciate for your approval of our revised content. Thank you for your valuable comments to improve the quality of our manuscript.
